# Photochemical skeletal editing: one-step transformation of diaryl dithiophenes into regiodefined helicenes

**DOI:** 10.1039/d6sc01717g

**Published:** 2026-03-25

**Authors:** Xiaoli Shi, Ling Mei, Chenxi Dong, Chunmei Zhao, Chen Chen, Yimin Xu, Wan Xu, Chunli Li, Guangxia Wang, Zhiying Ma, Hua Wang

**Affiliations:** a Institute of Nanoscience and Engineering, Henan University Kaifeng Henan 475004 China wangguangxia@henu.edu.cn mazy11@henu.edu.cn hwang@henu.edu.cn

## Abstract

Skeletal editing is reshaping synthetic design to allow the direct, atom-level manipulation of molecular frameworks. While single-atom insertion or deletion has been achieved in simple aromatic systems, strategies for editing *S*-heterocycles within complex, functional molecules remain underdeveloped due to the inertness of C–S bonds and the lack of mild, direct methods to reconstruct the entire aromatic skeleton. We report a photochemical skeletal editing approach that directly converts readily available diaryl dithiophenes into regiodefined π-extended helicenes, bypassing the multistep sequences and regioselectivity limitations of classical syntheses. This transformation consists of the formation of two benzene rings and a ring opening of thiophene through cascade-initiated steps under light. The process involves regioselective photocyclization, followed by C–S bond cleavage, a second annulation and desulfurization, all occurring in a one-pot synthetic operation. The reaction proceeds under mild conditions, displays broad substrate scope, and enables the efficient regioselective synthesis of diverse benzo-fused helicenes. The resulting π-extended helicenes exhibit good photophysical and chiroptical properties. Their performance in terms of circularly polarized luminescence (CPL) shows attractive luminescence dissymmetry factors (|*g*_lum_|) reaching magnitudes of the order of 10^−3^.

## Abstract 2

A novel photochemical skeletal editing strategy enables the efficient, regioselective one-step synthesis of diverse π-extended multiple [*n*]helicenes from diaryl dithiophenes under mild conditions with broad substrate scope.

## Graphical abstract

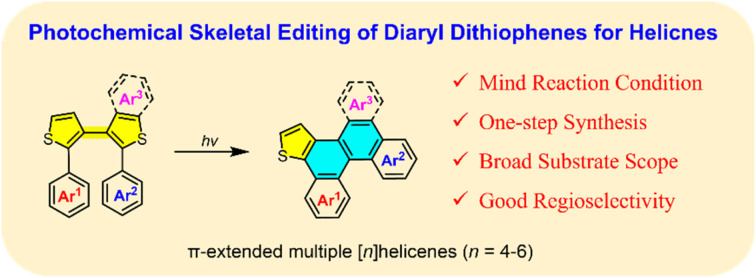

## Introduction

Skeletal editing^1–3^ is catalyzing a paradigm shift in synthetic chemistry, moving beyond traditional bond formation to allow the direct, atom-level rewriting of molecular frameworks. While transformative advances have been achieved in editing heterocycles,^4–9^*via* single-atom insertion or deletion, a more profound and challenging frontier lies in developing general and selective methods to directly convert robust heteroaromatic rings, particularly within a complex molecular skeleton, into new arenes or heterocycles. Such a capability would offer the most concise path to complex, functional architectures from abundant heteroaromatic precursors.

**Fig. 1:**
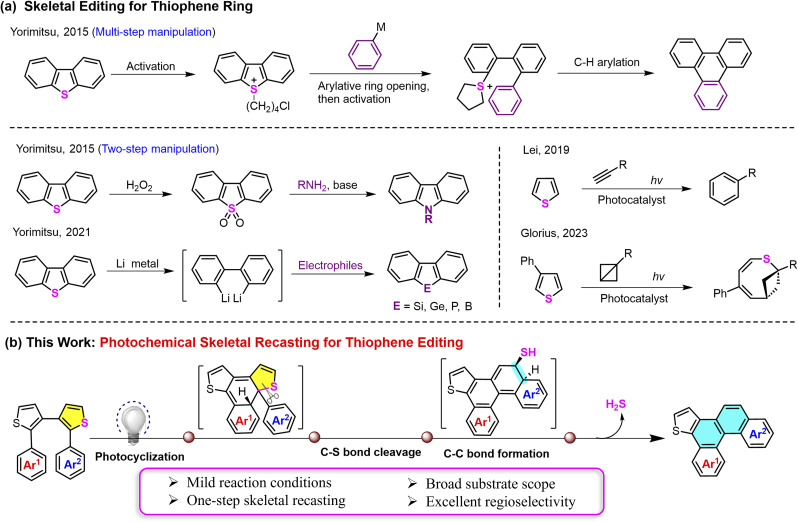
(a) Skeletal editing for a thiophene ring; (b) photochemical skeletal recasting for thiophene editing in our work.

Despite its transformative potential, the field of aromatic ring editing, especially concerning robust *S*-heterocycles like thiophenes, is still in its infancy. Thiophenes are ubiquitous in pharmaceuticals^10,11^ and organic materials,^12–14^ yet their endocyclic modification is notoriously difficult due to the inert C–S bonds and the requirement for high selectivity in polycyclic systems. Seminal work by Yorimitsu and co-workers has established “aromatic metamorphosis” methodologies,^15,16^ transforming dibenzothiophenes into other arenes, such as triphenylenes,^17^ carbazoles^18^ and heteroles^19^*via* multistep, stoichiometric activation sequences (Fig. 1a). Recent photocatalytic approaches have enabled one-step thiophene editing but rely on added reagents (*e.g.*, alkynes or bicyclobutane) to drive the conversion (Fig. 1a, Lei^20^ and Glorious^21^). Therefore, a general and direct strategy for the selective editing of a thiophene ring within heteroaromatic systems, which does not depend on pre-functionalization or stoichiometric exogenous reagents to seamlessly deconstruct and reconstruct the skeleton, remains elusive. Developing such “heterocycle exchange” strategies would hold great promise for opening novel pathways to diverse polycyclic aromatic hydrocarbons and functional π-systems that are difficult to access by conventional logic.

Helicenes,^22–24^ an important class of chiral polycyclic arenes with helical molecular skeletons, have attracted considerable interest in chiral recognition,^25,26^ asymmetric catalysis,^27,28^ and circularly polarized luminescence.^29–31^ However, their classical synthesis *via* Mallory photocyclization^32–36^ typically relies on the stepwise cyclization of stilbene-type precursors and suffers from limitations such as restricted structural diversity and unpredictable regioselectivity.^37–39^ Consequently, a method that could directly construct the helical framework from simple, readily available precursors through skeletal editing would represent a revolutionary shortcut.

Here, we introduce a photochemical aromatic ring editing strategy for the efficient synthesis of helicenes. We designed readily accessible diaryl dithiophenes as key precursors and exploited their photochemical reactivity to develop an unprecedented photoinduced “selective thiophene-to-benzene ring exchange” reaction. This transformation undergoes a cascade sequence of photocyclization with C–S bond cleavage of the thiophene ring and C–C bond formation for the construction of two new benzene rings to create π-extended helicenes under irradiation *via* one-step skeletal recasting (Fig. 1b). This process is distinct from any known helicene synthesis or skeletal editing reaction in three aspects: (1) it establishes aromatic ring editing as a viable and powerful new dimension in skeletal manipulation, moving beyond single-atom edits to whole-ring transmutation. (2) It provides the first general, one-step route to helicenes *via* skeletal editing, offering unparalleled regiocontrol and step economy from simple precursors. (3) It directly channels skeletal editing into the creation of sophisticated chiral materials with compelling photophysical and chiroptical properties, bridging a critical gap between methodological innovation and functional application. This strategy provides a fresh perspective for the synthesis of complex fused-ring molecules and is expected to accelerate the development of function-oriented, tailor-made helicene materials.

## Results and discussion

### Method development

**Fig. 2:**
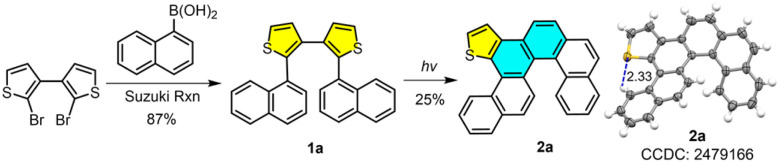
Synthetic route to 2a.

We initiated our investigation using 2,2′-dibromo-3,3′-bithiophene^40^ as the starting material. Suzuki coupling with 1-naphthylboronicacid afforded 2,2′-di(naphthalen-1-yl)-3,3′-bithiophene (1a) in 87% yield. Subsequent oxidative photocyclization of 1a, employing iodine and propylene oxide under irradiation with a medium-pressure Hg lamp, generated 2a in 25% yield (Fig. 2). Single-crystal X-ray diffraction analysis unambiguously confirmed 2a as an asymmetric π-extended helicene comprising fused carbo[5]helicene and thia[4]helicene subunits. Notably, precursor 1a contains two discrete thiophene rings, while product 2a retains only one thiophene unit within an entirely reformed polycyclic framework. This skeletal change confirms a reaction pathway that fundamentally diverges from classical photocyclization, which typically preserves the original heterocyclic core. Critically, the X-ray structure of 2a thus serves as definitive proof of concept, validating the successful reconstruction of the diaryl dithiophene skeleton into a complex, tailor-made helical architecture. This transformation provides direct structural evidence for our proposed skeletal editing strategy.

### Substrate scope

**Fig. 3:**
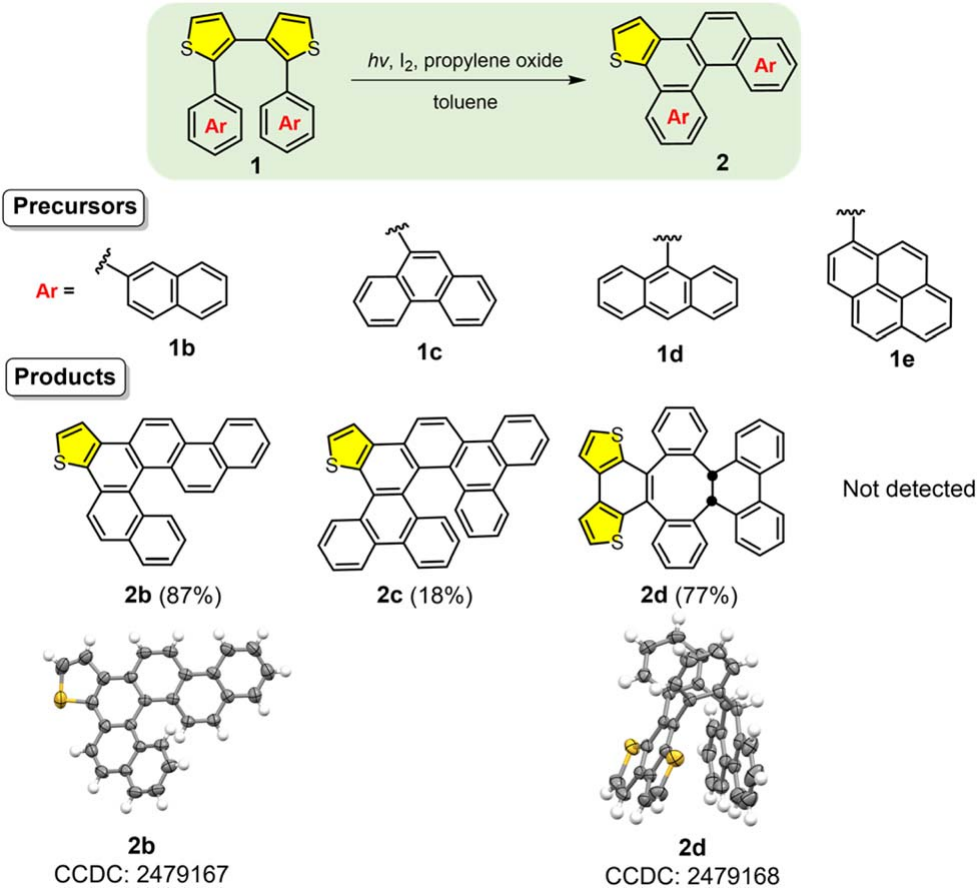
Substrate scope with symmetric diaryl dithiophene precursors and scope of the synthesis of helicenes.

To gain more insights into this intriguing photochemical skeletal editing strategy, we evaluated its substrate scope and limitations. A series of symmetric diaryl dithiophene precursors bearing 2-naphthyl, 9-phenanthryl, 9-anthryl and 1-pyrenyl substituents were investigated (Fig. 3). Photocyclization of 1b and 1c afforded products 2b and 2c in 87% and 18% yield, where the higher yield of 2b compared to 2a is attributed to the greater reactivity of the α-position in 2-naphthyl *versus* the β-position in 1-naphthyl. The lower yield of 2c is likely due to steric hindrance from the 9-phenanthryl group. X-ray crystallography confirmed 2b as an asymmetric π-extended [5]helicene. Interestingly, precursor 1d with a 9-anthryl group exhibited distinct behavior, undergoing anthracene [4 + 4] photodimerization^41–44^ to yield 2d, whose structure was confirmed by X-ray crystallography. In contrast, precursor 1e with a 1-pyrenyl group showed no reactivity under identical conditions, presumably due to severe steric constraints. These results validate the methodology and underscore the varied reactivity and mechanistic pathways among different aryl substituents.

**Fig. 4:**
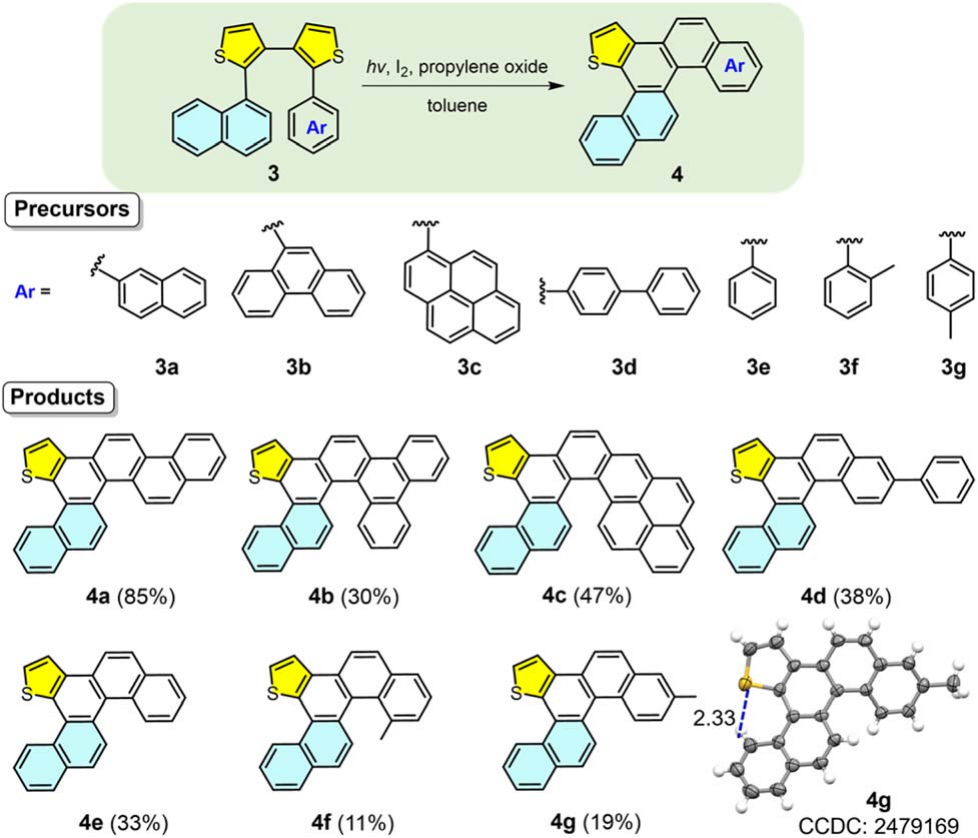
Substrate scope with asymmetric diaryl dithiophene precursors and scope of the synthesis of helicenes.

We next explored unsymmetric diaryl dithiophene precursors to elucidate the observed reactivity patterns. The synthesis of precursors 3a–3g is described in Scheme S2 according to two Suzuki reactions. The first one is the reaction of 2,2′-dibromo-3,3′-bithiophene with 1-naphthaleneboronic acid to offer 9, which then reacts with second aryl boronic acids, including 2-naphthyl, 9-phenanthryl, 1-pyrenyl, 4-biphenyl, phenyl, 2-methylphenyl or 4-methylphenyl groups. Photochemical reactions of 3a–3g afforded helicenes 4a–4g in yields of 85%, 30%, 47%, 38%, 33%, 11% and 19%, respectively (Fig. 4). The modest to low yields for 4b–4g are primarily attributed to the formation of large amounts of polar and inseparable oligomers under the irradiation conditions, rather than a loss of regioselectivity. Each product displayed a characteristic doublet peak near *δ* = 9.3 ppm in the ^1^H NMR spectrum. Full assignment of proton signals for 4g was achieved using ^1^H–^1^H NOESY spectra (Fig. S3 and S4), with the signal at 9.3 ppm corresponding to a terminal benzene ring proton (H_i_) of the thia[4]helicene skeleton. The remarkable downfield shift of proton H_i_ is attributed primarily to the formation of an intramolecular S⋯H interaction between H_i_ and the adjacent sulfur atom of the thiophene ring. This assignment is consistent with the reported NMR data for thia[4]helicenes^45^ and is further supported by the single-crystal X-ray structures of 2a and 4g. The S⋯H distance is 2.33 Å (Fig. 2 and 4), which is significantly shorter than the sum of their van der Waals radii, 2.95 Å for the two atoms, confirming the presence of the intramolecular S⋯H interactions. Structural analysis indicated that the thiophene linked to the 1-naphthyl group was retained, while the adjacent thiophene bearing other aryl groups underwent desulfurization and reconstruction. X-ray analysis of 4g confirmed its structure as a π-extended thia[4]helicene, supporting the proposed photochemical skeletal editing mechanism (*vide infra*) and demonstrating high selectivity for unsymmetric diaryl dithiophenes.

**Fig. 5:**
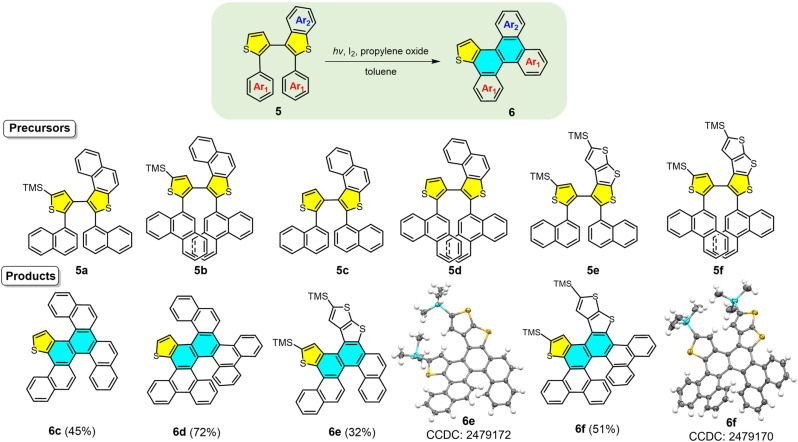
Substrate scope of skeletal expansion for diaryl dithiophene precursors and scope of the synthesis of helicenes.

To further assess the versatility and scalability of this reaction, we investigated substrates with increased complexity by modifying the dithiophene unit. Precursors 5a–5e were prepared by fusing benzene rings or thiophene rings onto bithiophene units bearing 2-naphthyl or 9-phenanthryl groups (Fig. 5). Precursors 5a and 5b, functionalized with TMS groups, did not undergo the photochemical reaction. However, without TMS groups, 5c and 5d reacted successfully to afford 6c and 6d in 45% and 72% yields, respectively. In contrast, TMS-containing precursors 5e and 5f worked to afford 6e and 6f in 32% and 51% yields, respectively. Compounds 6c–6f exhibited the characteristic doublet peak located near *δ* = 9.3 ppm in their ^1^H NMR spectra, confirming the presence of the thia[4]helicene moiety in their target molecular structures. X-ray diffraction analysis of 6e and 6f established their structures as π-extended multiple helicenes fused with carbo[5]/[6]helicene, thia[4]/[5]helicene subunits. Analysis revealed that the photochemical process retained the monothiophene ring, while the adjacent fused thiophene ring underwent desulfurization and reconstruction. The successful synthesis of helicenes 6c–6f demonstrates the applicability and selectivity of the photochemical skeletal editing strategy to multiple helicene molecular frameworks.

**Fig. 6:**
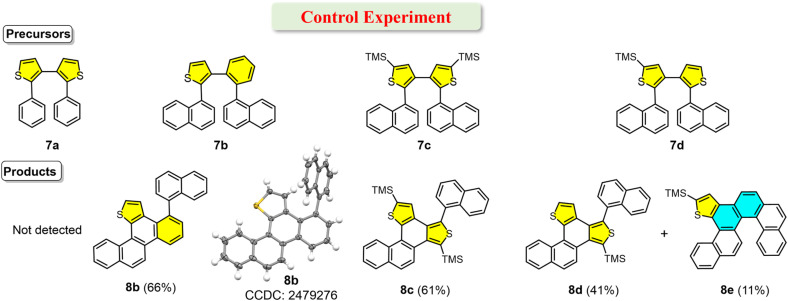
Substrate scope with symmetric/asymmetric precursors and scope of the synthesis of helicenes.

Control experiments were conducted to delineate the substrate scope in photochemical skeletal editing for the synthesis of helicene (Fig. 6). Symmetric diaryl dithiophene 7a with phenyl substituents did not react under irradiation, unlike unsymmetric analogue 3e, indicating that at least one aryl group must be a fused aromatic system (Ar_1_ or Ar_2_, Fig. 1b). Unsymmetric precursor 7b bearing two 1-naphthyl groups underwent the photochemical reaction, affording product 8b in 66% yield. X-ray crystallography confirmed that 8b possesses a thia[4]helicene skeleton, consistent with the traditional photocyclization mechanism. The photochemical reaction of 7c (with two TMS groups) afforded 8c in 61% yield, while 7d (with one TMS group) yielded both 8d (41%) and 8e (11%). ^1^H NMR spectra of 8b–8e showed characteristic downfield doublets, confirming the thia[4]helicene skeleton (Fig. S1). The ^1^H NMR spectrum of 8e closely resembled that of 2a, suggesting a similar fused carbo[5]/thia[4]helicene skeleton. Aside from the thiophene signals (*δ* = 6.19 ppm, singlet for 8c; *δ* = 6.19 and 6.06 ppm, doublets for 8d), ^1^H NMR spectra of 8c and 8d were nearly identical, indicating structural similarity. Furthermore, the UV-vis and fluorescence spectra of 8e closely resemble those of 2a, just as the spectra of 8c and 8d are similar to each other (Fig. S10), further supporting their structural similarity. These results indicate that replacement of one thiophene in the bithiophene by benzene or the presence of two TMS substituents prevents photochemical cyclization and desulfurization, although traditional photocyclization can still occur. With only one TMS substituent on the bithiophene, both photochemical skeletal editing and traditional photocyclization pathways are accessible, but the traditional pathway dominates. These substrate scope and control experiments establish three essential requirements for successful helicene construction *via* photochemical skeletal editing: (1) the precursor must contain two thiophene rings; (2) at least one of the two aryl substituents must be a fused aromatic ring system; (3) alkyl substituents (*e.g.*, TMS groups) cannot be attached to both thiophene rings simultaneously.

### Mechanistic study

To elucidate the mechanism of the photochemical skeletal editing, several mechanistic experiments were conducted. A deuterium–hydrogen labeling study was carried out at the α-position of both thiophene rings to trace the transformation of diaryl dithiophene precursors into the products. Comparison of the ^1^H NMR and ^1^H–^1^H NOESY spectra between labeled compound 2a′ and unlabeled 2a (Fig. S5 and S6) revealed complete retention of the deuterium label throughout the photochemical process, indicating that no hydrogen–deuterium exchange occurred. Additionally, the reaction was monitored using lead acetate test paper, which turned black, confirming the release of hydrogen sulfide during the reaction. The generation of hydrogen sulfide indicates that the thiophene ring undergoes ring opening and desulfurization during the reaction process.

**Fig. 7:**
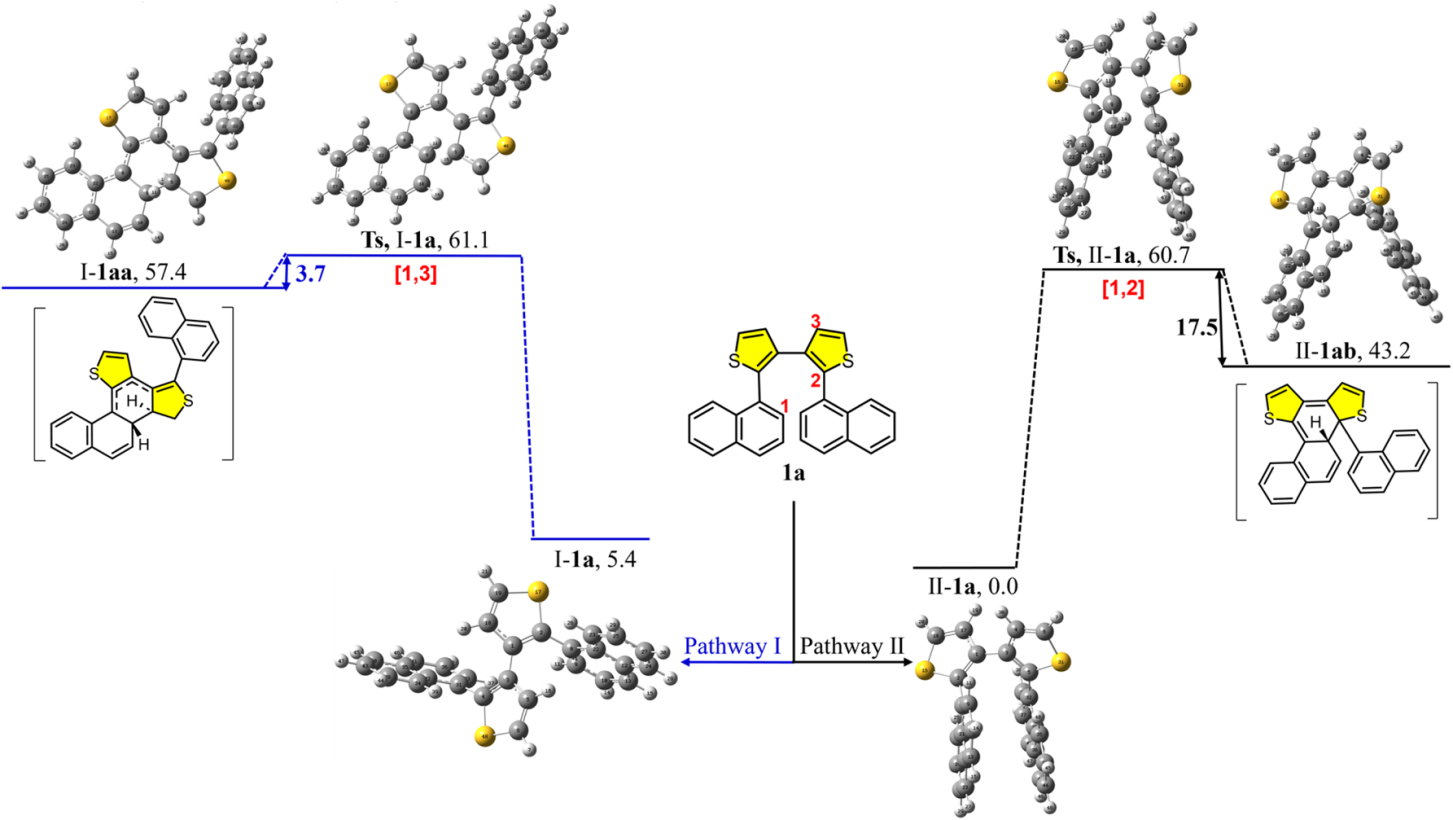
Two computed photocyclization pathways I and II. Precursor 1a and intermediates 1aa and 1ab of the photocyclization reaction were optimized at the ωB97XD/6-31G(d) level of theory. The corresponding transition states were located by broken-symmetry calculations at the UωB97XD/6-31G(d) level with the guess = mix keyword. All relative electronic energies are reported in kcal mol^−1^.

**Fig. 8:**
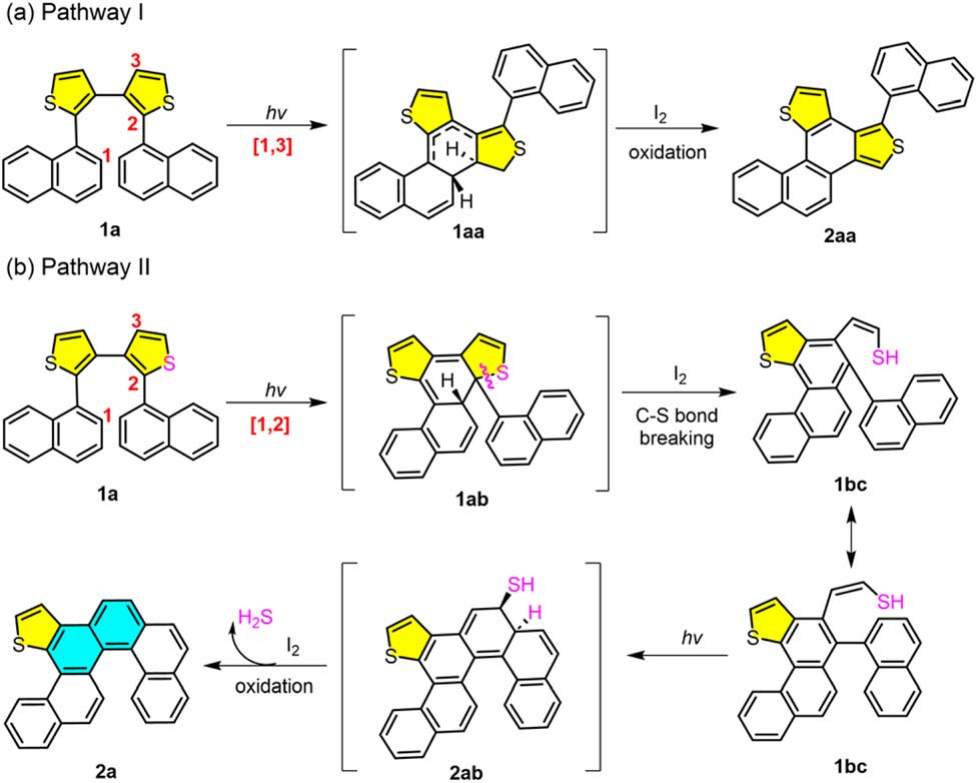
Proposed mechanism for traditional photocyclization pathway I (a) and photochemical cyclization and desulfurization pathway II (b) of diaryl dithiophene 1a.

To further explore the photochemical mechanism, we performed density functional theory (DFT) calculations. Based on our experimental results and previous reports on photocyclization mechanisms,^46–49^ two plausible pathways are proposed (Fig. 7 and 8). Following the traditional photocyclization mechanism, two key cyclization intermediates 1aa (pathway I, cyclization at the 1,3-position) and 1ab (pathway II, cyclization at the 1,2-position) were identified. The ground and transition states (TSs) of precursor 1a were optimized and confirmed to correspond to global minima at the DFT level, accounting for the most energetically favorable conformational transformation pathways.

Two distinct pathways originating from precursor 1a were identified: the traditional photocyclization pathway (I-1a) and a proposed alternative pathway (II-1a). The two ground states of precursor 1a exhibit a relative energy difference of 5.4 kcal mol^−1^. Based on the conformational arrangement of the two intermediate products during photocyclization, two corresponding transition states were located: TS-I-1a and TS-II-1a. The photocyclization process *via* TS-I-1a and TS-II-1a requires activation energies of 61.1 and 60.7 kcal mol^−1^, respectively. Furthermore, the energy barrier for the formation of I-1aa is 3.7 kcal mol^−1^*via* TS-I-1a, whereas the barrier leading to II-1ab*via* TS-II-1a is significantly higher (17.5 kcal mol^−1^). Although the energy difference between the two transition states is small, the energy gaps from the TSs to intermediate products I-1aa and II-1ab differ significantly. The small difference between TS-I-1a and intermediate I-1aa in pathway I suggests that I-1aa is unstable and prone to revert to the transition state. In contrast, the large energy difference from TS-II-1a to intermediate II-1ab in pathway II indicates that product II-1ab is more stable than I-1aa. Given that the two pathways involve transition states with comparable energies, the notable difference in intermediate stability strongly influences the product distribution. These results suggest that pathway II is more favorable and supports the formation of the helicene product. Furthermore, based on pathway II, the thiophene ring-opening step is determined in the subsequent photochemical reaction.

To further elucidate the structural requirements for the photochemical skeletal editing process, we performed DFT calculations on the three possible cyclization pathways for precursor 7b. As shown in Fig. S7, among the three pathways, intermediate product I-7ba derived from pathway I exhibits the greatest thermodynamic stability. Specifically, the energy gap from the transition state to this product is 11.4 kcal mol^−1^, which is significantly more favorable than the corresponding energy barriers for the other two intermediates II-7bb and III-7bc. Notably, the intermediate generated *via* photochemical skeletal editing (pathway III) proved to be the least stable. These computational results are in excellent agreement with the experimental observation that 7b exclusively affords 8b*via* the traditional photocyclization pathway. More importantly, comparison with the DFT results for 1a (Fig. 7) reveals a fundamental mechanistic insight: the presence of two thiophene rings is essential for the photochemical skeletal editing process; when one thiophene is replaced by a benzene ring (as in 7b), the reaction reverts to favoring the traditional photocyclization pathway.

**Fig. 9:**
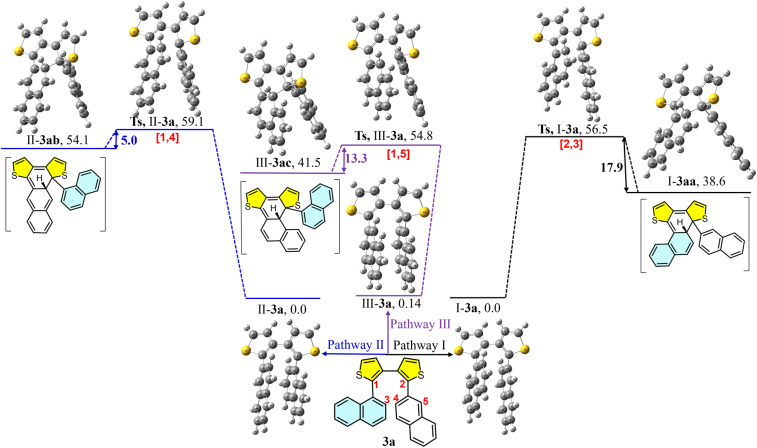
Three computed photocyclization pathways I, II and III. Precursor 3a and intermediates 3aa, 3ab and 3ac of the photocyclization reaction were optimized at the ωB97XD/6-31G(d) level of theory. The corresponding transition states were located by broken-symmetry calculations at the UωB97XD/6-31G(d) level with the guess = mix keyword. All relative electronic energies are reported in kcal mol^−1^.

To investigate the influence of different aryl substituents on reaction selectivity, we selected precursor 3a as a representative example and proposed three plausible cyclization pathways (Fig. 9). Based on the photocyclization mechanism established through DFT calculations (Fig. 7 and 8), three key cyclization intermediates 3aa (pathway I), 3ab (pathway II) and 3ac (pathway III) were identified (see Fig. S7 for details). The ground and transition states (TSs) of precursor 3a were optimized along the most energetically favorable conformational transformation pathways. These three ground states exhibit similar electronic energies. According to the three sets of cyclization positions (2,3 or 1,4 or 1,5), the corresponding transition states during photocyclization were located: TS-I-3a, TS-II-3a and TS-III-3a. The activation energies for photocyclization *via* these transition states are 56.5, 59.1 and 54.8 kcal mol^−1^, respectively. Although the energy differences among the three TSs are small, the energy gaps from the TSs to the corresponding intermediate products differ significantly. Specifically, the energy drop for cyclization at the 2,3-position is larger (17.9 kcal mol^−1^) than that at the 1,4- and 1,5-positions. Moreover, the newly formed C5–C9 bond lengths in the three intermediate products are 1.54 Å, 1.57 Å and 1.56 Å for products I-3aa, II-3ab and III-3ac, respectively. These data indicate that I-3aa is the most stable among the three intermediate products. Given that the three pathways involve transition states with comparable energies, the pronounced difference in intermediate stability plays a decisive role in governing the product distribution. These computational results and proposed mechanism (Fig. S9, pathway I, product 4a) suggest that the formation of thia[4]helicene (I-3aa) is energetically more favorable, consistent with experimental observation of the helicene product. Due to the key reactive site retaining similar electronic characteristics throughout the substrate series (3b–3g), this similarity supports the extrapolation of the 3a-based model to rationalize the general regiospecificity observed experimentally.

### The photophysical and chiroptical properties

The photophysical and chiroptical properties of selected π-extended multiple helicenes 6c–6f were investigated for their high luminescence efficiency. The UV-vis absorption and fluorescence emission spectra of 6c–6f were measured in dichloromethane. Compounds 6e and 6f exhibited similar absorption and emission profiles (Fig. S11), consistent with their analogous structures, as confirmed by X-ray crystallography. The fluorescence quantum yields (*Φ*_f_) were determined to be 6.9% for 6e and 8.2% for 6f. In contrast, 6c showed a distinct red shift in both the longest-wavelength absorption band and fluorescence emission compared to 6e and 6f, along with a higher *Φ*_f_ of 11.4%. This behavior suggests enhanced π-conjugation resulting from a more coplanar molecular geometry. Conversely, 6d exhibited a blue shift in its UV-vis absorption spectrum relative to 6c, likely due to severe structural distortion that reduces effective π-conjugation. Accordingly, the fluorescence quantum yield of 6d was almost negligible (Table S3), which can be attributed to its highly twisted framework. This distorted conformation suppresses the transition dipole moment and diminishes the radiative transition probability, resulting in a low radiative rate constant (*k*_r_ ∼10^−5^ s^−1^) and consequently an extremely low emission efficiency.

**Fig. 10:**
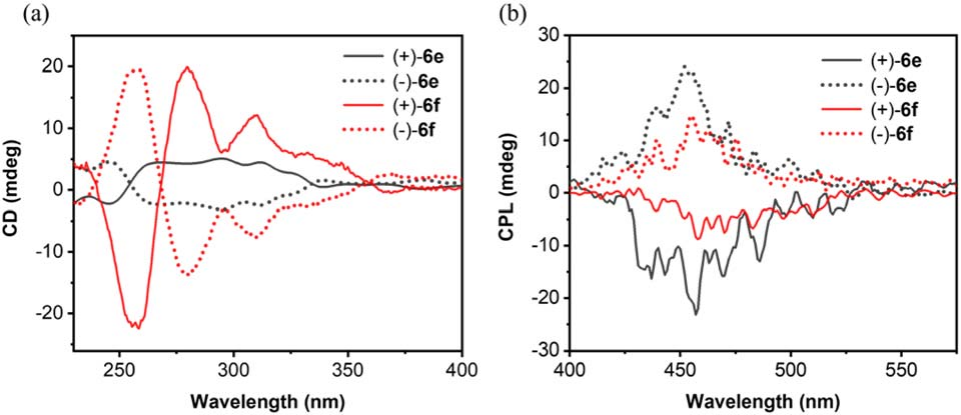
(a) CD and (b) CPL spectra of (+)-6e, (−)-6e, (+)-6f, and (−)-6f in CH_2_Cl_2_ (1 × 10^−5^ M, 298 K).

To evaluate the chiroptical properties, chiral resolution of 6c–6f was attempted. Successful separation of the enantiomers of *rac*-6e and *rac*-6f was achieved *via* chiral HPLC using a Daicel Chiralpak ID column with *n*-hexane/dichloromethane (3 : 1, v/v) as the eluent (Fig. S12). The specific rotations ([*α*]23 D) measured at 0.5 mg mL^−1^ were +3164° and −3058° for the enantiomers of 6e, and +3929° and −3900° for those of 6f. The chiroptical properties of 6e and 6f were studied in dichloromethane using circular dichroism (CD) and circular polarized luminescence (CPL) spectroscopy (Fig. 10). These enantiomers exhibited mirror-image CD spectra in the region 240–400 nm. The absorption dissymmetry factor (|*g*_abs_|) reached maximum values of 2.7 × 10^−4^ at 295 nm for 6e and 2.1 × 10^−4^ at 280 nm for 6f. Furthermore, the enantiomers of both compounds 6e and 6f showed distinct CPL with prominent luminescence dissymmetry factors (*g*_lum_) of 3.5 × 10^−3^ at 457 nm and 2.1 × 10^−3^ at 460 nm, respectively.

## Conclusions

In summary, we successfully developed a novel synthetic method for helicenes based on a photochemical “selective skeletal editing” strategy. Using diaryl dithiophenes as substrates, this strategy achieves the highly selective, direct “exchange” of one thiophene ring for two benzene rings *via* photoinduction, simultaneously driving intramolecular cyclization to efficiently construct various benzofused helicenes with well-defined regiochemistry. This method is scalable and exhibits broad substrate scopes, enabling highly regioselective access to a wide range of helicene architectures. Control experiments reveal distinct reactivity patterns, offering valuable mechanistic insights for the rational design of future helicene-based systems. Mechanistic and computational studies provide strong evidence for the formation of key intermediates, such as 1ab and 3aa (Fig. 7–9), and elucidate the contributions of regioselective photocyclization, thiophene ring-opening and desulfurization steps. Selected helicenes exhibit enriched photophysical properties, including distinctive UV-vis absorption, fluorescence emission, as well as CD and CPL activities, highlighting their potential for chiroptical applications. Importantly, this work moves beyond classical photochemical and skeletal editing by developing direct photochemical aromatic metamorphosis to provide the direct, regiocontrolled conversion of diaryl heterocycles into complex three-dimensional helical frameworks. This study not only demonstrates the immense potential of skeletal editing in constructing high-order complex molecules but also points the way for the future design of other types of “*S*-heterocyclic editing” reactions to directly synthesize various challenging functional polycyclic aromatic hydrocarbons, offering a step-economical route to chiral functional materials from simple precursors.

## Author contributions

X. S. and L. M. contributed to explore the substrate scope. C. D. and C. C. helped with the collection of some new compounds and data analysis. C. Z. developed the reaction methods and performed the experiments. Y. X. analyzed the photophysical data. W. X. and C. L. supervised the experimental work and analyzed the data. Z. M. conducted the mechanistic computational studies. G. W. directed the investigations and wrote the manuscript. H. W. conceived the concept and supervised the project. All authors discussed the results and commented on the manuscript.

## Conflicts of interest

There are no conflicts to declare.

## Supplementary Material

SC-017-D6SC01717G-s001

SC-017-D6SC01717G-s002

## Data Availability

CCDC 2479166 (2a), 2479167 (2b), 2479168 (2d), 2479169 (4g), 2479170 (6e), 2479172 (6f) and 2479276 (8b) contain the supplementary crystallographic data for this paper.^50*a*–*g*^ All data supporting the findings of this study are included in the main manuscript and the supplementary information (SI). Supplementary information: preparation methods, crystal data, calculation details, photophysical properties, HPLC analysis and NMR spectra. See DOI: https://doi.org/10.1039/d6sc01717g.

## References

[cit1] Kim S. F., Amber C., Bartholomew G. L., Sarpong R. (2025). Skeletal Editing Strategies Driven by Total Synthesis. Acc. Chem. Res..

[cit2] Wu F.-P., Tyler J. L., Glorius F. (2025). Diversity-Generating Skeletal Editing Transformations. Acc. Chem. Res..

[cit3] Ding L. L., Fan Y., Lu H. J. (2025). Skeletal Editing Based on Nitrogen-atom Manipulation. Chem. Soc. Rev..

[cit4] Jurczyk J., Woo J., Kim S. F., Dherange B. D., Sarpong R., Levin M. D. (2022). Single-atom Logic for Heterocycle Editing. Nat. Synth..

[cit5] Kim D., You J., Lee D. H., Hong H., Kim D., Park Y. (2024). Photocatalytic Furan-to-Pyrrole Conversion. Science.

[cit6] Li J. H., Tang P. C., Fan Y., Lu H. J. (2025). Skeletal Editing of Pyrrolidines by Nitrogen-Atom Insertion. Science.

[cit7] Bartholomew G. L., Carpaneto F., Sarpong R. (2022). Skeletal Editing of Pyrimidines to Pyrazoles by Formal Carbon Deletion. J. Am. Chem. Soc..

[cit8] Tian D., He Y. P., Yang L. S., Li Z. C., Wu H. (2025). Switchable Skeletal Editing of Quinolines Enabled by Cyclizative Sequential Rearrangements. Nat. Chem..

[cit9] Shi C. Q., Wang E. H., Zhang Y. X., Ma T. Y., Zhang R. C., Huang H., He Y., Peng Q., Feng Z. (2026). Iron-Catalyzed Divergent Skeletal Editing of Benzofurans. CCS Chem..

[cit10] Shah R., Verma P. K. (2018). Therapeutic Importance of Synthetic Thiophene. Chem. Cent. J..

[cit11] llardi E. A., Vitaku E., Njardarson J. T. (2014). Data-Mining for Sulfur and Fluorine: An Evaluation of Pharmaceuticals to Reveal Opportunities for Drug Design and Discovery. J. Med. Chem..

[cit12] Shi J. W., Li Y., Jia M., Xu L., Wang H. (2011). Organic Semiconductors Based on Annelated β-oligothiophenes and Its Application for Organic Field-effect Transistors. J. Mater. Chem..

[cit13] Zhang C., Liu Y., Ma Z. Y., Wang G. X., Li C. L., Yang F. X., Shi J. W., Li R. J., Wang H. (2022). Dragon-Boat-Type Heptathienoacenes: Synthesis, Structures, and Their Applications in OFETs. Org. Lett..

[cit14] Wu R. S., Meng X. L., Yang Q., Zhang W. J., Shen S. S., Yang L. S., Li M., Chen Y., Zhou Y. Y., Song J. S. (2024). Synergistically Regulating the Conjugation Length and Side Chain on Oligothiophene-Based Fully Nonfused Ring Electron Acceptors for Efficient Organic Solar Cells. ACS Appl. Polym. Mater..

[cit15] Yorimitsu H. (2025). Aromatic Metamorphosis: Skeletal Editing of Aromatic Rings. Acc. Chem. Res..

[cit16] Nogi K., Yorimitsu H. (2017). Aromatic Metamorphosis: Conversion of An Aromatic Skeleton into a Different Ring System. Chem. Commun..

[cit17] Vasu D., Yorimitsu H., Osuka A. (2015). Palladium-Assisted “Aromatic Metamorphosis” of Dibenzothiophenes into Triphenylenes. Angew. Chem., Int. Ed..

[cit18] Bhanuchandra M., Murakami K., Vasu D., Yorimitsu H., Osuka A. (2015). Transition-Metal-Free Synthesis of Carbazoles and Indoles by an SNArBased “Aromatic Metamorphosis” of Thiaarenes. Angew. Chem., Int. Ed..

[cit19] Kaga A., Iida H., Tsuchiya S., Saito H., Nakano K., Yorimitsu H. (2021). Aromatic Metamorphosis of Thiophenes by Means of Desulfurative Dilithiation. Chem.–Eur. J..

[cit20] Song C. L., Dong X., Wang Z. J., Liu K., Chiang C.-W., Lei A. (2019). Visible-Light-Induced [4+2] Annulation of Thiophenes and Alkynes to Construct Benzene Rings. Angew. Chem., Int. Ed..

[cit21] Wang H. M., Shao H. L., Das A., Dutta S., Chan H. T., Daniliuc C., Houk K. N., Glorious F. (2023). Dearomative Ring Expansion of Thiophenes by Bicyclobutane Insertion. Science.

[cit22] Shen Y., Chen C.-F. (2012). Helicenes: Synthesis and Applications. Chem. Rev..

[cit23] Mori T. (2021). Chiroptical Properties of Symmetric Double, Triple, and Multiple Helicenes. Chem. Rev..

[cit24] Tsurusaki A., Kamikawa K. (2021). Multiple Helicenes Featuring Synthetic Approaches and Molecular Structures. Chem. Lett..

[cit25] Nakazaki M., Yamamoto K., Ikeda T., Kitsuki T., Okamoto Y. (1983). Synthesis and Chiral Recognition of Novel Crown Ethers Incorporating Helicene Chiral Centres. J. Chem. Soc. Chem. Commun..

[cit26] Malik A. U., Gan F. W., Shen C. H., Yu N., Wang R. B., Crassous J., Shu M. H., Qiu H. B. (2018). Chiral Organic Cages with a Triple-Stranded Helical Structure Derived from Helicene. J. Am. Chem. Soc..

[cit27] Matsumoto A., Yonemitsu K., Ozaki H., Míšek J., Starý I., Stará I. G., Soai K. (2017). Reversal of the Sense of Enantioselectivity Between 1- and 2-aza[6]helicenes Used as Chiral Inducers of Asymmetric Autocatalysis. Org. Biomol. Chem..

[cit28] Sakamoto D., Sánchez I. G., Rybáček J., Vacek J., Bednárová L., Pazderková M., Pohl R., Císařová I., Stará I. G., Starý I. (2022). Cycloiridated Helicenes as Chiral Catalysts in the Asymmetric Transfer Hydrogenation of Imines. ACS Catal..

[cit29] Zhao W. L., Li M., Lu H. Y., Chen C. F. (2019). Advances in Helicene Derivatives with Circularly Polarized Luminescence. Chem. Commun..

[cit30] Tian X. Q., Shoyama K., Mahlmeister B., Brust F., Stolte M., Würthner F. (2023). Naphthalimide-Annulated [n] Helicenes: Red Circularly Polarized Light Emitters. J. Am. Chem. Soc..

[cit31] Dang L. P., Xu W., Qiu S., Yu Y. J., Ma Z. Y., Yue L., Su H., Li C. L., Wang H. (2024). Construction and Circularly Polarized Luminescence of Thiophene Based Multiple Helicenes. Org. Lett..

[cit32] Martin R. H., Baes M. (1975). Helicenes: Photosyntheses of [11], [12] and [14]helicene. Tetrahedron.

[cit33] Hoffmann N. (2014). Photochemical Reactions Applied to the Synthesis of Helicenes and Helicene-like Compounds. J. Photochem. Photobiol. C Photochem. Rev..

[cit34] Mori K., Murase T., Fujita M. (2015). One-step Synthesis of [16]helicene. Angew. Chem., Int. Ed..

[cit35] Liu X. M., Yu P. P., Xu L., Yang J. J., Shi J. W., Wang Z. H., Cheng Y. X., Wang H. (2013). Synthesis for the Mesomer and Racemate of Thiophene-Based Double Helicene under Irradiation. J. Org. Chem..

[cit36] Sun Z., Xu W., Qiu S., Ma Z. Y., Li C. L., Zhang S., Wang H. (2024). Thia[*n*]helicenes with Long Persistent Phosphorescence. Chem. Sci..

[cit37] Laarhoven W. H. (1983). Photochemical Cyclizations and Intramolecular Cycloadditions of Conjugated Arylolefins. Part I: Photocyclization with Dehydrogenation. Recl. Trav. Chim. Pays-Bas.

[cit38] Sato K., Yamagishi T., Arai S. (2000). Synthesis of Novel Azonia[5]helicenes Containing Terminal Thiophene Rings. J. Heterocycl. Chem..

[cit39] Fujikawa T., Preda D. V., Segawa Y., Itami K., Scott L. T. (2016). Corannulene-Helicene Hybrids: Chiral π-Systems Comprising Both Bowl and Helical Motifs. Org. Lett..

[cit40] Nenajdenko V. G., Gribkov D. V., Sumerin V. V., Balenkova E. S. (2003). A Novel Synthesis of dithieno[2,3-b:3′,2′-d]thiophene and its Bromo Derivatives. Synthesis.

[cit41] Becker H. D. (1993). Unimolecular Photochemistry of Anthracenes. Chem. Rev..

[cit42] Bouas-Laurent H., Castellan A., Desvergne J. P., Lapouyade R. (2001). Photodimerization of Anthracenes in Fluid Solutions: (part 2) Mechanistic Aspects of the Photocycloaddition and of the Photochemical and Thermal Cleavage. Chem. Soc. Rev..

[cit43] Wang X., Liu W. G., Tung C. H., Wu L. Z., Cong H. (2019). A Monophosphine Ligand Derived From Anthracene Photodimer: Synthetic Applications for Palladium-catalyzed Coupling Reactions. Org. Lett..

[cit44] Shao F., Wang W., Yang W. M., Yang Z. L., Zhang Y., Lan J. G., Schlüter A. D., Zenobi R. (2021). In-situ Nanospectroscopic Imaging of Plasmoninduced two-dimensional [4+4]-cycloaddition Polymerization on Au(111). Nat. Commun..

[cit45] Sankar E., Raju P., Karunakaran J., Mohanakrishnan A. K. (2017). Synthetic Utility of Arylmethylsulfones: Annulative π-extension of Aromatics and Hetero-aromatics Involving Pd(0)-catalyzed Heck Coupling Reactions. J. Org. Chem..

[cit46] Mallory F. B. and Mallory C. W., Photocyclization of Stilbenes and Related Molecules, in: Organic Reactions, John Wiley & Sons, New York, 1984, vol. 30, pp. 1–456

[cit47] Molloy M. S., Snyder J. A., Bragg A. E. (2014). Structural and Solvent Control of Nonadiabatic Photochemical Bond Formation: Photocyclization of o-Terphenyl in Solution. J. Phys. Chem. A.

[cit48] Weber J., Clennan E. L. (2019). Origin of the Preferential Formation of Helicenes in Mallory Photocyclizations. Temperature as a Tool to Influence Reaction Regiochemistry. J. Org. Chem..

[cit49] Hulley E. B., Clennan E. L. (2024). Dihydrophenanthrene Open-Shell Singlet Diradicals and Their Roles in the Mallory Photocyclization Reaction. J. Am. Chem. Soc..

[cit50] (a) CCDC 2479166: Experimental Crystal Structure Determination, 2026, 10.5517/ccdc.csd.cc2p6s3t

