# Supplementary material for: Photochemical skeletal editing: one-step transformation of diaryl dithiophenes into regiodefined helicenes
Source: Chem Sci. 2026 Mar 25;17(20):10082–91. doi: 10.1039/d6sc01717g (PMC13055911; doi:10.1039/d6sc01717g)
Supplement: SC-017-D6SC01717G-s001 [file SC-017-D6SC01717G-s001.pdf]

Supplementary Information (SI) for Chemical Science.
This journal is © The Royal Society of Chemistry 2026

Supporting Information

Photochemical Skeletal Editing: One-Step Transformation of Diaryl Dithiophenes into Regiodefined Helicenes

Xiaoli Shi§, Ling Mei§, Chenxi Dong, Chunmei Zhao, Chen Chen, Yimin Xu, Wan Xu, Chunli Li, Guangxia Wang*, Zhiying Ma* and Hua Wang*

Institute of Nanoscience and Engineering, Henan University, Kaifeng, 475004, China

E-mail address: wangguangxia@henu.edu.cn; mazy11@henu.edu.cn; hwang@henu.edu.cn

Table of Contents

1. General Procedures and Materials 2
2. Syntheses of New Compounds 3
3. Crystal Structures and Crystal Data 32
4. ^{1}H NMR Studies for **2a**, **8b**, **8c**, **8d**, **8e** and **2a'** 34
5. ^{1}H-^{1}H NOESY Spectra for **4g**, **2a** and **2a'** 35
6. DFT calculation and proposed mechanism 37
7. Photophysical Studies 38
8. HPLC Analysis of **6e** and **6f** 39
9. NMR Spectra and HRMS Spectra of New Compounds 40
10. Reference 130

1. General Procedures and Materials

All starting chemicals were obtained from commercial sources and used without further purification. Anhydrous tetrahydrofuran (THF) was distilled sodium benzophenone under argon. Concentration of *n*-BuLi (in hexane) was determined by titration with N-pivaloyl-*o*-toluidine.[S1] Compound 2,2'-dibromo-3,3'-bithiophene[S2], **10**[S3], **11**[S4], **14**[S5] and **17**[S6] was prepared as previously reported. Column chromatography was carried out on silica gel using 200-300 and 300-400 mesh. Analytical thin-layer chromatography (TLC) was performed using precoated TLC plates with silica gel GF-254.

NMR spectra were obtained with a Bruker spectrometer (^{1}H, ^{13}C, Bruker AVANCE 400 MHz, Bruker AVANCE 500MHz) using chloroform-*d* ($CDCl_3$), dichloromethane-d_2 (CD_2Cl_2), 1,1,2,2-tetrachloroethane-d_2 (CD_2Cl_4) and dimethyl sulfoxide-d_6 (DMSO-d_6) as solvent. The chemical shift references were as follows: (^{1}H) $CDCl_3$, 7.26 ppm; (^{1}H) CD_2Cl_2, 5.32 ppm; (^{1}H) CD_2Cl_4, 6.00 ppm; (^{1}H) DMSO-d_6, 2.50 ppm; (^{13}C) chloroform-*d*, 77.16 ppm; (^{13}C) dichloromethane-d_2, 54.00 ppm. IR spectra were recorded on FT-IR spectrometer with thin KBr disk. High resolution mass spectra (DART, EI, MALDI) were acquired on FT-ICR, TOF spectrometer. Melting point determination was taken on a Melt-Temp apparatus and was uncorrected. The specific rotations ([α]23 D) were recorded on a Digipol P910 polarimeter. Circular dichroism (CD) spectra were obtained using Chirascan TM spectropolarimeter (Aviv Biomedical Inc., United Kingdom), equipped with a Peltier temperature controller. Circularly polarized luminescence (CPL) properties were measured with a CPL-300 spectrophotometer (JASCO).

2. Syntheses of New Compounds

Scheme S1. Synthesis of compounds 2a–2d.

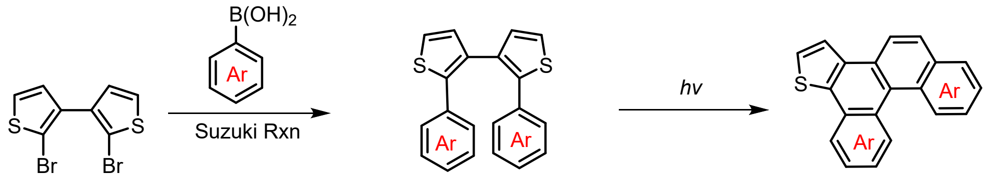

1a–1d **2a–2d**

Synthesis of 1a

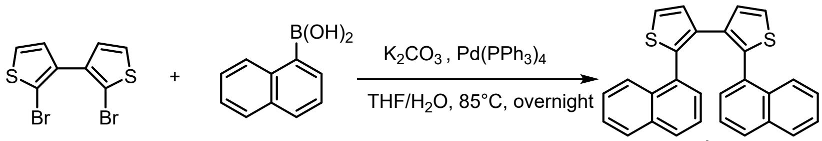

1a

A mixture of 2,2'-dibromo-3,3'-bithiophene (1.0 g, 3.08 mmol), 1-naphthaleneboronic acid (4.4 g, 2.57 mmol), K_2CO_3 (816.9 mg, 5.91 mmol), and $Pd(PPh_3)_4$ (59.4 mg, 0.05 mmol) and deoxidized water (3 mL) were added into THF (30 mL) and stirred under Ar atmosphere for 36 h at 85 °C. The solution was concentrated under reduce pressure. The crude product was extracted with dichloromethane and water, dried over anhydrous $MgSO_4$. After the solvent was removed under vacuum, the residue was purified by silica gel column chromatography with petrol ether (60-90 °C) as the eluent to afford **1a** as a white solid (470 mg, 49%). M.P.: 197 - 199 °C. ^{1}H NMR (500 MHz, CD_2Cl_2) δ 7.77 (d, J = 8.2 Hz, 2H), 7.66 (d, J = 8.2 Hz, 2H), 7.49 (d, J = 8.5 Hz, 2H), 7.41 (ddd, J_1 = 8.1 Hz, J_2 = 6.8 Hz, J_3 = 1.1 Hz, 2H), 7.27 (ddd, J_1 = 8.2 Hz, J_2 = 6.8 Hz, J_3 = 1.2 Hz, 2H), 7.22 (d, J = 5.3 Hz, 2H), 7.19 (dd, J_1 = 8.1 Hz, J_2 = 7.2 Hz, 2H), 6.97 (dd, J_1 = 11.5 Hz, J_2 = 3.0 Hz, 4H). ^{13}C NMR (100 MHz, CD_2Cl_2) δ 137.77, 135.37, 134.14, 132.44, 132.28, 129.94, 129.36, 128.81, 128.52, 126.38, 126.28, 126.20, 125.59, 125.17. HRMS (DART-FTICR): m/z $[M+H]^+$ calcd for $[C_{28}H_{19}S_2]$ 419.0923, found 419.0921. IR (KBr): 3042, 1589, 1502, 1382, 892, 847, 789, 732 cm^{-1}.

Synthesis of 1b

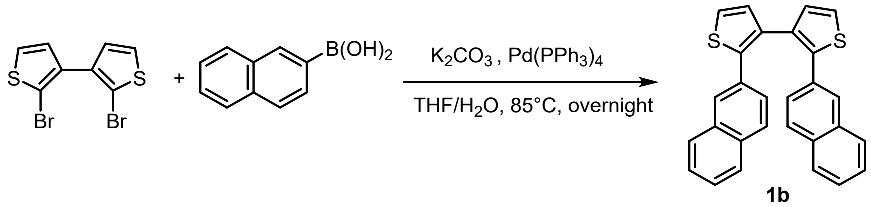

1b

A mixture of 2,2'-dibromo-3,3'-bithiophene (97.8 mg, 0.30 mmol), 2-naphthaleneboronic acid (108.4 mg, 0.63 mmol), K_2CO_3 (167.2 mg, 1.21 mmol), and $Pd(PPh_3)_4$ (20.8 mg, 0.02 mmol) and

deoxidized water (3 mL) were added into THF (30 mL) and stirred under Ar atmosphere for 12 h at 85 °C. The solution was concentrated under reduce pressure. The crude product was extracted with dichloromethane and water, dried over anhydrous $MgSO_4$. After the solvent was removed under vacuum, the residue was purified by silica gel column chromatography with petrol ether (60-90 °C) as the eluent to afford **1b** as a white solid (113.0 mg, 87%). M.P.: 187-188 °C. ^1H NMR (400 MHz, CD_2Cl_2) δ 7.73 (d, J = 8.0 Hz, 2H), 7.56 (d, J = 8.5 Hz, 2H), 7.50-7.47 (m, 4H), 7.42 (t, J = 7.2 Hz, 2H), 7.36 (t, J = 7.5 Hz, 2H), 7.31 (d, J = 5.2 Hz, 2H), 7.23 (dd, J_1 = 8.5, J_2 =1.7 Hz, 2H), 6.98 (d, J = 5.2 Hz, 2H). ^{13}C NMR (100 MHz, CD_2Cl_2) δ 140.7, 133.8, 133.7, 132.9, 132.4, 131.3, 128.3, 128.1, 127.9, 127.9, 127.1, 126.6, 126.5, 124.9. HRMS (MALDI): m/z $[M]^+$ calcd for $[C_{28}H_{18}S_2]$ 418.0844, found 418.0840. IR (KBr): 3049, 1596, 1499, 819, 720 cm^{-1}.

Synthesis of 1c

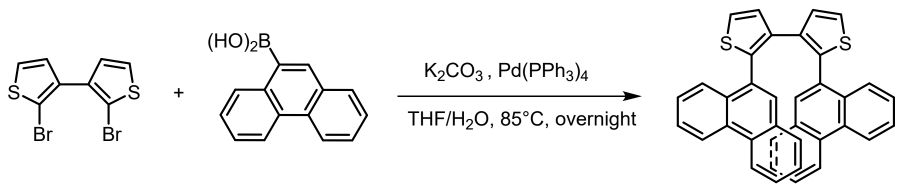

1c

A mixture of 2,2'-dibromo-3,3'-bithiophene (103.5 mg, 0.32 mmol), 9-phenanthreneboronic acid (135.5 mg, 0.61 mmol), K_2CO_3 (176.9 mg, 1.28 mmol), and $Pd(PPh_3)_4$ (34.7 mg, 0.03 mmol) and deoxidized water (1.3 mL) were added into THF (10 mL) and stirred under Ar atmosphere for 12 h at 85 °C. The solution was concentrated under reduce pressure. The crude product was extracted with dichloromethane and water, dried over anhydrous $MgSO_4$. After the solvent was removed under vacuum, the residue was purified by silica gel column chromatography with petrol ether (60-90 °C) as the eluent to afford **1c** as a yellow solid (113.0 mg, 71%). M.P.: 276-278 °C. ^1H NMR (400 MHz, CD_2Cl_2) δ 8.47 (d, J = 8.3 Hz, 4H), 7.56 (t, J = 8.0 Hz, 2H), 7.45 (dd, J = 15.7, 7.6 Hz, 6H), 7.34 (d, J = 7.8 Hz, 2H), 7.31 (d, J = 5.3 Hz, 2H), 7.23 - 7.17 (m, 4H), 7.01 (s, 2H). ^{13}C NMR (100 MHz, CD_2Cl_2) δ 137.87, 135.66, 131.56, 130.99, 130.95, 130.78, 130.51, 130.20, 130.13, 128.98, 127.26, 127.05, 126.80, 126.72, 126.51, 125.43, 122.87, 122.81. HRMS (MALDI-TOF): m/z $[M]^+$ calcd for $[C_{36}H_{22}S_2]$ 518.11462, found 518.11574. IR(KBr): 3062, 1598, 1374, 1334, 848, 753, 737, 716 cm^{-1}.

Synthesis of 1d

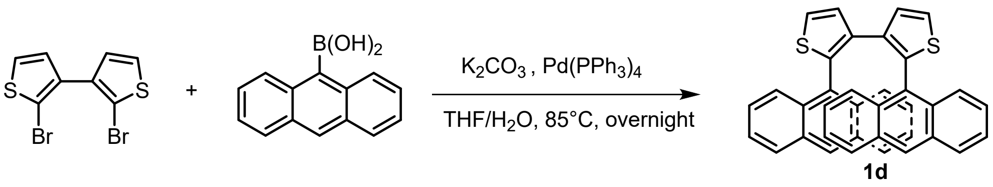

A mixture of 2,2'-dibromo-3,3'-bithiophene (99.2 mg, 0.31 mmol), 9-anthraceneboronic acid (128.8 mg, 0.58 mmol), K_2CO_3 (168.6 mg, 1.22 mmol), and $Pd(PPh_3)_4$ (34.7 mg, 0.03 mmol) and deoxidized water (1.3 mL) were added into THF (10 mL) and stirred under Ar atmosphere for 12 h at 85 °C. The solution was concentrated under reduce pressure. The crude product was extracted with dichloromethane and water, dried over anhydrous $MgSO_4$. After the solvent was removed under vacuum, the residue was purified by silica gel column chromatography with petrol ether (60-90 °C) as the eluent to afford **1d** as a yellow solid (82.0 mg, 54%). M.P.: > 300 °C. ^{1}H NMR (400 MHz, $CDCl_3$) δ 8.35 (s, 2H), 7.96 (d, J = 8.4 Hz, 4H), 7.58 (d, J = 8.8 Hz, 4H), 7.45 (t, J = 7.4 Hz, 4H), 7.33 (t, J = 7.4 Hz, 4H), 7.05 (d, J = 5.4 Hz, 2H), 6.65 (d, J = 5.3 Hz, 2H). ^{13}C NMR (100 MHz, $CDCl_3$) δ 136.00, 134.81, 131.90, 131.30, 128.79, 128.58, 128.29, 128.18, 126.72, 126.05, 125.65, 125.26. HRMS (DART): m/z $[M+H]^+$ calcd for $[C_{36}H_{23}S_2]$ 519.1236, found 519.1233. IR (KBr): 3045, 1443, 1326, 852, 728 cm^{-1}.

Synthesis of 2a

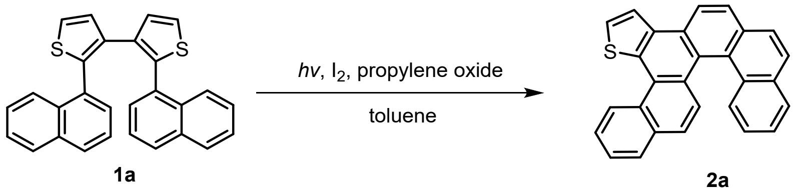

A mixture of **1a** (80.0 mg, 0.19 mmol), I_2 (145.5 mg, 0.57 mmol, propylene oxide (0.4 mL) was added into anhydrous toluene (120 mL) under argon. Then equally distributed into 8 quartz tubes. The reaction mixture was irradiation with a high-pressure Hg lamp for 0.4 h. After being quenched with $Na_2S_2O_3$, the reaction mixture was extracted with CH_2Cl_2 and H_2O, and then dried over $MgSO_4$. After the solvent was removed under vacuum, the residue was purified by silica gel column chromatography (eluent: PE/DCM = 5/1) and subsequently precipitated from a mixture of CH_2Cl_2/ CH_3OH to afford **2a** as a grayish-white solid (18.6 mg, 25%). M.P.: 265-268 °C. ^{1}H NMR (400 MHz, $CDCl_3$) δ 9.31 (d, J = 8.4 Hz, 1H), 8.50 (d, J = 8.4 Hz, 1H), 8.28 (d, J = 5.6 Hz, 1H), 8.23 (dd, J_1 = 8.7 Hz, J_2 = 4.8 Hz, 2H), 8.06 (d, J = 8.4 Hz, 1H), 8.00-7.95 (m, 4H), 7.88 (t, J = 7.7 Hz, 1H), 7.84 (d, J = 5.5 Hz, 1H), 7.71 (t, J = 7.4 Hz, 1H), 7.54-7.49 (m, 2H), 7.22 (t, J = 7.7 Hz, 1H). ^{13}C NMR

(100 MHz, $CDCl_3$) δ 136.33, 134.41, 132.95, 132.77, 131.82, 131.15, 129.93, 129.75, 129.55, 129.00, 128.62, 128.56, 128.08, 127.97, 127.61, 127.44, 126.90, 126.56, 126.49, 126.43, 126.34, 125.92, 125.70, 125.01, 123.89, 122.78, 122.62. HRMS (MALDI): m/z $[M]^+$ calcd for $[C_{28}H_{16}S]$ 384.09544, found 384.09672. IR (KBr): 3042, 1504, 1459, 828, 742 cm^{-1}.

Synthesis of 2b

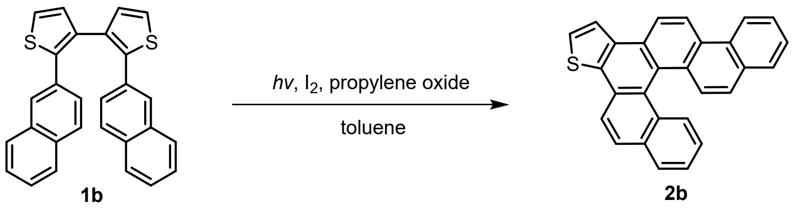

A mixture of **1b** (83.0 mg, 0.20 mmol), I_2 (149.7 mg, 0.59 mmol, propylene oxide (0.4 mL) was added into anhydrous toluene (120 mL) under argon. Then equally distributed into 8 quartz tubes. The reaction mixture was irradiation with a high-pressure Hg lamp for 0.4 h. After being quenched with $Na_2S_2O_3$, the reaction mixture was extracted with CH_2Cl_2 and H_2O, and then dried over $MgSO_4$. After the solvent was removed under vacuum, the residue was purified by silica gel column chromatography (eluent: PE/DCM = 5/1) and subsequently precipitated from a mixture of CH_2Cl_2/ CH_3OH to afford **2b** as a grayish-white solid (66.6 mg, 87%). M.P.: 286-287 °C. ^{1}H NMR (400 MHz, CD_2Cl_2) δ 8.99 (d, J = 8.8 Hz, 1H), 8.88 (d, J = 8.3 Hz, 1H), 8.63 (d, J = 8.8 Hz, 1H), 8.22 (d, J = 8.6 Hz, 1H), 8.18 (d, J = 8.6 Hz, 1H), 8.14 (d, J = 5.4 Hz, 1H), 8.09 (dd, J_1 = 8.9 Hz, J_2 = 4.6 Hz, 2H), 8.02 (d, J = 7.9 Hz, 1H), 7.93 (d, J = 7.6 Hz, 1H), 7.77 - 7.73 (m, 2H), 7.66 (t, J = 7.4 Hz, 1H), 7.56-7.50 (m, 2H), 7.27 (t, J = 7.7 Hz, 1H). ^{13}C NMR (100 MHz, CD_2Cl_2) δ 137.96, 136.15, 132.76, 132.37, 132.10, 131.01, 130.45, 129.91, 129.21, 129.01, 128.89, 128.82, 128.73, 128.60, 128.57, 127.25, 127.18, 126.90, 126.40, 126.08, 125.81, 125.24, 124.89, 123.86, 123.64, 123.17, 122.93, 122.92. HRMS (MALDI): m/z $[M]^+$ calcd for $[C_{28}H_{16}S]$ 384.0967, found 384.0962. IR (KBr): 3046, 1602, 1254, 1088, 1032, 8144, 748 cm^{-1}.

Synthesis of 2c

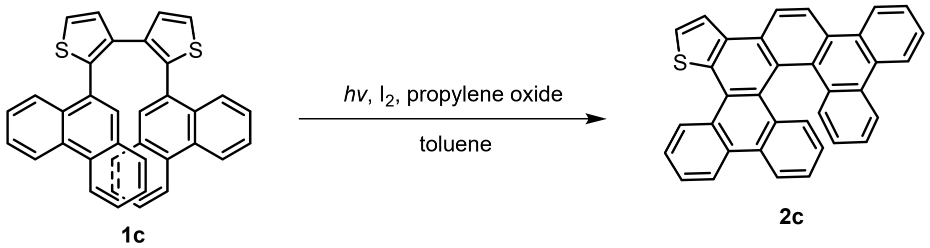

A mixture of **1c** (72.1 mg, 0.14 mmol), I_2 (176.4 mg, 0.70 mmol, propylene oxide (0.7 mL) was added into anhydrous toluene (105 mL) under argon. Then equally distributed into 7 quartz tubes. The reaction mixture was irradiation with a high-pressure Hg lamp for 17 h. After being quenched with $Na_2S_2O_3$, the reaction mixture was extracted with CH_2Cl_2 and H_2O, and then dried over $MgSO_4$. After the solvent was removed under vacuum, the residue was purified by silica gel column chromatography (eluent: PE/DCM = 5/1) and subsequently precipitated from a mixture of CH_2Cl_2/ CH_3OH to afford **2c** as a grayish-white solid (12.0 mg, 18%). M.P.: > 300 °C. ^1H NMR (400 MHz, $CDCl_3$) δ 9.30 (d, J = 7.9 Hz, 1H), 8.88 (d, J = 8.8 Hz, 1H), 8.80 (t, J = 7.3 Hz, 2H), 8.70 (d, J = 7.7 Hz, 1H), 8.67 (d, J = 8.7 Hz, 1H), 8.45 (dd, J_1 = 7.9, J_2 = 5.3 Hz, 2H), 8.28 (d, J = 5.5 Hz, 1H), 7.91 (t, J = 7.6 Hz, 1H), 7.86-7.74 (m, 4H), 7.55 (d, J = 8.4 Hz, 1H), 7.43 (d, J = 8.3 Hz, 1H), 7.25-7.21 (m, 2H), 6.69 (dd, J_1= 12.2, J_2 = 7.5 Hz, 2H). ^{13}C NMR (100 MHz, $CDCl_3$) δ 136.60, 134.48, 131.07, 130.95, 130.57, 130.33, 130.01, 129.86, 129.76, 129.54, 129.42, 129.16, 129.13, 128.38, 128.37, 128.03, 127.63, 127.55, 127.37, 127.31, 126.84, 126.79, 126.54, 126.47, 126.22, 125.98, 125.59, 123.99, 123.76, 123.52, 123.00, 122.97, 122.83, 122.82, 122.52, 122.26. HRMS (MALDI): m/z $[M]^+$ calcd for $[C_{36}H_{20}S]$ 484.12774, found 484.12802. IR (KBr): 3070, 2922, 1453, 742 cm^{-1}.

Synthesis of 2d

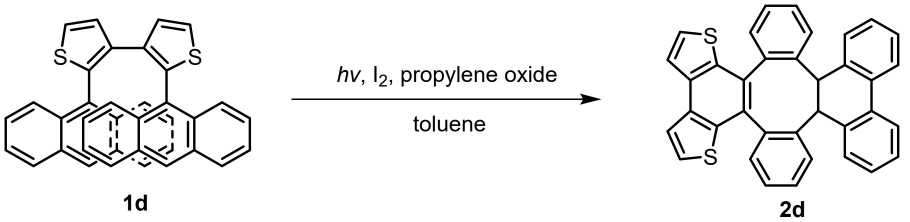

1d **2d**

A mixture of **1d** (80.0 mg, 0.15 mmol), I_2 (116.8 mg, 0.46 mmol), propylene oxide (0.4 mL) was added into anhydrous toluene (105 mL) under argon. Then equally distributed into 7 quartz tubes. The reaction mixture was irradiation with a high-pressure Hg lamp for 3.5 h. After being quenched with $Na_2S_2O_3$, the reaction mixture was extracted with CH_2Cl_2 and H_2O, and then dried over $MgSO_4$. After the solvent was removed under vacuum, the residue was purified by silica gel column chromatography (eluent: PE/DCM = 5/1) and subsequently precipitated from a mixture of CH_2Cl_2/ CH_3OH to afford **2d** as a grayish-white solid (61.0 mg, 77%). M.P.: > 300 °C. ^1H NMR (400 MHz, $CDCl_3$) δ 7.36 (d, J = 5.3 Hz, 2H), 7.31 (d, J = 5.3 Hz, 2H), 6.85 (dd, J_1 = 7.3 Hz, J_2 =1.3 Hz, 4H), 6.79 (td, J_1 = 7.3 Hz, J_2 = 1.3 Hz, 4H), 6.73 (td, J_1 = 7.5 Hz, J_2 = 1.5 Hz, 4H), 6.66 (dd, J_1 = 7.7 Hz, J_2 = 1.1 Hz, 4H), 4.55 (s, 2H). ^{13}C NMR (100 MHz, $CDCl_3$) δ 148.09, 142.85, 140.79, 134.81, 130.75,

126.97, 126.62, 126.11, 125.24, 121.28, 63.24. HRMS (DART): m/z $[M+H]^+$ calcd for $[C_{36}H_{23}S_2]$ 519.1236, found 519.1235. IR (KBr): 3065, 3019, 2932, 1460, 697 cm^{-1}.

Scheme S2. Synthesis of compounds 4a–4g.

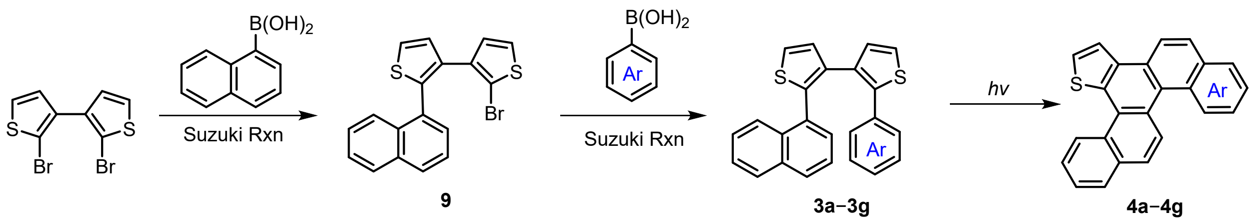

Synthesis of 9

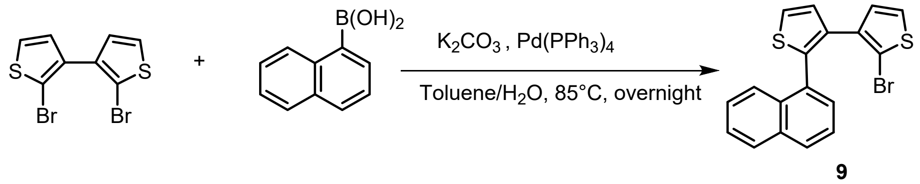

A mixture of 2,2'-dibromo-3,3'-bithiophene (1.0 g, 3.08 mmol), 1-naphthaleneboronic acid (442.3 mg, 2.57 mmol), K_2CO_3 (816.9 mg, 5.91 mmol), and $Pd(PPh_3)_4$ (59.4 mg, 0.05 mmol) and deoxidized water (3 mL) were added into toluene (50 mL) and stirred under Ar atmosphere for 36 h at 85 °C. The solution was concentrated under reduce pressure. The crude product was extracted with dichloromethane and water, dried over anhydrous $MgSO_4$. After the solvent was removed under vacuum, the residue was purified by silica gel column chromatography with petrol ether (60-90 °C) as the eluent to afford **9** as a yellow solid (470.0 mg, 49%). M.P.: 164-166 °C. ^{1}H NMR (400 MHz, $CDCl_3$) δ 7.83 (d, J = 3.8 Hz, 1H), 7.82 (d, J = 3.8 Hz, 1H), 7.79 (d, J = 8.5 Hz, 1H), 7.49-7.46 (m, 2H), 7.46-7.40 (m, 3H), 7.36 (ddd, J_1 = 8.2 Hz, J_2 = 6.9 Hz, J_3 = 1.3 Hz, 1H), 6.82 (d, J = 5.7 Hz, 1H), 6.32 (d, J = 5.7 Hz, 1H). ^{13}C NMR (100 MHz, $CDCl_3$) δ 138.74, 136.81, 133.68, 133.63, 132.33, 131.64, 129.43, 129.38, 129.32, 128.92, 128.21, 126.33, 126.11, 126.04, 125.25, 125.20, 124.82, 109.92. HRMS (DART): m/z $[M+H]^+$ calcd for $[C_{18}H_{12}BrS_2]$ 370.9558, found 370.9557. IR (KBr): 3084, 3048, 1532, 1380, 961, 843, 789, 724 cm^{-1}.

Synthesis of 3a

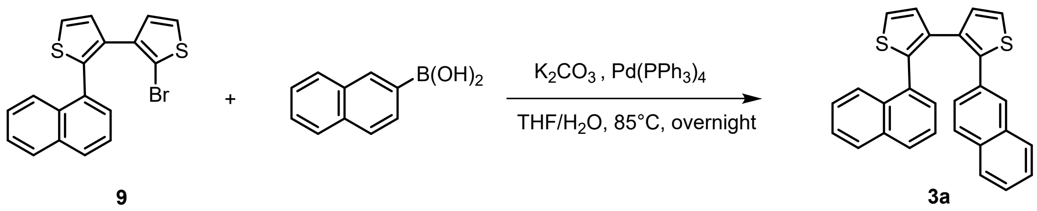

A mixture of 9 (100.0 mg, 0.27 mmol), 2-naphthaleneboronic acid (55.0 mg, 0.32 mmol), K_2CO_3 (85.6 mg, 0.62 mmol), and $Pd(PPh_3)_4$ (15.6 mg, 0.01 mmol) and deoxidized water (0.6 mL) were added into THF (10 mL) and stirred under Ar atmosphere for 36 h at 85 °C. The solution was concentrated under reduce pressure. The crude product was extracted with dichloromethane and water, dried over anhydrous $MgSO_4$. After the solvent was removed under vacuum, the residue was purified by silica gel column chromatography with petrol ether (60-90 °C) as the eluent and was washed by ether to afford **3a** as a yellow solid (95.3 mg, 84%). M.P.: 163-165 °C. ^{1}H NMR (400 MHz, CD_2Cl_2) δ 7.74 (dd, J_1 = 8.9 Hz, J_2 = 3.7 Hz, 2H), 7.68 (dd, J_1 = 8.1 Hz, J_2 = 5.0 Hz, 2H), 7.64 - 7.59 (m, 2H), 7.47-7.43 (m, 2H), 7.42-7.40 (m, 2H), 7.31 (t, J = 7.5 Hz, 1H), 7.18-7.43 (m, 2H), 7.10 (dd, J_1 = 5.2 Hz, J_2 = 2.1 Hz, 2H), 7.07 (d, J = 8.0 Hz, 1H), 7.02 (d, J = 6.2 Hz, 1H), 6.85 (d, J = 5.2 Hz, 1H). ^{13}C NMR (100 MHz, CD_2Cl_2) δ 140.36, 138.25, 135.53, 134.02, 133.83, 133.55, 132.97, 132.50, 132.36, 132.14, 130.86, 130.44, 129.67, 128.82, 128.48, 128.34, 128.30, 128.04, 128.00, 127.32, 126.66, 126.51, 126.23, 126.15, 126.12, 125.51, 125.38, 124.52. HRMS (MALDI): m/z $[M]^+$ calcd for $[C_{28}H_{18}S_2]$ 418.08427, found 418.08444. IR (KBr): 3046, 1590, 1500, 847, 805, 730 cm^{-1}.

Synthesis of 3b

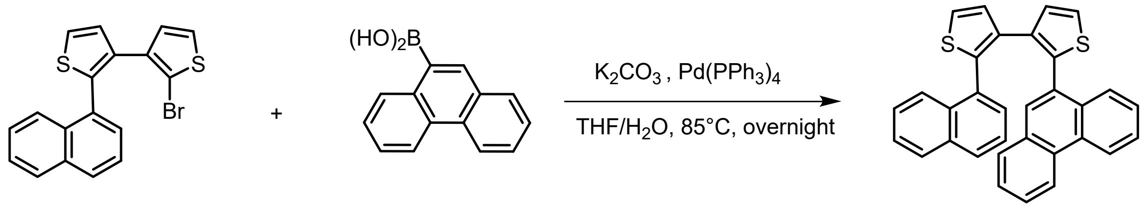

9

3b

A mixture of **9** (100.0 mg, 0.27 mmol), 9-phenanthreneboronic acid (71.9 mg, 0.32 mmol), K_2CO_3 (85.6 mg, 0.62 mmol), and $Pd(PPh_3)_4$ (15.6 mg, 0.01 mmol) and deoxidized water (0.6 mL) were added into THF (10 mL) and stirred under Ar atmosphere for 36 h at 85 °C. The solution was concentrated under reduce pressure. The crude product was extracted with dichloromethane and water, dried over anhydrous $MgSO_4$. After the solvent was removed under vacuum, the residue was purified by silica gel column chromatography (eluent: PE/DCM = 5/1) to afford **3b** as a yellow solid (116.0 mg, 92%). M.P.: 215-218 °C. ^{1}H NMR (400 MHz, $CDCl_3$) δ 8.57 (d, J = 8.3 Hz, 1H), 8.54 (d, J = 8.3 Hz, 1H), 7.63-7.49 (m, 7H), 7.48 (d, J = 8.3 Hz, 1H), 7.37 (d, J = 10.7 Hz, 1H), 7.35-7.31 (m, 1H), 7.25-7.22 (m, 1H), 7.21 (d, J = 5.3 Hz, 1H), 7.09 (d, J = 5.3 Hz, 1H), 7.08-7.04 (m, 3H), 6.99 (t, J = 6 Hz, 1H), 6.87 (d, J = 5.8 Hz, 1H). ^{13}C NMR (125 MHz, $CDCl_3$) δ 137.59, 137.37,

135.04, 134.90, 133.50, 131.91, 131.22, 130.65, 130.48, 130.30, 129.87, 129.68, 129.46, 128.94, 128.77, 128.41, 127.75, 126.80, 126.76, 126.50, 126.36, 126.25, 125.72, 125.56, 124.99, 124.85, 124.81, 122.55, 122.48. HRMS (MALDI): m/z $[M]^+$ calcd for $[C_{32}H_{20}S_2]$ 468.09832, found 468.10009. IR (KBr): 3048, 1496, 1439, 1376, 850, 790, 719 cm^{-1}

Synthesis of 3c

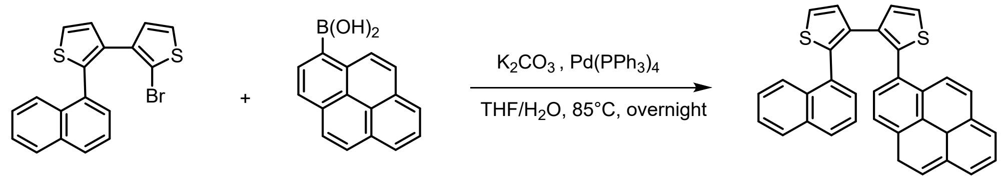

A mixture of **9** (100.0 mg, 0.27 mmol), 2-pyrenylboronic acid (79.5 mg, 0.32 mmol), K_2CO_3 (85.6 mg, 0.62 mmol), and $Pd(PPh_3)_4$ (15.6 mg, 0.01 mmol) and deoxidized water (0.3 mL) were added into toluene (7 mL) and stirred under Ar atmosphere for 36 h at 85 °C. The solution was concentrated under reduce pressure. The crude product was extracted with dichloromethane and water, dried over anhydrous $MgSO_4$. After the solvent was removed under vacuum, the residue was purified by silica gel column chromatography (eluent: PE/DCM = 5/1) to afford **3c** as a yellow solid (116.0 mg, 92%). M.P.: 192-194 °C. ^{1}H NMR (400 MHz, $CDCl_3$) δ 8.17 (d, J = 7.5 Hz, 1H), 8.11 (d, J = 7.5 Hz, 1H), 8.01 (dd, J_1 = 16.1 Hz, J_2 = 8.3 Hz, 2H), 7.88 (d, J = 8.8 Hz, 1H), 7.76 (d, J = 9.2 Hz, 1H), 7.72 (d, J = 7.8 Hz, 1H), 7.68 (d, J = 9.2 Hz, 1H), 7.49 (d, J = 8.2 Hz, 1H), 7.43 (d, J = 8.2 Hz, 1H), 7.35 (dd, J_1 = 6.5 Hz, J_2 = 3.2 Hz, 2H), 7.23 (d, J = 5.3 Hz, 1H), 7.14 (d, J = 5.3 Hz, 2H), 7.11-7.07 (m, 2H), 6.94 (t, J = 7.6 Hz, 1H), 6.81 (d, J = 6.9 Hz, 1H), 6.70 (t, J = 8 Hz, 1H). ^{13}C NMR (100 MHz, $CDCl_3$) δ 138.09, 137.68, 135.23, 134.81, 133.52, 131.71, 131.67, 131.38, 130.93, 130.85, 129.78, 129.65, 129.23, 128.74, 128.60, 128.15, 127.57, 127.52, 127.44, 127.17, 125.95, 125.43, 125.39, 125.33, 125.16, 125.07, 124.99, 124.84, 124.76, 124.61, 124.45. HRMS (MALDI): m/z $[M]^+$ calcd for $[C_{34}H_{20}S_2]$ 492.10099, found 492.10009. IR (KBr): 3038, 1591, 1536, 844, 782, 764, 717 cm^{-1}.

Synthesis of 3d

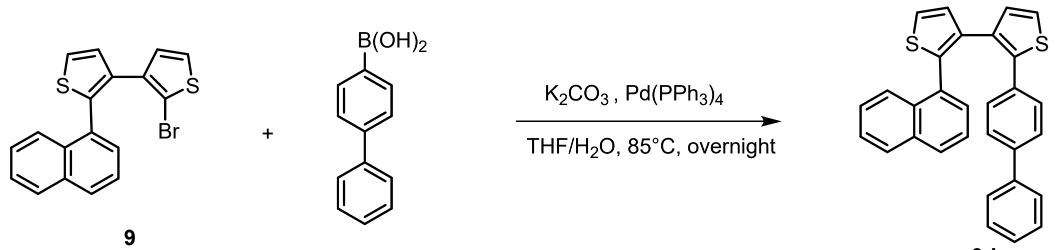

9

3d

A mixture of **9** (100.0 mg, 0.27 mmol), 4-biphenylboronicacid (64.0 mg, 0.32 mmol), K_2CO_3 (85.6 mg, 0.62 mmol), and $Pd(PPh_3)_4$ (15.6 mg, 0.01 mmol) and deoxidized water (0.6 mL) were added into THF (10 mL) and stirred under Ar atmosphere for 36 h at 85 °C. The solution was concentrated under reduce pressure. The crude product was extracted with dichloromethane and water, dried over anhydrous $MgSO_4$. After the solvent was removed under vacuum, the residue was purified by silica gel column chromatography (eluent: PE/DCM = 5/1) to afford **3d** as a yellow solid (97.6 mg, 81%). M.P.: 177-179 °C. ^1H NMR (400 MHz, CD_2Cl_2) δ 7.79 (d, J = 8.2 Hz, 1H), 7.74 (t, J = 8.3 Hz, 2H), 7.59-7.55 (m, 2H), 7.48-7.44 (m, 3H), 7.39-7.33 (m, 4H), 7.28-7.22 (m, 2H), 7.14 (d, J = 5.2 Hz, 1H), 7.12-7.03 (m, 4H), 6.84 (d, J = 5.2 Hz, 1H). ^{13}C NMR (100 MHz, CD_2Cl_2) δ 141.08, 140.31, 140.06, 138.17, 135.54, 134.10, 134.00, 133.38, 132.42, 132.21, 130.88, 130.44, 129.79, 129.49, 129.34, 128.81, 128.48, 127.92, 127.46, 127.44, 126.35, 126.18, 125.60, 125.57, 124.37. HRMS (MALDI): m/z $[M]^+$ calcd for $[C_{30}H_{20}S_2]$ 444.10061, found 444.10009. IR (KBr): 3049, 1513, 1387, 844, 764, 708, 686 cm^{-1}.

Synthesis of 3e

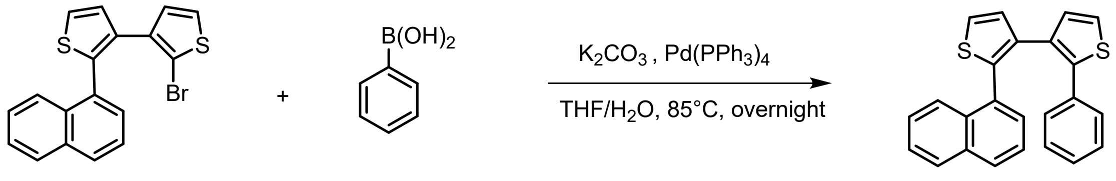

9

3e

A mixture of **9** (434.0 mg, 1.17 mmol), phenylboronic acid (171.0 mg, 1.40 mmol), K_2CO_3 (371.6 mg, 2.69 mmol), and $Pd(PPh_3)_4$ (67.5 mg, 0.06 mmol) and deoxidized water (2.7 mL) were added into THF (45 mL) and stirred under Ar atmosphere for 30 h at 85 °C. The solution was concentrated under reduce pressure. The crude product was extracted with dichloromethane and water, dried over anhydrous $MgSO_4$. After the solvent was removed under vacuum, the residue was purified by silica gel column chromatography (eluent: PE/DCM = 5/1) to afford **3e** as a yellow solid (397.0 mg, 92%). M.P.: 165-167 °C. ^1H NMR (400 MHz, CD_2Cl_4) δ 7.81 (d, J = 7.9 Hz, 1H), 7.75 (d, J = 8.2 Hz, 2H),

7.43-7.39 (m, 2H), 7.31 (t, J = 7.6 Hz, 1H), 7.29-7.25 (m, 1H), 7.17-7.13 (m, 3H), 7.10 (d, J = 7.1 Hz, 1H), 7.06 (d, J = 5.2 Hz, 1H), 7.05-7.02 (m, 3H), 6.77 (d, J = 5.2 Hz, 1H). ^{13}C NMR (100 MHz, $CDCl_3$) δ 139.90, 137.72, 135.08, 134.59, 133.63, 132.68, 132.13, 131.85, 130.31, 129.98, 129.37, 128.82, 128.40, 128.38, 128.10, 127.25, 126.08, 125.96, 125.75, 125.23, 124.89, 123.80. HRMS (MALDI): m/z $[M]^+$ calcd for $[C_{24}H_{16}S_2]$ 368.06696, found 368.06879. IR (KBr): 3046, 1588, 1495, 1439, 1380, 1329, 849, 766, 689 cm^{-1}.

Synthesis of 3f

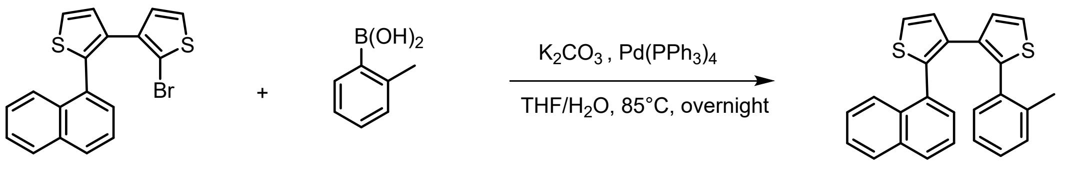

9 3f

A mixture of **9** (200.0 mg, 0.54 mmol), 2-methylbenzeneboronic acid (87.9 mg, 0.65 mmol), K_2CO_3 (171.2 mg, 1.24 mmol), and $Pd(PPh_3)_4$ (31.1 mg, 0.03 mmol) and deoxidized water (2 mL) were added into THF (20 mL) and stirred under Ar atmosphere for 30 h at 85 °C. The solution was concentrated under reduce pressure. The crude product was extracted with dichloromethane and water, dried over anhydrous $MgSO_4$. After the solvent was removed under vacuum, the residue was purified by silica gel column chromatography (eluent: PE/DCM = 5/1) to afford **3f** as a yellow solid (171.0 mg, 83%). M.P.: 139-141 °C. ^{1}H NMR (500 MHz, $CDCl_3$) δ 7.81 (d, J = 8.2 Hz, 1H), 7.77 (d, J = 8.2 Hz, 1H), 7.69 (d, J = 8.5 Hz, 1H), 7.41 (t, J = 7.5 Hz, 1H), 7.31-7.27 (m, 3H), 7.09 - 7.03 (m, 3H), 6.98 (d, J = 7.6 Hz, 1H), 6.93 (d, J = 5.3 Hz, 1H), 6.90 (t, J = 7.5 Hz, 1H), 6.77 (d, J = 5.3 Hz, 2H), 1.84 (s, 3H). ^{13}C NMR (100 MHz, $CDCl_3$) δ 138.85, 137.23, 135.05, 133.93, 133.86, 133.75, 132.33, 132.13, 131.19, 130.17, 129.38, 129.19, 129.06, 128.40, 128.08, 127.89, 126.18, 126.07, 125.81, 125.58, 125.42, 124.84, 124.10, 20.27. HRMS (DART): m/z $[M+H]^+$ calcd for $[C_{25}H_{19}S_2]$ 383.0923, found 383.0920. IR (KBr): 3051, 2959, 2917, 1541, 1497, 1446, 1378, 853, 768, 722 cm^{-1}.

Synthesis of 3g

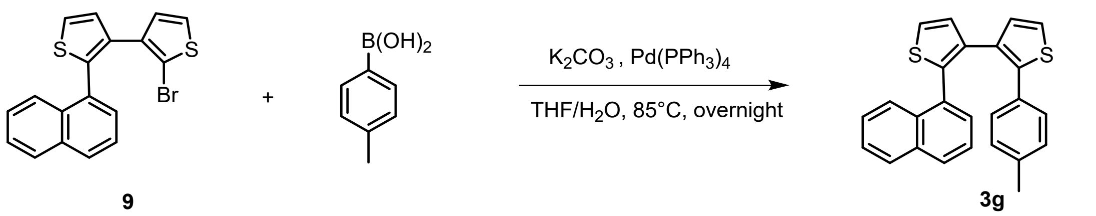

9 3g

A mixture of **9** (150.0 mg, 0.40 mmol), 4-methylbenzeneboronic acid (65.9 mg, 0.49 mmol), K_2CO_3 (128.4 mg, 0.93 mmol), and $Pd(PPh_3)_4$ (23.3 mg, 0.02 mmol) and deoxidized water (1 mL) were added into THF (15 mL) and stirred under Ar atmosphere for 30 h at 85 °C. The solution was concentrated under reduce pressure. The crude product was extracted with dichloromethane and water, dried over anhydrous $MgSO_4$.After the solvent was removed under vacuum, the residue was purified by silica gel column chromatography (eluent: PE/DCM = 5/1) to afford **3g** as a yellow solid (143.6 mg, 95%). M.P.: 189-191 °C. ^{1}H NMR (500 MHz, $CDCl_3$) δ 7.78 (d, J = 9.4 Hz, 2H), 7.74 (d, J = 8.0 Hz, 1H), 7.39 (t, J = 7.5 Hz, 1H), 7.36 (d, J = 5.2 Hz, 1H), 7.31-7.27 (m, 2H), 7.13 (d, J = 7.1 Hz, 1H), 7.03 (d, J = 5.2 Hz, 1H), 6.97 (d, J = 5.2 Hz, 1H), 6.95-6.91 (m, 4H), 6.71 (d, J = 5.2 Hz, 1H), 2.26 (s, 3H). ^{13}C NMR (100 MHz, $CDCl_3$) δ 140.06, 137.57, 137.01, 135.24, 133.68, 132.35, 132.21, 131.91, 131.66, 130.28, 130.03, 129.33, 129.11, 128.67, 128.39, 128.06, 126.16, 125.85, 125.68, 125.16, 124.82, 123.41, 21.26. HRMS (DART): m/z [M+H]$^+$ calcd for [$C_{25}H_{19}S_2$] 383.0923, found 383.0921. IR (KBr): 3042, 2908, 1505, 1428, 1381, 847, 805, 724 cm^{-1}.

Synthesis of 4a

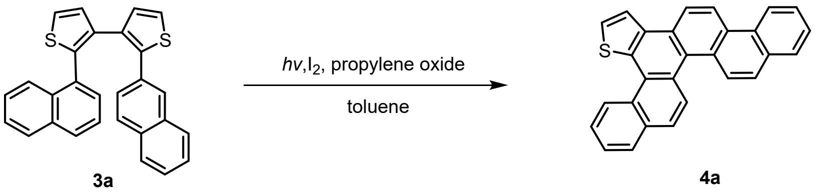

A mixture of **3a** (60.0 mg, 0.14 mmol), I_2 (109.1 mg, 0.43 mmol, propylene oxide (0.04 mL) was added into anhydrous toluene (90 mL) under argon. Then equally distributed into 6 quartz tubes. The reaction mixture was irradiation with a high-pressure Hg lamp for 17 h. After being quenched with $Na_2S_2O_3$, the reaction mixture was extracted with CH_2Cl_2 and H_2O, and then dried over $MgSO_4$. After the solvent was removed under vacuum, the residue was purified by silica gel column chromatography (eluent: PE/DCM = 5/1) and subsequently precipitated from a mixture of CH_2Cl_2/ CH_3OH to afford **4a** as a grayish-white solid (47.6 mg, 85%). M.P.: 266-269 °C. ^{1}H NMR (400 MHz, $CDCl_3$) δ 9.31 (d, J = 8.5 Hz, 1H), 8.98 (d, J = 8.9 Hz, 1H), 8.92 (d, J = 9.0 Hz, 1H), 8.87 (dd, J = 8.7, 3.3 Hz, 2H), 8.65 (d, J = 8.9 Hz, 1H), 8.29 (d, J = 5.6 Hz, 1H), 8.11 (d, J = 7.8 Hz, 1H), 8.02 (d, J = 7.9 Hz, 1H), 7.94 (t, J = 10 Hz, 2H), 7.88 (t, J = 6 Hz, 1H), 7.83 (d, J = 5.5 Hz, 1H), 7.78 - 7.73 (m, 2H), 7.69 (t, J = 7.3 Hz, 1H). ^{13}C NMR (100 MHz, $CDCl_3$) δ 136.40, 134.61, 132.65, 131.72, 130.64, 129.90, 129.75, 128.77, 128.54, 128.38, 127.71, 127.40, 127.27, 127.20, 127.05,

126.87, 126.77, 126.62, 126.61, 126.47, 126.31, 124.74, 123.43, 122.74, 122.60. HRMS (MALDI): m/z $[M+H]^+$ calcd for $[C_{28}H_{17}S]$ 384.10430, found 385.10455. IR (KBr): 3047, 2921, 1467, 827, 744 cm^{-1}.

Synthesis of 4b

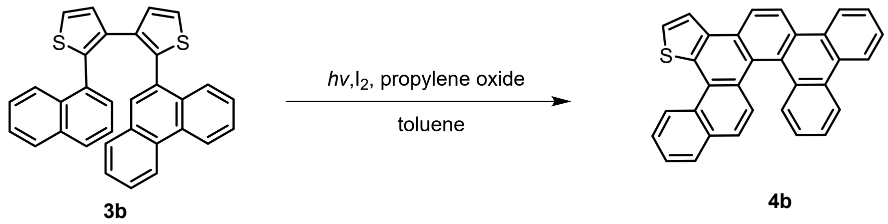

A mixture of **3b** (80.0 mg, 0.17 mmol), I_2 (130.0 mg, 0.51 mmol, propylene oxide (0.08 mL) was added into anhydrous toluene (120 mL) under argon. Then equally distributed into 8 quartz tubes. The reaction mixture was irradiation with a high-pressure Hg lamp for 17 h. After being quenched with $Na_2S_2O_3$, the reaction mixture was extracted with CH_2Cl_2 and H_2O, and then dried over $MgSO_4$. After the solvent was removed under vacuum, the residue was purified by silica gel column chromatography (eluent: PE/DCM = 5/1) and subsequently precipitated from a mixture of CH_2Cl_2/CH_3OH to afford **4b** as a grayish-white solid (22.2 mg, 30%). M.P.: > 300 °C. ^1H NMR (400 MHz, $CDCl_3$) δ 9.31 (d, J = 8.5 Hz, 1H), 8.77 (d, J = 8.7 Hz, 1H), 8.75 - 8.70 (m, 2H), 8.63 (d, J = 8.1 Hz, 1H), 8.58 (d, J = 8.6 Hz, 1H), 8.29 (d, J = 5.5 Hz, 1H), 8.20 (d, J = 9.0 Hz, 1H), 8.08 (d, J = 8.3 Hz, 1H), 7.96 (d, J = 7.6 Hz, 1H), 7.89 - 7.86 (m, 1H), 7.85 (d, J = 5.5 Hz, 1H), 7.79 - 7.74 (m, 2H), 7.70 (t, J = 7.4 Hz, 1H), 7.55 (t, J = 7.5 Hz, 1H), 7.48 (d, J = 9.1 Hz, 1H), 7.17 (t, J = 7.1 Hz, 1H). ^{13}C NMR (100 MHz, $CDCl_3$) δ 136.19, 134.48, 132.95, 131.16, 130.91, 130.48, 130.10, 129.94, 129.91, 129.90, 129.81, 129.34, 128.77, 128.75, 127.82, 127.63, 127.35, 127.00, 126.96, 126.54, 126.51, 126.43, 126.32, 126.12, 125.67, 124.09, 124.01, 123.59, 123.39, 123.23, 122.60, 122.31. HRMS (MALDI): m/z $[M]^+$ calcd for $[C_{32}H_{18}S]$ 434.11257, found 434.11237. IR (KBr): 3043, 1437, 825, 737 cm^{-1}.

Synthesis of 4c

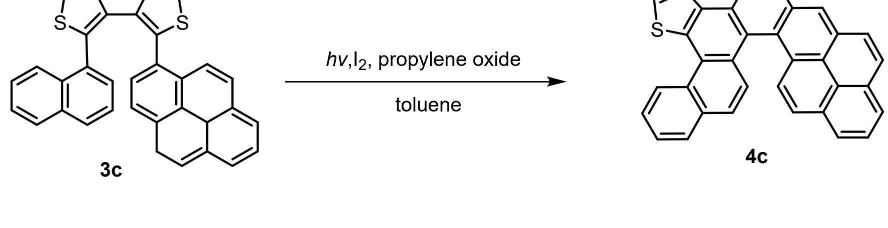

A mixture of **3c** (160.0 mg, 0.32 mmol), I_2 (247.3 mg, 0.97 mmol, propylene oxide (0.16 mL) was added into anhydrous toluene (160 mL) under argon. Then equally distributed into 16 quartz tubes. The reaction mixture was irradiation with a high-pressure Hg lamp for 17 h. After being quenched with $Na_2S_2O_3$, the reaction mixture was extracted with CH_2Cl_2 and H_2O, and then dried over $MgSO_4$. After the solvent was removed under vacuum, the residue was purified by silica gel column chromatography (eluent: PE/DCM = 5/1) and subsequently precipitated from a mixture of CH_2Cl_2/CH_3OH to afford **4c** as a grayish-white solid (70.0 mg, 47%). M.P.: > 300 °C. ^1H NMR (400 MHz, $CDCl_3$) δ 9.37 (d, J = 8.5 Hz, 1H), 8.70 (s, 1H), 8.60 (d, J = 8.6 Hz, 1H), 8.39-8.34 (m, 3H), 8.21 (t, J = 6.5 Hz, 2H), 8.17 (t, J = 6.5 Hz, 2H), 8.10 (d, J = 9.0 Hz, 1H), 8.02 (t, J = 7.4 Hz, 2H), 7.94 (d, J = 8.3 Hz, 1H), 7.89 (t, J = 7.4 Hz, 2H), 7.75 (t, J = 7.4 Hz, 1H), 7.53 (d, J = 8.9 Hz, 1H). ^{13}C NMR (100 MHz, $CDCl_3$) δ 136.37, 134.79, 132.87, 131.85, 131.30, 130.89, 130.29, 129.95, 129.69, 129.30, 128.72, 128.63, 128.44, 128.20, 128.03, 127.82, 126.98, 126.66, 126.59, 126.56, 126.33, 125.76, 125.40, 125.24, 125.07, 124.96, 124.56, 124.54, 124.13, 124.00, 122.91, 122.69. HRMS (MALDI): m/z $[M+H]^+$ calcd for $[C_{34}H_{19}S]$ 459.11987, found 459.12020. IR (KBr): 3034, 1600, 874, 831, 739 cm^{-1}.

Synthesis of 4d

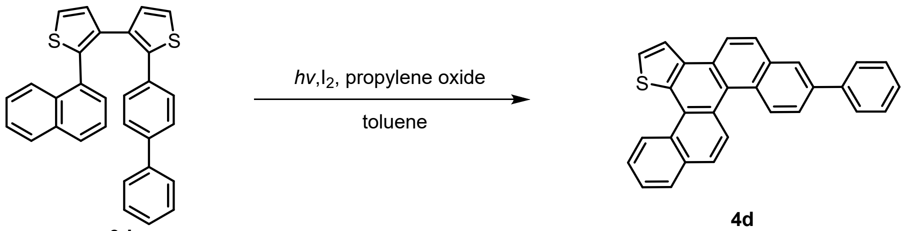

3d 4d

A mixture of **3d** (80.0 mg, 0.18 mmol), I_2 (137.0 mg, 0.54 mmol), propylene oxide (0.08 mL) was added into anhydrous toluene (120 mL) under argon. Then equally distributed into 8 quartz tubes. The reaction mixture was irradiation with a high-pressure Hg lamp for 17 h. After being quenched with $Na_2S_2O_3$, the reaction mixture was extracted with CH_2Cl_2 and H_2O, and then dried over $MgSO_4$. After the solvent was removed under vacuum, the residue was purified by silica gel column chromatography (eluent: PE/DCM = 5/1) and subsequently precipitated from a mixture of CH_2Cl_2/CH_3OH to afford **4d** as a grayish-white solid (28.4 mg, 38%). M.P.: 254-256 °C. ^1H NMR (400 MHz, $CDCl_3$) δ 9.31 (d, J = 8.4 Hz, 1H), 9.03 (dd, J_1 = 8.9 Hz, J_2 = 3.3 Hz, 2H), 8.48 (d, J = 8.8 Hz, 1H), 8.26 (dd, J_1 = 10.0 Hz, J_2 = 3.7 Hz, 2H), 8.11 (d, J = 9.0 Hz, 2H), 7.95 (d, J = 9.0 Hz, 2H),

7.86 (t, J = 8.1 Hz, 1H), 7.83 (t, J = 6 Hz, 3H), 7.75 (t, J = 7.4 Hz, 1H), 7.55 (t, J = 7.6 Hz, 2H), 7.43 (t, J = 7.3 Hz, 1H). ^{13}C NMR (100 MHz, $CDCl_3$) δ 140.74, 138.45, 136.59, 134.61, 133.56, 132.62, 129.92, 129.65, 129.51, 129.12, 128.60, 128.50, 127.95, 127.78, 127.68, 127.52, 127.13, 127.05, 126.60, 126.55, 126.47, 126.42, 126.25, 125.47, 125.05, 122.84, 122.59. HRMS (MALDI): m/z $[M]^+$ calcd for [$C_{30}H_{18}S$] 410.11080, found 410.11237. IR (KBr): 3037, 2921, 1491, 1436, 840, 749, 692 cm^{-1}.

Synthesis of 4e

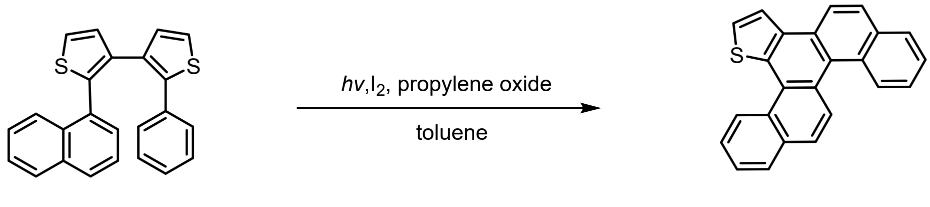

A mixture of **3e** (160.0 mg, 0.43 mmol), I_2 (330 mg, 1.30 mmol, propylene oxide (0.16 mL) was added into anhydrous toluene (160 mL) under argon. Then equally distributed into 16 quartz tubes. The reaction mixture was irradiation with a high-pressure Hg lamp for 17 h. After being quenched with $Na_2S_2O_3$, the reaction mixture was extracted with CH_2Cl_2 and H_2O, and then dried over $MgSO_4$. After the solvent was removed under vacuum, the residue was purified by silica gel column chromatography (eluent: PE/DCM = 5/1) and subsequently precipitated from a mixture of CH_2Cl_2/CH_3OH to afford **4e** as a grayish-white solid (48.2 mg, 33%). M.P.: 167-170 °C. ^{1}H NMR (100 MHz, $CDCl_3$) δ 9.30 (d, J = 8.5 Hz, 1H), 9.00 (d, J = 9.0 Hz, 1H), 8.96 (d, J = 8.3 Hz, 1H), 8.45 (d, J = 8.7 Hz, 1H), 8.24 (d, J = 5.6 Hz, 1H), 8.10 (d, J = 8.3 Hz, 1H), 8.06 (t, J = 7.5 Hz, 2H), 7.92 (d, J = 9.0 Hz, 1H), 7.88 (t, J = 7.7 Hz, 1H), 7.81 (d, J = 5.5 Hz, 1H), 7.74 (t, J = 7.7 Hz, 1H), 7.68 (t, J = 7.5 Hz, 1H), 7.64 (t, J = 6.7 Hz, 1H). ^{13}C NMR (100 MHz, $CDCl_3$) δ 136.60, 134.61, 133.24, 132.61, 130.52, 129.94, 129.02, 128.64, 128.48, 128.40, 128.04, 127.83, 127.11, 127.03, 127.01, 126.58, 126.53, 126.47, 126.35, 126.26, 125.97, 124.98, 122.61, 122.42. HRMS (MALDI): m/z $[M]^+$ calcd for [$C_{24}H_{14}S$] 334.07942, found 334.08107. IR (KBr): 3041, 2919, 1425, 1368, 821, 784, 740 cm^{-1}.

Synthesis of 4f

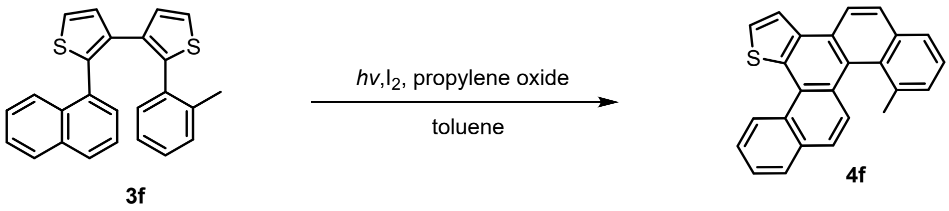

A mixture of **3f** (90.0 mg, 0.24 mmol), I_2 (238.9 mg, 0.94 mmol, propylene oxide (0.16 mL) was added into anhydrous toluene (135 mL) under argon. Then equally distributed into 9 quartz tubes. The reaction mixture was irradiation with a high-pressure Hg lamp for 17 h. After being quenched with $Na_2S_2O_3$, the reaction mixture was extracted with CH_2Cl_2 and H_2O, and then dried over $MgSO_4$. After the solvent was removed under vacuum, the residue was purified by silica gel column chromatography (eluent: PE/DCM = 5/1) and subsequently precipitated from a mixture of CH_2Cl_2/ CH_3OH to afford **4f** as a grayish-white solid (8.8 mg, 11%). M.P.: 162-164 °C. ^{1}H NMR (400 MHz, $CDCl_3$) δ 9.29 (d, J = 8.4 Hz, 1H), 8.35 (d, J = 8.6 Hz, 1H), 8.25 (d, J = 5.6 Hz, 1H), 8.08-8.05 (m, 2H), 8.00 (d, J = 8.6 Hz, 1H), 7.92-7.87 (m, 2H), 7.83 (d, J = 5.6 Hz, 1H), 7.81 (d, J = 8.9 Hz, 1H), 7.73 (t, J = 7.3 Hz, 1H), 7.63 (t, J = 7.5 Hz, 1H), 7.55 (d, J = 7.1 Hz, 1H), 2.37 (s, 3H). ^{13}C NMR (100 MHz, $CDCl_3$) δ 136.43, 136.30, 134.41, 133.75, 132.37, 130.38, 129.84, 129.82, 129.66, 128.73, 128.68, 128.45, 128.04, 126.82, 126.49, 126.37, 126.03, 125.70, 125.28, 124.59, 122.53, 121.71, 24.97. HRMS (DART): m/z $[M+H]^+$ calcd for $[C_{25}H_{17}S]$ 349.1045, found 349.1045. IR (KBr): 3039, 2910, 1440, 812, 737 cm^{-1}.

Synthesis of 4g

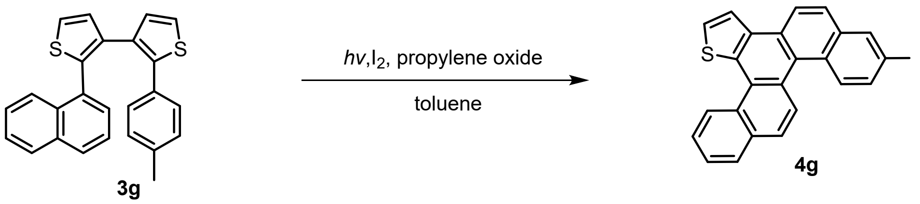

A mixture of **3g** (140.0 mg, 0.37 mmol), I_2 (278.7 mg, 1.10 mmol, propylene oxide (0.14 mL) was added into anhydrous toluene (140 mL) under argon. Then equally distributed into 14 quartz tubes. The reaction mixture was irradiation with a high-pressure Hg lamp for 6 h. After being quenched with $Na_2S_2O_3$, the reaction mixture was extracted with CH_2Cl_2 and H_2O, and then dried over $MgSO_4$. After the solvent was removed under vacuum, the residue was purified by silica gel column chromatography (eluent: PE/DCM = 5/1) and subsequently precipitated from a mixture of CH_2Cl_2/CH_3OH to afford **4g** as a grayish-white solid (24.4 mg, 19%). M.P.: 247-249 °C. ^{1}H NMR

(400 MHz, $CDCl_3$) δ 9.30 (d, J = 8.4 Hz, 1H), 8.99 (d, J = 9.0 Hz, 1H), 8.85 (d, J = 8.6 Hz, 1H), 8.42 (d, J = 8.7 Hz, 1H), 8.23 (d, J = 5.6 Hz, 1H), 8.09 (d, J = 6.8 Hz, 1H), 7.98 (d, J = 8.7 Hz, 1H), 7.91 (d, J = 9.0 Hz, 1H), 7.88-7.85 (m, 2H), 7.80 (d, J = 5.5 Hz, 1H), 7.73 (t, J = 7.4 Hz, 1H), 7.52 (d, J = 8.6 Hz, 1H), 2.63 (s, 3H). ^{13}C NMR (100 MHz, $CDCl_3$) δ 136.65, 135.72, 134.21, 133.47, 132.59, 129.95, 128.86, 128.50, 128.45, 128.18, 128.06, 128.04, 128.01, 127.81, 126.96, 126.94, 126.69, 126.50, 126.47, 126.43, 126.39, 124.86, 122.58, 122.42, 21.54. HRMS (DART): m/z $[M+H]^+$ calcd for $[C_{25}H_{17}S]$ 349.1045, found 349.1046. IR (KBr): 3050, 2972, 2917, 1610, 883, 734 cm^{-1}.

Scheme S3. Synthesis of compounds 6c and 6d.

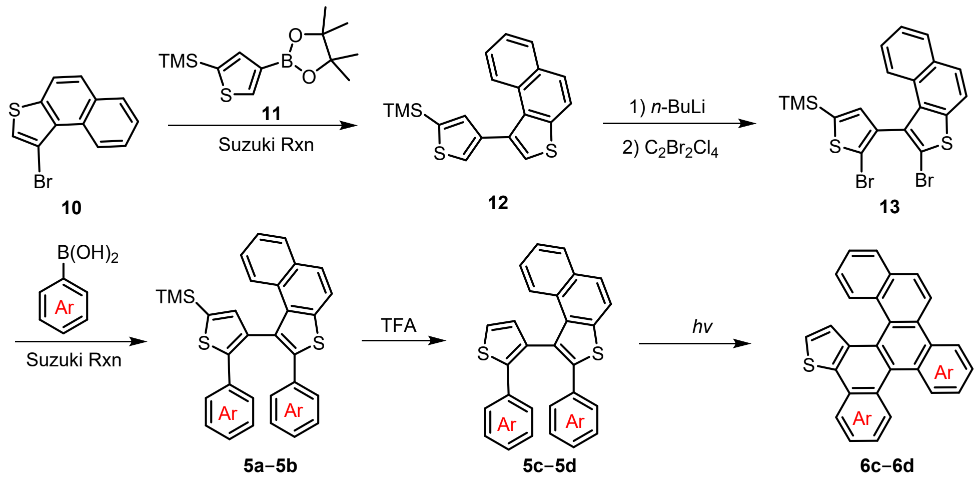

Synthesis of 12

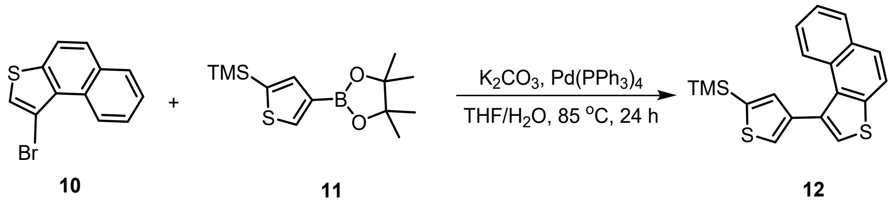

A mixture of **10** (100.0 mg, 0.38 mmol), **11** (214.5 mg, 0.76 mmol), K_2CO_3 (157.6 mg, 1.14 mmol), and $Pd(PPh_3)_4$ (22.0 mg, 0.02 mmol) and deoxidized water (1 mL) were added into THF (10 mL) and stirred under Ar atmosphere for 24 h at 85 °C. The solution was concentrated under reduce pressure. The crude product was extracted with dichloromethane and water, dried over anhydrous $MgSO_4$. After the solvent was removed under vacuum, the residue was purified by silica gel column chromatography (eluent: PE/DCM = 5/1) to afford **12** as a yellow solid (12.3 mg, 87%). M.P.: 73-74 ^oC. ^1H NMR (400 MHz, $CDCl_3$) δ 7.92 (d, J = 8.0 Hz, 1H), 7.89 (d, J = 8.8 Hz, 1H), 7.85 (d, J = 8.4 Hz, 1H), 7.76 (d, J = 8.4 Hz, 1H), 7.63 (d, J = 0.8 Hz, 1H), 7.48-7.44 (m, 1H), 7.42 (s, 1H), 7.35 (d,

J = 1.2 Hz, 1H), 7.32-7.28 (m, 1H), 0.38 (s, 9H); ^{13}C NMR (100 MHz, $CDCl_3$) δ 140.84, 140.19, 138.69, 136.38, 134.80, 133.10, 131.92, 130.03, 128.74, 125.81, 125.76, 125.13, 124.80, 123.87, 121.03, 0.14; HRMS (MALDI) *m/z* $[M]^+$ calcd for $[C_{19}H_{18}S_2Si]$ 338.0614; found 338.0616; IR (KBr): 3118, 2956, 1365, 1244, 1006, 839, 764 cm^{-1}.

Synthesis of 13

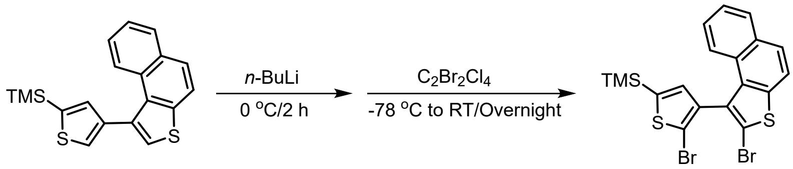

12 13

n-BuLi (2.4 M in hexane, 0.56 mL, 1.34 mmol) was transferred by syringe into the solution of **12** (206.0 mg, 0.61 mmol) in anhydrous Et_2O (20 mL) at -78 °C. After keeping at -78 °C for 2 h, $C_2Br_2Cl_4$ (495.0 mg, 1.52 mmol) was added at -78 °C, and then the reaction mixture was warmed up slowly to ambient temperature overnight. The reaction mixture was quenched with CH_3OH at -78 °C, and extracted with CH_2Cl_2 and H_2O, and then dried over $MgSO_4$. After the solvent was removed under vacuum, the residue was purified by silica gel column chromatography with petrol ether (60-90 °C) as the eluent to afford **13** as a yellow solid (282.6 mg, 93.6%). M.P.: 98-99 °C. ^{1}H NMR (400 MHz, $CDCl_3$) δ 7.90 (d, J = 8.0 Hz, 1H), 7.77 (s, 2H), 7.50-7.44 (m, 2H), 7.34-7.30 (m, 1H), 7.07 (s, 1H), 0.36 (s, 9H); ^{13}C NMR (100 MHz, $CDCl_3$) δ 143.1, 138.8, 138.1, 135.8, 133.0, 132.9, 131.8, 129.1, 128.8, 126.6, 126.1, 125.5, 122.7, 119.9, 117.9, 115.7, 0.0; HRMS (EI) *m/z* $[M]^+$ calcd for $[C_{19}H_{16}Br_2S_2Si]$ 493.8824; found 493.8823; IR (KBr): 3058, 2949, 1369, 1249, 987, 837, 800 cm^{-1}.

Synthesis of 5a

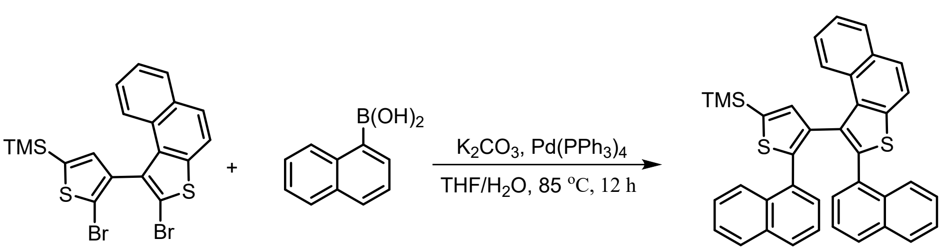

13 5a

A mixture of **13** (92.0 mg, 0.22 mmol), 1-naphthaleneboronic acid (78.3 mg, 0.46 mmol), K_2CO_3 (119.9 mg, 0.87 mmol), and $Pd(PPh_3)_4$ (25.1 mg, 0.02 mmol) and deoxidized water (1 mL) were added into THF (10 mL) and stirred under Ar atmosphere for 12 h at 85 °C. The solution was concentrated under reduce pressure. The crude product was extracted with dichloromethane and water, dried over anhydrous $MgSO_4$. After the solvent was removed under vacuum, the residue was

purified by silica gel column chromatography with petrol ether (60-90 °C) as the eluent to afford **5a** as a yellow solid (91.3 mg, 81.2%). M.P.: 179-180 °C. ^{1}H NMR (400 MHz, $CDCl_3$) δ 8.13 (d, J = 8.4 Hz, 1H), 8.04 (d, J = 8.4 Hz, 1H), 7.88-7.82 (m, 2H), 7.58-7.42 (m, 8H), 7.26-7.09 (m, 4H), 6.88-6.71(m, 5H), 0.35 (s, 9H); ^{13}C NMR (100 MHz, $CDCl_3$) δ 144.4, 140.2, 138.3, 138.0, 137.0, 134.6, 133.4, 133.2 , 132.4, 132.3, 132.0, 131.6, 131.4, 129.2, 129.1, 128.4, 128.3, 128.0, 127.6, 127.5, 126.2, 125.6, 125.4, 125.3, 125.2, 124.8, 124.5, 124.0, 120.7, 0.0; HRMS (MALDI) *m/z* $[M]^+$ calcd for $[C_{39}H_{30}S_2Si]$ 590.1553; found 590.1566; IR (KBr): 3055, 2953, 1253, 1009, 841, 771, 740 cm^{-1}.

Synthesis of 5c

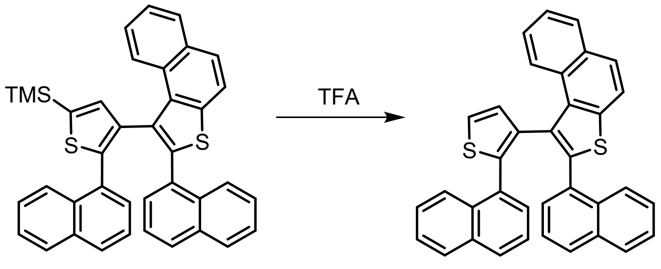

Compound **5a** (740.0 mg, 1.252 mmol, 1.0 eq.) was dissolved in dichloromethane (30 mL). TFA (2 mL) was slowly added dropwise to the above reaction mixture, and the reaction was kept at room temperature for 0.5 hours. The reaction mixture was quenched with saturated $NaHCO_3$.The solution was concentrated under reduce pressure. The crude product was extracted with dichloromethane and water, dried over anhydrous $MgSO_4$. After the solvent was removed under vacuum, the residue was purified by silica gel column chromatography with petrol ether (60-90 °C) as the eluent to afford **5c** as a yellow solid (639.9 mg, 98.5%). M.P.: 250-251 °C. ^{1}H NMR (400 MHz, $CDCl_3$) δ ^{1}H NMR (400 MHz, $CDCl_3$) δ 8.13 (d, J = 8.4 Hz, 1H), 8.03 (d, J = 8.0 Hz, 1H), 7.88-7.82 (m, 2H), 7.60-7.45 (m, 8H), 7.34 (d, J = 4.8 Hz, 1H), 7.27-7.07(m, 4H), 6.87-6.76 (m, 5H); ^{13}C NMR (100 MHz, $CDCl_3$) δ 139.41, 138.30, 135.56, 134.66, 133.40, 133.23, 132.32, 132.22, 132.15, 131.33, 131.19, 131.16, 130.98, 130.95, 130.25, 129.23, 129.13, 128.55, 128.42, 128.17, 127.54, 126.42, 125.72, 125.51, 125.37, 124.79, 124.56, 123.82, 120.63; HRMS (MALDI) *m/z* $[M]^+$ calcd for $[C_{36}H_{22}S_2]$ 518.1157; found 518.1159; IR (KBr): 3045, 1589, 1500, 1373, 794, 771, 744 cm^{-1}.

Synthesis of 5b

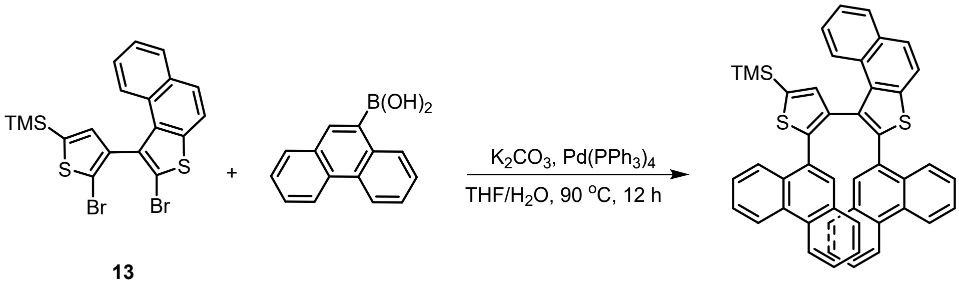

13

5b

A mixture of **19** (930.0 mg, 1.87 mmol), 9-phenanthracenylboronic acid (873.7 mg, 3.94 mmol), K_2CO_3 (1.0358 g, 7.49 mmol), and $Pd(PPh_3)_4$ (216.5 mg, 0.19 mmol) and deoxidized water (8 mL) were added into THF (80 mL) and stirred under Ar atmosphere for 12 h at 90 °C. The solution was concentrated under reduce pressure. The crude product was extracted with dichloromethane and water, dried over anhydrous $MgSO_4$. After the solvent was removed under vacuum, the residue was purified by silica gel column chromatography with silica gel column chromatography (eluent: PE/DCM = 10/1) to afford **5b** as a yellow solid (722.3 mg, 55.8%). M.P.: 282-283 °C. ^{1}H NMR (400 MHz, $CDCl_3$) δ 8.38 (d, J = 8.4 Hz, 2H), 8.34-8.01 (m, 3H), 7.91 (d, J = 8.0 Hz, 1H), 7.68 (s, 2H), 7.63-7.38 (m, 4H), 7.38-7.25 (m, 3H), 7.25-7.11 (m, 2H), 7.03 (t, J = 7.4 Hz, 1H), 6.87-6.49 (m, 3H), 6.48-6.28 (m, 2H),0.36 (s, 9H); ^{13}C NMR (100 MHz, $CDCl_3$) δ 144.57, 140.11, 138.43, 138.19, 137.09, 134.30, 132.66, 132.60, 132.35, 130.94, 130.60, 130.30, 130.22, 130.02, 129.98, 129.45, 129.15, 128.79, 126.79, 126.53, 126.42, 126.37, 126.21, 126.03, 125.82, 125.73, 125.22, 124.15, 122.07, 122.00, 120.67, 0.05; HRMS (MALDI) *m/z* $[M]^+$ calcd for $[C_{47}H_{34}S_2Si]$ 690.1866; found 690.1870; IR (KBr): 3060, 2954, 1448, 1248, 1010, 837, 742 cm^{-1}.

Synthesis of 5d

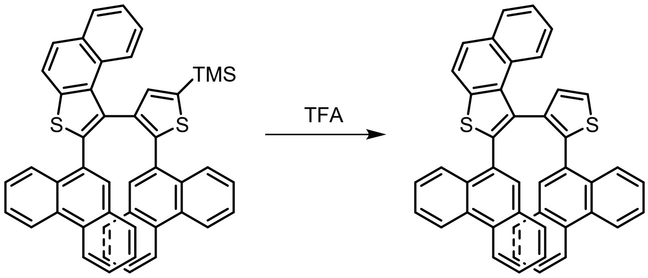

5b 5d

Compound **5b** (700.0 mg, 1.01 mmol) was dissolved in dichloromethane (30 mL). TFA (2 mL) was slowly added dropwise to the above reaction mixture, and the reaction was kept at room temperature for 0.5 hours. The reaction mixture was quenched with saturated $NaHCO_3$. The solution was concentrated under reduce pressure. The crude product was extracted with dichloromethane and

water, dried over anhydrous $MgSO_4$. After the solvent was removed under vacuum, the residue was purified by silica gel column chromatography with petrol ether (60-90 °C) as the eluent to afford **5d** as a yellow solid (619.7 mg, 98.8%). M.P.: 297-298 °C. ^1H NMR (400 MHz, $CDCl_3$) δ 8.56-8.25 (m, 5H), 8.08 (d, J = 8.4 Hz, 1H), 7.85 (s, 2H), 7.62-7.41 (m, 9H), 7.32-7.28 (m, 2H), 7.23-7.20 (m, 1H), 6.98-6.96 (m, 3H), 6.68-6.29 (m, 3H); ^{13}C NMR (100 MHz, $CDCl_3$) δ 139.51, 138.35, 135.65, 134.43, 132.39, 130.92, 130.83, 130.29, 130.13, 130.06, 129.78, 129.21, 128.93, 128.83, 126.88, 126.67, 126.44, 126.32, 126.09, 125.83, 125.48, 125.23, 124.00, 122.13, 122.08, 120.65; HRMS (MALDI) *m/z* $[M]^+$ calcd for $[C_{44}H_{26}S_2]$ 618.1470; found 618.1464; IR (KBr): 3066, 1697, 1600, 1450, 1373, 1245, 744 cm^{-1}.

Synthesis of 6c

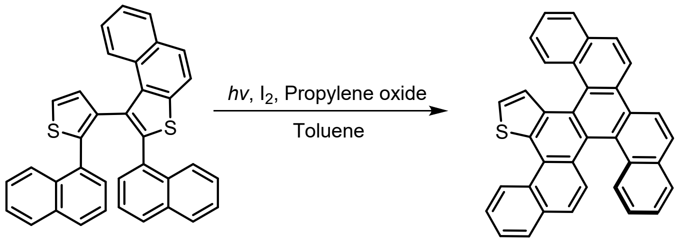

5c 6c

A mixture of **5c** (40.0 mg, 0.08mmol), I_2 (58.7 mg,0.23mmol), propylene oxide (0.14 mL) was added into anhydrous toluene (20 mL) under argon. The reaction mixture was irradiation with a high-pressure Hg lamp for 1h. After being quenched with $Na_2S_2O_3$, the reaction mixture was extracted with CH_2Cl_2 and H_2O, and then dried over $MgSO_4$. After the solvent was removed under vacuum, the residue was purified by silica gel column chromatography (eluent: PE/DCM = 7/1) to afford **6c** as a yellow solid (14.9 mg, 39.8%). M.P.: >300 °C. ^1H NMR (400 MHz, $CDCl_3$) δ 9.40 (d, J = 8.5 Hz, 1H), 8.03-7.99 (m, 2H), 7.90 (d, J = 8.5 Hz, 1H), 7.88-7.83 (m, 2H), 7.70-7.61 (m, 4H), 7.52 (t, J = 7.5 Hz, 1H), 7.49-7.44 (m, 3H), 7.32 (t, J = 7.5 Hz, 1H), 7.23 (t, J = 7.5 Hz, 1H), 7.00-6.97 (m, 2H), 6.11 (d, J = 13.0 Hz, 1H), 5.90 (d, J = 13.0 Hz, 1H); ^{13}C NMR (100 MHz, $CDCl_3$) δ 138.50, 137.76, 136.41, 135.75, 134.93, 134.79, 134.21, 133.94, 133.74, 133.29, 133.17, 132.97, 132.92, 132.50, 130.72, 129.76, 129.65, 129.14, 129.07, 129.00, 128.45, 128.19, 127.92, 127.82, 127.18, 126.76, 126.57, 126.40, 126.26, 126.02, 126.00, 125.81, 125.66, 121.10; HRMS (MALDI) *m/z* $[M]^+$ calcd for $[C_{36}H_{20}S]$ 484.1280; found 484.1273; IR (KBr): 3041, 2920, 1620, 1267, 1169, 792, 737 cm^{-1}.

Synthesis of 6d

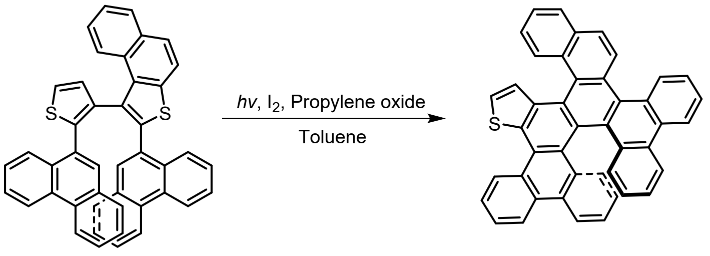

A mixture of **5d** (100.0 mg, 0.16 mmol), I_2 (164.1 mg,0.65 mmol), propylene oxide (0.14 mL) was added into anhydrous toluene (50 mL) under argon. The reaction mixture was irradiation with a high-pressure Hg lamp for 6 h. After being quenched with $Na_2S_2O_3$, the reaction mixture was extracted with CH_2Cl_2 and H_2O, and then dried over $MgSO_4$. After the solvent was removed under vacuum, the residue was purified by silica gel column chromatography with silica gel column chromatography (eluent: PE/DCM = 4/1) to afford **6d** as a yellow solid (58.7 mg, 62.0%). M.P.: > 300 ^oC. ^1H NMR (400 MHz, CD_2Cl_2) δ 9.44 (d, J = 8.0 Hz, 1H), 8.74 (d, J = 7.6 Hz, 1H), 8.49 (d, J = 8.0 Hz, 1H), 8.40 (d, J = 8.4 Hz, 1H), 8.35 (d, J = 8.4 Hz, 1H), 8.00 (d, J = 8.4 Hz, 1H), 7.85 (t, J = 7.6 Hz, 1H), 7.80 (d, J = 8.0 Hz, 1H), 7.77-7.70 (m, 2H), 7.50-7.48 (m, 3H), 7.43 (t, J = 7.4 Hz, 1H), 7.28-7.21 (m, 2H), 7.18 (d, J = 8.8 Hz, 1H), 7.13-7.06 (m, 2H), 6.90 (d, J = 8.0 Hz, 2H), 6.57 (q, J = 7.2 Hz, 1H), 6.45 (d, J = 8.4 Hz, 1H), 6.26 (d, J = 5.2 Hz, 1H); ^{13}C NMR (100 MHz, $CDCl_3$) δ 139.84, 138.04, 136.38, 135.25, 134.94, 134.72, 134.26, 134.19, 131.83 , 131.13, 131.03, 130.93, 130.28, 130.07, 129.87, 129.82, 129.75, 129.30, 128.76, 128.27, 128.09, 127.74, 127.64, 127.55, 127.44, 126.89, 126.85, 126.79, 126.64, 126.37, 126.23, 126.15, 126.08, 125.59, 125.25, 125.03, 123.56, 122.85, 122.29, 121.93; HRMS (MALDI) m/z $[M]^+$ calcd for $[C_{44}H_{24}S]$ 584.1593; found 584.1593; IR (KBr): 3057, 1498, 1441, 1251, 906, 812, 731 cm^{-1}.

Scheme S4. Synthesis of compounds 6e and 6f

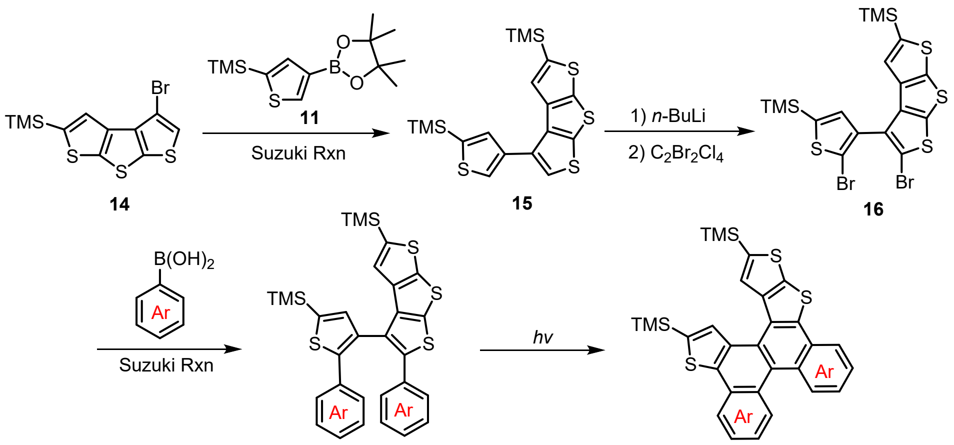

Synthesis of 15

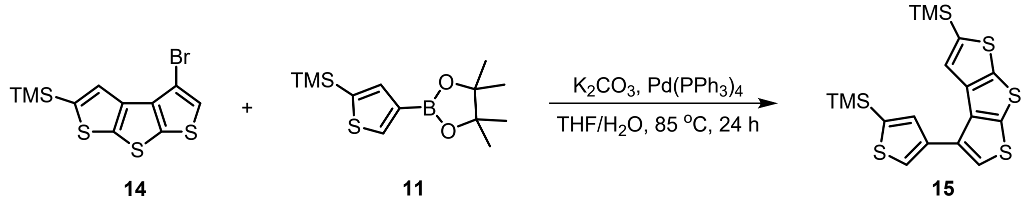

A mixture of **14** (562.1 mg, 1.62 mmol), **11** (730.8 mg, 2.59 mmol), K_2CO_3 (670.9 mg, 4.85 mmol), and $Pd(PPh_3)_4$ (56.1 mg, 0.05 mmol) and deoxidized water (6 mL) were added into THF (60 mL) and stirred under Ar atmosphere for 24 h at 85 °C. The solution was concentrated under reduce pressure. The crude product was extracted with dichloromethane and water, dried over anhydrous $MgSO_4$. After the solvent was removed under vacuum, the residue was purified by silica gel column chromatography with petrol ether (60-90 °C) as the eluent to afford **15** as a yellow solid (473.4 mg, 70.9%). M.P.: 100-101 °C. ^1H NMR (400 MHz, $CDCl_3$) δ 7.71 (s, 1H), 7.53 (s, 1H), 7.37 (s, 1H), 7.28 (s, 1H), 0.39 (s, 9H), 0.34 (s, 9H); ^{13}C NMR (100 MHz, $CDCl_3$) δ 144.65, 144.04, 141.33, 140.41, 140.23, 137.90, 136.38, 135.00, 130.48, 127.70, 125.60, 123.55, 0.15, 0.07; HRMS (MALDI) *m/z* $[M]^+$ calcd for $[C_{18}H_{22}S_4Si_2]$ 422.0137; found 422.0130; IR (KBr): 3089, 2954, 1249, 1006, 837, 752, 626 cm^{-1}.

Synthesis of 16

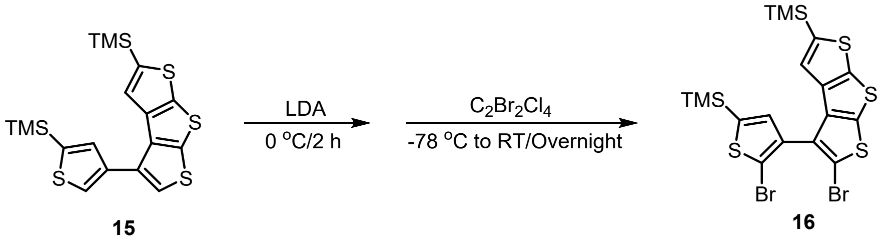

n-BuLi (2.0 M in hexane, 0.71 mL, 0.56 mmol) was added dropwise to diisopropylamine (0.24 mL, 0.68 mmol) in anhydrous Et_2O (15 mL) at 0 °C. After keeping at 0 °C for 0.5 h, the newly prepared LDA solution was transferred by syringe into the solution of **15** (239.0 mg, 0.57 mmol) in anhydrous Et_2O (20 mL) at -78 °C. After keeping at -78 °C for 2 h, $C_2Br_2Cl_4$ (461.0 mL, 1.41 mmol) was added at -78 °C, and then the reaction mixture was warmed up slowly to ambient temperature overnight. The reaction mixture was quenched with CH_3OH at -78 °C, and extracted with CH_2Cl_2 and H_2O, and then dried over $MgSO_4$. After the solvent was removed under vacuum, the residue was purified by silica gel column chromatography with petrol ether (60-90 °C) as the eluent to afford **16** as a white solid (326.2 mg, 99.5%). M.P.: 49-50 °C. ^1H NMR (400 MHz, $CDCl_3$) δ 7.14 (s, 1H), 6.80

(s, 1H), 0.36 (s, 9H), 0.29 (s, 9H); ^{13}C NMR (100 MHz, $CDCl_3$) δ 144.7, 143.8, 142.4, 140.5, 137.0, 136.4, 135.8, 135.5, 128.9, 124.9, 117.3, 112.0, 0.0, -0.1; HRMS (MALDI) *m/z* $[M]^+$ calcd for $[C_{18}H_{20}Br_2S_4Si_2]$ 577.8348; found 577.8335; IR (KBr): 2954, 1400, 1251, 983, 838, 756, 626 cm^{-1}.

Synthesis of 5e

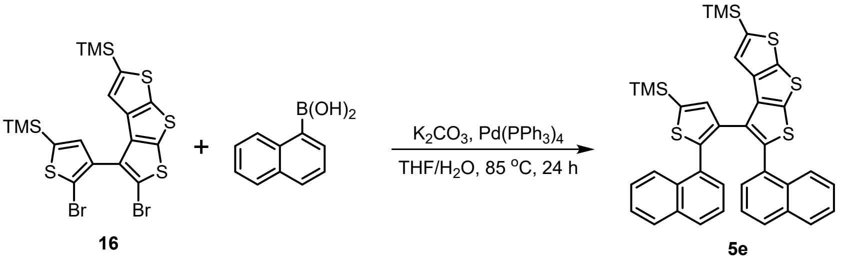

A mixture of **16** (288.0 mg, 0.50 mmol), 1-biphenylboronicacid (213.3 mg, 1.24 mmol), K_2CO_3 (274.2 mg, 1.98 mmol), and $Pd(PPh_3)_4$ (34.4 mg, 0.03 mmol) and deoxidized water (3 mL) were added into THF (30 mL) and stirred under Ar atmosphere for 24 h at 85 °C. The solution was concentrated under reduce pressure. The crude product was extracted with dichloromethane and water, dried over anhydrous $MgSO_4$. After the solvent was removed under vacuum, the residue was purified by silica gel column chromatography with petrol ether (60-90 °C) as the eluent to afford **5e** as a yellow solid (231.3 mg, 69%). M.P.: 199-200 °C. ^{1}H NMR (400 MHz, DMSO-d_6) δ 7.77-7.75 (m, 2H), 7.72 (d, J = 8.0 Hz, 1H), 7.60 (d, J = 7.4 Hz, 2H), 7.39-7.26 (m, 3H), 7.19(m, 1H), 7.11 (t, J = 7.6 Hz, 1H), 7.05 (d, J = 6.6 Hz, 1H), 6.97-6.95 (m, 2H), 6.83 (t, J = 7.7 Hz, 1H), 6.45(m, 1H), 6.34 (d, J = 4.0 Hz, 1H), 0.38 (s, 9H), 0.34 (s, 9H); ^{13}C NMR (100 MHz, $CDCl_3$) δ 144.54, 144.27, 143.95, 141.39, 140.13, 139.93, 138.53, 138.06, 136.97, 134.51, 133.47, 131.44, 131.34, 131.12, 128.91, 128.35, 128.25, 127.90, 127.76, 127.72, 127.66, 125.73, 125.53, 125.50, 125.43, 125.32, 124.90, 124.77, 0.22, 0.18; HRMS (MALDI) *m/z* $[M]^+$ calcd for $[C_{38}H_{34}S_4Si_2]$ 674.1076; found 674.1062; IR (KBr): 3043, 2950, 1406, 1249, 999, 839, 773 cm^{-1}.

Synthesis of 5f

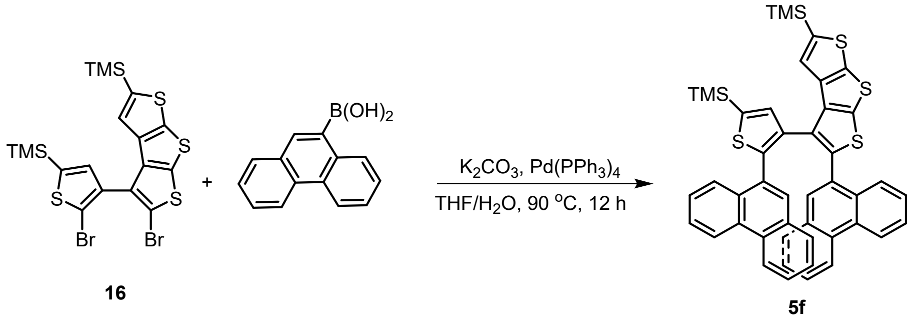

A mixture of **16** (610.0 mg, 1.05 mmol), 9-Phenanthraceylboronic acid (513.4 mg, 2.31 mmol),

K_2CO_3 (581.0 mg, 4.24 mmol), and $Pd(PPh_3)_4$ (121.4 mg, 0.105 mmol) and deoxidized water (6 mL) were added into THF (60 mL) and stirred under Ar atmosphere for 12 h at 90 °C. The solution was concentrated under reduce pressure. The crude product was extracted with dichloromethane and water, dried over anhydrous $MgSO_4$. After the solvent was removed under vacuum, the residue was purified by silica gel column chromatography (eluent: PE/DCM = 10/1) to afford **5f** as a yellow solid (416.8 mg, 51%). M.P.: 189-190 °C. ^{1}H NMR (400 MHz, DMSO-d_6) δ 8.63-8.56 (m, 4H), 8.31 (s, 1H), 7.83 (s, 1H), 7.62-7.54 (m, 2H), 7.41-7.19 (m, 8H), 6.93-6.45 (m, 4H), 0.40 (s, 9H), 0.37 (s, 9H); ^{13}C NMR (100 MHz, $CDCl_3$) δ 144.60, 144.34, 144.10, 141.44, 140.20, 139.94, 137.92, 137.03, 134.79, 131.08, 130.94, 130.26, 130.17, 130.08, 129.94, 129.79, 129.19, 128.57, 128.08, 126.64, 126.45, 126.30, 126.26, 126.02, 125.90, 125.64, 122.08, 121.96, 0.24, 0.23; HRMS (MALDI) *m/z* $[M]^+$ calcd for $[C_{46}H_{38}S_4Si_2]$ 774.1389; found 774.1376; IR (KBr): 2951, 1628, 1446, 1250, 1003, 837 cm^{-1}.

Synthesis of 6e

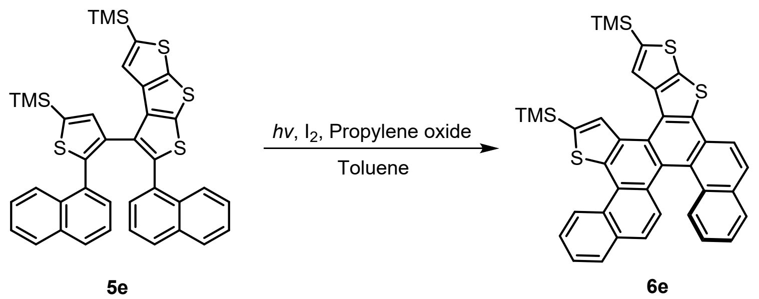

A mixture of **5e** (40.0 mg, 0.06 mmol), I_2 (60.1 mg,0.24 mmol), propylene oxide (0.14 mL) was added into anhydrous toluene (20 mL) under argon. The reaction mixture was irradiation with a high-pressure Hg lamp for 4 h. After being quenched with $Na_2S_2O_3$, the reaction mixture was extracted with CH_2Cl_2 and H_2O, and then dried over $MgSO_4$. After the solvent was removed under vacuum, the residue was purified by silica gel column chromatography with petrol ether (60-90 °C) as the eluent to afford **6e** as a yellow solid (12.1 mg, 31.8%). M.P.: 225-226 °C. ^{1}H NMR (500 MHz, $CDCl_3$) δ 9.57 (d, J = 8.5 Hz, 1H), 8.61 (s, 1H), 8.19-8.16 (m, 3H), 8.04-8.03 (m, 2H), 7.98-7.96 (m, 2H), 7.89 (t, J = 7.8 Hz, 1H), 7.70 (t, J = 7.3 Hz, 1H), 7.58 (d, J = 9.0 Hz, 1H), 7.48 (t, J = 7.3 Hz, 1H), 7.20 (t, J = 7.5 Hz, 1H), 0.55 (s, 9H), 0.41 (s, 9H); ^{13}C NMR (125 MHz, $CDCl_3$) δ 146.19, 144.04, 143.14, 143.10, 139.48, 138.26, 136.28, 133.43, 132.61, 132.52, 131.12, 130.04, 129.75, 129.48, 129.30, 129.28, 128.92, 128.51, 128.29, 127.43, 127.11, 126.44, 126.31, 126.09, 125.83, 125.59,

125.54, 125.43, 124.19, 123.75, 122.16, 0.30, 0.17; HRMS (MALDI) *m/z* $[M]^+$ calcd for $[C_{38}H_{32}S_3Si_2]$ 640.1199; found 640.1183; IR (KBr): 3055, 2958, 1248, 999, 910, 837, 740 cm^{-1}.

Synthesis of 6f

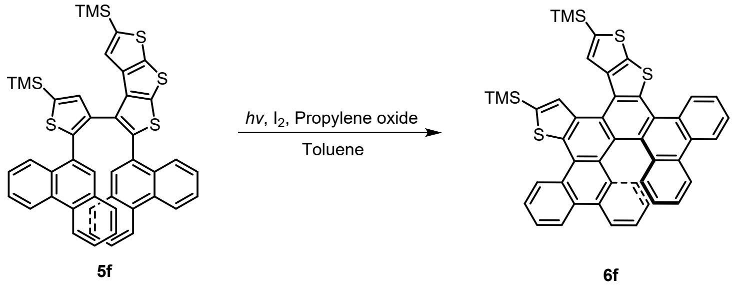

A mixture of **5f** (40.0 mg, 0.05 mmol), I_2 (52.4 mg,0.21 mmol), propylene oxide (0.14 mL) was added into anhydrous toluene (20 mL) under argon. The reaction mixture was irradiation with a high-pressure Hg lamp for 5 h. After being quenched with $Na_2S_2O_3$, the reaction mixture was extracted with CH_2Cl_2 and H_2O, and then dried over $MgSO_4$. After the solvent was removed under vacuum, the residue was purified by silica gel column chromatography (eluent: PE/DCM = 7/1) to afford **6f** as a yellow solid (18.4 mg, 48.2%). M.P.: > 300 °C. ^1H NMR (400 MHz, $CDCl_3$) δ 9.35 (d, J = 8.0 Hz, 1H), 9.16 (d, J = 8.0 Hz, 1H), 8.75 (d, J = 8.0 Hz, 2H), 8.63 (s, 1H), 8.39 (t, J = 7.2 Hz, 1H), 8.10 (s, 1H), 7.95-7.88 (m, 2H), 7.85-7.81 (m, 2H), 7.38 (t, J = 7.4 Hz, 2H), 7.20 (t, J = 7.4 Hz, 2H), 6.71-6.64 (m, 2H), 0.51 (s, 9H), 0.44 (s, 9H); ^{13}C NMR (100 MHz, $CDCl_3$) δ 146.15, 144.79, 143.56, 141.74, 141.24, 140.83, 135.71, 134.14, 131.47, 131.40, 131.04, 130.97, 129.78, 129.69, 129.45, 129.26, 129.21, 128.83, 128.58, 127.92, 127.85, 127.68, 127.60, 127.44, 127.36, 126.89, 126.42, 126.19, 126.12, 125.94, 125.72, 124.82, 123.79, 123.59, 122.89, 122.85, 121.84, 0.25; HRMS (MALDI) *m/z* $[M]^+$ calcd for $[C_{46}H_{36}S_3Si_2]$ 740.1512; found 740.1495; IR (KBr): 3060, 2956, 1433, 1247, 997, 835, 756, 746 cm^{-1}.

Scheme S5. Synthesis of compounds 8b and 8c

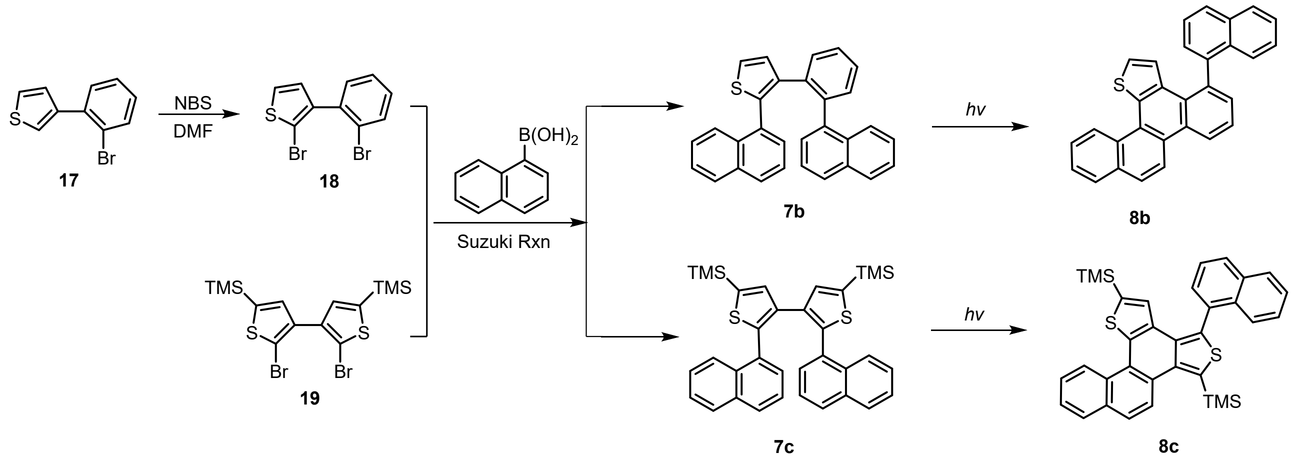

Synthesis of 18

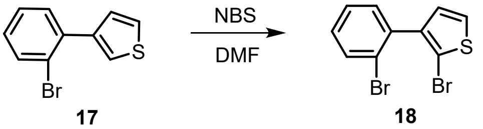

Compound **17** (205.0 mg, 0.857 mmol, 1.0 eq.) was stirred and mixed well with DMF in an ice-water bath. NBS (175.5 mg, 0.986 mmol, 1.15 eq.) was dissolved in DMF (2 mL) and slowly added dropwise to the reaction mixture. The reaction was carried out at 0 °C for 14 h. After being quenched with $Na_2S_2O_3$, the reaction mixture was extracted with EA and H_2O, and then dried over $MgSO_4$. After the solvent was removed under vacuum, the residue was purified by silica gel column chromatography with petrol ether (60-90 °C) as the eluent to afford **18** as a yellowish oil (151.0 mg, 55%). ^{1}H NMR (400 MHz, $CDCl_3$) δ 7.68 (d, J = 8.4 Hz, 1H), 7.39-7.36 (m, 1H), 7.34 (d, J = 2.0 Hz, 1H), 7.31 (d, J = 5.6 Hz, 1H), 7.27-7.23 (m, 1H), 6.98 (d, J = 5.6 Hz, 1H); ^{13}C NMR (100 MHz, $CDCl_3$) δ 141.3, 136.4, 133.1, 131.9, 129.7, 129.5, 127.3, 125.5, 123.7, 111.4; HRMS (EI) *m/z* $[M]^+$ calcd for $[C_{10}H_6Br_2S]$ 315.8551; found 315.8548; IR (KBr): 2927, 1730, 1467, 1028, 871, 752, 638 cm^{-1}.

Synthesis of 7b

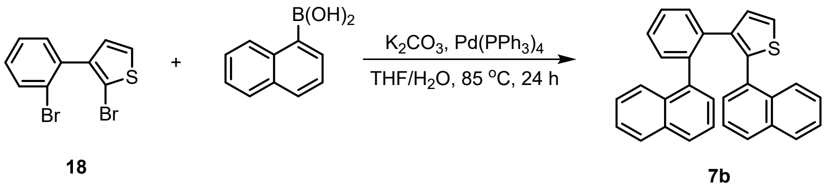

A mixture of **18** (356.0 mg, 1.12 mmol), 1-biphenylboronicacid (404.3 mg, 2.35 mmol), K_2CO_3 (618.8 mg, 4.48 mmol), and $Pd(PPh_3)_4$ (129.3 mg, 0.11 mmol) and deoxidized water (5 mL) were added into THF (40 mL) and stirred under Ar atmosphere for 24 h at 85 °C. The solution was concentrated under reduce pressure. The crude product was extracted with dichloromethane and water, dried over anhydrous $MgSO_4$. After the solvent was removed under vacuum, the residue was purified by silica gel column chromatography with petrol ether (60-90 °C) as the eluent to afford **7b** as a yellow solid (350.1 mg, 76%). M.P.: 177-179 °C. ^1H NMR (400 MHz, DMSO-d_6) δ 7.79 (d, J = 8.2 Hz, 1H), 7.74 (t, J = 8.3 Hz, 2H), 7.59-7.55 (m, 2H), 7.48-7.44 (m, 3H), 7.39-7.33 (m, 4H), 7.28-7.22 (m, 2H), 7.14 (d, J = 5.2 Hz, 1H), 7.12-7.03 (m, 4H), 6.84 (d, J = 5.2 Hz, 1H). ^{13}C NMR (100 MHz, $CDCl_3$) δ 141.08, 140.31, 140.06, 138.17, 135.54, 134.10, 134.00, 133.38, 132.42, 132.21, 130.88, 130.44, 129.79, 129.49, 129.34, 128.81, 128.48, 127.92, 127.46, 127.44, 126.35, 126.18, 125.60, 125.57, 124.37. HRMS (MALDI): m/z $[M]^+$ calcd for $[C_{30}H_{20}S_2]$ 444.10061, found 444.10009. IR (KBr): 3049, 1513, 1387, 844, 764, 708, 686 cm^{-1}.

Synthesis of 7c

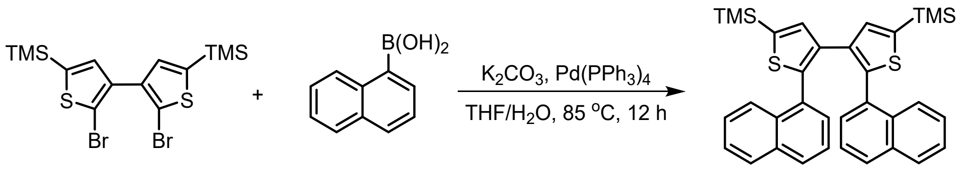

19 7c

A mixture of **19** (1.0000 g, 2.14 mmol), 1-biphenylboronicacid (771.1 mg, 4.48 mmol), K_2CO_3 (1.18 mg, 8.54 mmol), and $Pd(PPh_3)_4$ (148.0 mg, 0.13 mmol) and deoxidized water (8 mL) were added into THF (100 mL) and stirred under Ar atmosphere for 12 h at 85 °C. The solution was concentrated under reduce pressure. The crude product was extracted with dichloromethane and water, dried over anhydrous $MgSO_4$. After the solvent was removed under vacuum, the residue was purified by silica gel column chromatography with silica gel column chromatography with petrol ether (60-90 °C) as the eluent to afford **7c** as a yellow solid (865.0 mg, 72%). M.P.: 199-200 ^{o}C. ^1H NMR (400 MHz, $CDCl_3$) δ 7.71 (d, J = 8.0 Hz, 2H), 7.59 (d, J = 8.0 Hz, 2H), 7.48 (d, J = 8.4 Hz, 2H), 7.36 (t, J = 7.0 Hz, 2H), 7.21 (t, J = 7.0 Hz, 2H), 7.15 (t, J = 7.6 Hz, 2H), 6.98 (s, 2H), 6.95 (d, J = 6.4 Hz, 2H), 0.17 (s, 18H); ^{13}C NMR (100 MHz, $CDCl_3$) δ 142.41, 139.23, 136.40, 136.27, 133.65, 132.39, 131.79, 128.61, 128.07, 127.91, 126.22, 125.64, 125.59, 125.10, 0.10; HRMS (MALDI) *m/z*

[M]$^+$ calcd for [$C_{34}H_{34}S_2Si_2$] 562.1635; found 562.1627; IR (KBr): 3046, 2952, 2894, 1390, 1253, 1001, 846 cm^{-1}.

Synthesis of 8b

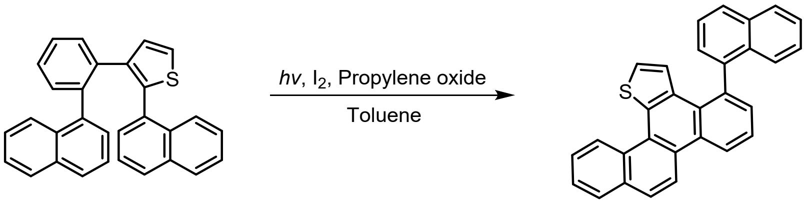

7b **8b**

A mixture of **7b** (40.0 mg, 0.10 mmol), I_2 (73.8 mg, 0.291) mmol, propylene oxide (0.14 mL) was added into anhydrous toluene (20 mL) under argon. The reaction mixture was irradiation with a high-pressure Hg lamp for 4.5 h. After being quenched with $Na_2S_2O_3$, the reaction mixture was extracted with CH_2Cl_2 and H_2O, and then dried over $MgSO_4$. After the solvent was removed under vacuum, the residue was purified by silica gel column chromatography with petrol ether (60-90 °C) as the eluent and subsequently precipitated from a mixture of CH_2Cl_2/CH_3OH to afford **8b** as a grayish-white solid 26.1 mg, 65.6%). M.P.: 239-240 °C. ^{1}H NMR (400 MHz, $CDCl_3$) δ 9.42 (d, J = 8.8 Hz, 1H), 9.01 (d, J = 8.4 Hz, 1H), 8.90 (d, J = 9.2 Hz, 1H), 8.06 (q, J = 8.0 Hz, 3H), 7.98 (d, J = 8.0 Hz, 1H), 7.83-7.78 (m, 2H), 7.70 (t, J = 7.4 Hz, 1H), 7.67-7.63 (m, 2H), 7.57 (d, J = 6.8 Hz, 1H), 7.50-7.47 (m, 2H), 7.25 (t, J = 7.6 Hz, 1H), 7.09 (d, J = 6.0 Hz, 1H), 6.48 (d, J = 6.0 Hz, 1H); ^{13}C NMR (100 MHz, $CDCl_3$) δ 137.51, 135.95, 135.43, 133.93, 133.12, 132.58, 131.07, 130.40, 129.74, 128.96, 128.48, 128.32, 128.18, 128.01, 127.35, 127.32, 127.13, 126.49, 126.47, 126.41, 126.36, 126.27, 126.05, 125.77, 125.47, 124.15, 124.08, 122.35; HRMS (MALDI) *m/z* [M]$^+$ calcd for [$C_{30}H_{18}S$] 410.1124; found 410.1119; IR (KBr): 3051, 1591, 1506, 1380, 1284, 781, 721 cm^{-1}.

Synthesis of 8c

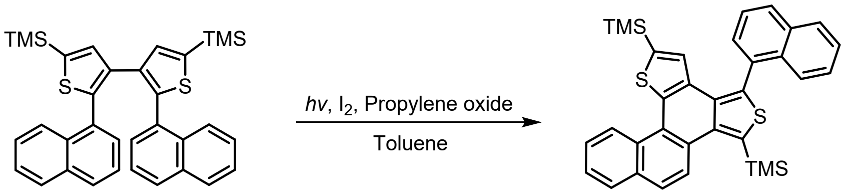

7c **8c**

A mixture of **7c** (100 mg, 0.18mmol), I_2 (180.3 mg,0.71mmol), propylene oxide (0.14 mL) was added into anhydrous toluene (50mL) under argon. The reaction mixture was irradiation with a high-pressure Hg lamp for 2h. After being quenched with $Na_2S_2O_3$, the reaction mixture was extracted with CH_2Cl_2 and H_2O, and then dried over $MgSO_4$. After the solvent was removed under vacuum,

the residue was purified by silica gel column chromatography with petrol ether (60-90 °C) as the eluent and subsequently precipitated from a mixture of CH_2Cl_2/CH_3OH to afford **8c** as a grayish-white solid (61.3 mg, 61%). M.P.: 299-300 °C. ^{1}H NMR (400 MHz, $CDCl_3$) δ 9.14 (d, J = 8.4 Hz, 1H), 8.01-7.97 (m, 2H), 7.91-7.84 (m, 2H), 7.67-7.63 (m, 2H), 7.53-7.47 (m, 2H), 7.45 (d, J = 6.8 Hz, 1H), 7.35 (d, J = 8.4 Hz, 1H), 7.28 (d, J = 8.0 Hz, 1H), 7.22 (d, J = 9.2 Hz, 1H), 6.19 (s, 1H), 0.54 (s, 9H), -0.03 (s, 9H); ^{13}C NMR (100 MHz, $CDCl_3$) δ 139.0, 136.5, 136.2, 133.9, 133.4, 132.5, 132.4, 130.3, 129.6, 129.2, 128.9, 128.4, 128.2, 127.3, 127.2, 126.6, 126.5, 126.2, 126.1, 126.0, 125.7, 125.6, 124.2, -0.84, -1.92; HRMS (MALDI) *m/z* [M]$^+$ calcd for [$C_{34}H_{32}S_2Si_2$] 560.1478; found 560.1483; IR (KBr): 2960, 1446, 1253, 1036, 922, 843, 750 cm^{-1}.

Scheme S6. Synthesis of compounds 8d and 8e

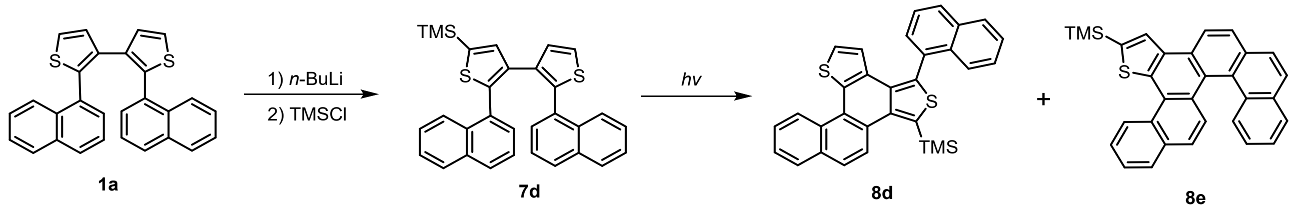

Synthesis of 7d

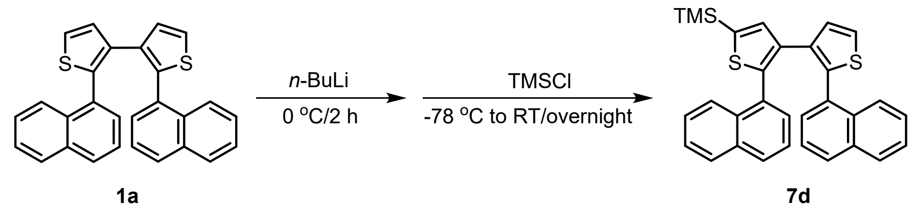

n-BuLi (2.4 M in hexane, 0.55 mL, 1.31 mmol) was added dropwise to a solution of **1a** (500.0 mg, 1.19 mmol) in anhydrous THF (10 mL) at -78 °C. After the mixture was stirred at -78 °C for 2 h, TMSCl (0.2 mL, 1.55 mmol) was slowly added at -78 °C, and then the reaction mixture was warmed up to ambient temperature overnight. The reaction mixture was quenched with CH_3OH at -78 °C, and extracted with CH_2Cl_2 and H_2O, and then dried over $MgSO_4$. After the solvent was removed under vacuum, the residue was purified by silica gel column chromatography with petrol ether (60-90 °C) as the eluent to afford **7d** as a white solid (393.2 mg, 67%). M.P.: 70-71 °C. ^{1}H NMR (400 MHz, $CDCl_3$) δ 7.72 (d, J = 8.4 Hz, 2H), 7.59 (d, J = 8.4 Hz, 2H), 7.50-7.47 (m, 2H), 7.37 (t, J = 7.0 Hz, 2H), 7.23 (t, J = 10.4 Hz, 2H), 7.19 (d, J = 5.2 Hz, 1H), 7.15 (t, J = 7.6 Hz, 2H), 6.99 (s,

1H), 6.97-6.93 (m, 3H), 0.16 (s, 9H); ^{13}C NMR (100 MHz, $CDCl_3$) δ 142.54, 139.40, 137.18, 136.53, 136.13, 134.97, 133.66, 133.64, 132.23, 132.08, 131.97, 131.84, 129.37, 128.89, 128.60, 128.23, 128.14, 127.99, 127.96, 126.11, 126.08, 125.80, 125.73, 125.65, 125.64, 125.14, 125.10, 124.64, 0.13; HRMS (DART) *m/z* $[M+H]^+$ calcd for $[C_{31}H_{27}S_2Si]$ 491.1318; found 491.1322; IR (KBr): 3053, 2956, 1382, 1251, 1005, 841, 772 cm^{-1}.

Synthesis of 8d and 8e

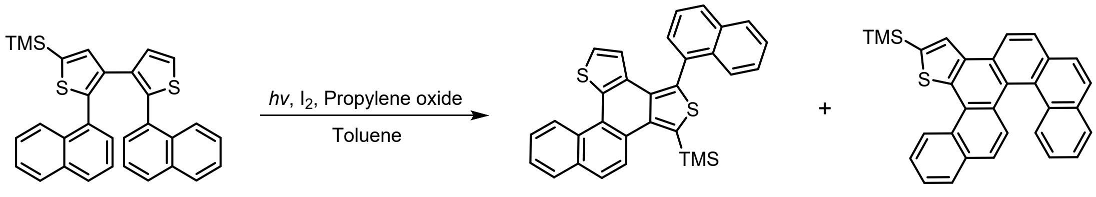

A mixture of **7d** (40.0 mg, 0.08 mmol), I_2 (82.7 mg,0.33mmol), propylene oxide (0.14 mL) was added into anhydrous toluene (20 mL) under argon. The reaction mixture was irradiation with a high-pressure Hg lamp for 1h. After being quenched with $Na_2S_2O_3$, the reaction mixture was extracted with CH_2Cl_2 and H_2O, and then dried over $MgSO_4$. After the solvent was removed under vacuum, the residue was purified by silica gel column chromatography with petrol ether (60-90 °C) as the eluent to afford **8d** as a grayish-white solid (8.2 mg, 41.2%) and **8e** as a grayish-white solid (2.1 mg, 11.3%).

Compound **8d**: M.P.: 292-293 °C. ^{1}H NMR (400 MHz, $CDCl_3$) δ 9.14 (d, J = 8.8 Hz, 1H), 8.00 (t, J = 10.0 Hz, 2H), 7.90 (d, J = 8.0 Hz, 1H), 7.86 (d, J = 7.6 Hz, 1H), 7.65 (t, J = 7.4 Hz, 2H), 7.52 (d, J = 8.8 Hz, 1H), 7.49 (d, J = 7.2 Hz, 1H), 7.44 (d, J = 6.4 Hz, 1H), 7.35 (d, J = 8.4 Hz, 1H), 7.29 (d, J = 7.2 Hz, 1H), 7.16 (d, J = 9.2 Hz, 1H), 6.19 (d, J = 10.0 Hz, 1H), 6.06 (d, J = 10.0 Hz, 1H), 0.53 (s, 9H); ^{13}C NMR (100 MHz, $CDCl_3$) δ 139.04, 136.19, 135.41, 133.95, 133.32, 132.47, 132.37, 131.20, 130.20, 129.56, 129.14, 129.10, 128.98, 128.43, 128.29, 127.55, 127.26, 126.63, 126.47, 126.32, 126.29, 126.18, 125.94, 125.91, 125.82, 125.07, 121.45, 119.86, -0.92; HRMS (DART) *m/z* $[M+H]^+$ calcd for $[C_{31}H_{25}S_2Si]$ 489.1161; found 489.1160; IR (KBr): 3045, 1768, 1707, 1587, 1535, 1251, 841 cm^{-1}.

Compound **8e**: M.P.: 180-181 °C. ^{1}H NMR (500 MHz, $CDCl_3$) δ 9.39 (d, J = 8.5 Hz, 1H), 8.56 (d, J = 8.5 Hz, 1H), 8.38 (s, 1H), 8.24-8.21 (m, 2H), 8.05 (d, J = 8.5 Hz, 1H), 7.99-7.96 (m, 4H), 7.90 (t, J = 7.75 Hz, 1H), 7.71 (t, J = 7.5 Hz, 1H), 7.52-7.50 (m, 2H), 7.22 (t, J = 7.75 Hz, 1H), 0.55 (s, 9H); ^{13}C NMR (100 MHz, $CDCl_3$) δ 137.68, 132.92, 132.75, 131.77, 131.17, 130.06, 129.77, 129.60,

129.35, 128.94, 128.57, 128.03, 127.96, 127.55, 127.51, 126.93, 126.83, 126.48, 126.45, 126.33, 125.93, 125.80, 125.00, 124.02, 122.88, 0.15; HRMS (DART) *m/z* $[M+H]^+$ calcd for $[C_{31}H_{25}SSi]$ 457.1411; found 457.1440; IR (KBr): 3086, 2887, 1716, 1579, 1234, 1128, 835 cm^{-1}.

3. Crystal Structure and Crystal Data

X-ray Single crystal diffraction for Compounds **2a**, **2b**, **2d**, **4g**, **6e**, **6f** and **8b** data were performed on a diffractometer with CCD detector using Mo Kα radiation (λ= 0.71073 Å) or Cu Kα radiation (λ= 1.54184 Å) source. Their structures were solved by direct methods using SHELXTL and refined with full-matrix least-squares calculations on F^2 using SHELXL-97. All non-hydrogen atoms were refined anisotropically. All hydrogen atoms were positioned by geometric idealization. Additional crystal data and refinement information were summarized in Table S1 and S2.

Table S1. Crystal data and structure refinement for **2a**, **2b**, **2d** and **4g**.

compound	**2a**	**2b**	**2d**	**4g**
CCDC	2479166	2479167	2479168	2479169
Formula	$C_{28}H_{16}S$	$C_{28}H_{16}S$	$C_{36}H_{22}S_2$	$C_{25}H_{16}S$
fw	384.47	384.47	518.65	348.44
Temp (K)	293(2)	295.1(3)	293(2) K	297.11
Wavelength (Å)	1.54184	1.54184	0.71073	0.71073
crystal system	orthorhombic	monoclinic	monoclinic	orthorhombic
space group	Pbca	P21	*P* 1 21/n 1	Aea2
a (Å)	18.3772(6)	13.7281(2)	10.3233(7)	15.3336(16)
b (Å)	8.0425(3)	8.24130(10)	14.2924(10)	28.904(3)
c (Å)	24.7367(8)	16.4447(2)	17.4218(12)	7.6135(8)
α (deg)	90	90	90	90
β (deg)	90	91.3250(10)	95.611(3)	90
γ (deg)	90	90	90	90
V (Å^3)	3656.1(2)	1860.01(4)	2558.2(3)	3374.3(6)
Z	8	4	4	8
D_c (g/cm^3)	1.397	1.373	1.347	1.372
μ (mm^{-1})	1.641	1.612	0.233	0.197
F (000)	1600.0	800.0	1080	1456.0
crystal size (mm)	0.14×0.1×0.09	0.11×0.12×0.14	0.29×0.18×0.15	0.21×0.15×0.04

reflns collected	8481	13572	23381	23706
unique reflns (R_{int})	3451(0.0321)	5535(0.0312)	4696(0.0407)	3769(0.1127)
θ range (deg)	7.148-141.854	5.376-151.466	1.847-25.398	5.314-56.55
data/restraints/params	3451/0/262	5535/1/523	4696/0/343	3769/1/238
final R indices	0.0541/0.1518	0.0348/0.0960	0.0352/0.0930	0.0574/0.1056
R indices (all data)	0.0669/0.1619	0.0360/0.0971	0.0402/0.0956	0.1178/0.1333
GOF on F^2	1.092	1.072	1.05	1.013
ρ_{max}/ρ_{min}(e·Å^{-3})	0.24/-0.35	0.15/-0.30	0.177/-0.211	0.20/-0.22

Table S2. Crystal data and structure refinement for **6e, 6f** and **8b.**

compound	**6e**	**6f**	**8b**
CCDC	2479172	2479170	2479276
Formula	$2C_{38}H_{32}S_3Si_2(2CHCl_3)$	$C_{46}H_{36}S_3Si_2$	$C_{30}H_{18}S$
fw	1520.72	741.11	410.50
Temp (K)	150	150	150
Wavelength (Å)	0.71073	0.71073	0.71073
crystal system	monoclinic	orthorhombic	monoclinic
space group	P 1 21/c 1	P c a 21	P 1 21/c 1
a (Å)	37.2215(17)	15.9945(10)	6.5519(2)
b (Å)	16.0807(7)	11.1752(7)	11.8227(6)
c (Å)	13.1675(5)	21.4452(10)	25.9099(13)
α (deg)	90	90	90
β (deg)	81.889(2)	90	97.126(2)
γ (deg)	90	90	90
V (Å^3)	7802.5(6)	3833.2(4)	1991.51(15)
Z	4	4	4
D_c (g/cm^3)	1.295	1.284	1.369
μ (mm^{-1})	0.484	0.289	0.178
F (000)	3152	1552	856
crystal size (mm)	0.21×0.19×0.09	0.14×0.13×0.12	0.25×0.16×0.12
reflns collected	62127	34366	21631
unique reflns (R_{int})	19376(0.1221)	9150(0.1299)	4944(0.054)

θ range (deg)	2.011-28.362	2.223-28.308	2.34-28.327
data/restraints/params	19376 / 192 / 952	9150/1/466	4944/0/280
final R indices	0.0850/0.2002	0.0558/0.0869	0.0420/0.0949
R indices (all data)	0.1729/0.2494	0.1223/0.1072	0.0670/0.1063
GOF on F^2	1.028	1.003	1.034
ρ_{max}/ρ_{min}(e·Å^{-3})	0.568/-0.884	0.243/-0.270	0.266/-0.304

4. ^{1}H NMR Studies for 2a, 8b, 8c, 8d, 8e and 2a'

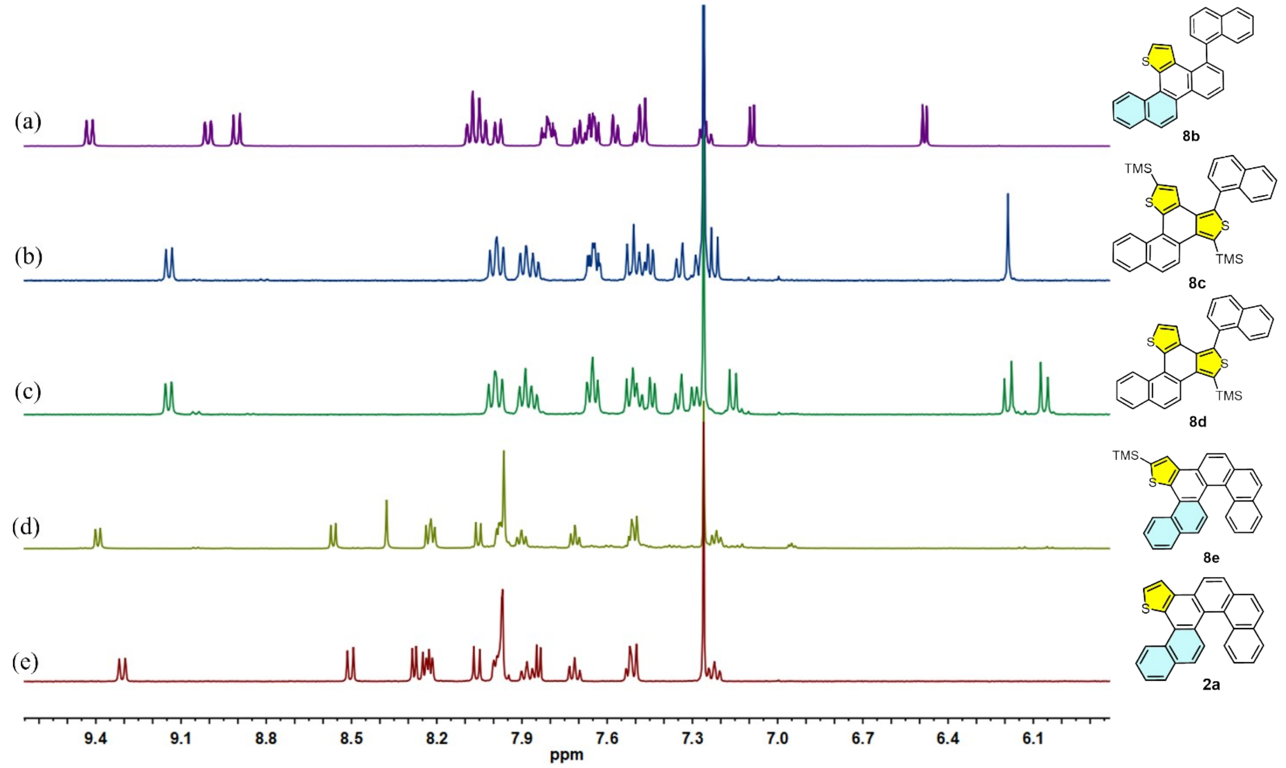

Figure S1. Partial ^{1}H NMR spectra of compounds **8b** (a), **8c** (b), **8d** (c), **8e** (d) and **2a** (e) in $CDCl_3$ (400 MHz, 298 K).

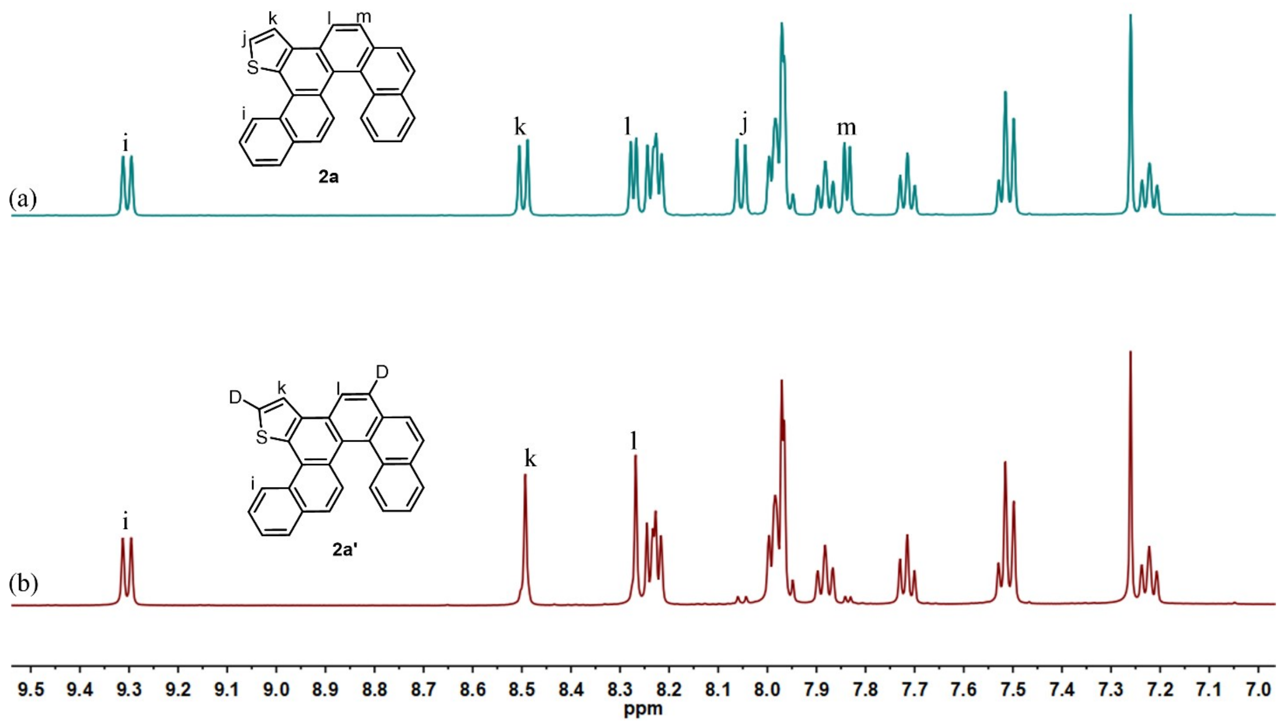

Figure S2. Partial ^{1}H NMR spectra of compounds **2a** (a) and **2a'** (b) in $CDCl_3$ (400 MHz, 298 K).

5. ^{1}H-^{1}H NOESY Spectra for 4g, 2a and 2a'

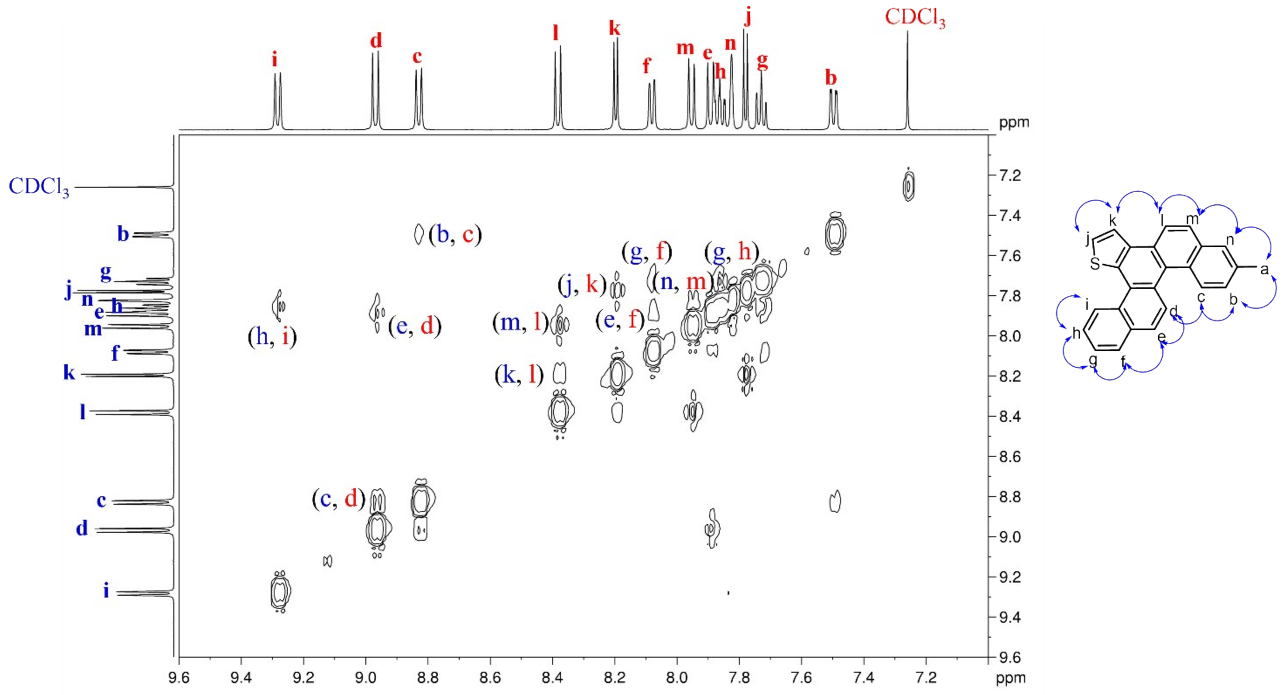

Figure S3. Partial ^{1}H-^{1}H NOESY spectra (500 MHz, 298 K) of **4g** in $CDCl_3$.

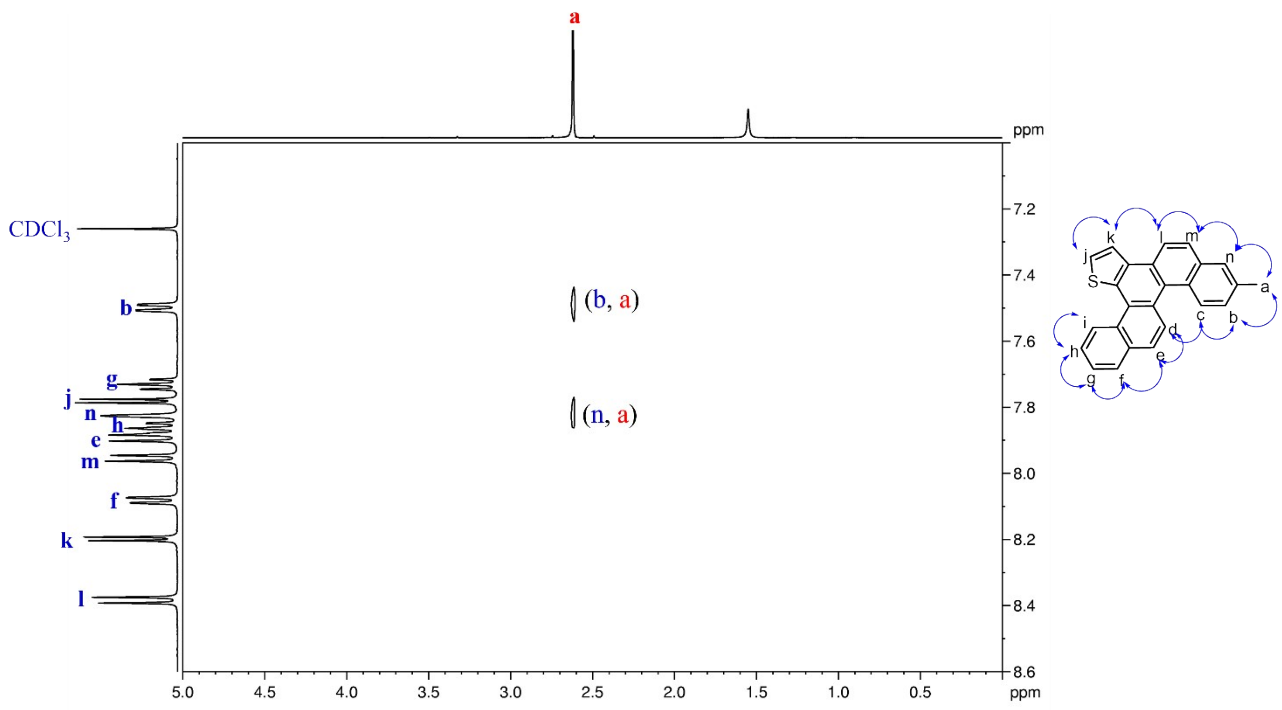

Figure S4. ^1H-^1H NOESY spectra (500 MHz, 298 K) of **4g** in $CDCl_3$.

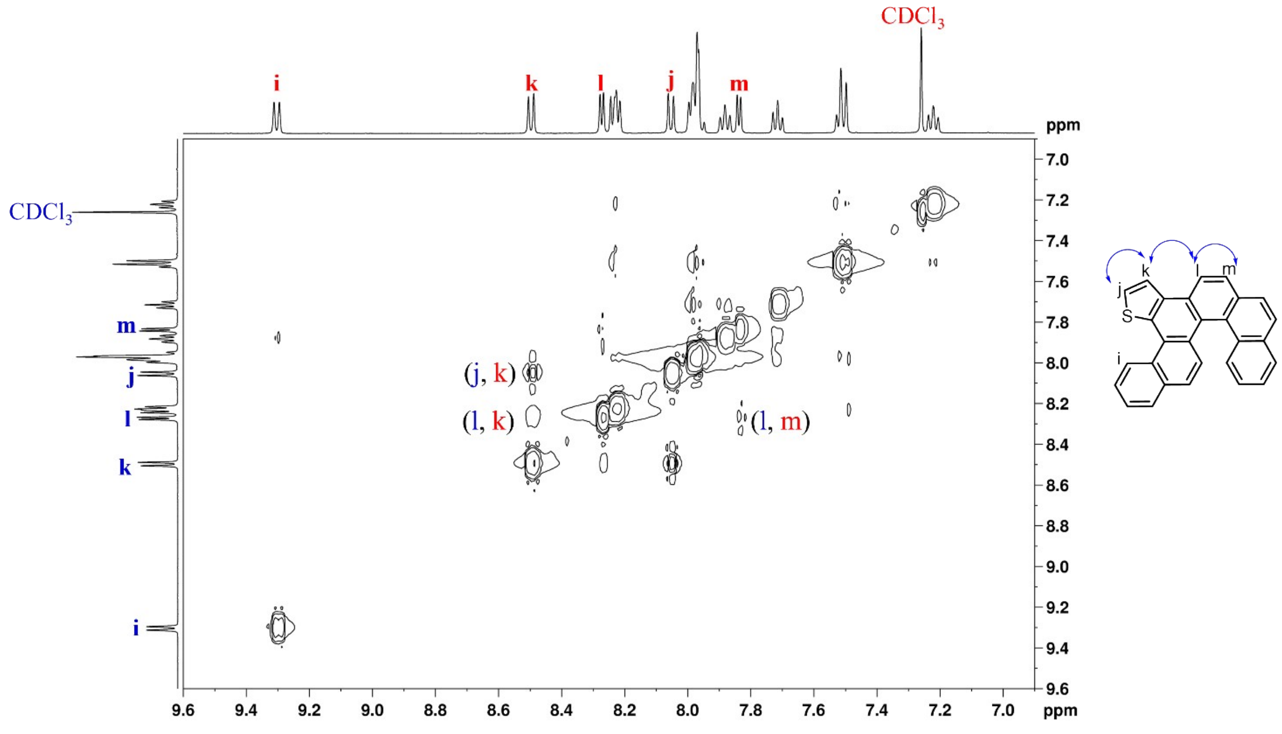

Figure S5. Partial ^1H-^1H NOESY spectra (500 MHz, 298 K) of **2a** in $CDCl_3$.

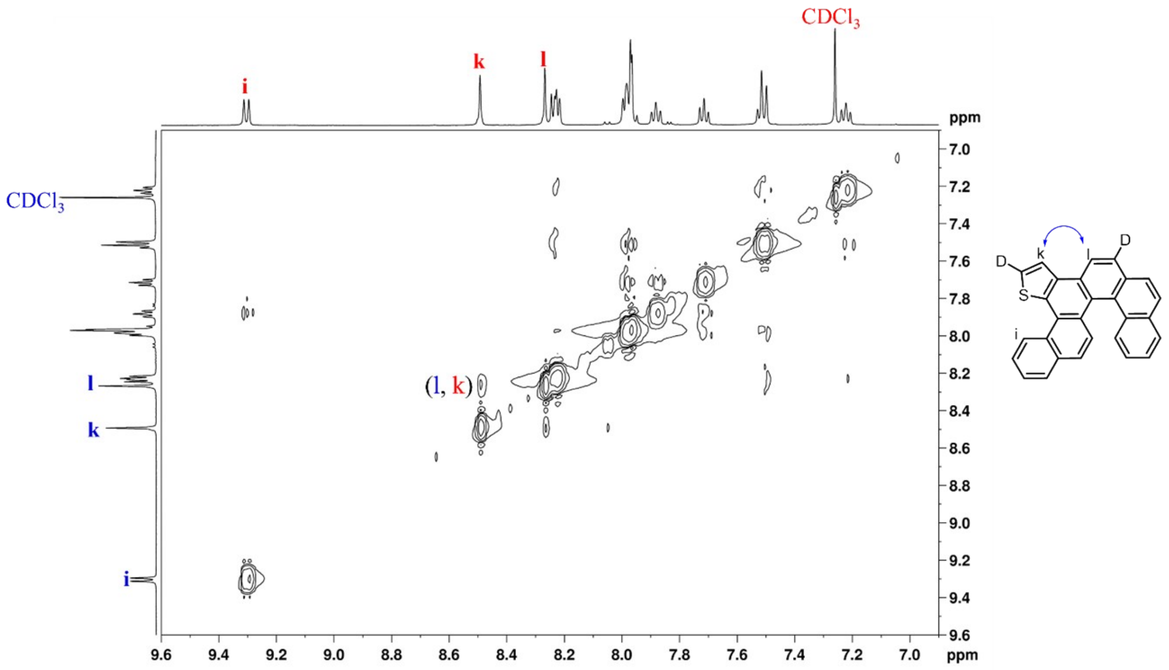

Figure S6. Partial ^{1}H-^{1}H NOESY spectra (500 MHz, 298 K) of **2a'** in $CDCl_3$.

6. DFT calculation and proposed mechanism

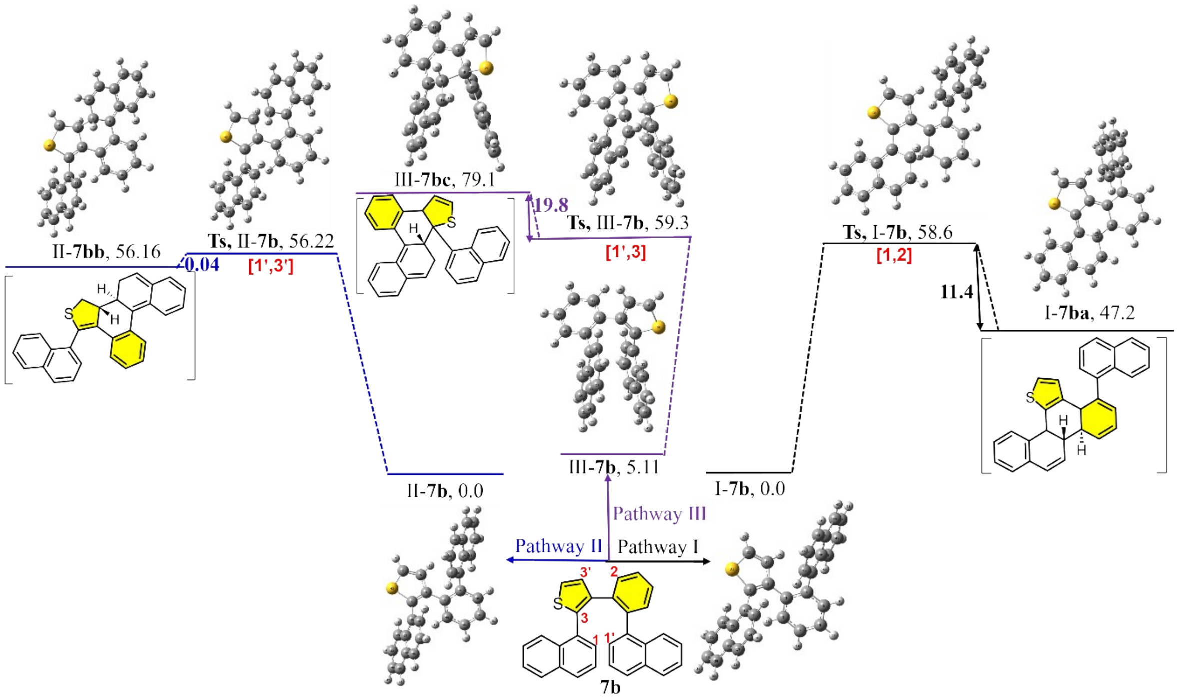

Figure S7. Computed three photocyclization pathways I, II and III. Precursor **7b** and intermediates **7ba**, **7bb** and **7bc** of the photocyclization reaction were optimized at the ωB97XD/6-31G(d) level of theory. The corresponding transition states were located by broken-symmetry calculations at the UωB97XD/6-31G(d) level with the Guess=Mix keyword. All relative electronic energies are reported in kcal/mol.

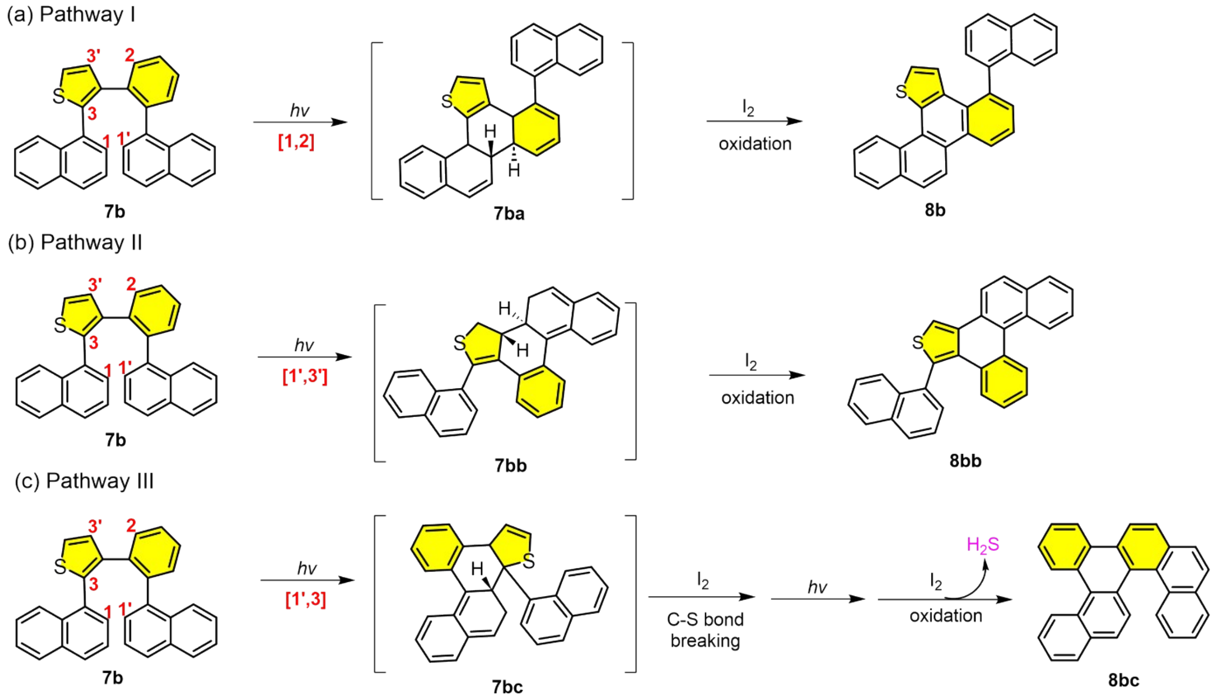

Figure S8. Proposed mechanism for the traditional photocyclization pathway I (a) and II (b) and photochemical cyclization and desulfurization pathway III (c) of precursor **7b**.

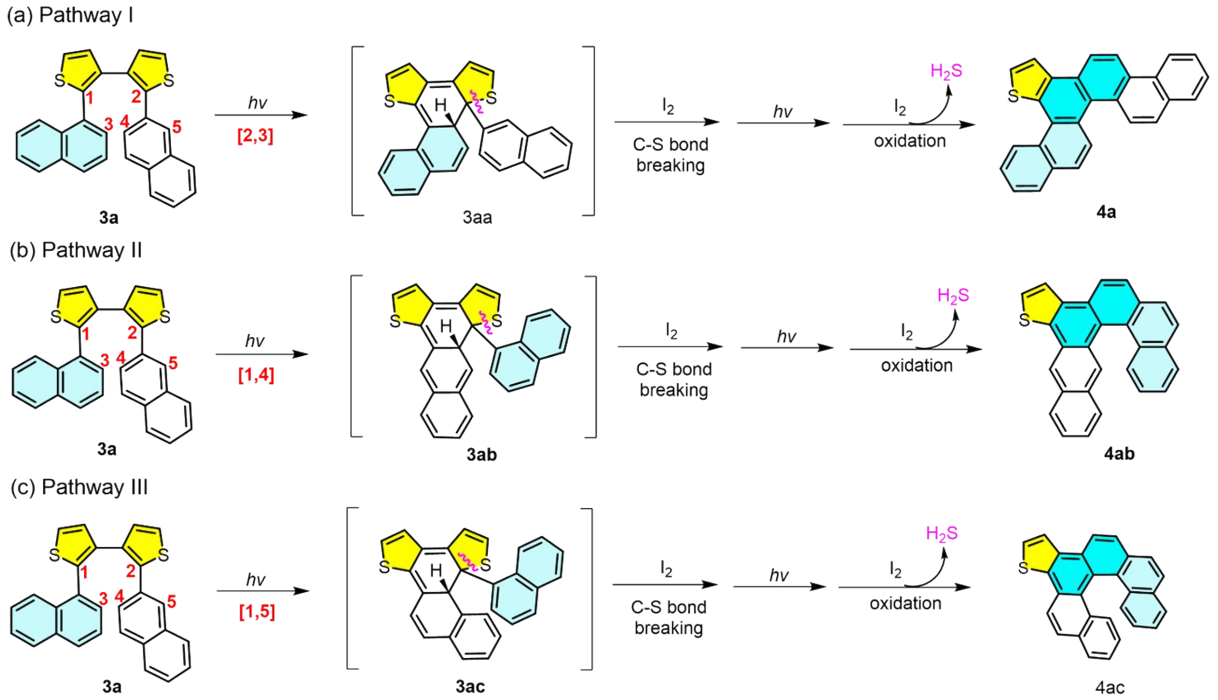

Figure S9. Proposed mechanism for photochemical cyclization and desulfurization of unsymmetric diaryl dithiophenes **3a** in pathway I (a), II (b) and III (c).

7. Photophysical Studies

UV-vis spectra were measured on a Shimadzu UV-1900 with a double-beam spectrophotometer at room temperature. Fluorescence spectra were measured on a JY HORIBA FluoroLog-3 spectrofluorometer. The fluorescence quantum yields (Φ_f) of **6c–6f** were characterized in dichloromethane with quinine sulfate ($\Phi_f = 0.55$, 1×10^{-5} M in 0.5 M H_2SO_4) as a standard[S5]. The radiative (k_r) and radiationless (k_{nr}) rates of were calculated based on $k_r = \Phi_f/\tau$ and $k_{nr} = (1-\Phi_f)/\tau$.

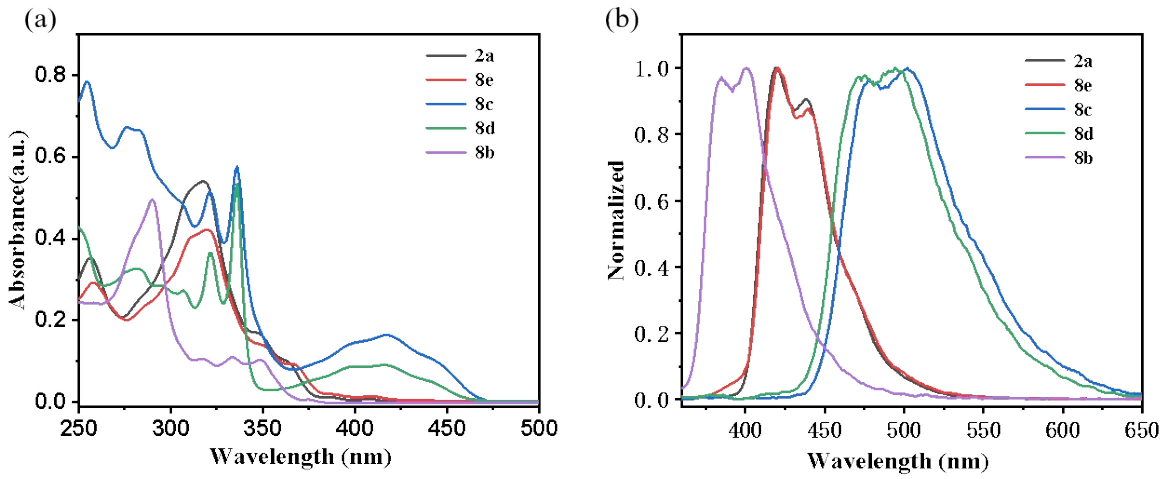

Figure S10. The UV–vis absorption spectra (a) and normalized fluorescence emission spectra (b) of **8b**–**8e** and **2a** in dichloromethane (c = 1.0 ×10^{-5} M; λ_{ex} = 340 nm).

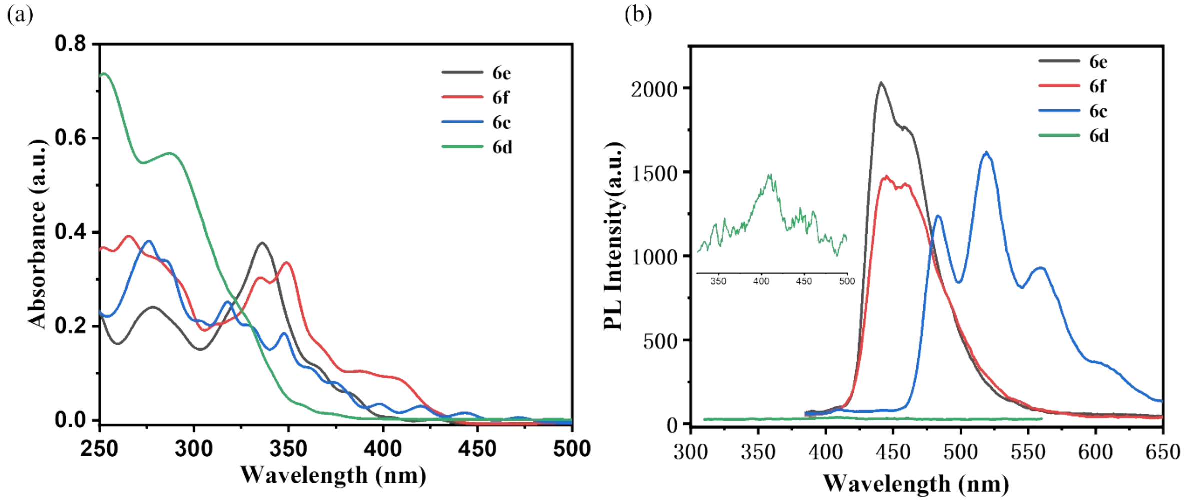

Figure S11. The UV–vis absorption spectra (a) and fluorescence emission spectra (b) of **6e**–**6f** in dichloromethane (c = 1.0 ×10^{-5} M; λ_{ex} = 365 nm for **6c**, **6e** and **6f**; λ_{ex} = 290 nm for **6d**).

Table S3. Photophysical properties of compounds **6c–6f**.

Compound	$\lambda_{abs, max}$ (nm)	λ_{em} (nm)	Φ_f (%)	τ (ns)	k_r (s^{-1})	k_{nr} (s^{-1})
**6c**	350, 420	519	11.4	1.30	0.09	0.68
**6d**	288	435	0.01	6.61	0.00002	0.15
**6e**	336	457	6.9	0.97	0.07	0.96
**6f**	338, 408	460	8.2	0.42	0.19	2.19

8. HPLC analysis of 6e and 6f

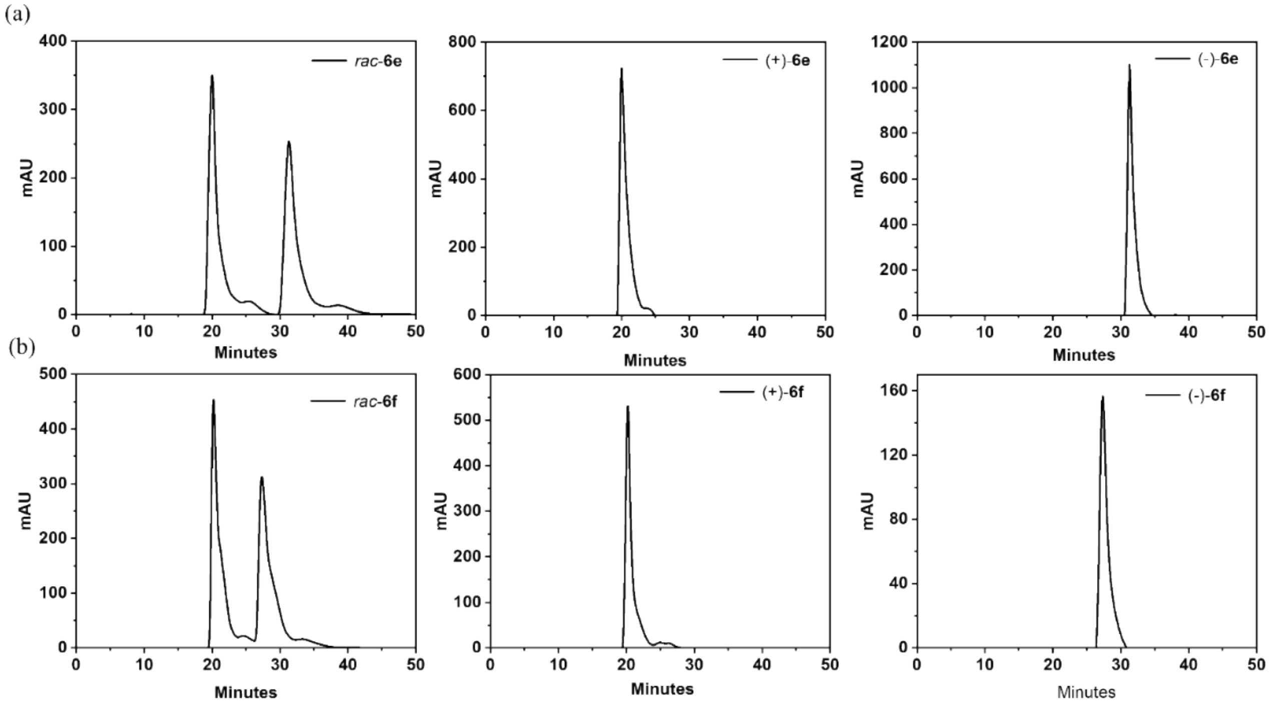

Figure S12. Elution profiles of *rac*-**6e** (a) and *rac*-**6f** (b) on Chiralpak ID (column, 25 × 0.46 cm (I.D.); eluent, hexane/DCM (3/1, v/v); flow rate, 1.0 mL min^{-1}; temperature, ca. 25 °C). The chromatograms depict UV traces recorded at 254 nm. The enantiomeric excess (*ee*) values were determined to be no less than 98%.

9. NMR Spectra and HRMS Spectra of New Compounds

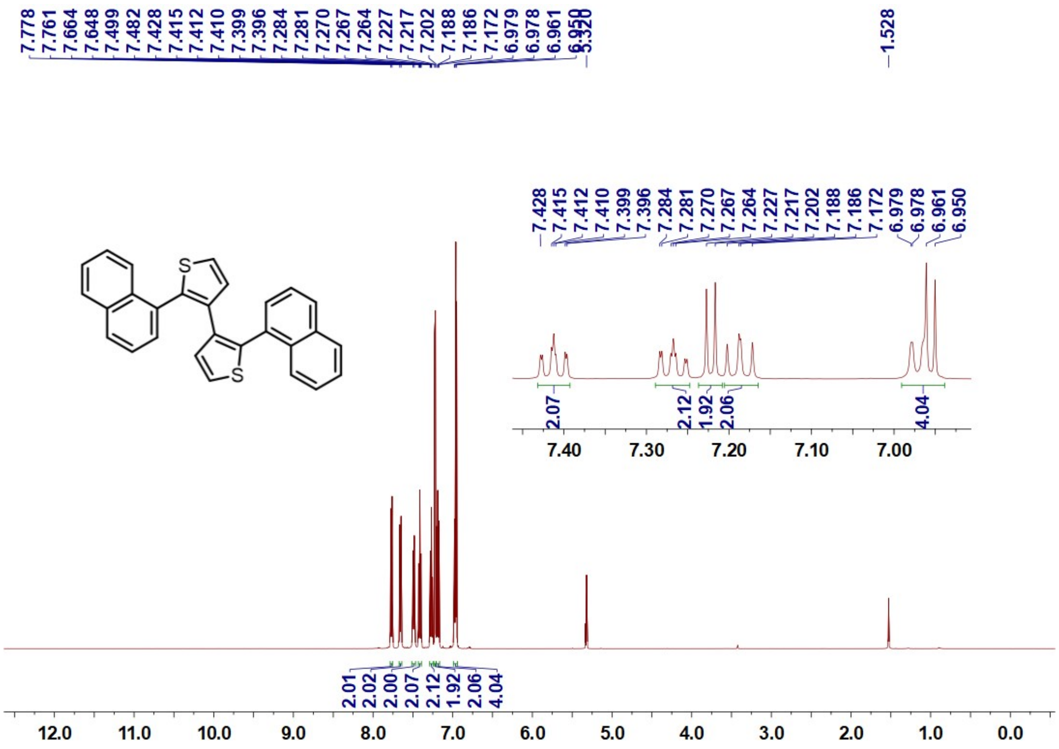

Figure 13. ^{1}H NMR (400 MHz, CD_2Cl_2) spectrum of **1a**.

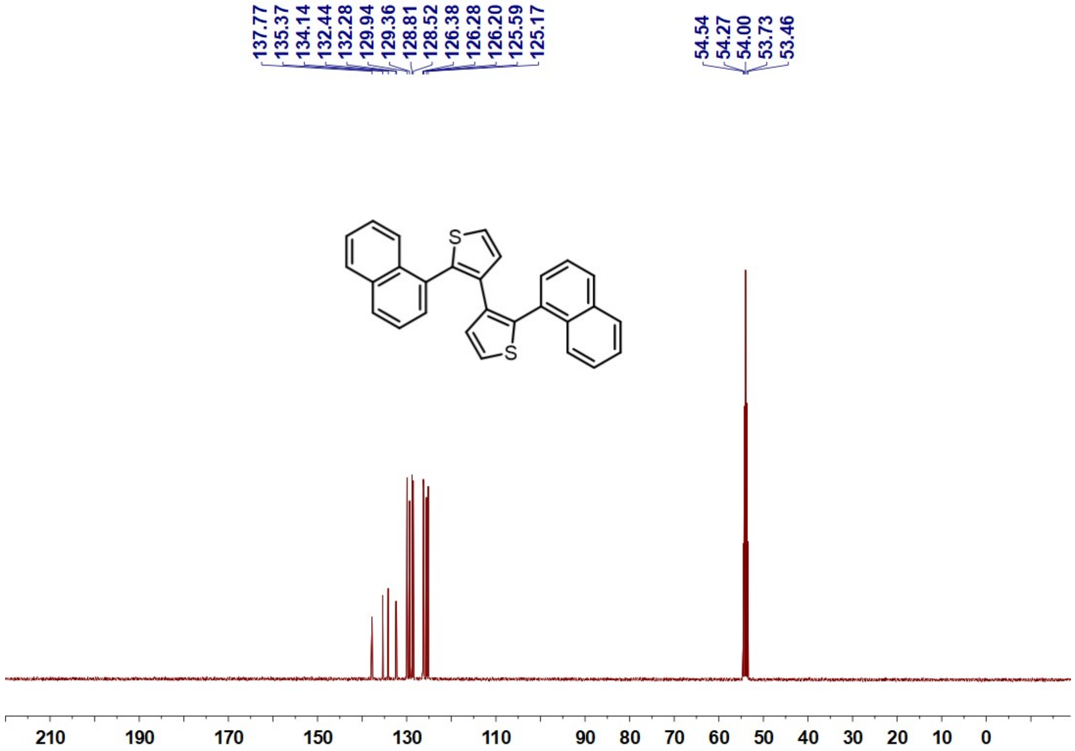

Figure 14. ^{13}C NMR (100 MHz, CD_2Cl_2) spectrum of **1a**.

National Center for Organic Mass Spectrometry in Shanghai
Shanghai Institute of Organic Chemistry
Chinese Academic of Sciences
High Resolution MS DATA REPORT

Instrument: Thermo Fisher Scientific LTQ FTICR-MS

Card Serial Number : D20220035

Sample Serial Number: SXL-1-23

Operator : DONG Date: 2021/12/02

Operation Mode: DART POSITIVE

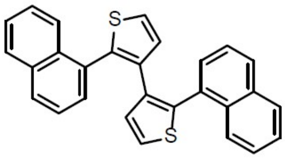

Elemental composition search on mass 419.0921

m/z= 414.0921-424.0921

m/z	Theo. Mass	Delta (ppm)	RDB equiv.	Composition
419.0921	419.0920	0.24	19.5	$C_{27}H_{19}OSSi$
	419.0923	-0.31	19.5	$C_{28}H_{19}S_2$
	419.0923	-0.40	1.0	$C_{14}H_{29}O_5NS_4$
	419.0916	1.25	10.5	$C_{20}H_{23}O_2N_2S_3$
	419.0916	1.28	0.5	$C_{12}H_{31}N_4S_5Si$
	419.0927	-1.43	25.5	$C_{28}H_{11}ON_4$
	419.0914	1.77	20.5	$C_{27}H_{15}O_5$
	419.0914	1.80	10.5	$C_{19}H_{23}O_3N_2S_2Si$
	419.0929	-1.92	0.0	$C_{14}H_{33}ONS_5Si$
	419.0932	-2.53	16.0	$C_{21}H_{17}O_5N_3Si$

Figure S15. HRMS-DART spectrum of **1a**.

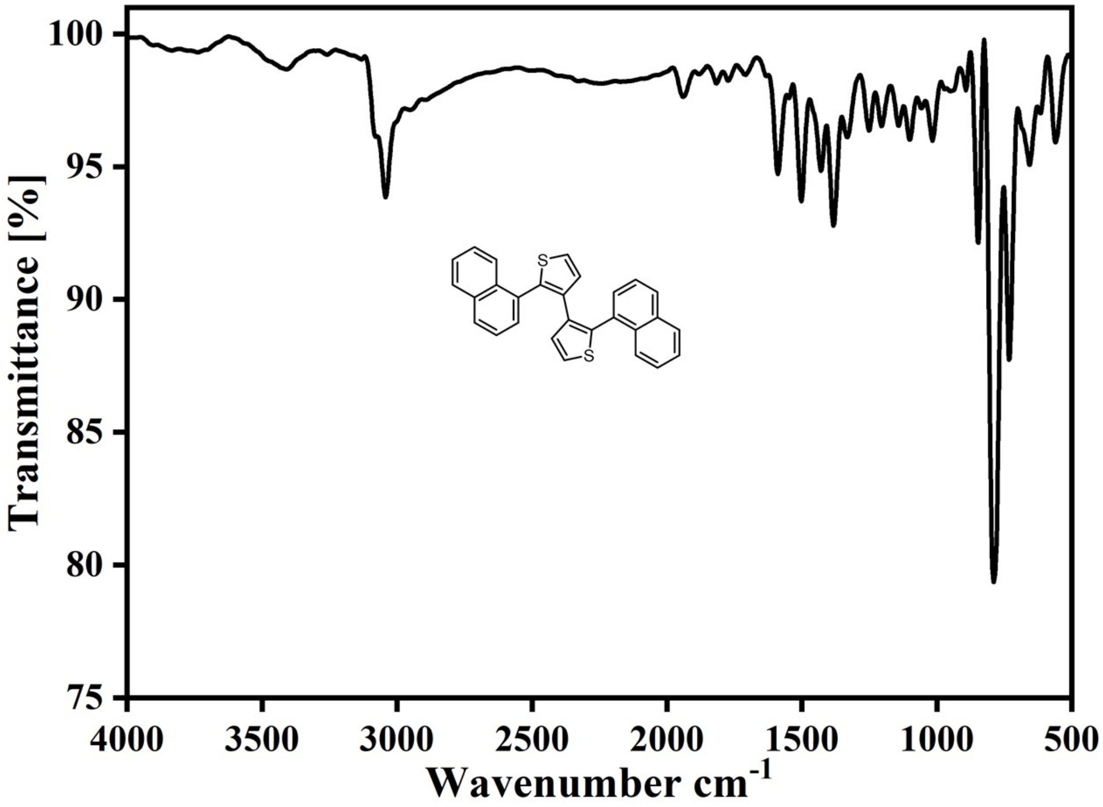

Figure S16. IR spectrum of **1a**.

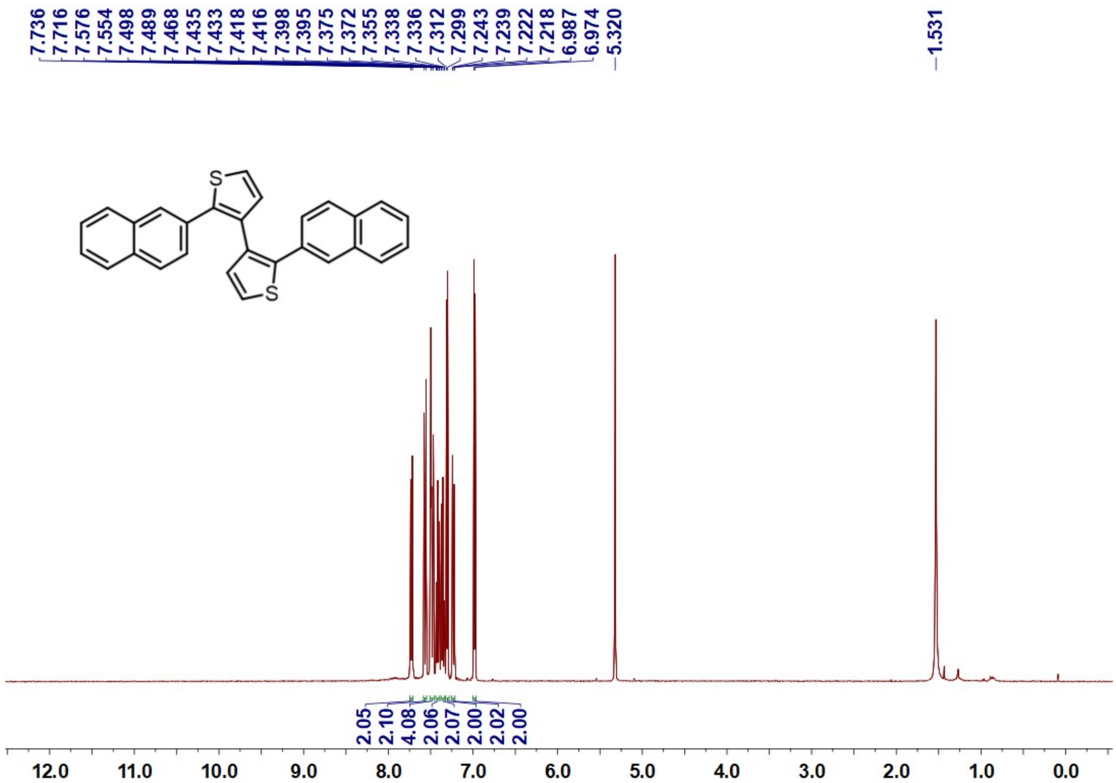

Figure S17. ^{1}H NMR (400 MHz, CD_2Cl_2) spectrum of **1b**.

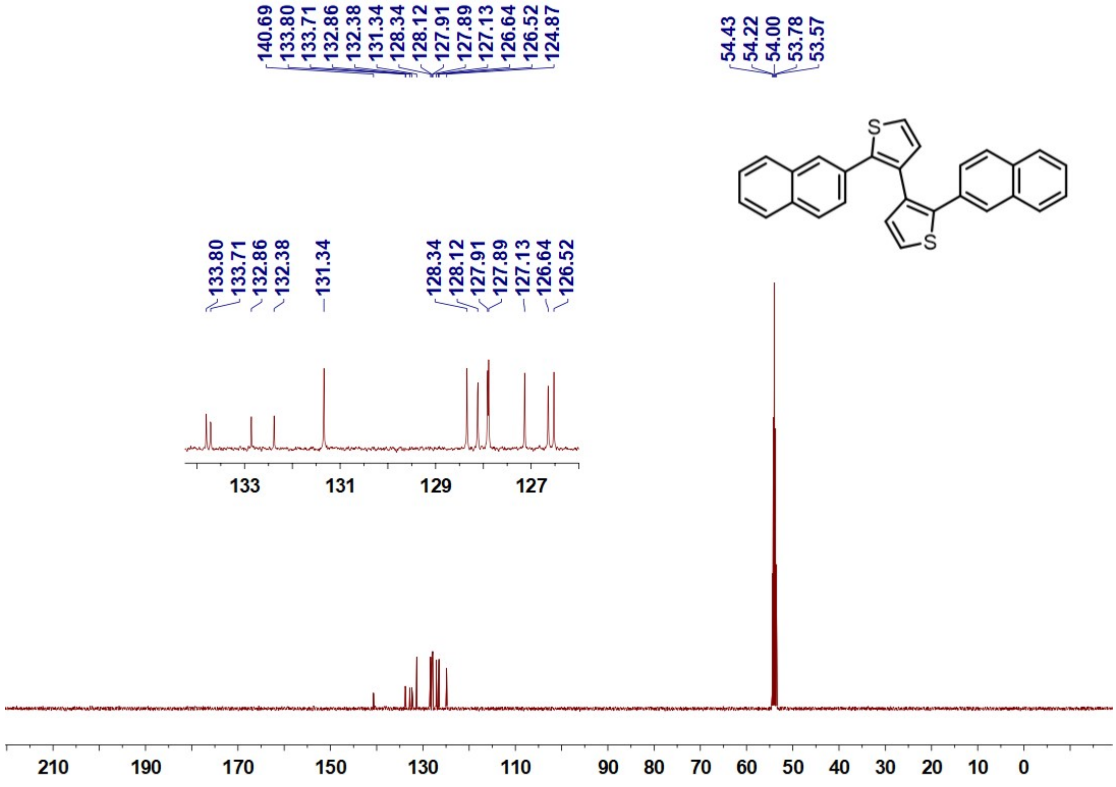

Figure S18. ^{13}C NMR (100 MHz, CD_2Cl_2) spectrum of **1b**.

National Center for Organic Mass Spectrometry in Shanghai
Shanghai Institute of Organic Chemistry
Chinese Academic of Sciences
High Resolution MALDI-MS REPORT

Instrument: Thermo Scientific Q Exactive HF Orbitrap-FTMS

Card Serial Number: E-W201277

Sample Serial Number: SXL-1-27-COL-PP

Operator: Songw Date: 2020/11/03

Operation Mode: MALDI Positive Ion Mode

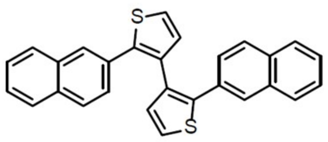

Elemental composition search on mass 418.08

m/z= 413.08-423.08

m/z	Theo. Mass	Delta (ppm)	RDB equiv.	Composition
418.0840	418.0844	-0.94	20.0	$C_{28}H_{18}S_2$

Figure S19. HRMS-MALDI spectrum of **1b**.

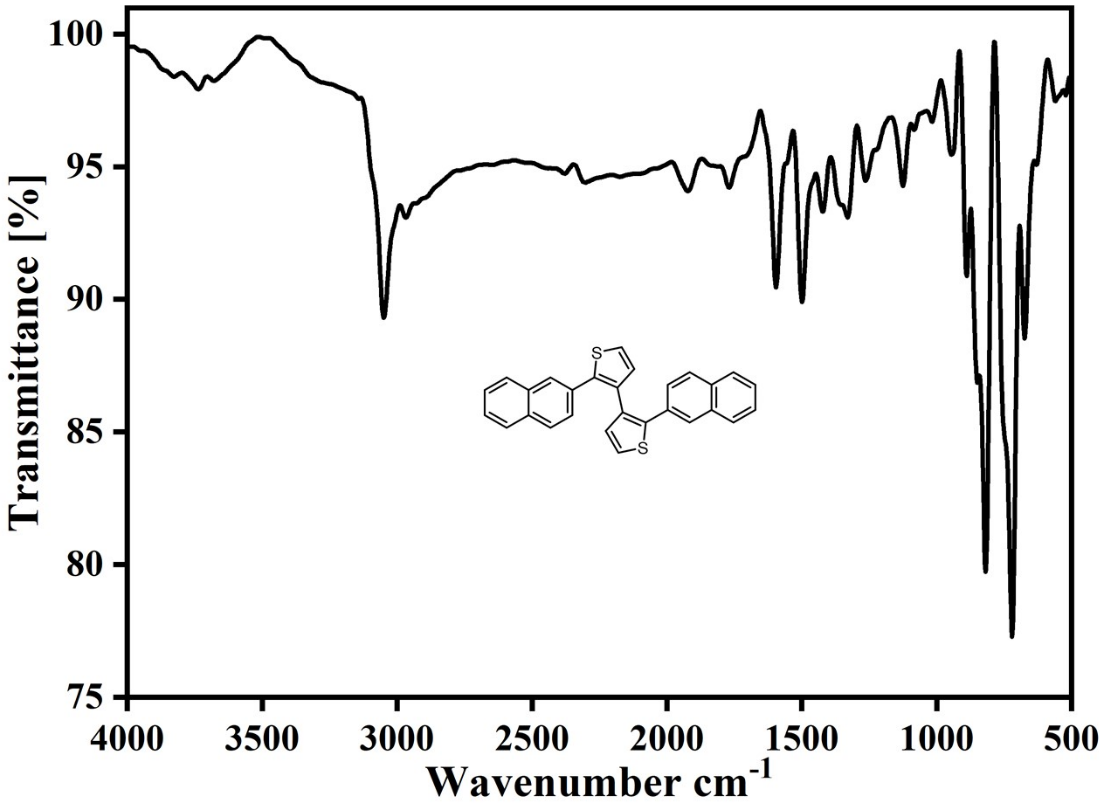

Figure S20. IR spectrum of **1b**.

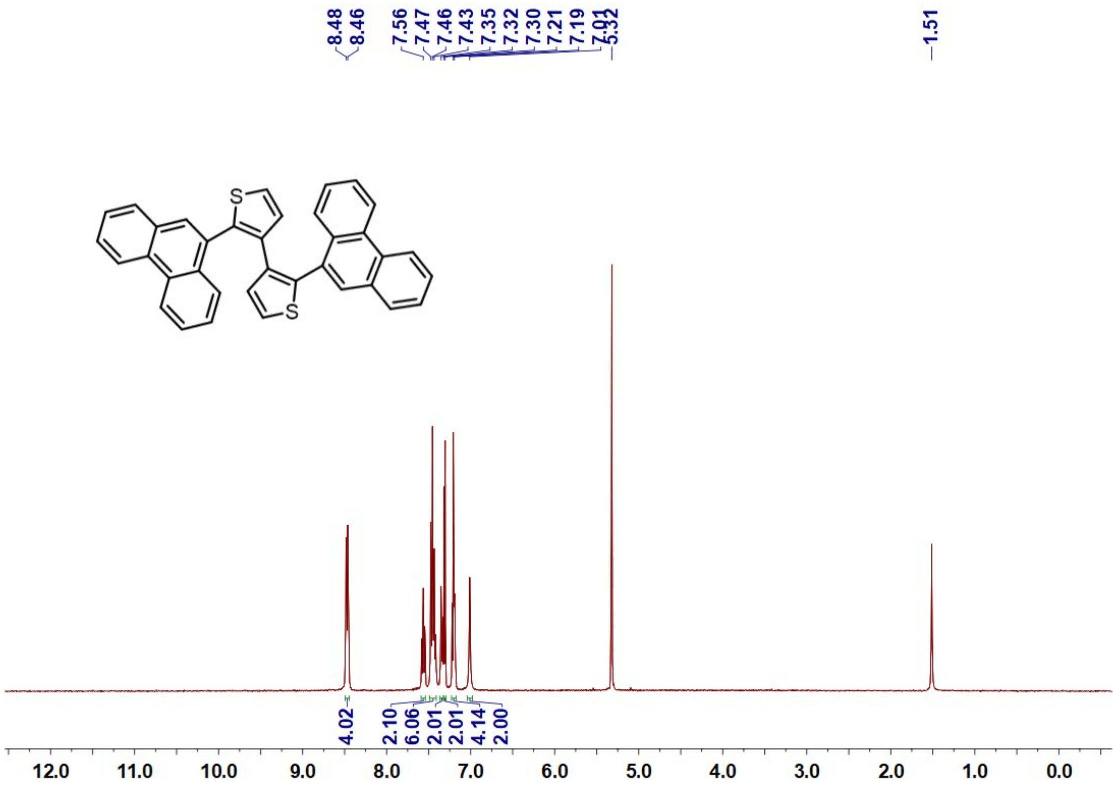

Figure S21. ^{1}H NMR (400 MHz, CD_2Cl_2) spectrum of **1c**.

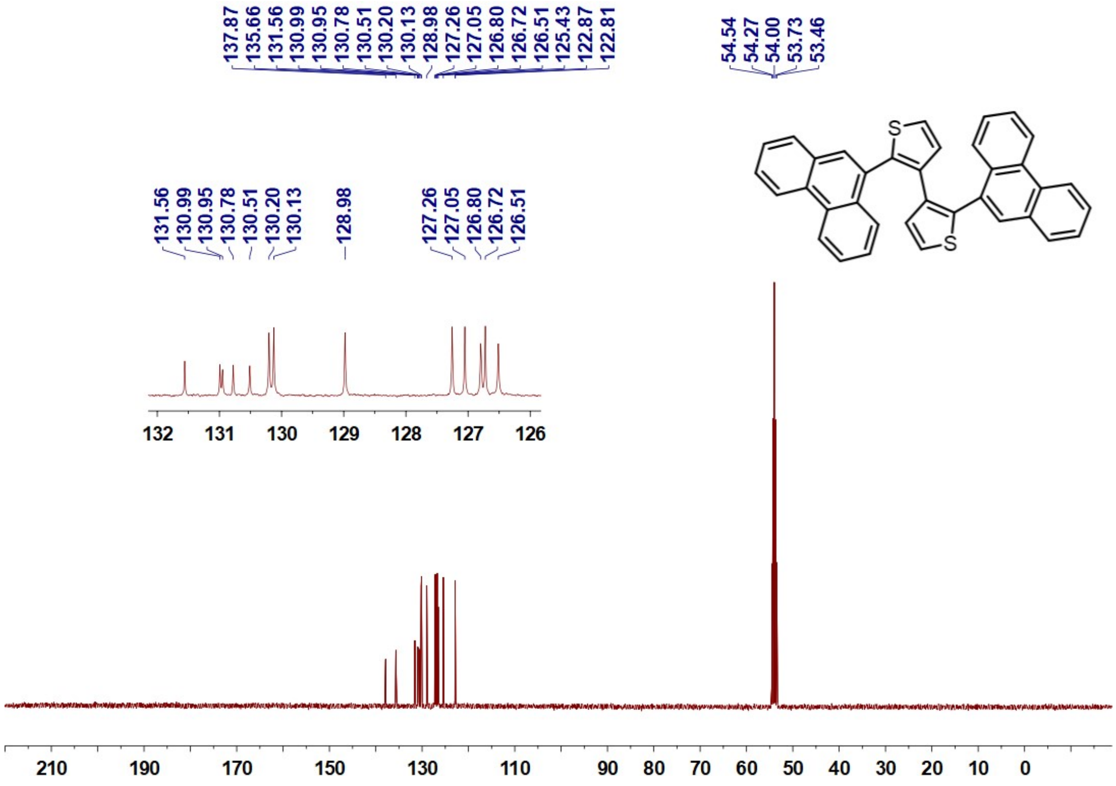

Figure S22. ^{1}H NMR (100 MHz, CD_2Cl_2) spectrum of **1c**.

National Center for Organic Mass Spectrometry in Shanghai
Shanghai Institute of Organic Chemistry
Chinese Academic of Sciences
HIGH RESOLUTION MS REPORT

Instrument: JMS-S3000 MALDI-TOFMS

Sample Serial Number: SXL-1-53-COL-PP

Operator: Zhang, Li

Date: 2021/03/12

Operation Mode: MALDI-Positive

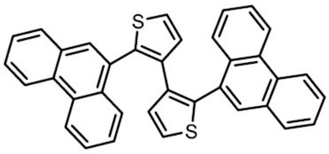

Elemental Composition Estimation

Parameters:

Mass	Tolerance	Electron Mode	Charge	DBE Range	Max Results
518.11462 ± 0.00259	5.0 ppm	Odd/Even	+1	-0.5 - 200.0	100

Elements

C	H	S	O	Na
0 - 36	0 - 200	0 - 7	0 - 0	0 - 0

Results:

#	Formula	Mass	DBE	Abs. Error (u)	Error (u)	Error (ppm)
1	C36 H22 S2	518.11574	26.0	0.00113	-0.00113	-2.18

Figure S23. HRMS-MALDI spectrum of **1c**.

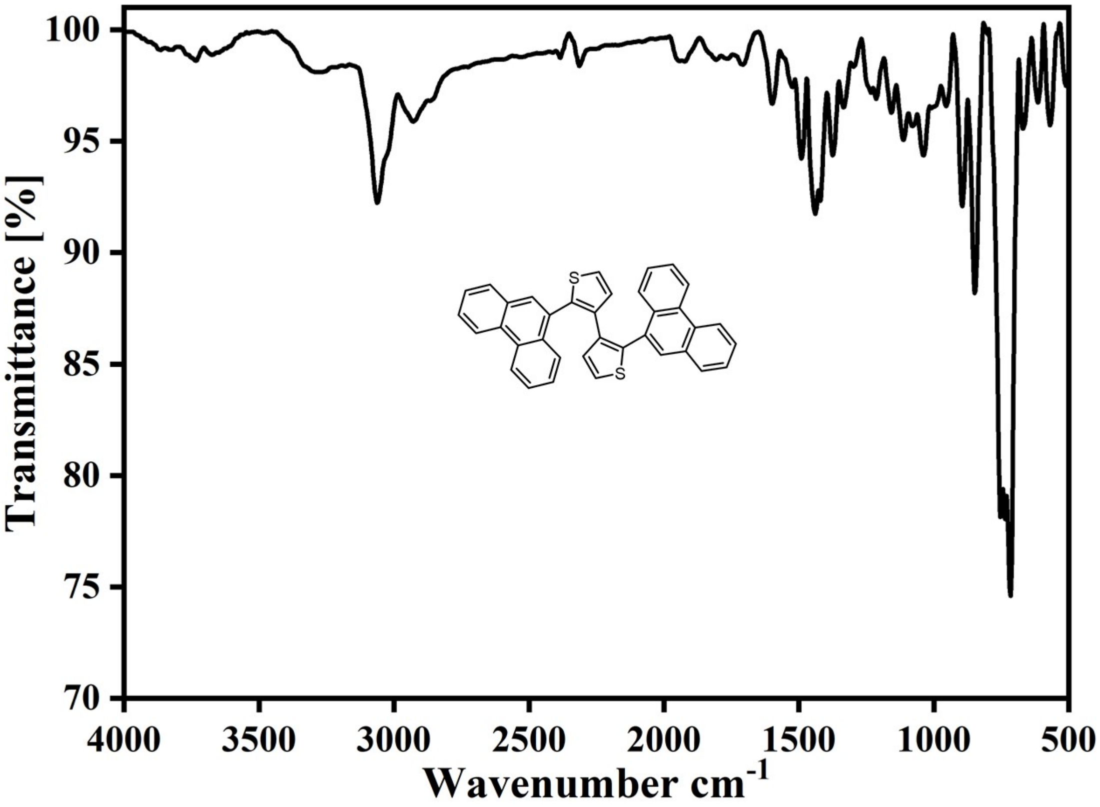

Figure S24. IR spectrum of **1c**.

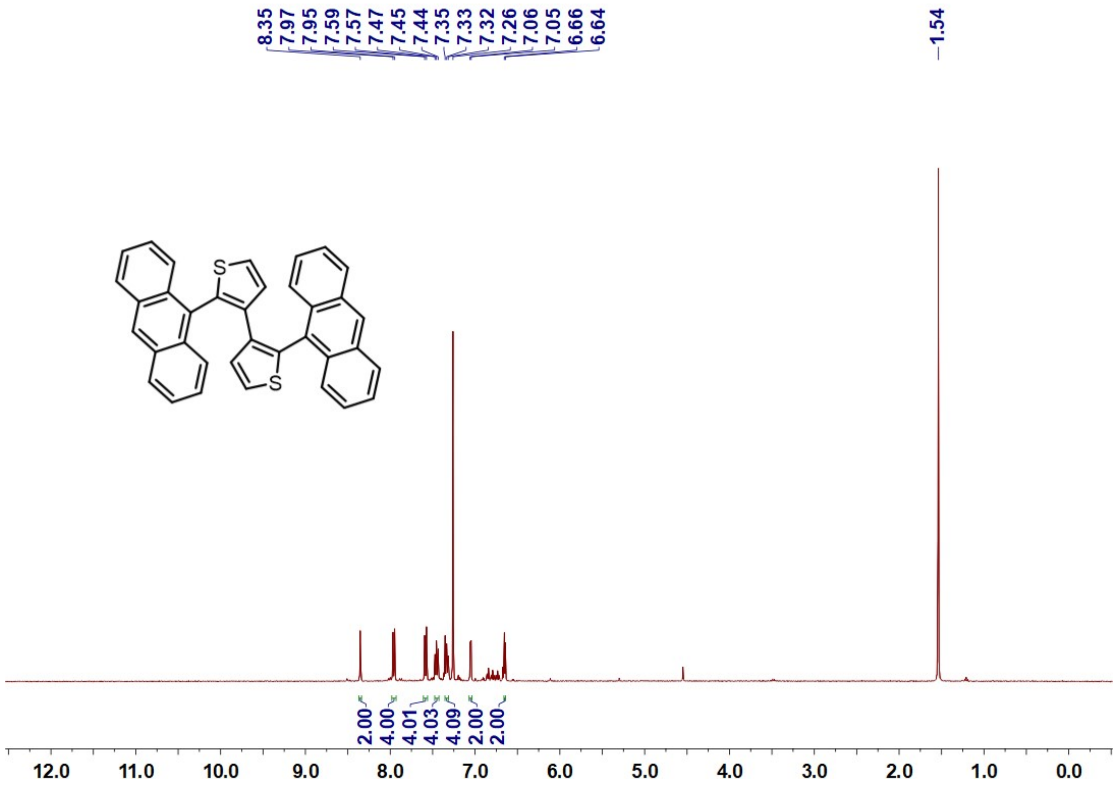

Figure S25. ^{1}H NMR (400 MHz, $CDCl_3$) spectrum of **1d**.

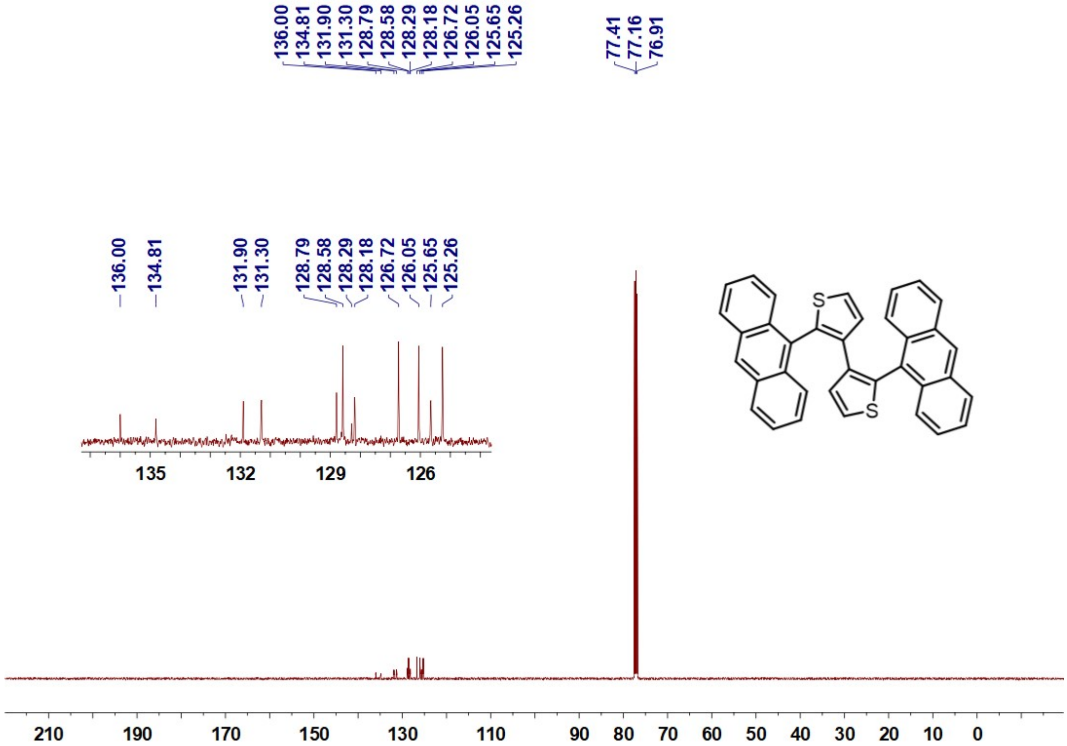

Figure S26. ^{13}C NMR (100 MHz, $CDCl_3$) spectrum of **1d**.

National Center for Organic Mass Spectrometry in Shanghai
Shanghai Institute of Organic Chemistry
Chinese Academic of Sciences
High Resolution MS DATA REPORT

Instrument: Thermo Fisher Scientific LTQ FTICR-MS

Card Serial Number : D20220031

Sample Serial Number: SXL-1-42

Operator : DONG Date: 2021/12/02

Operation Mode: DART POSITIVE

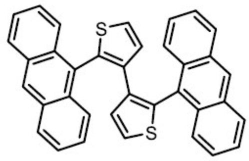

Elemental composition search on mass 519.1233

m/z= 514.1233-524.1233

m/z	Theo. Mass	Delta (ppm)	RDB equiv.	Composition
519.1233	519.1233	0.00	25.5	$C_{35}H_{23}OSSi$
	519.1236	-0.44	25.5	$C_{36}H_{23}S_2$
	519.1236	-0.52	7.0	$C_{22}H_{33}O_5NS_4$
	519.1229	0.82	16.5	$C_{28}H_{27}O_2N_2S_3$
	519.1229	0.84	6.5	$C_{20}H_{35}N_4S_5Si$
	519.1227	1.23	26.5	$C_{35}H_{19}O_5$
	519.1227	1.26	16.5	$C_{27}H_{27}O_3N_2S_2Si$
	519.1240	-1.34	31.5	$C_{36}H_{15}ON_4$
	519.1242	-1.75	6.0	$C_{22}H_{37}ONS_5Si$
	519.1223	2.07	7.5	$C_{20}H_{31}O_4N_4S_4$

Figure S27. HRMS-DART spectrum of **1d**.

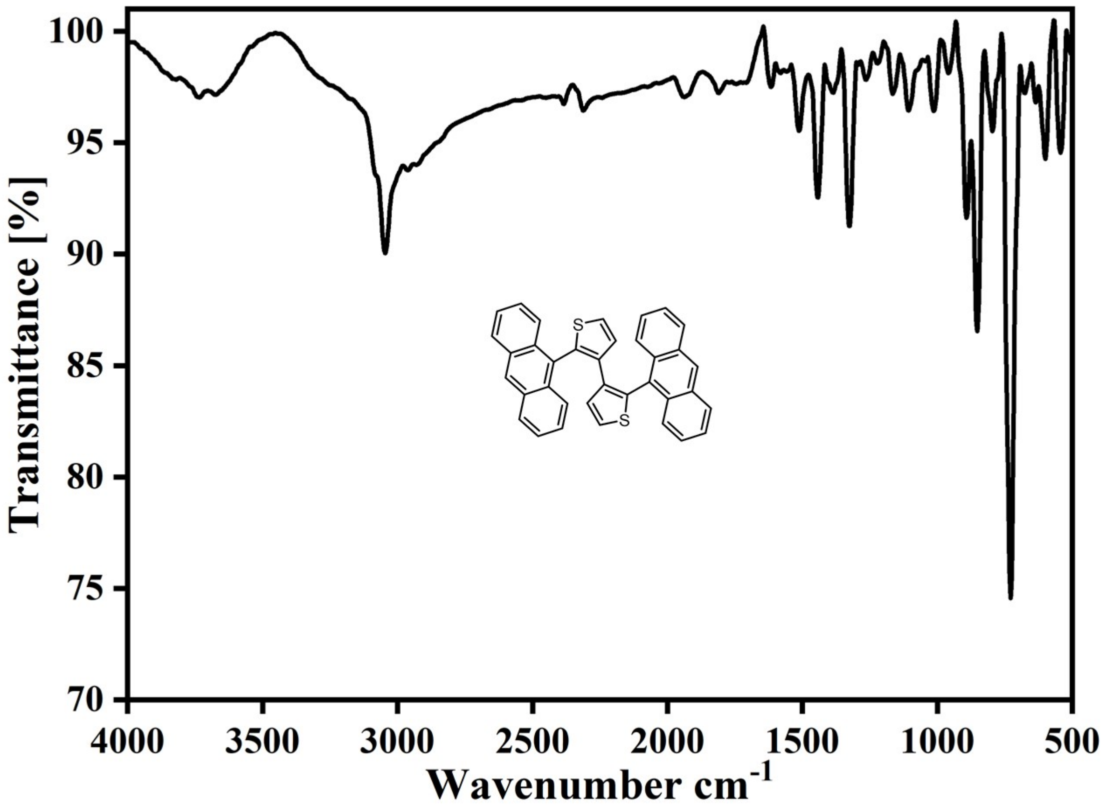

Figure S28. IR spectrum of **1d**.

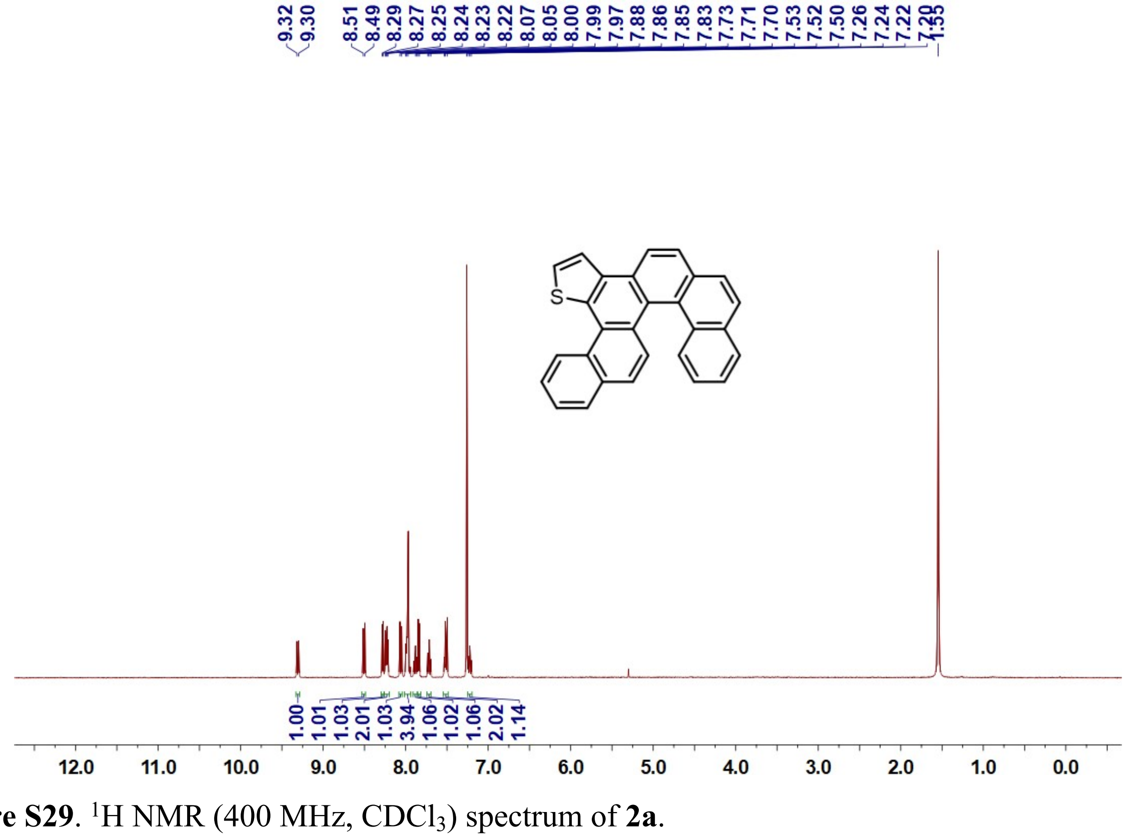

Figure S29. ^{1}H NMR (400 MHz, $CDCl_3$) spectrum of **2a**.

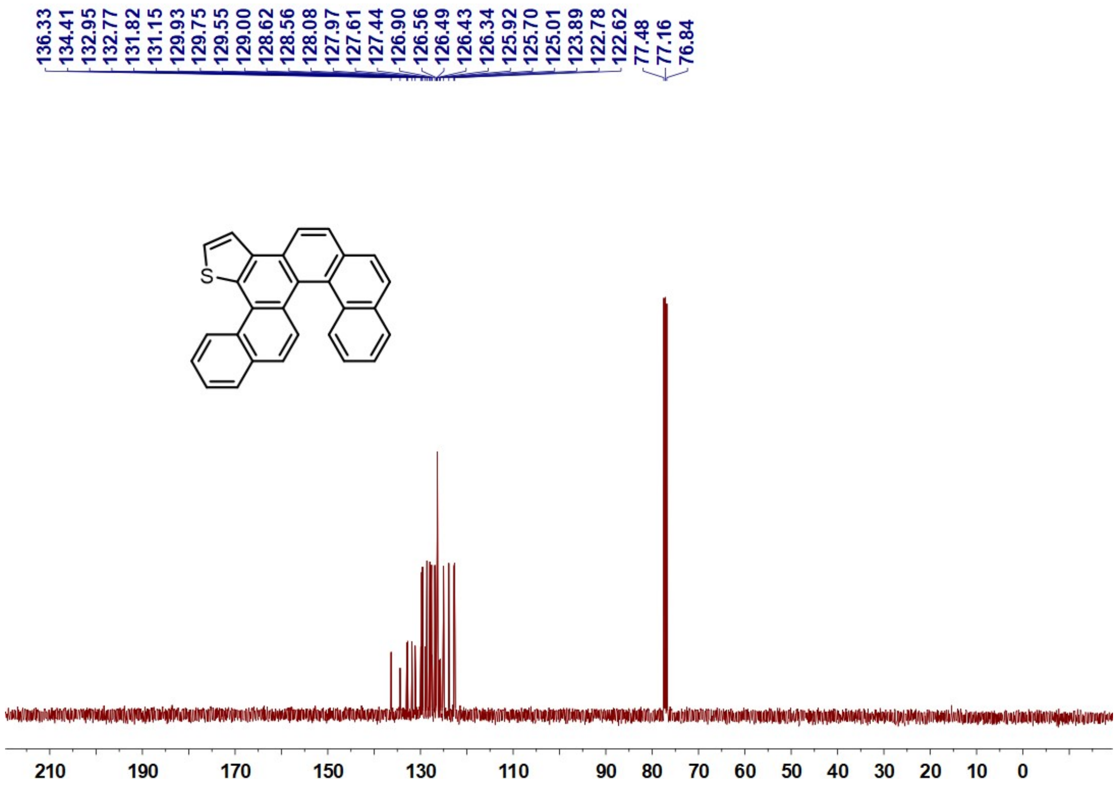

Figure S30. ^{13}C NMR (100 MHz, $CDCl_3$) spectrum of **2a**.

National Center for Organic Mass Spectrometry in Shanghai
Shanghai Institute of Organic Chemistry
Chinese Academic of Sciences
HIGH RESOLUTION MS REPORT

Instrument: JMS-S3000 MALDI-TOFMS

Sample Serial Number: SXL-2-47-COL-PP-HR

Operator: Zhang, Li

Date: 2021/08/18

Operation Mode: MALDI-Positive

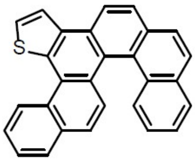

Elemental Composition Estimation

Parameters:

Mass	Tolerance	Electron Mode	Charge	DBE Range	Max Results
384.09544 ± 0.00192	5.0 ppm	Odd/Even	+1	-0.5 - 200.0	100

Elements

C	0 - 28	H	0 - 200	Br	0 - 0	Si	0 - 0	S	0 - 1

Results:

#	Formula	Mass	DBE	Abs. Error (u)	Error (u)	Error (ppm)
1	C28 H16 S	384.09672	21.0	0.00128	-0.00128	-3.34

Figure S31. HRMS-MADIL spectrum of **2a**.

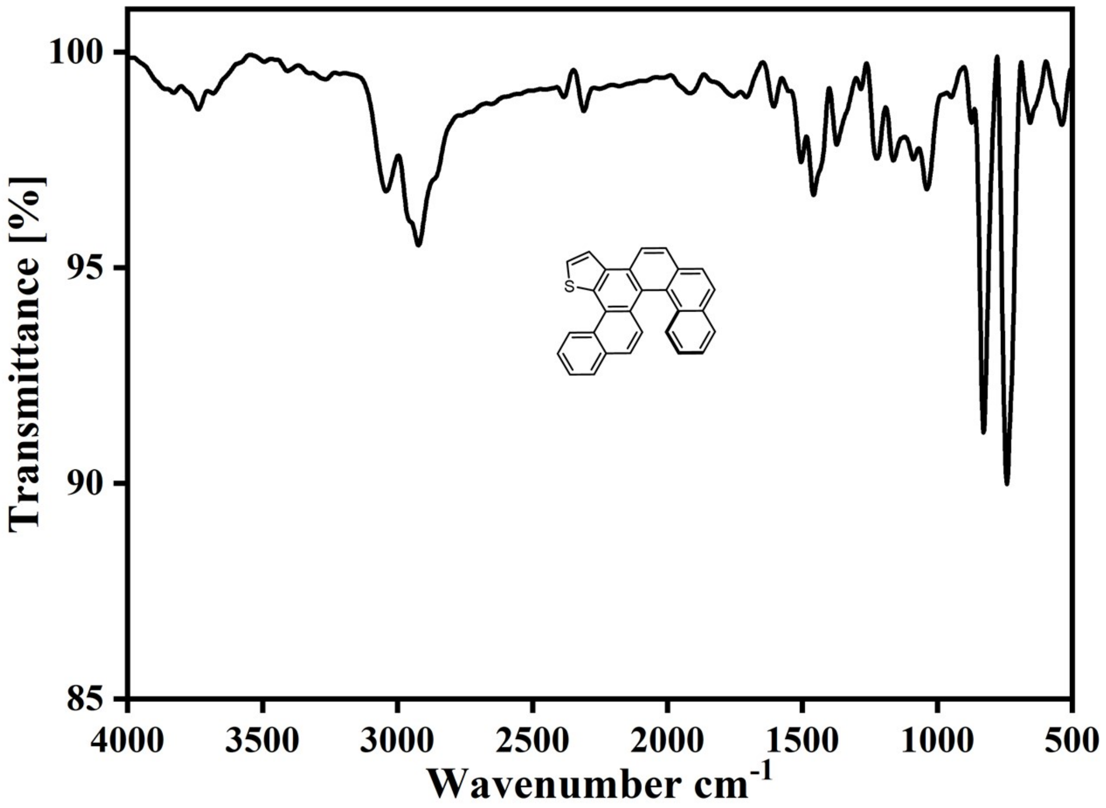

Figure S32. IR spectrum of **2a**.

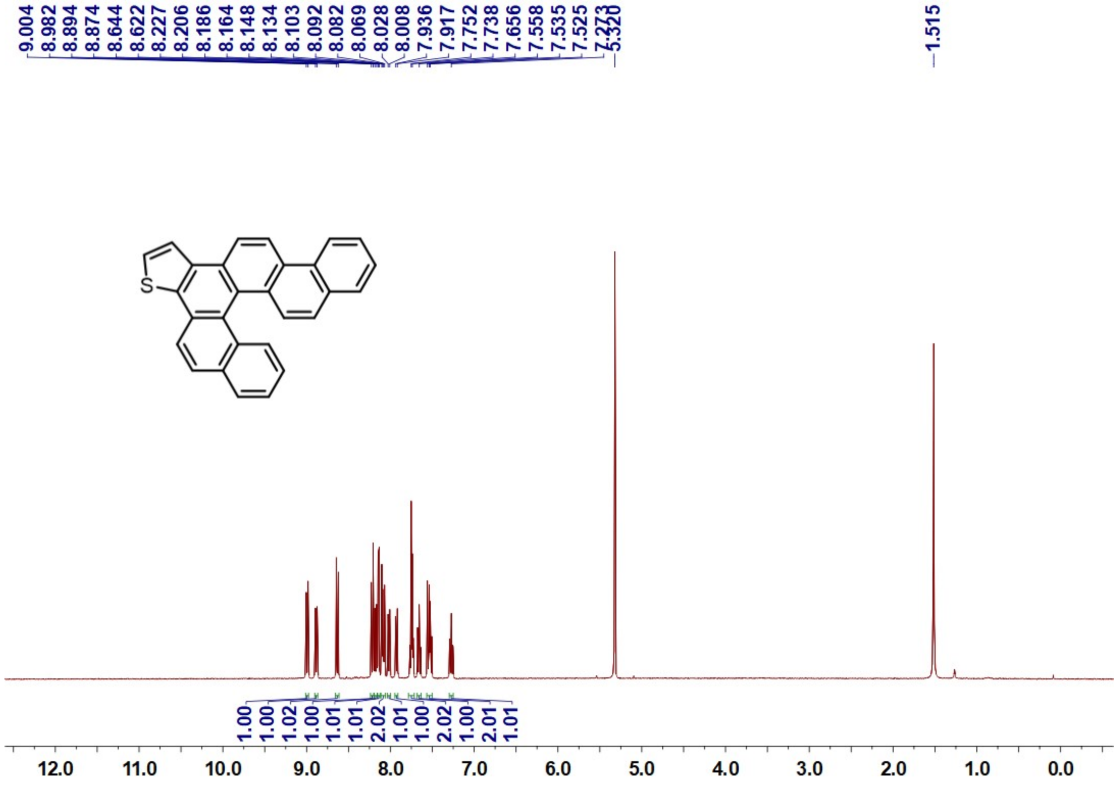

Figure S33. ^{1}H NMR (400 MHz, CD_2Cl_2) spectrum of **2b**.

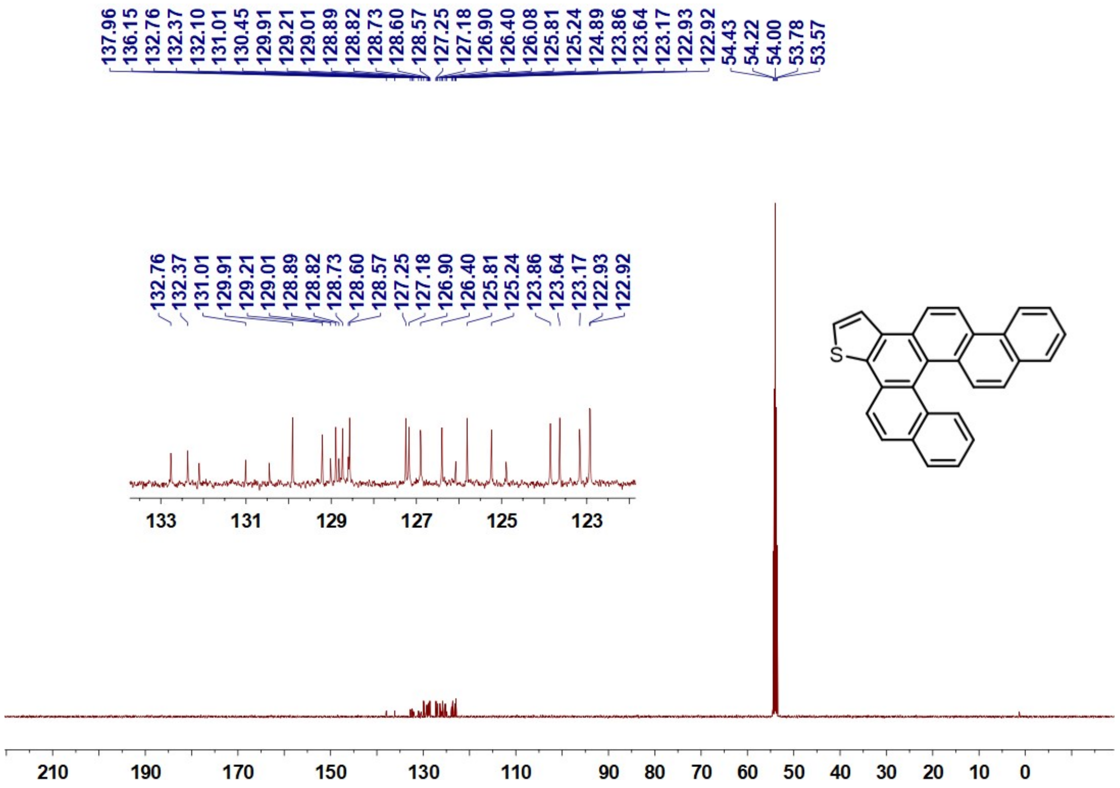

Figure S34. ^{13}C NMR (100 MHz, CD_2Cl_2) spectrum of **2b**.

National Center for Organic Mass Spectrometry in Shanghai
Shanghai Institute of Organic Chemistry
Chinese Academic of Sciences
High Resolution MALDI-MS REPORT

Instrument: Thermo Scientific Q Exactive HF Orbitrap-FTMS

Card Serial Number: E-W201278

Sample Serial Number: SXL-1-48-COL-PP

Operator: Songw Date: 2020/11/03

Operation Mode: MALDI Positive Ion Mode

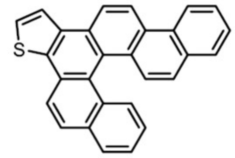

Elemental composition search on mass 384.10

m/z= 379.10-389.10

m/z	Theo. Mass	Delta (ppm)	RDB equiv.	Composition
384.0962	384.0967	-1.31	21.0	$C_{28}H_{16}S$

Figure S35. HRMS-MALDI spectrum of **2b**.

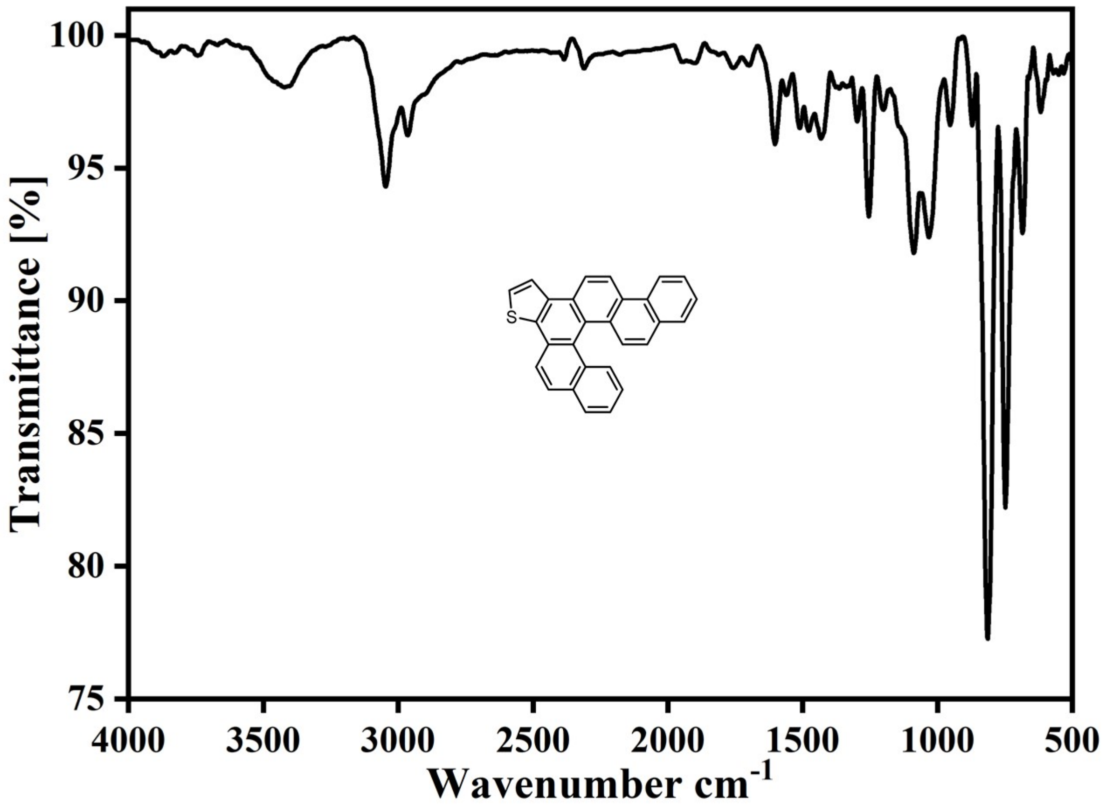

Figure S36. IR spectrum of **2b.**

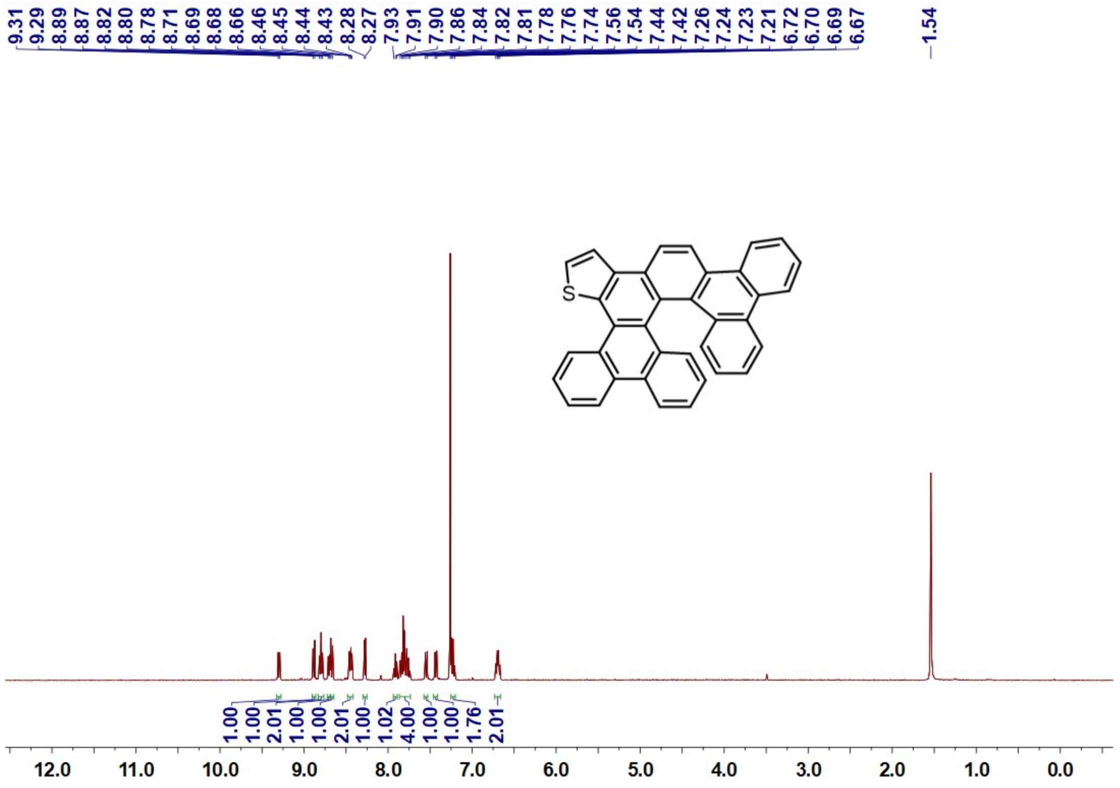

Figure S37. ^{1}H NMR (400 MHz, $CDCl_3$) spectra of **2c**.

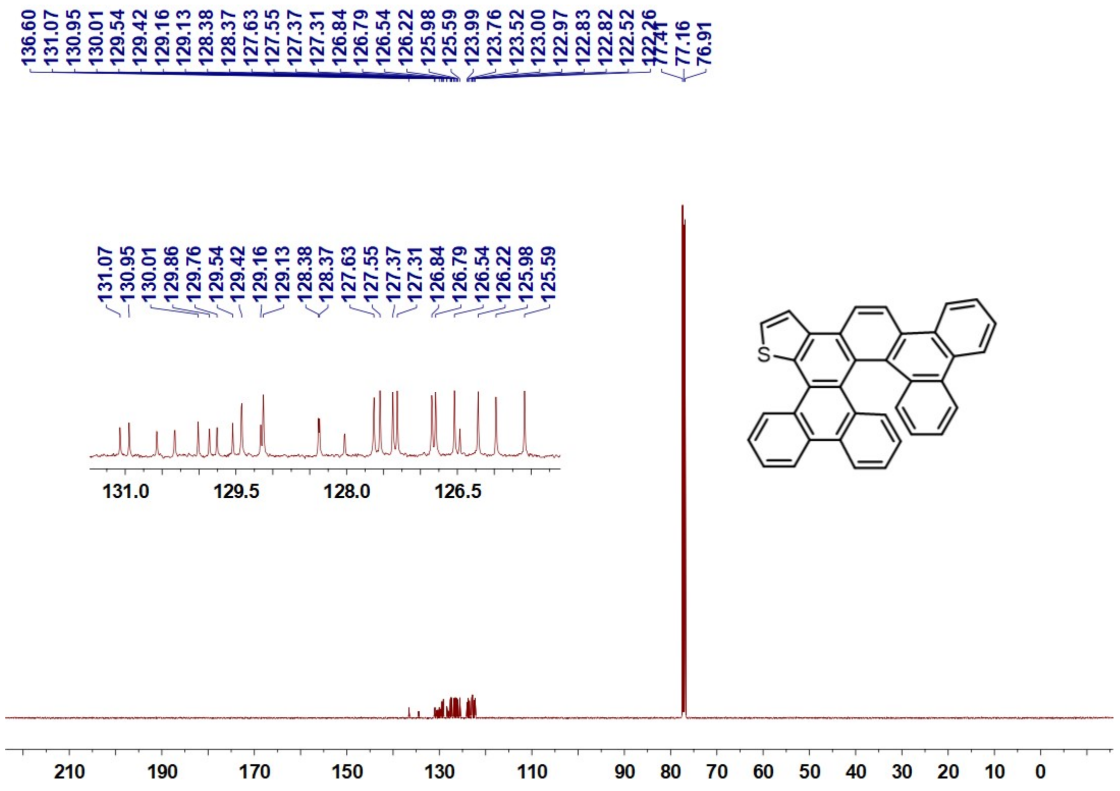

Figure S38. ^{13}C NMR (100 MHz, $CDCl_3$) spectra of **2c**

National Center for Organic Mass Spectrometry in Shanghai
Shanghai Institute of Organic Chemistry
Chinese Academic of Sciences
HIGH RESOLUTION MS REPORT

Instrument: JMS-S3000 MALDI-TOFMS

Sample Serial Number: SXL-1-74-COL-PP-CRY

Operator: Zhang, Li

Date: 2021/03/12

Operation Mode: MALDI-Positive

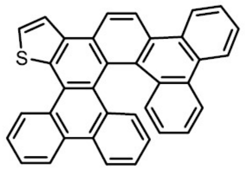

Elemental Composition Estimation

Parameters:

Mass	Tolerance	Electron Mode	Charge	DBE Range	Max Results
484.12774 ± 0.00242	5.0 ppm	Odd/Even	+1	-0.5 - 200.0	100

Elements

C	H	S	N	Si
0 - 36	0 - 70	0 - 1	0 - 0	0 - 3

Results:

#	Formula	Mass	DBE	Abs. Error (u)	Error (u)	Error (ppm)
1	C36 H20 S	484.12802	27.0	0.00028	-0.00028	-0.59

Figure S39. HRMS-MALDI spectrum of **2c**.

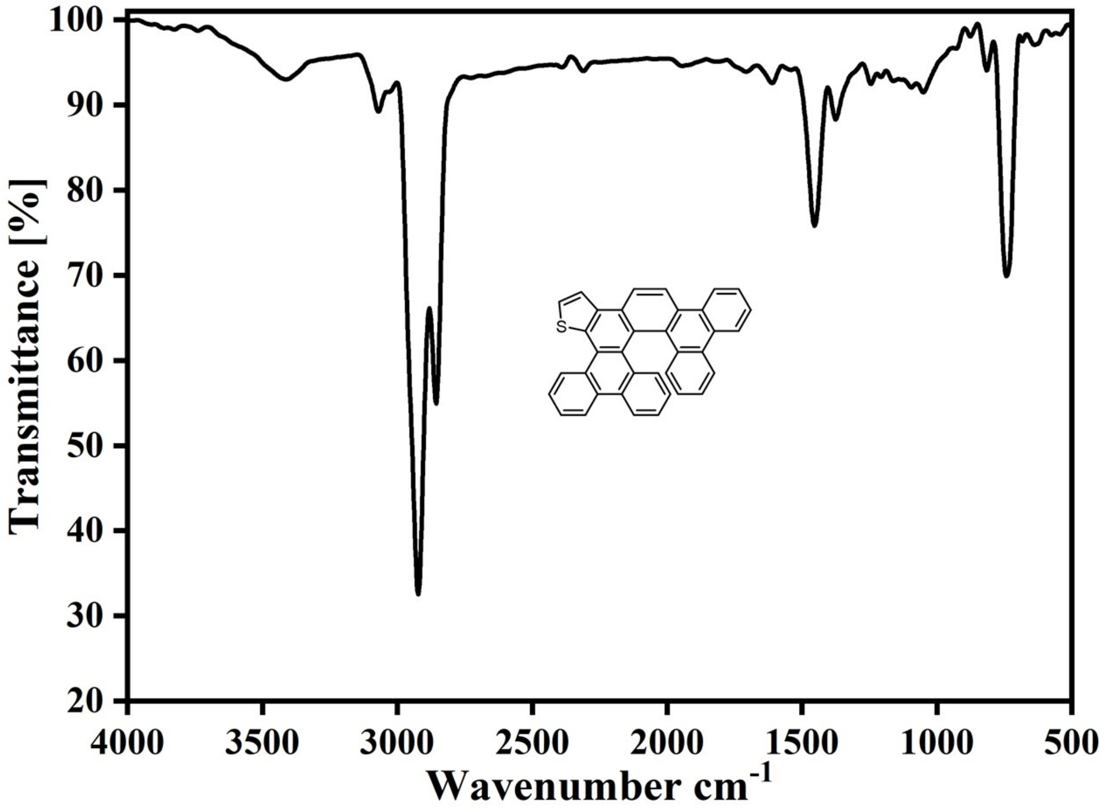

Figure S40. IR spectrum of **2c**.

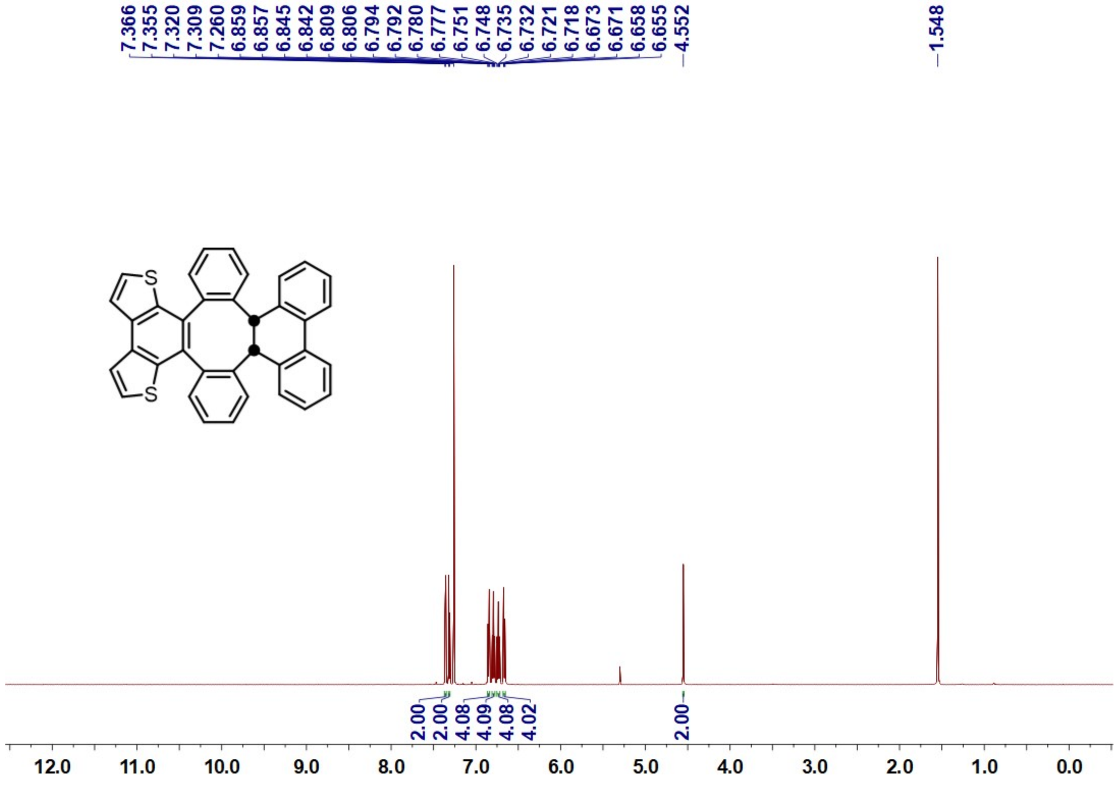

Figure S41. ^{1}H NMR (400 MHz, $CDCl_3$) spectrum of **2d**.

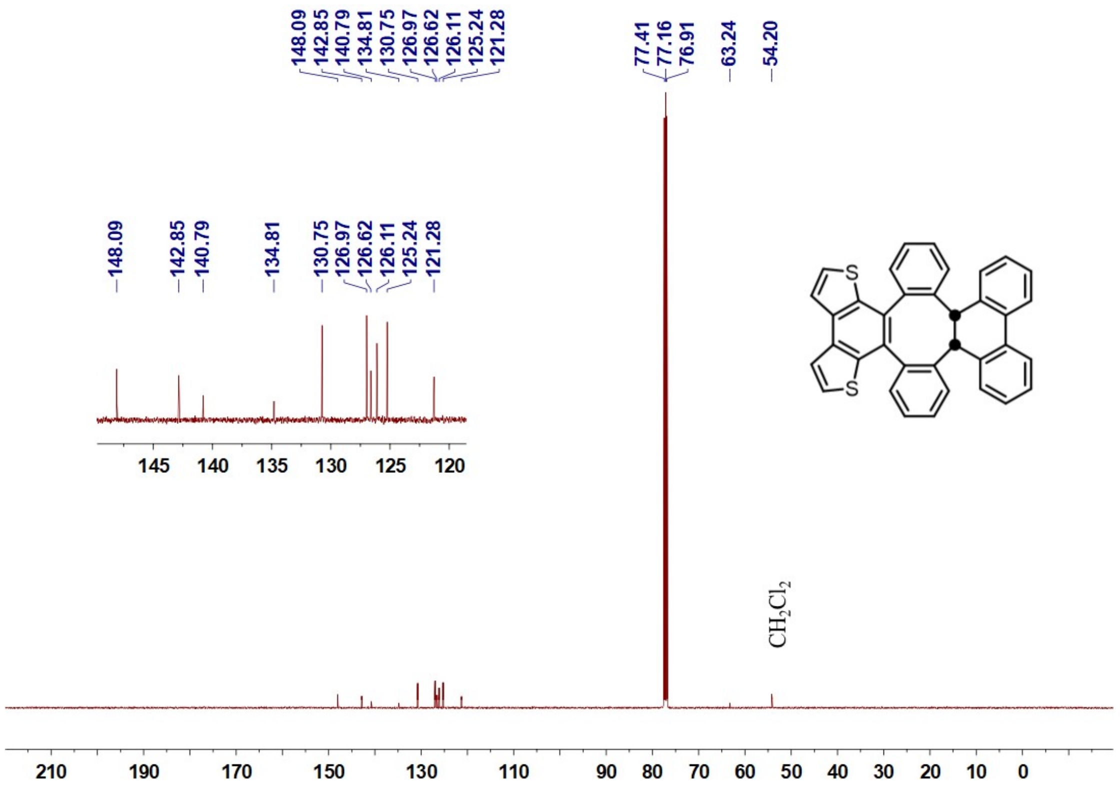

Figure S42. ^{13}C NMR (100 MHz, $CDCl_3$) spectra of **2d**

National Center for Organic Mass Spectrometry in Shanghai
Shanghai Institute of Organic Chemistry
Chinese Academic of Sciences
High Resolution MS DATA REPORT

Instrument: Thermo Fisher Scientific LTQ FTICR-MS

Card Serial Number : D20220033

Sample Serial Number: SXL-1-52

Operator : DONG Date: 2021/12/02

Operation Mode: DART POSITIVE

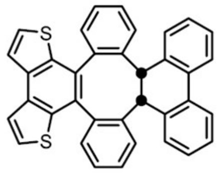

Elemental composition search on mass 519.1235

m/z= 514.1235-524.1235

m/z	Theo. Mass	Delta (ppm)	RDB equiv.	Composition
519.1235	519.1233	0.21	25.5	$C_{35}H_{23}OSSi$
	519.1236	-0.23	25.5	$C_{36}H_{23}S_2$
	519.1236	-0.30	7.0	$C_{22}H_{33}O_5NS_4$
	519.1229	1.03	16.5	$C_{28}H_{27}O_2N_2S_3$
	519.1229	1.05	6.5	$C_{20}H_{35}N_4S_5Si$
	519.1240	-1.13	31.5	$C_{36}H_{15}ON_4$
	519.1227	1.44	26.5	$C_{35}H_{19}O_5$
	519.1227	1.47	16.5	$C_{27}H_{27}O_3N_2S_2Si$
	519.1242	-1.53	6.0	$C_{22}H_{37}ONS_5Si$
	519.1245	-2.02	22.0	$C_{29}H_{21}O_5N_3Si$

Figure S43. HRMS-DART spectrum of **2d**.

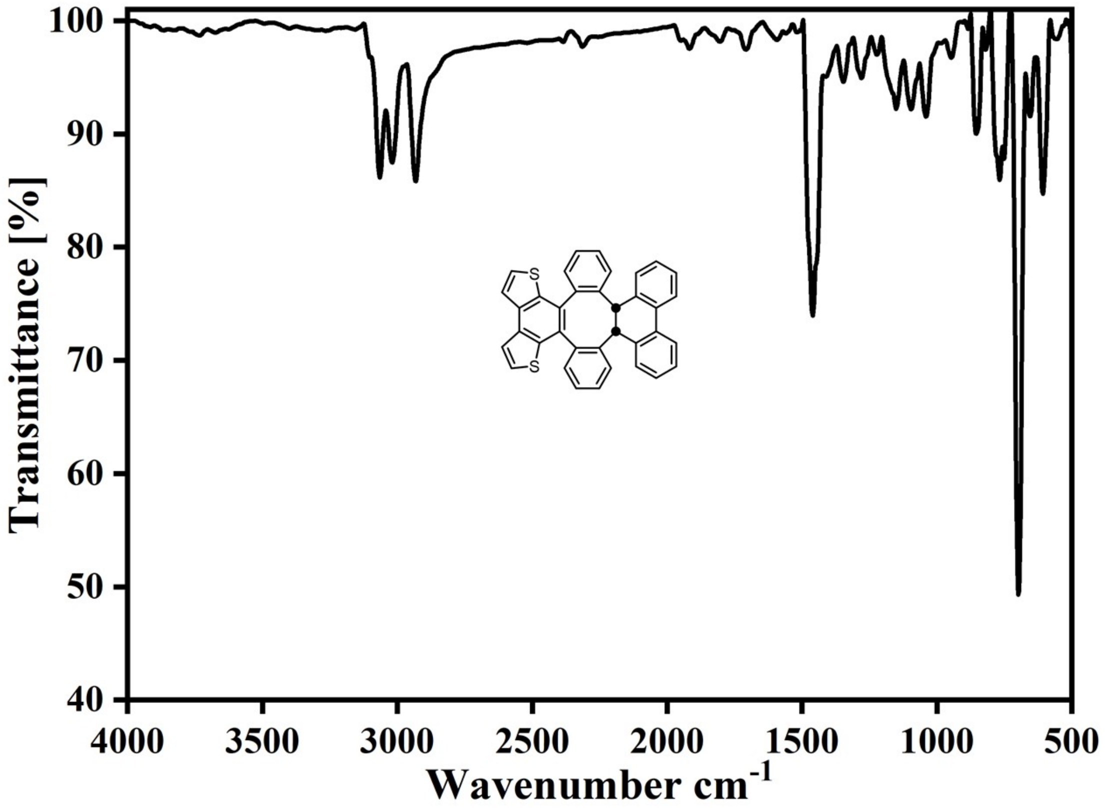

Figure S44. IR spectrum of **2d**.

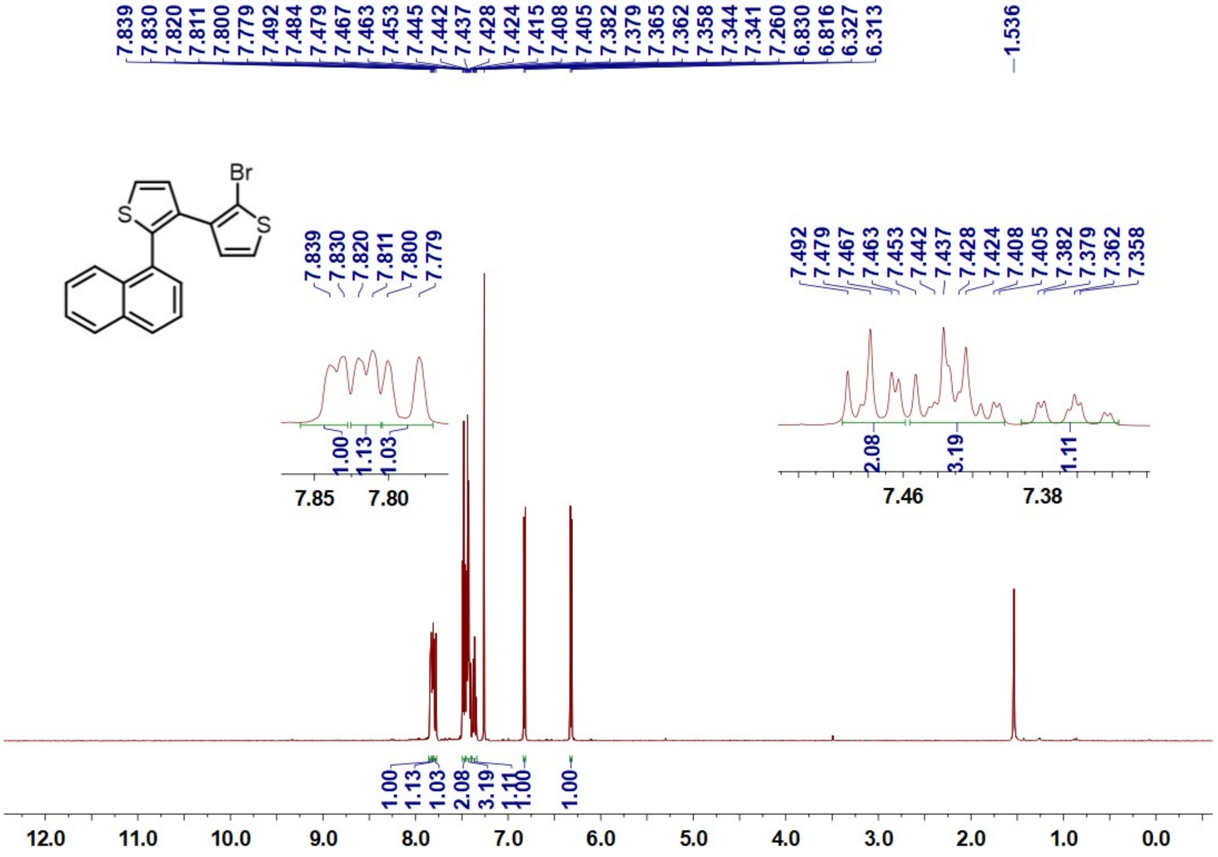

Figure S45. ^{1}H NMR (400 MHz, $CDCl_3$) spectrum of **9.**

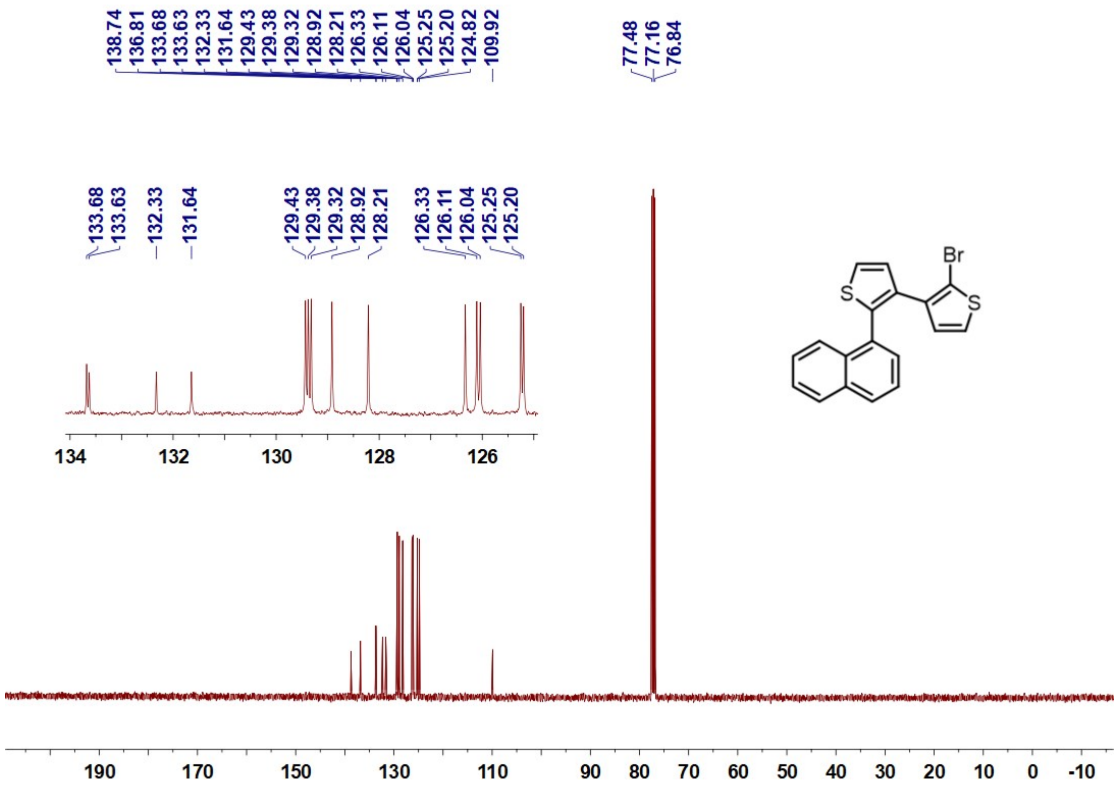

Figure S46. ^{13}C NMR (100 MHz, $CDCl_3$) spectra of **9**.

National Center for Organic Mass Spectrometry in Shanghai
Shanghai Institute of Organic Chemistry
Chinese Academic of Sciences
High Resolution MS DATA REPORT

Instrument: Thermo Fisher Scientific LTQ FTICR-MS

Card Serial Number : D20213177

Sample Serial Number: sxl-2-24-col-pp

Operator : DONG Date: 2021/06/09

Operation Mode: DART POSITIVE

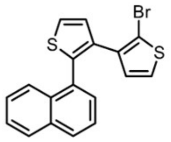

Elemental composition search on mass 370.9557

m/z= 365.9557-375.9557

m/z	Theo. Mass	Delta (ppm)	RDB equiv.	Composition
370.9557	370.9557	-0.01	20.5	$C_{19}O_4N_2FS$
	370.9557	0.11	2.0	$C_7H_{10}O_3N_3BrF_4S$
	370.9557	0.16	1.5	$C_{10}H_{13}BrF_5S_2$
	370.9556	0.27	9.0	$C_{13}H_{11}N_3BrFS_2$
	370.9558	-0.27	12.5	$C_{18}H_{12}BrS_2$
	370.9559	-0.43	5.5	$C_{12}H_{11}O_3BrF_3S$
	370.9555	0.58	16.5	$C_{17}H_2ON_2F_3S_2$
	370.9559	-0.59	10.5	$C_{12}H_8ON_4FS_4$
	370.9560	-0.70	3.0	$C_9H_{10}ONF_5S_4$
	370.9560	-0.74	3.5	$C_6H_7O_4N_4F_4S_3$

Figure S47. HRMS-DART spectrum of **9**.

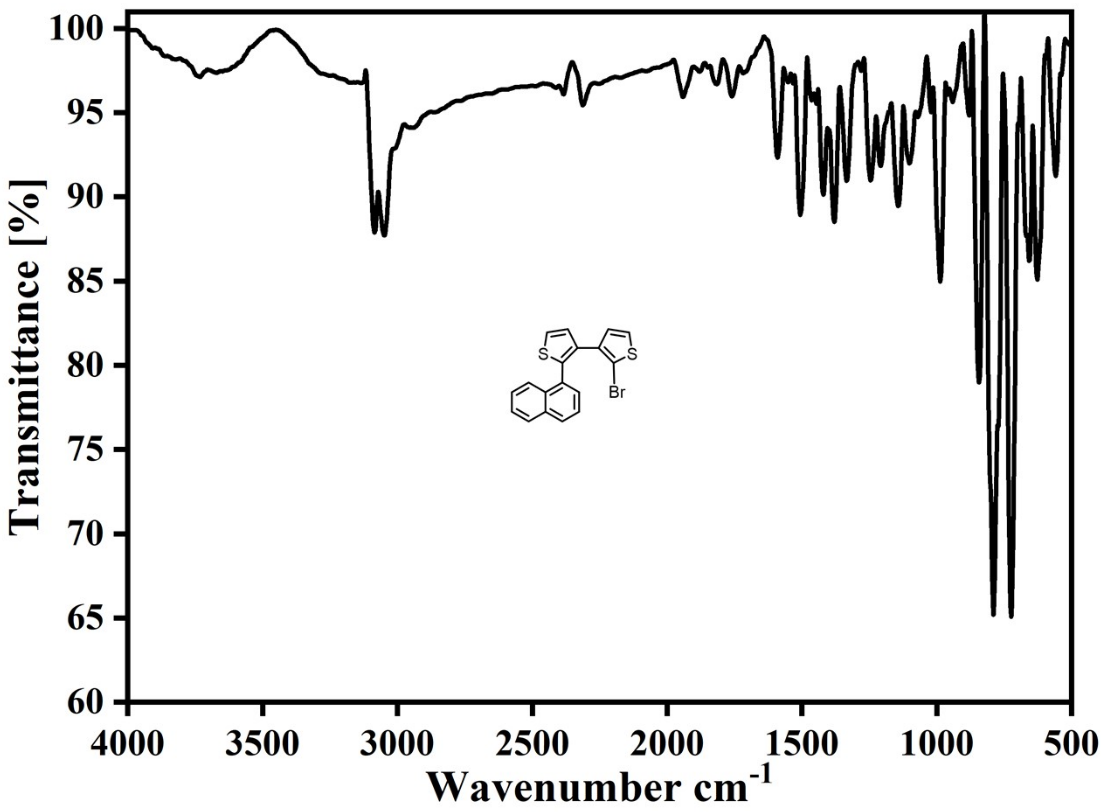

Figure S48. IR spectrum of **9**.

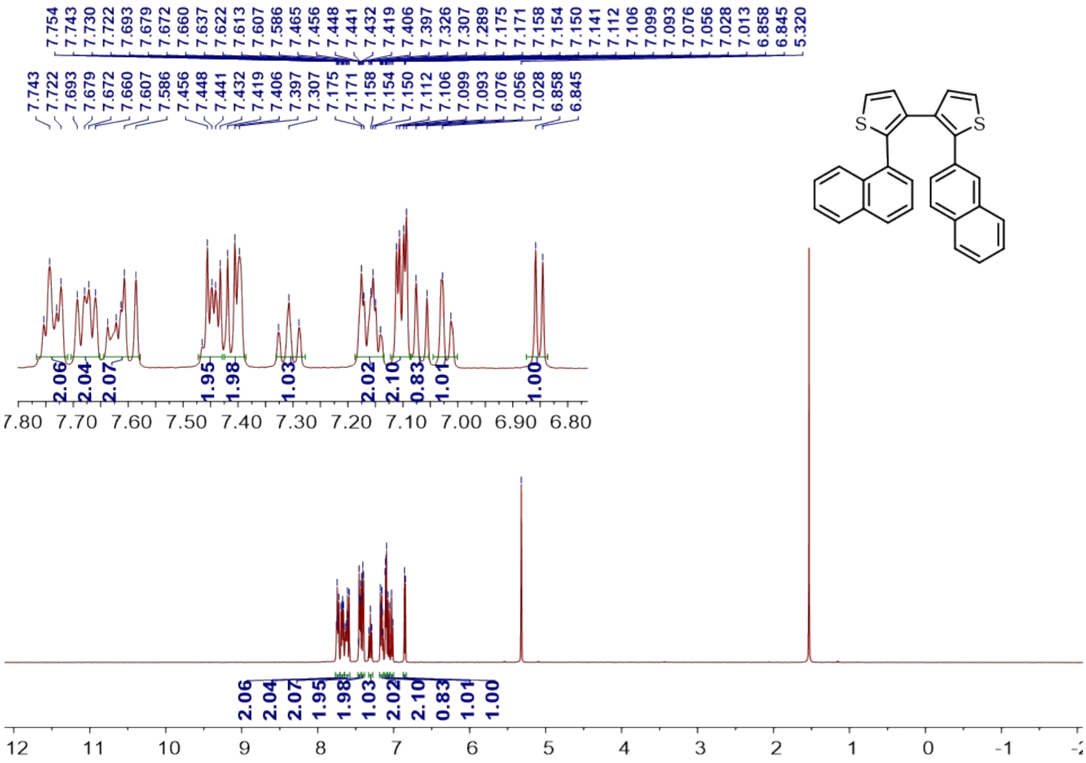

Figure S49. ^{1}H NMR (400 MHz, CD_2Cl_2) spectra of **3a**.

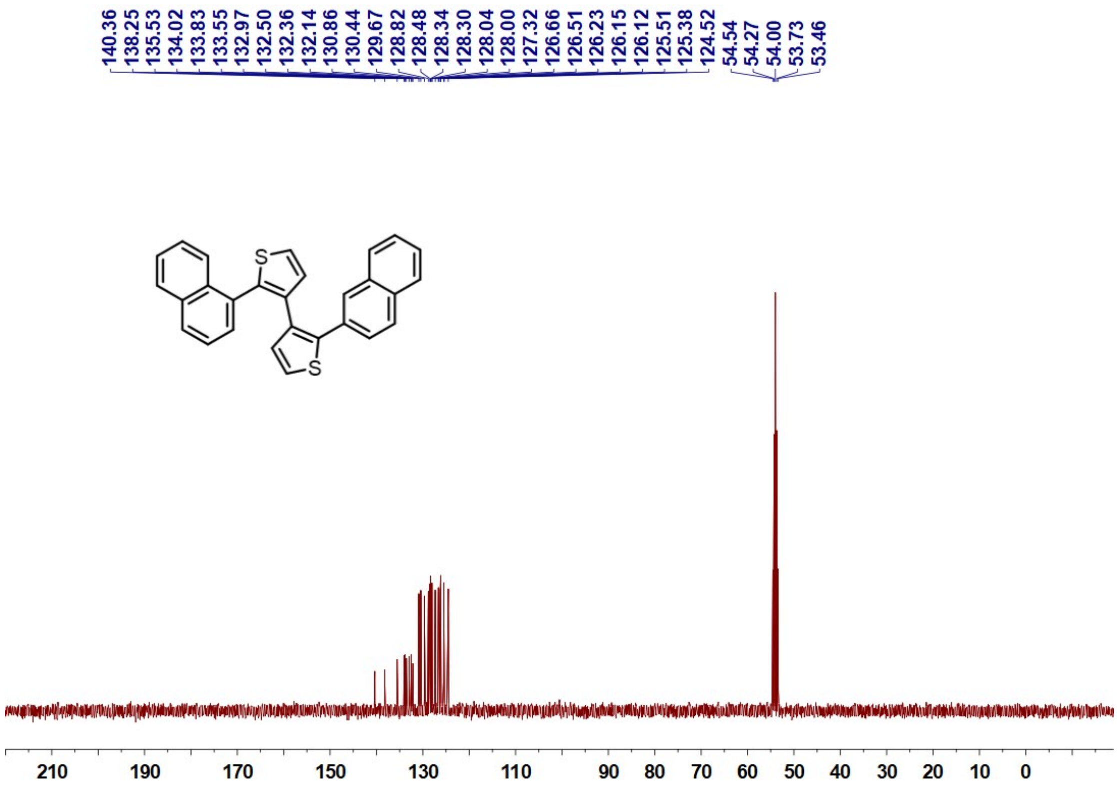

Figure S50. ^{13}C NMR (100 MHz, CD_2Cl_2) spectra of **3a.**

National Center for Organic Mass Spectrometry in Shanghai
Shanghai Institute of Organic Chemistry
Chinese Academic of Sciences
HIGH RESOLUTION MS REPORT

Instrument: JMS-S3000 MALDI-TOFMS

Sample Serial Number: SXL-2-35-COL-WS

Operator: Zhang, Li

Date: 2021/04/29

Operation Mode: MALDI-Positive

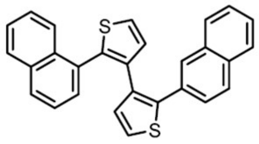

Elemental Composition Estimation

Parameters:

Mass	Tolerance	Electron Mode	Charge	DBE Range	Max Results
418.08427 ± 0.00209	5.0 ppm	Odd/Even	+1	-0.5 - 200.0	100

Elements

C	0 - 28	H	0 - 200	N	0 - 0	O	0 - 0	I	0 - 0	S	0 - 2	Si	0 - 3

Results:

#	Formula	Mass	DBE	Abs. Error (u)	Error (u)	Error (ppm)
1	C28 H18 S2	418.08444	20.0	0.00018	-0.00018	-0.42

Figure S51. HRMS-MALDI spectrum of **3a**.

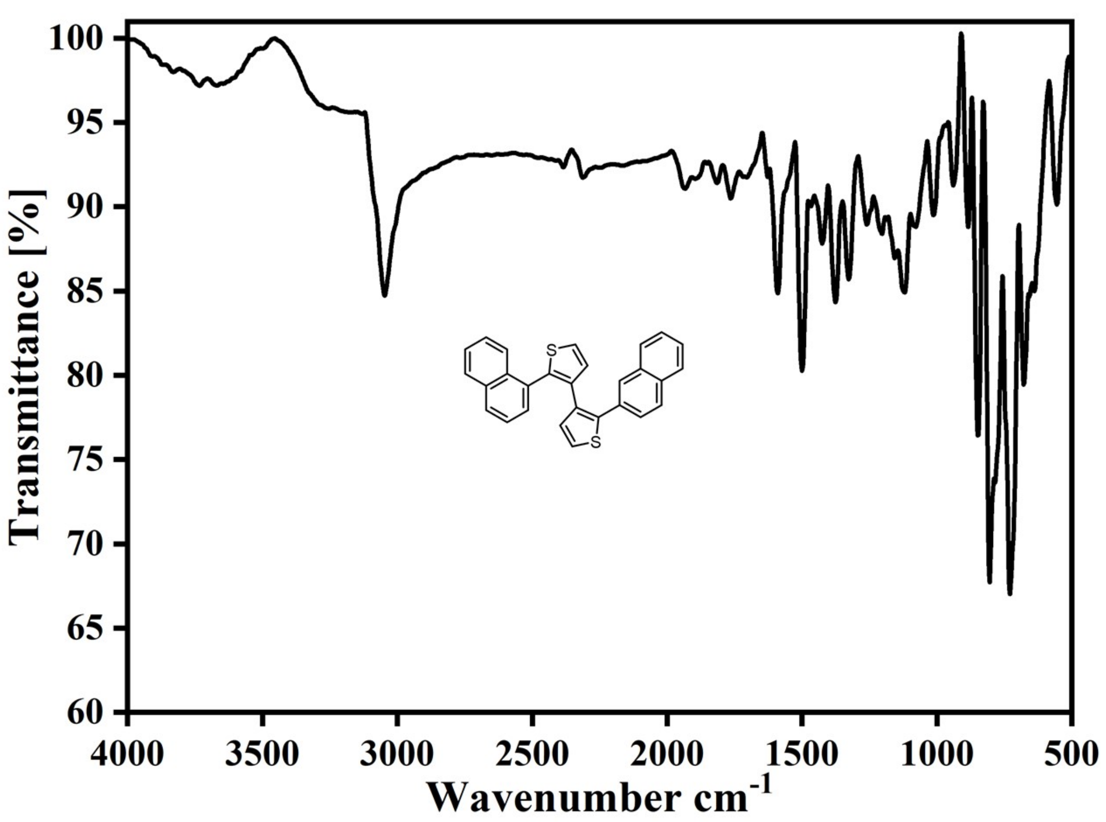

Figure S52. IR spectrum of **3a**.

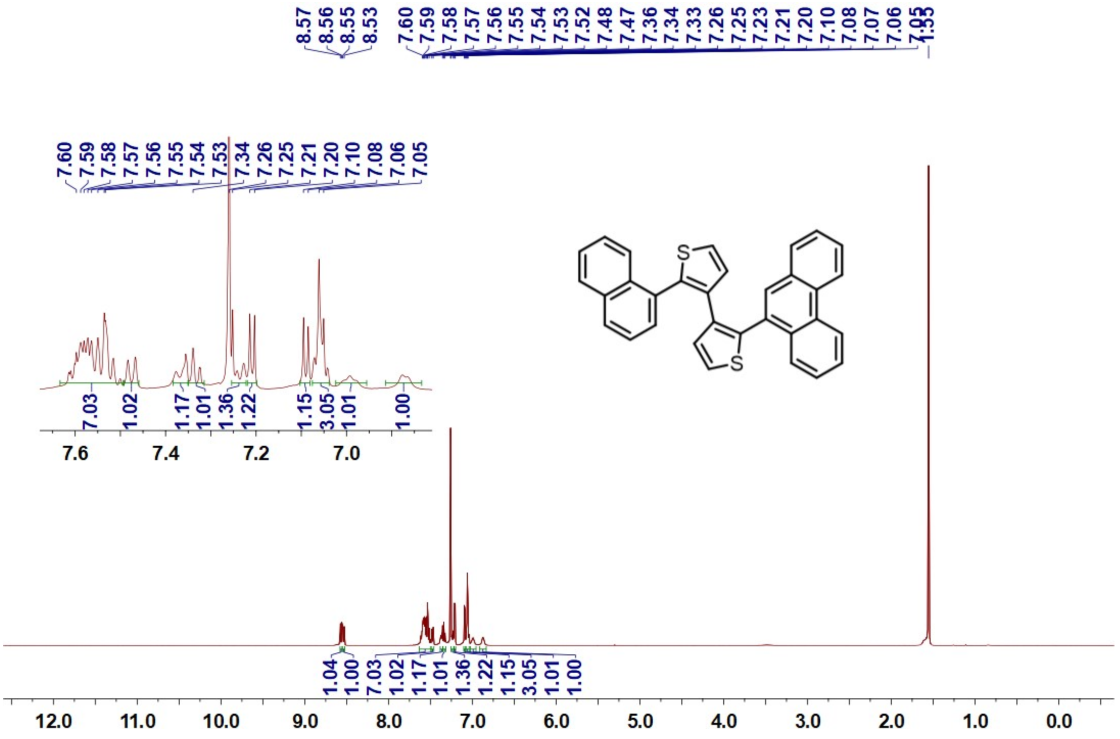

Figure S53. ^{1}H NMR (400 MHz, $CDCl_3$) spectra of **3b**.

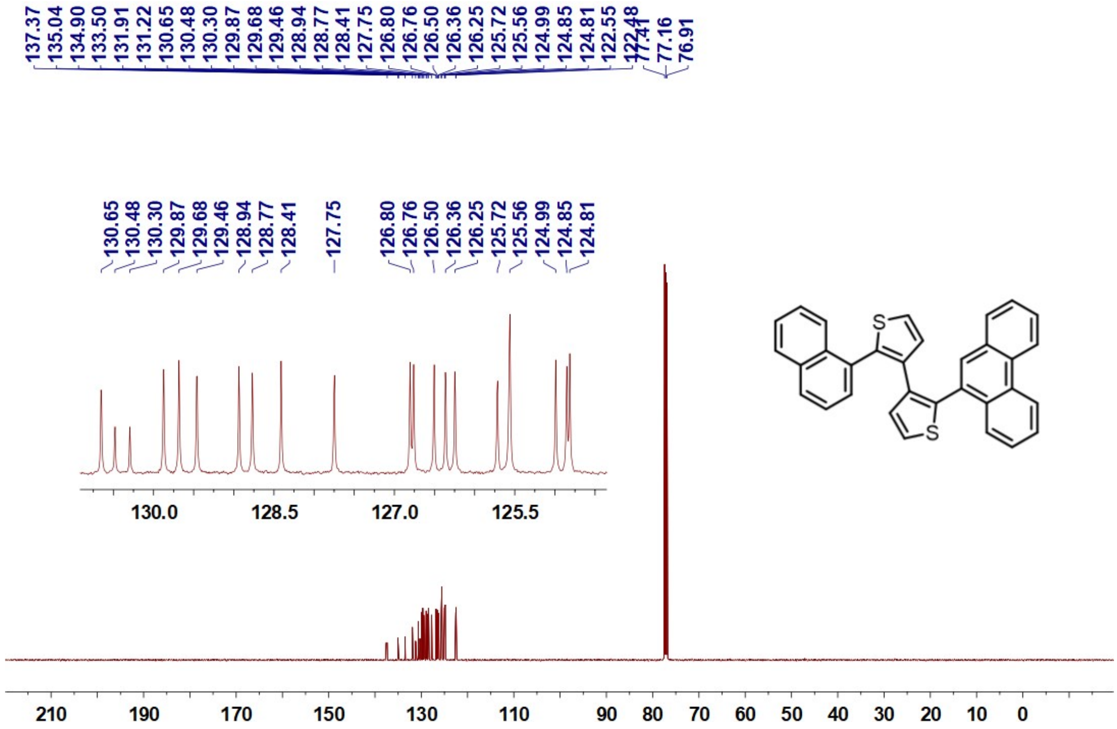

Figure S54. ^{13}C NMR (125 MHz, $CDCl_3$) spectra of **3b.**

National Center for Organic Mass Spectrometry in Shanghai
Shanghai Institute of Organic Chemistry
Chinese Academic of Sciences
HIGH RESOLUTION MS REPORT

Instrument: JMS-S3000 MALDI-TOFMS

Sample Serial Number: SXL-2-42-COL-PP-HR

Operator: Zhang, Li

Date: 2021/08/18

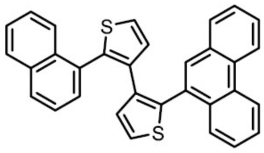

Operation Mode: MALDI-Positive

Elemental Composition Estimation

Parameters:

Mass	Tolerance	Electron Mode	Charge	DBE Range	Max Results
468.09832 ± 0.00234	5.0 ppm	Odd/Even	+1	-0.5 - 200.0	100

Elements

C 0 - 32 H 0 - 200 Br 0 - 0 Si 0 - 0 S 0 - 2

Results:

#	Formula	Mass	DBE	Abs. Error (u)	Error (u)	Error (ppm)
1	C32 H20 S2	468.10009	23.0	0.00178	-0.00178	-3.80

Figure S55. HRMS-MALDI spectrum of complex **3b**.

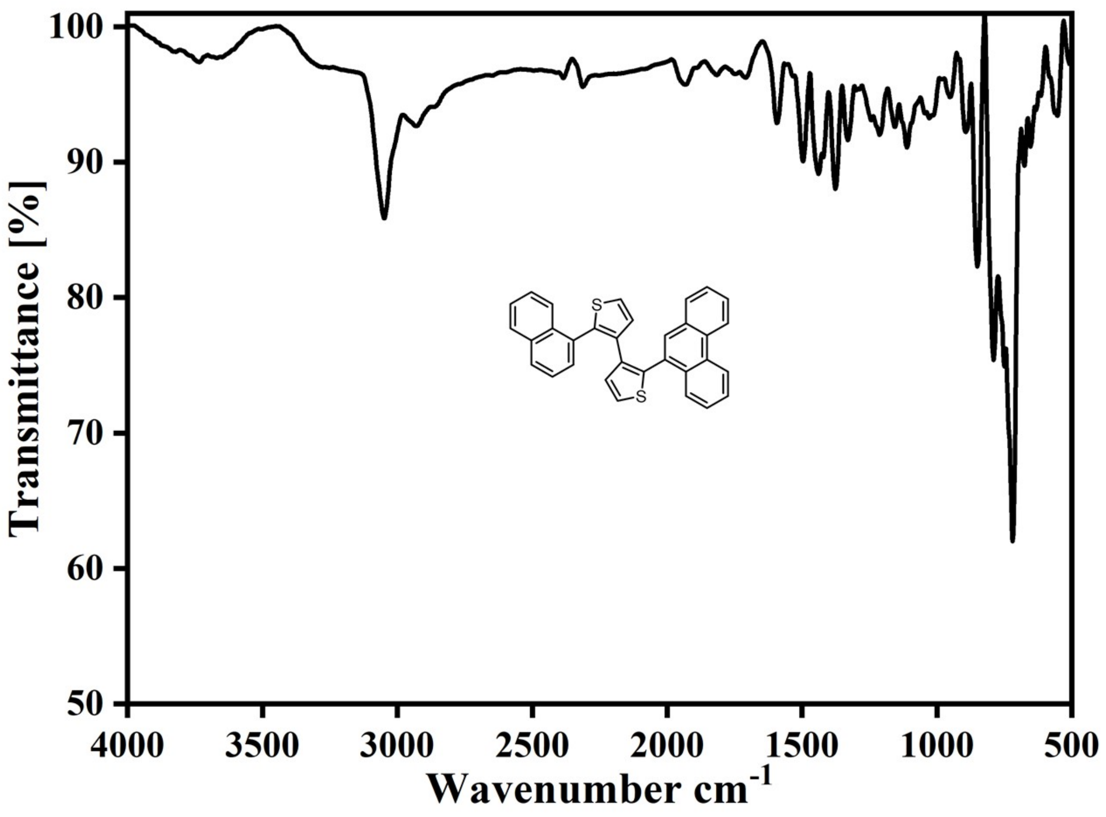

Figure S56. IR spectrum of **3b**.

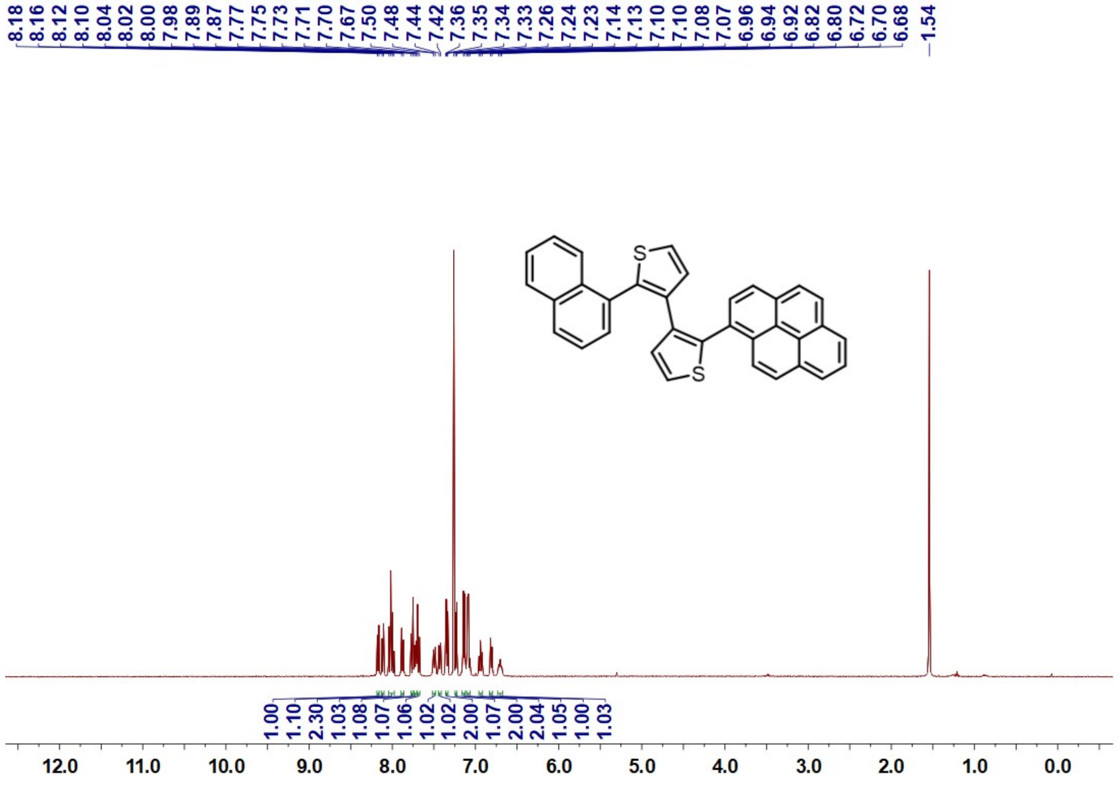

Figure S57. ^{1}H NMR (400 MHz, $CDCl_3$) spectra of **3c**.

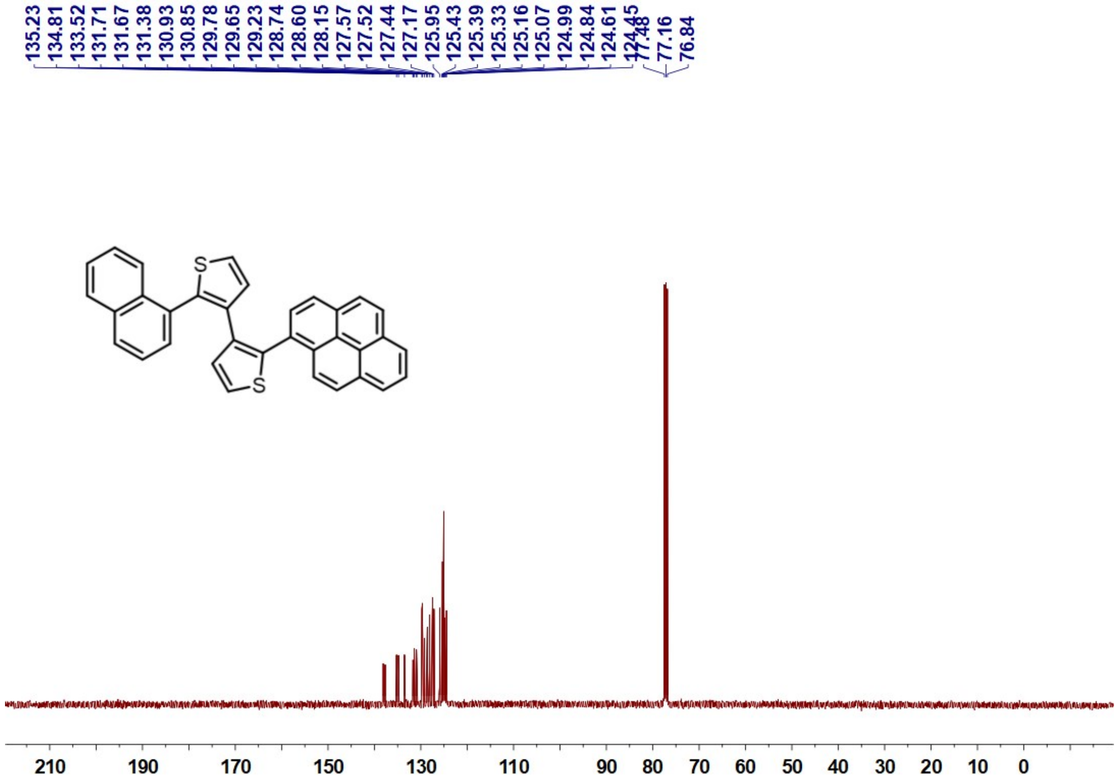

Figure S58. ^{13}C NMR (100 MHz, $CDCl_3$) spectra of **3c.**

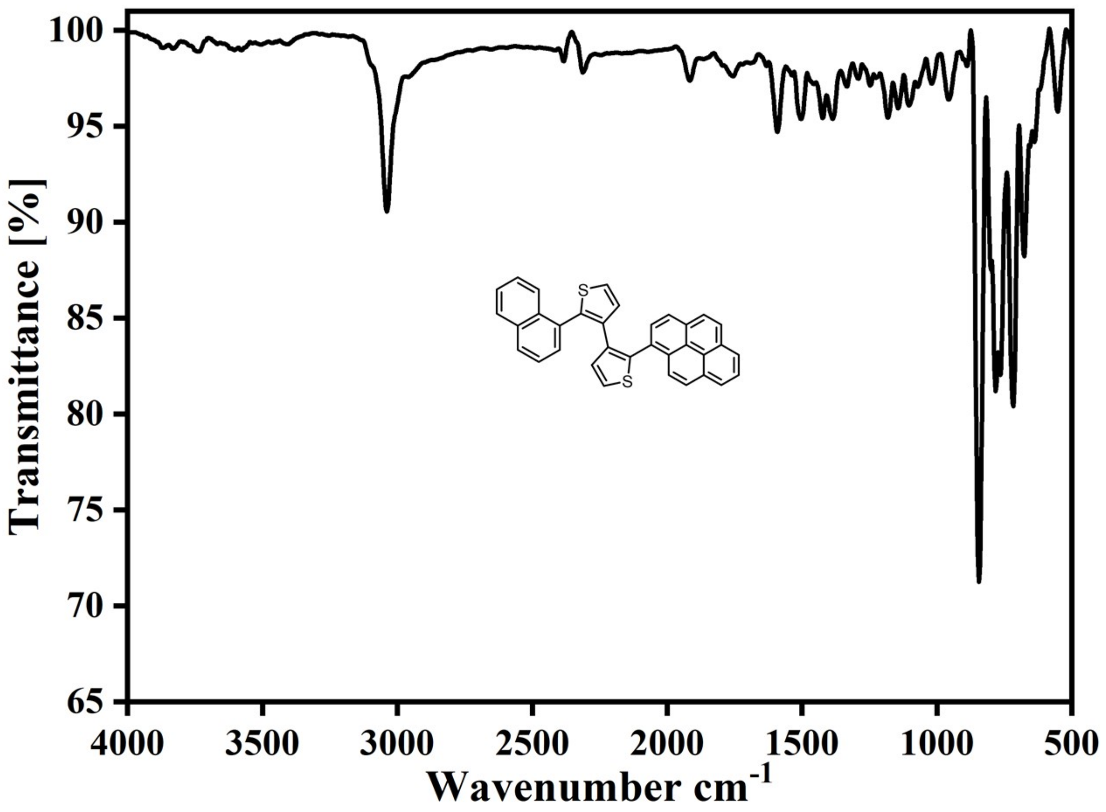

Figure S59. IR spectrum of **3c**

National Center for Organic Mass Spectrometry in Shanghai
Shanghai Institute of Organic Chemistry
Chinese Academic of Sciences
HIGH RESOLUTION MS REPORT

Instrument: JMS-S3000 MALDI-TOFMS

Sample Serial Number: SXL-2-22-COL-WS

Operator: Zhang, Li

Date: 2021/04/29

Operation Mode: MALDI-Positive

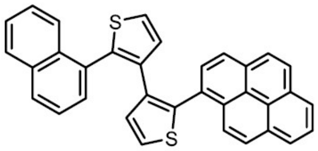

Elemental Composition Estimation

Parameters:

Mass	Tolerance	Electron Mode	Charge	DBE Range	Max Results
492.10099 ± 0.00246	5.0 ppm	Odd/Even	+1	-0.5 - 200.0	100

Elements

C	H	N	O	I	S	Si
0 - 34	0 - 200	0 - 0	0 - 0	0 - 0	0 - 2	0 - 3

Results:

#	Formula	Mass	DBE	Abs. Error (u)	Error (u)	Error (ppm)
1	C34 H20 S2	492.10009	25.0	0.00090	0.00090	1.83

Figure S60. HRMS-MALDI spectrum of **3c**.

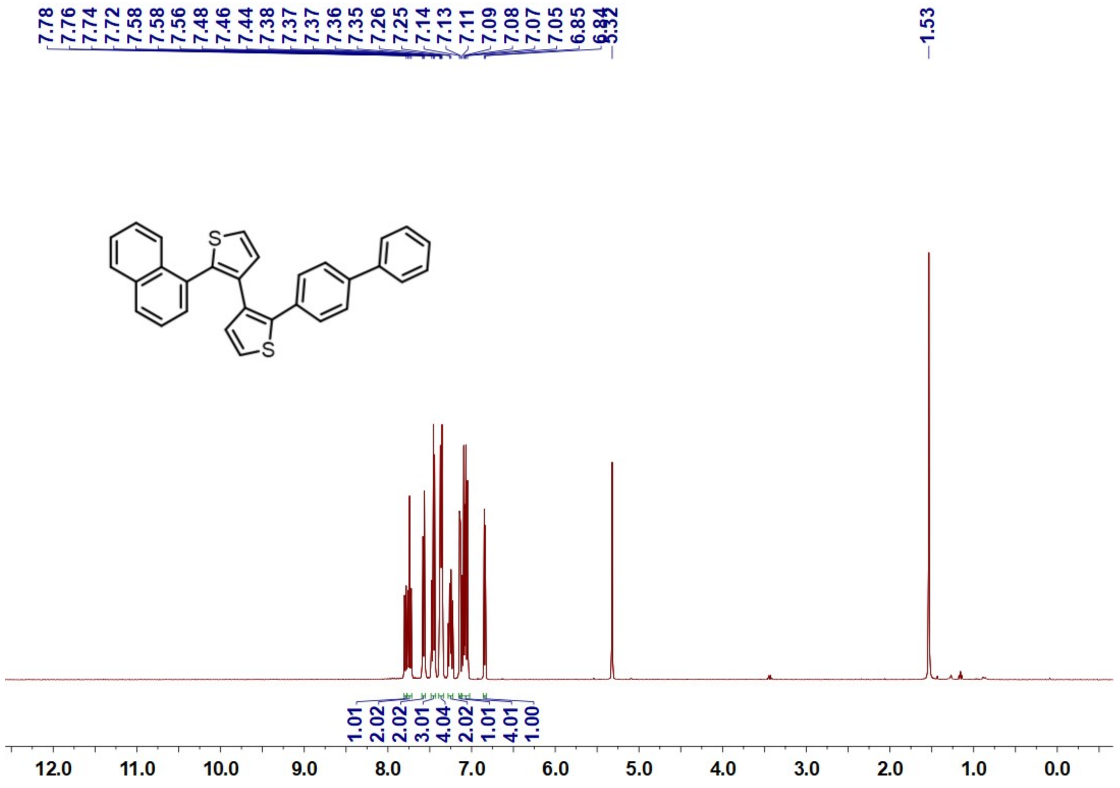

Figure S61. ^{1}H NMR (400 MHz, CD_2Cl_2) spectrum of **3d**.

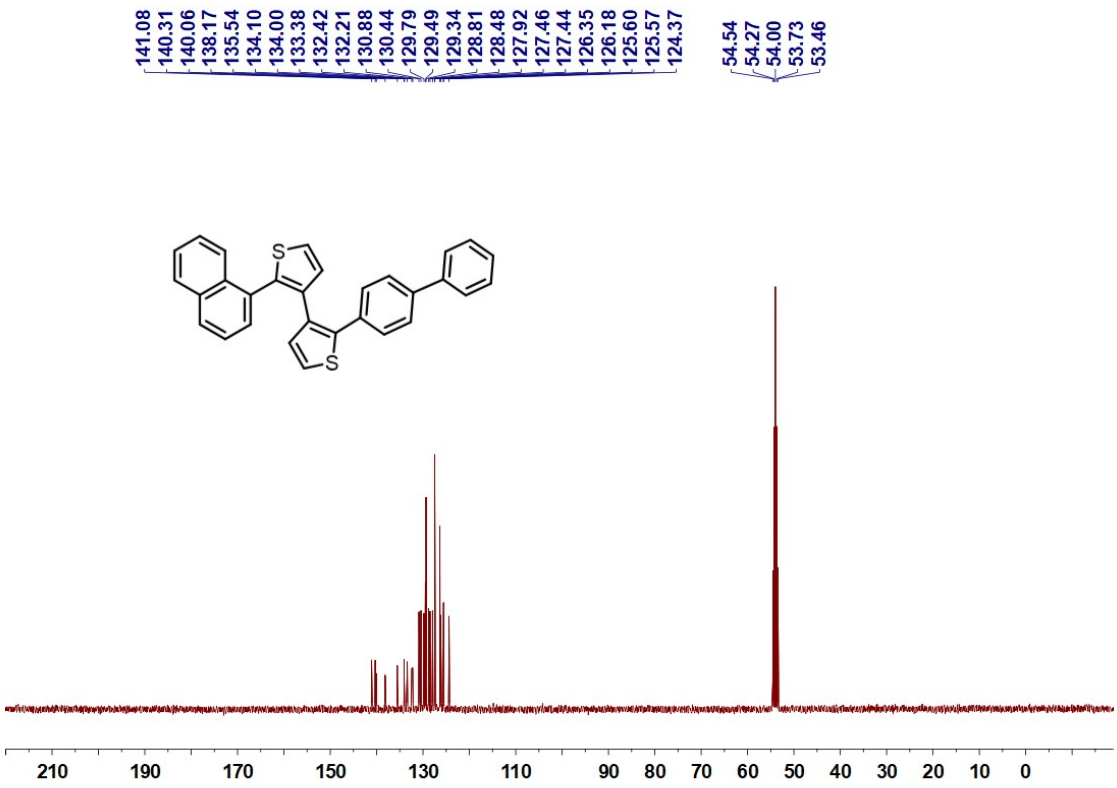

Figure S62. ^{13}C NMR (100 MHz, CD_2Cl_2) spectrum of **3d**.

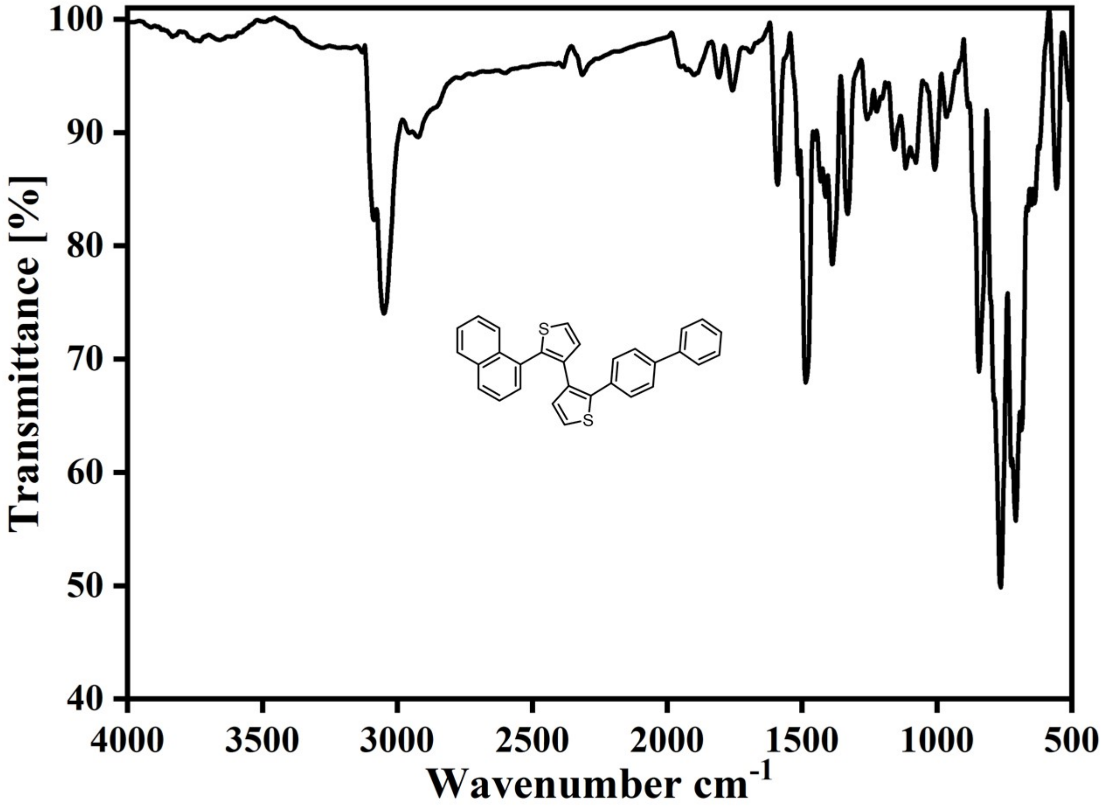

Figure S63. IR spectrum of **3d**.

National Center for Organic Mass Spectrometry in Shanghai
Shanghai Institute of Organic Chemistry
Chinese Academic of Sciences
HIGH RESOLUTION MS REPORT

Instrument: JMS-S3000 MALDI-TOFMS

Sample Serial Number: SXL-2-37-COL-PP-HR

Operator: Zhang, Li

Date: 2021/08/18

Operation Mode: MALDI-Positive

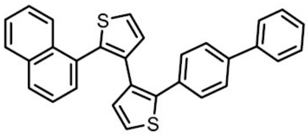

Elemental Composition Estimation

Parameters:

Mass	Tolerance	Electron Mode	Charge	DBE Range	Max Results
444.10061 ± 0.00222	5.0 ppm	Odd/Even	+1	-0.5 - 200.0	100

Elements

C	0 - 30	H	0 - 200	Br	0 - 0	Si	0 - 0	S	0 - 2

Results:

#	Formula	Mass	DBE	Abs. Error (u)	Error (u)	Error (ppm)
1	C30 H20 S2	444.10009	21.0	0.00051	0.00051	1.15

Figure S64. HRMS-MALDI spectrum of **3d**.

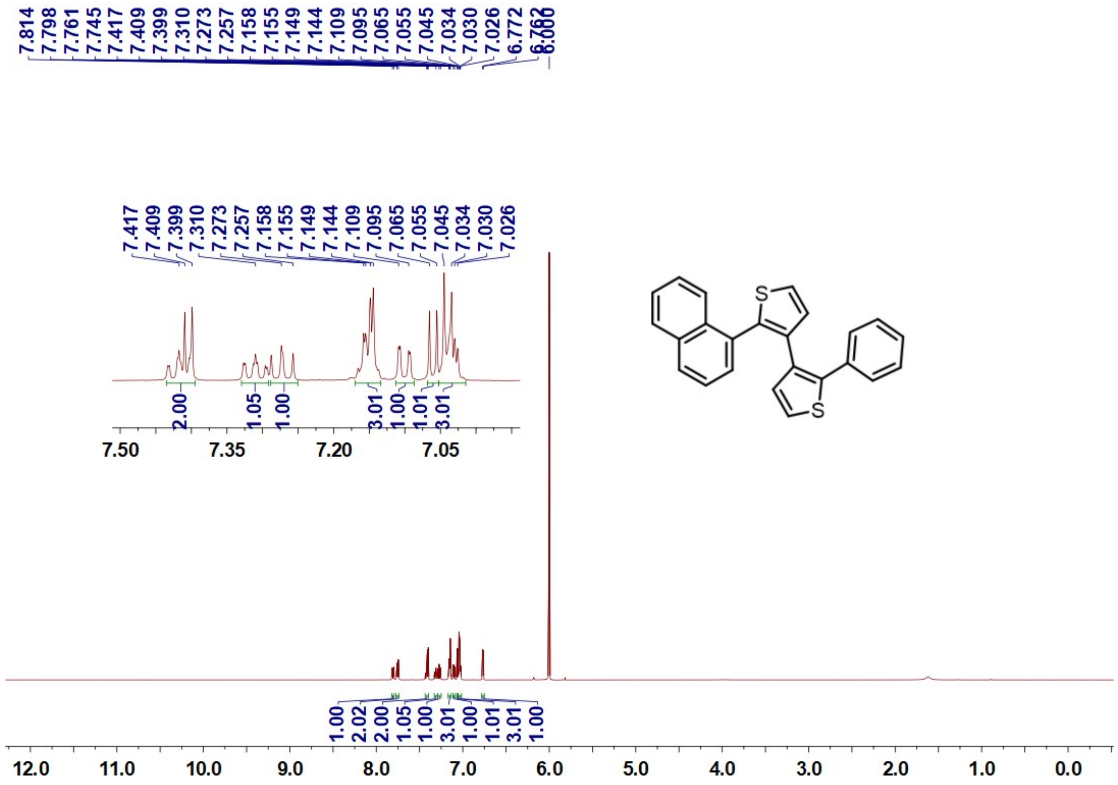

Figure S65. ^{1}H NMR (400 MHz, CD_2Cl_4) spectrum of **3e**.

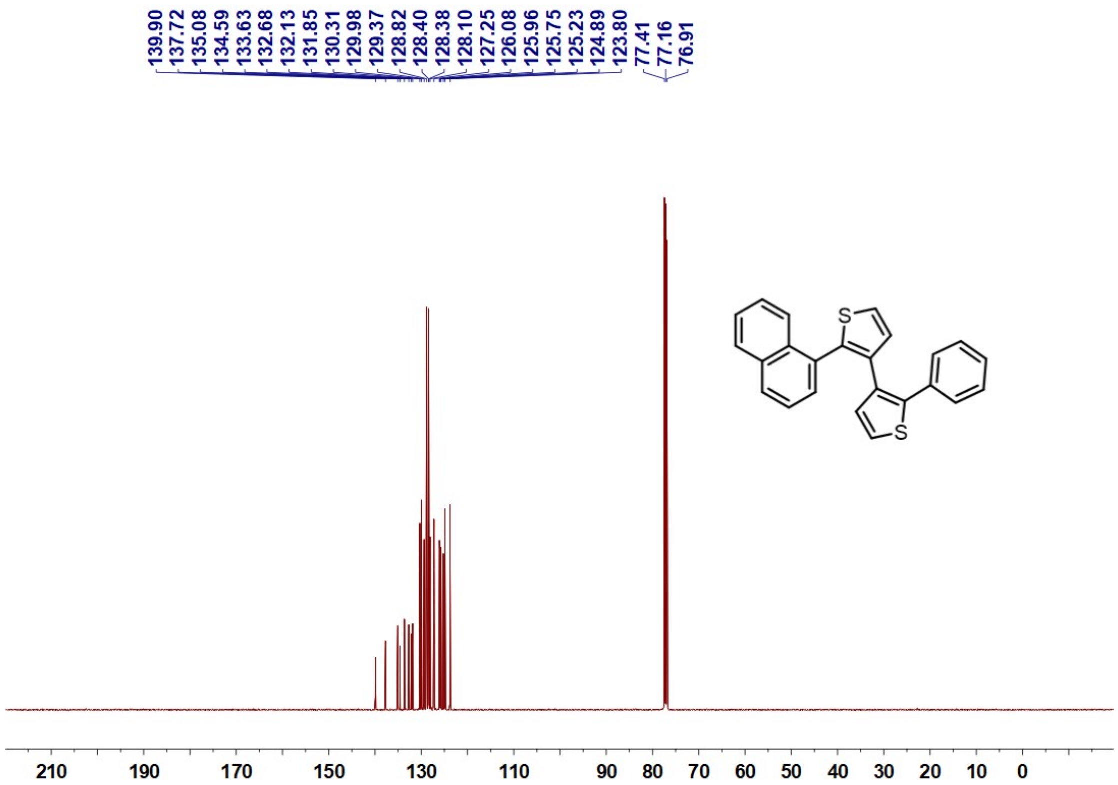

Figure S66. ^{13}C NMR (100 MHz, $CDCl_3$) spectrum of **3e**.

National Center for Organic Mass Spectrometry in Shanghai
Shanghai Institute of Organic Chemistry
Chinese Academic of Sciences
HIGH RESOLUTION MS REPORT

Instrument: JMS-S3000 MALDI-TOFMS

Sample Serial Number: SXL-3-64

Operator: Zhang, Li

Date: 2021/12/01

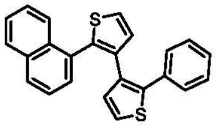

Operation Mode: MALDI-Positive

Elemental Composition Estimation

Parameters:

Mass	Tolerance	Electron Mode	Charge	DBE Range	Max Results
368.06696 ± 0.00184	5.0 ppm	Odd/Even	+1	-0.5 - 200.0	100

Elements

C 0 - 25 H 0 - 20 S 0 - 2

Results:

#	Formula	Mass	DBE	Abs. Error (u)	Error (u)	Error (ppm)
1	C24 H16 S2	368.06879	17.0	0.00183	-0.00183	-4.97

Figure S67. HRMS-MALDI spectrum of **3e**.

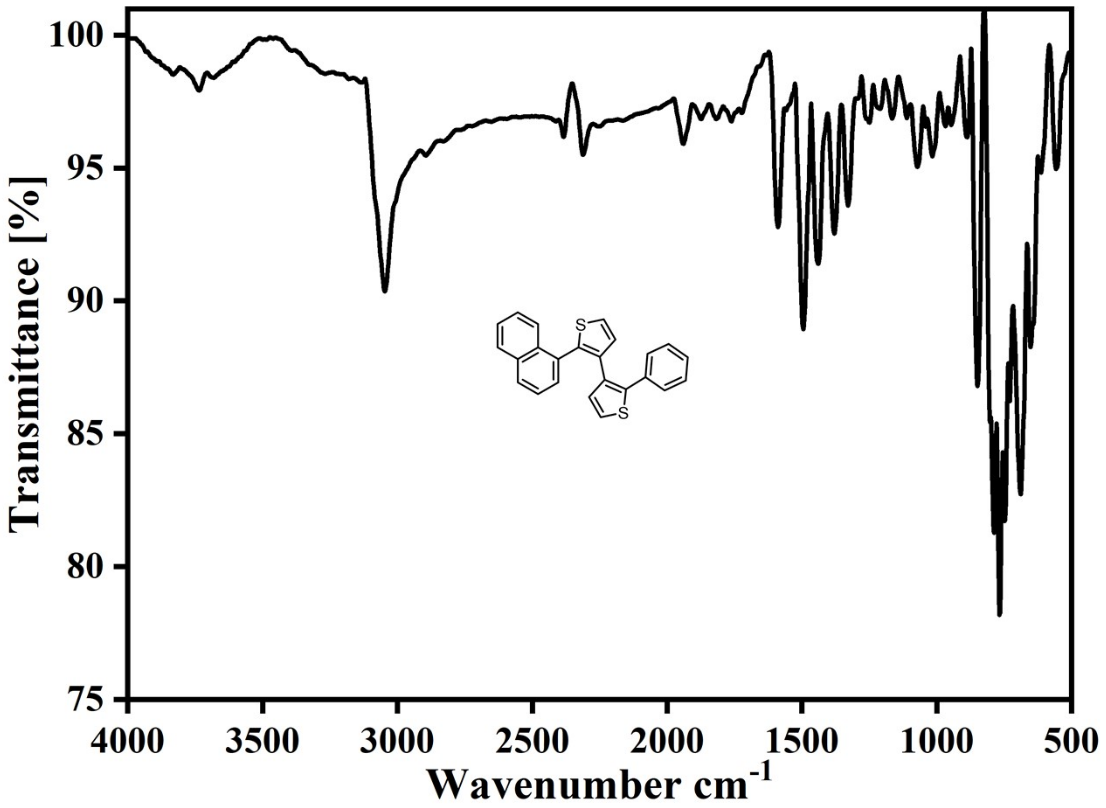

Figure S68. IR spectrum of **3e**.

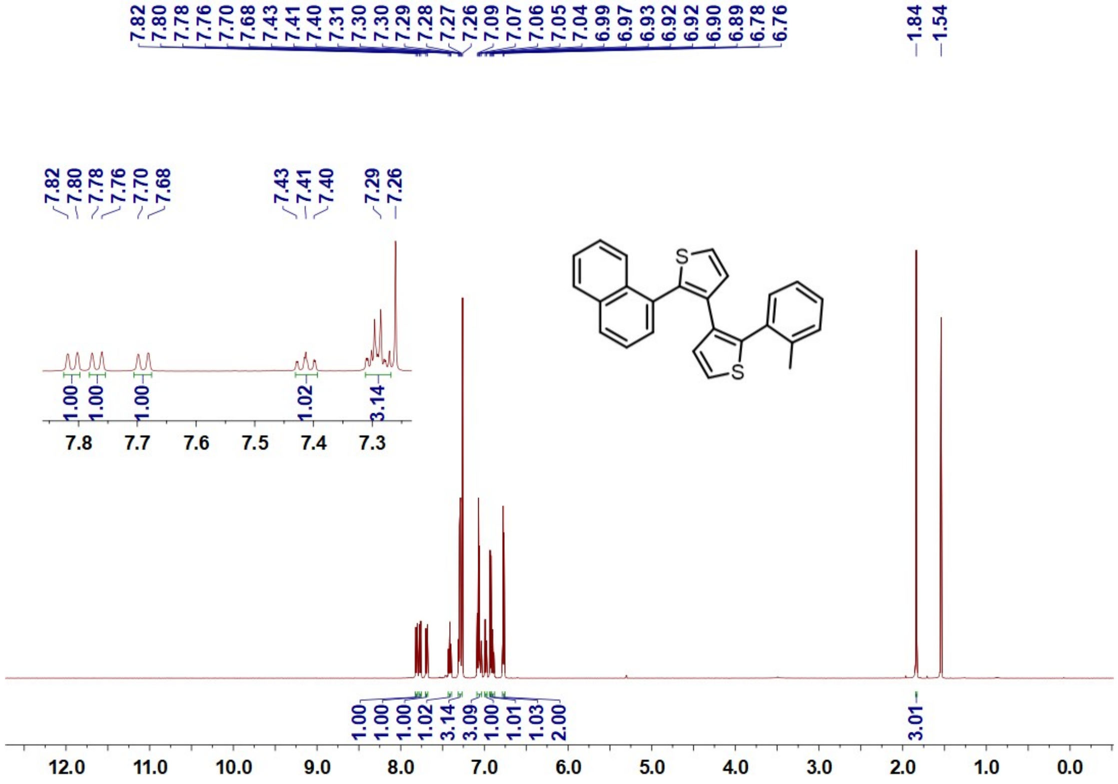

Figure S69. ^{1}H NMR (500 MHz, $CDCl_3$) spectrum of **3f**.

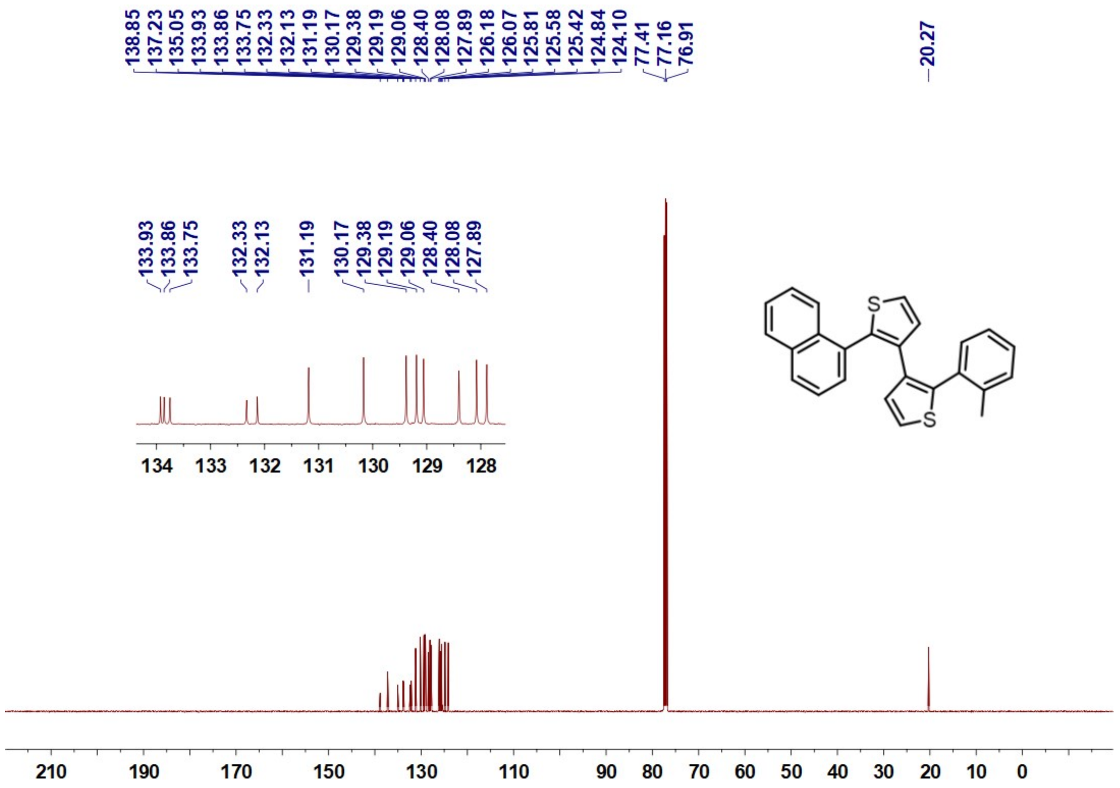

Figure S70. ^{13}C NMR (125 MHz, $CDCl_3$) spectrum of **3f**.

National Center for Organic Mass Spectrometry in Shanghai
Shanghai Institute of Organic Chemistry
Chinese Academic of Sciences
High Resolution MS DATA REPORT

Instrument: Thermo Fisher Scientific LTQ FTICR-MS

Card Serial Number : D20220029

Sample Serial Number: SXL-3-68

Operator : DONG Date: 2021/12/02

Operation Mode: DART POSITIVE

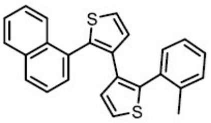

Elemental composition search on mass 383.0920

m/z= 378.0920-388.0920

m/z	Theo. Mass	Delta (ppm)	RDB equiv.	Composition
383.0920	383.0920	0.00	16.5	$C_{24}H_{19}OSSi$
	383.0923	-0.60	16.5	$C_{25}H_{19}S_2$
	383.0916	1.11	7.5	$C_{17}H_{23}O_2N_2S_3$
	383.0914	1.67	17.5	$C_{24}H_{15}O_5$
	383.0914	1.70	7.5	$C_{16}H_{23}O_3N_2S_2Si$
	383.0927	-1.82	22.5	$C_{25}H_{11}ON_4$
	383.0932	-3.02	13.0	$C_{18}H_{17}O_5N_3Si$
	383.0907	3.51	17.0	$C_{22}H_{17}N_3SSi$
	383.0934	-3.59	3.0	$C_{11}H_{25}O_2N_5S_3Si$
	383.0934	-3.62	13.0	$C_{19}H_{17}O_4N_3S$

Figure S71. HRMS-DART spectrum of **3f**.

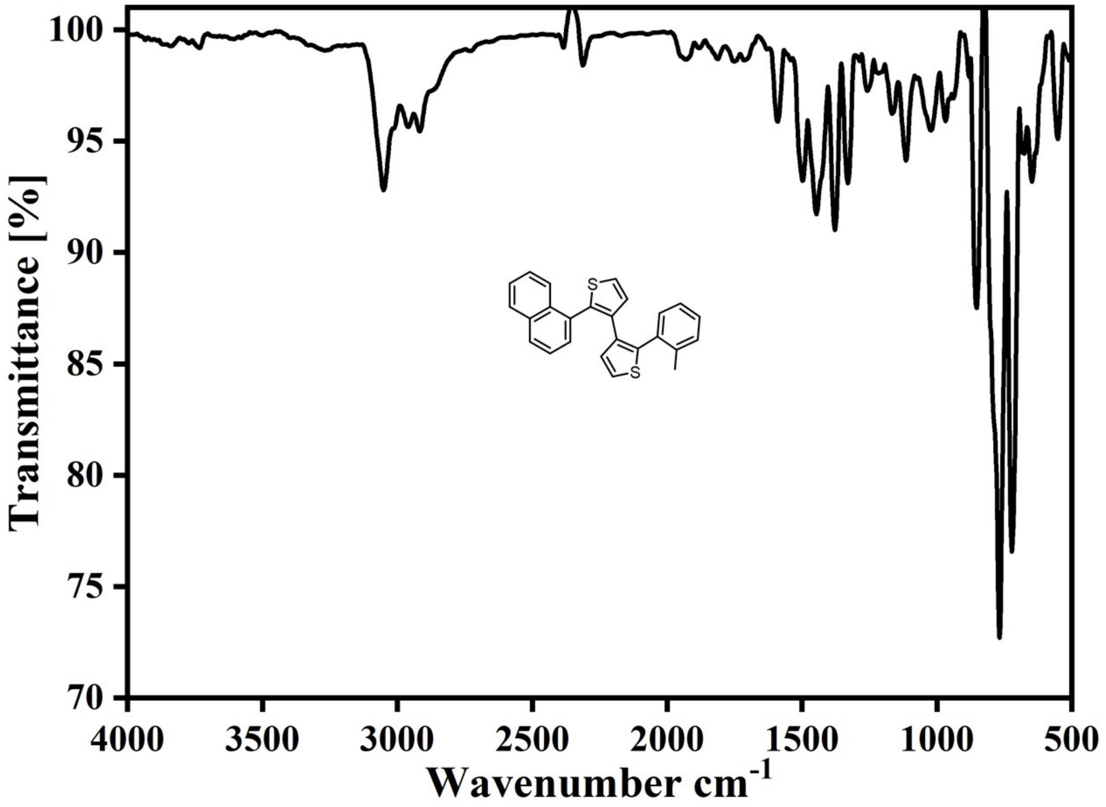

Figure S72. IR spectrum of **3f**.

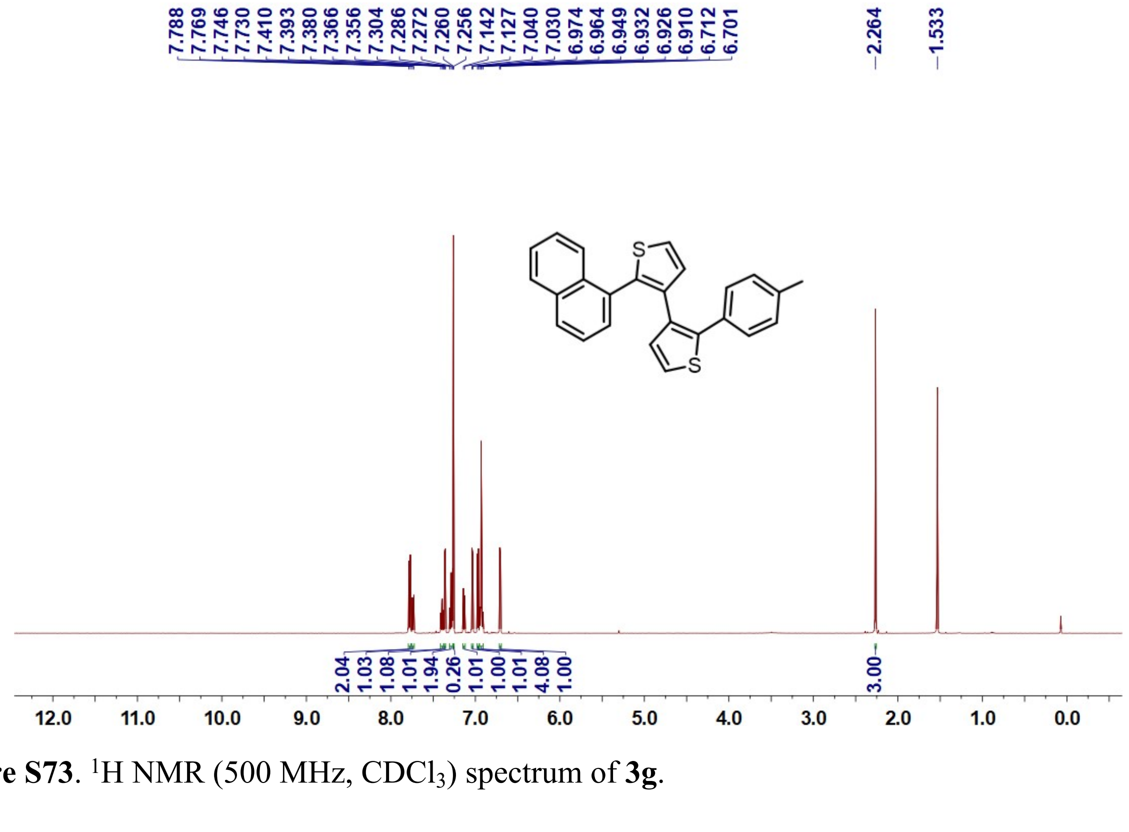

Figure S73. ^{1}H NMR (500 MHz, $CDCl_3$) spectrum of **3g**.

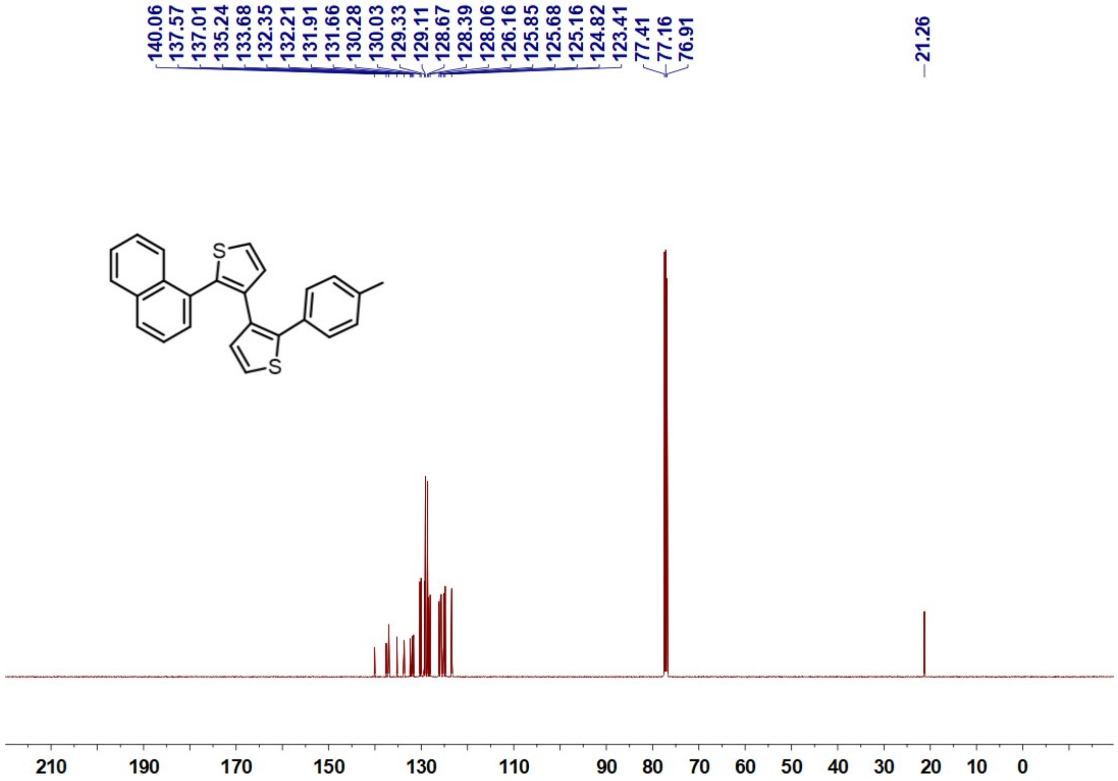

Figure S74. ^{13}C NMR (100 MHz, $CDCl_3$) spectrum of **3g**.

National Center for Organic Mass Spectrometry in Shanghai
Shanghai Institute of Organic Chemistry
Chinese Academic of Sciences
High Resolution MS DATA REPORT

Instrument: Thermo Fisher Scientific LTQ FTICR-MS

Card Serial Number : D20220027

Sample Serial Number: SXL-3-72

Operator : DONG Date: 2021/12/02

Operation Mode: DART POSITIVE

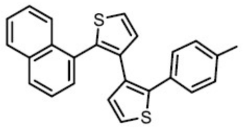

Elemental composition search on mass 383.0921

m/z= 378.0921-388.0921

m/z	Theo. Mass	Delta (ppm)	RDB equiv.	Composition
383.0921	383.0920	0.08	16.5	$C_{24}H_{19}OSSi$
	383.0923	-0.52	16.5	$C_{25}H_{19}S_2$
	383.0916	1.18	7.5	$C_{17}H_{23}O_2N_2S_3$
	383.0927	-1.74	22.5	$C_{25}H_{11}ON_4$
	383.0914	1.75	17.5	$C_{24}H_{15}O_5$
	383.0914	1.78	7.5	$C_{16}H_{23}O_3N_2S_2Si$
	383.0932	-2.95	13.0	$C_{18}H_{17}O_5N_3Si$
	383.0934	-3.51	3.0	$C_{11}H_{25}O_2N_5S_3Si$
	383.0934	-3.55	13.0	$C_{19}H_{17}O_4N_3S$
	383.0907	3.59	17.0	$C_{22}H_{17}N_3SSi$

Figure S75. HRMS-DART spectrum of **3g**.

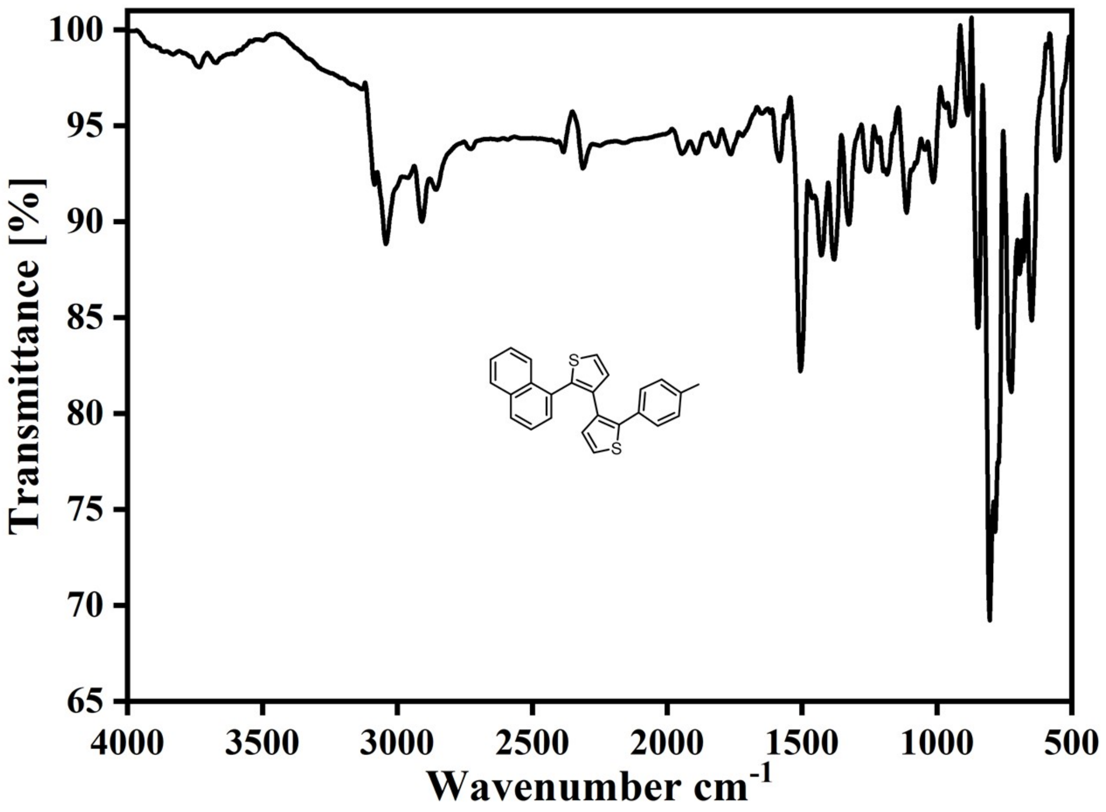

Figure S76. IR spectrum of **3g**.

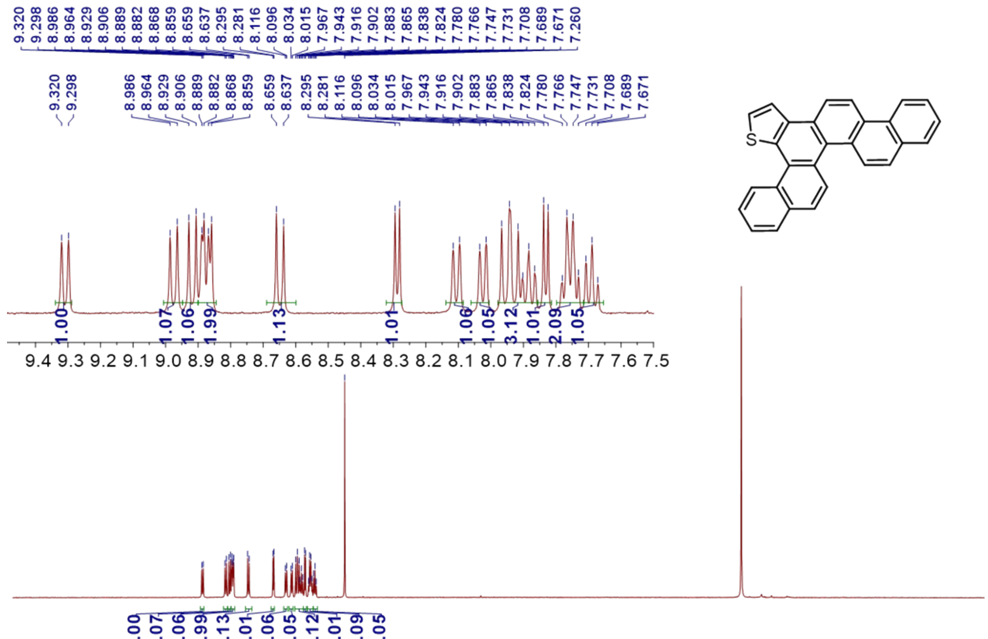

Figure S77. ^{1}H NMR (400 MHz, $CDCl_3$) spectrum of **4a**.

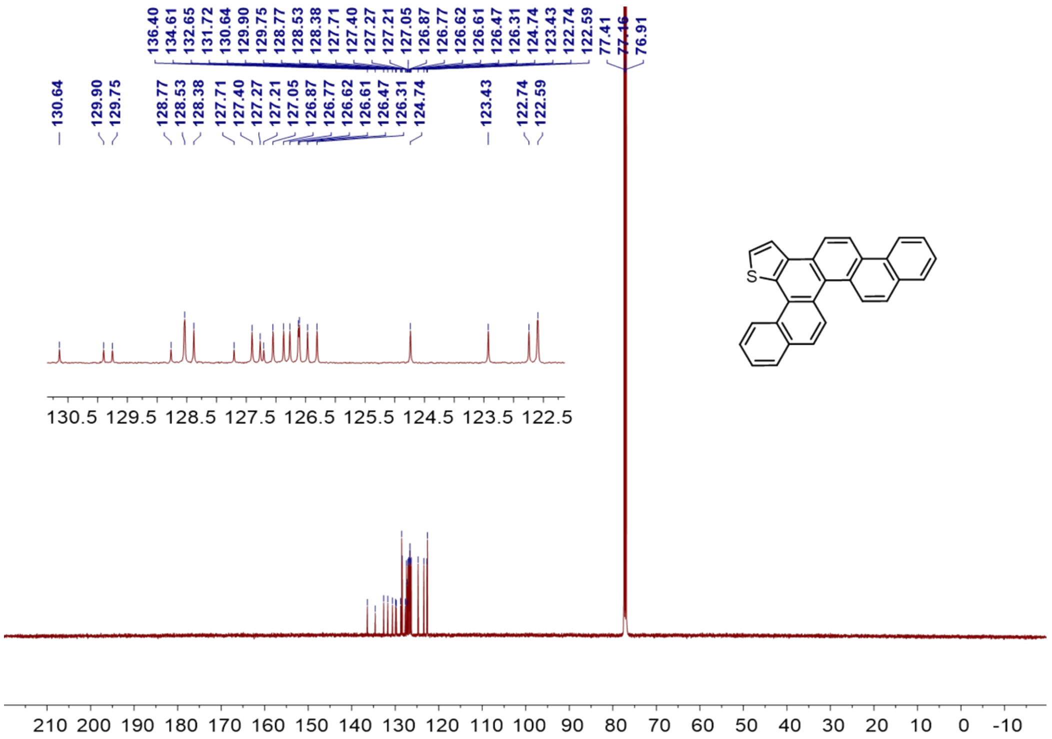

Figure S78. ^{13}C NMR (100 MHz, $CDCl_3$) spectrum of **4a**.

National Center for Organic Mass Spectrometry in Shanghai
Shanghai Institute of Organic Chemistry
Chinese Academic of Sciences
HIGH RESOLUTION MS REPORT

Instrument: JMS-S3000 MALDI-TOFMS

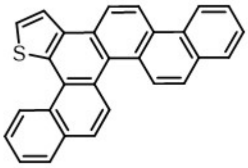

Sample Serial Number: SXL-2-45-COL-PP

Operator: Zhang, Li

Date: 2021/04/29

Operation Mode: MALDI-Positive

Elemental Composition Estimation

Parameters:

Mass	Tolerance	Electron Mode	Charge	DBE Range	Max Results
385.10430 ± 0.00193	5.0 ppm	Odd/Even	+1	-0.5 - 200.0	100

Elements

C	0 - 28	H	0 - 200	N	0 - 0	O	0 - 0	I	0 - 0	S	0 - 1	Si	0 - 3

Results:

#	Formula	Mass	DBE	Abs. Error (u)	Error (u)	Error (ppm)
1	C28 H17 S	385.10455	20.5	0.00024	-0.00024	-0.63

Figure S79. HRMS-MALDI spectrum of **4a**.

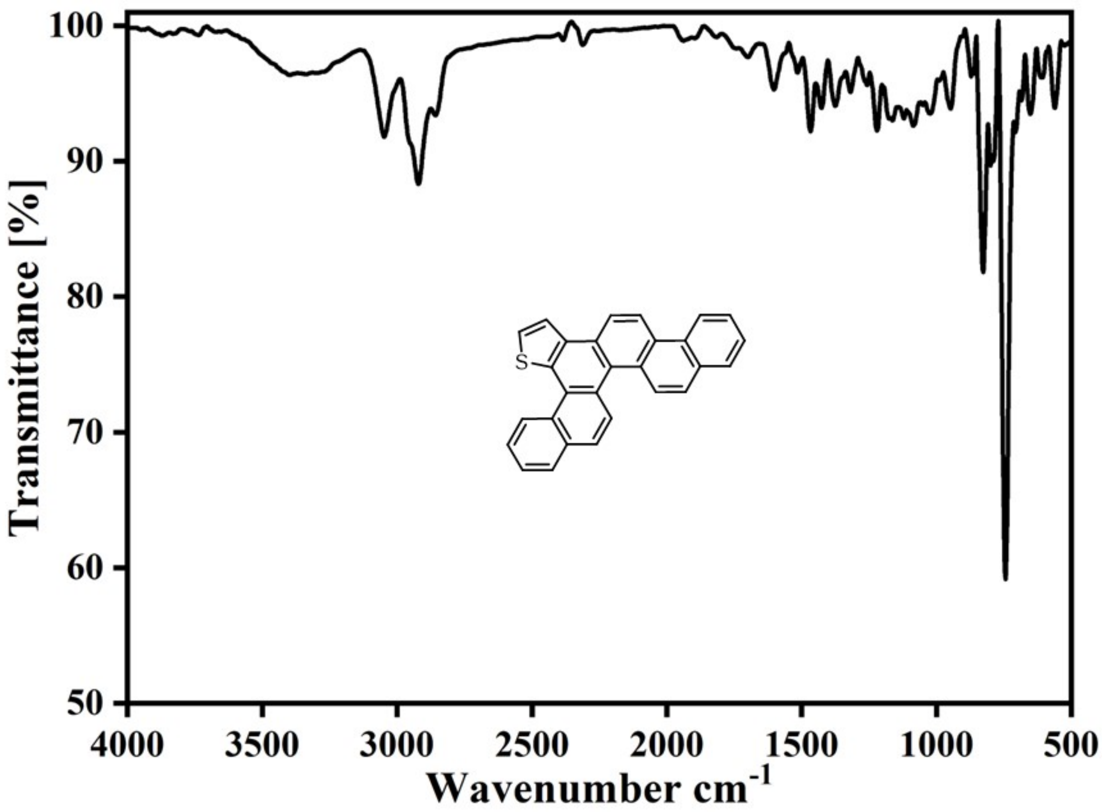

Figure S80. IR spectrum of **4a**.

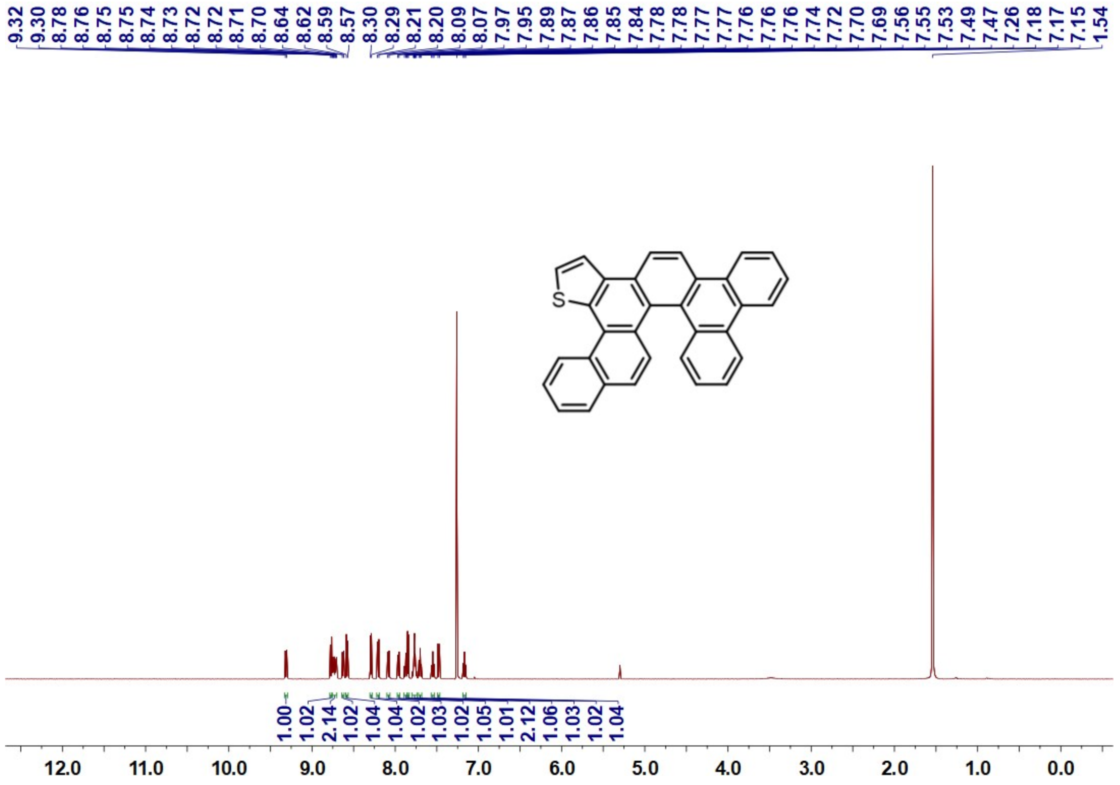

Figure S81. ^{1}H NMR (400 MHz, $CDCl_3$) spectrum of **4b**.

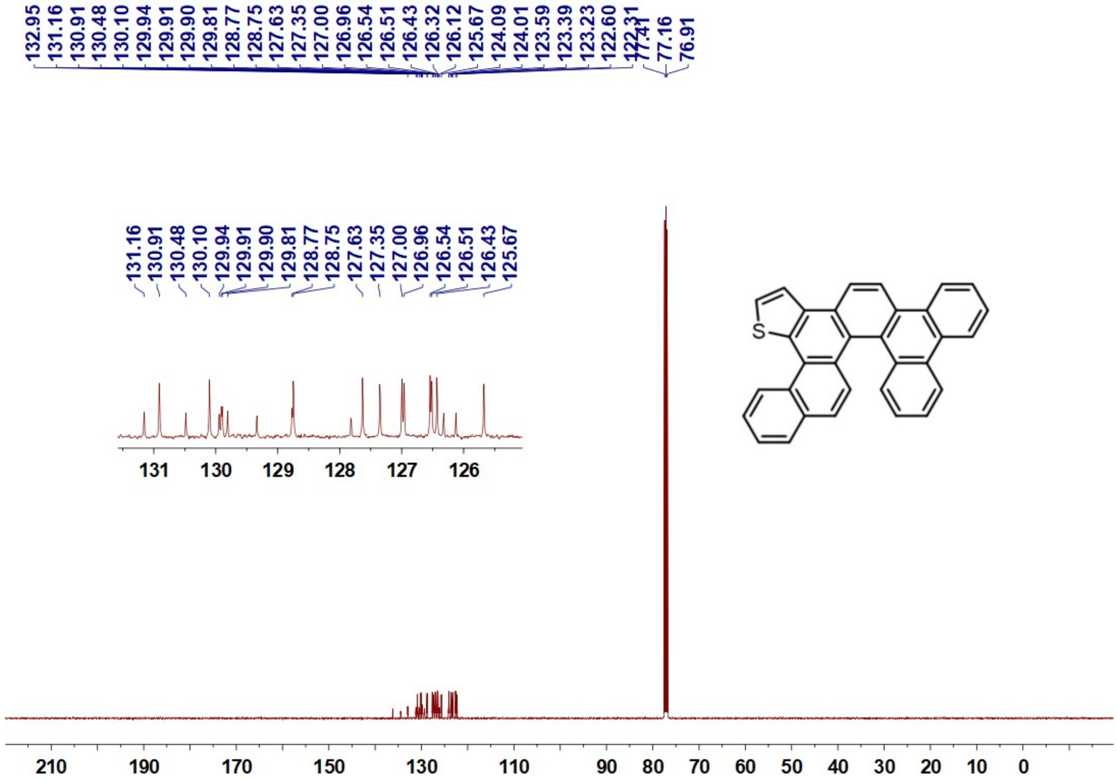

Figure S82. ^{13}C NMR (100 MHz, $CDCl_3$) spectrum of **4b**.

National Center for Organic Mass Spectrometry in Shanghai
Shanghai Institute of Organic Chemistry
Chinese Academic of Sciences
HIGH RESOLUTION MS REPORT

Instrument: JMS-S3000 MALDI-TOFMS

Sample Serial Number: SXL-2-50-COL-PP-HR

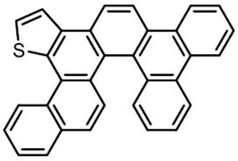

Operator: Zhang, Li

Date: 2021/08/18

Operation Mode: MALDI-Positive

Elemental Composition Estimation

Parameters:

Mass	Tolerance	Electron Mode	Charge	DBE Range	Max Results
434.11257 ± 0.00217	5.0 ppm	Odd/Even	+1	-0.5 - 200.0	100

Elements

C 0 - 32 H 0 - 200 Br 0 - 0 Si 0 - 0 S 0 - 1

Results:

#	Formula	Mass	DBE	Abs. Error (u)	Error (u)	Error (ppm)
1	C32 H18 S	434.11237	24.0	0.00020	0.00020	0.46

Figure S83. HRMS-MALDI spectrum of **4b**.

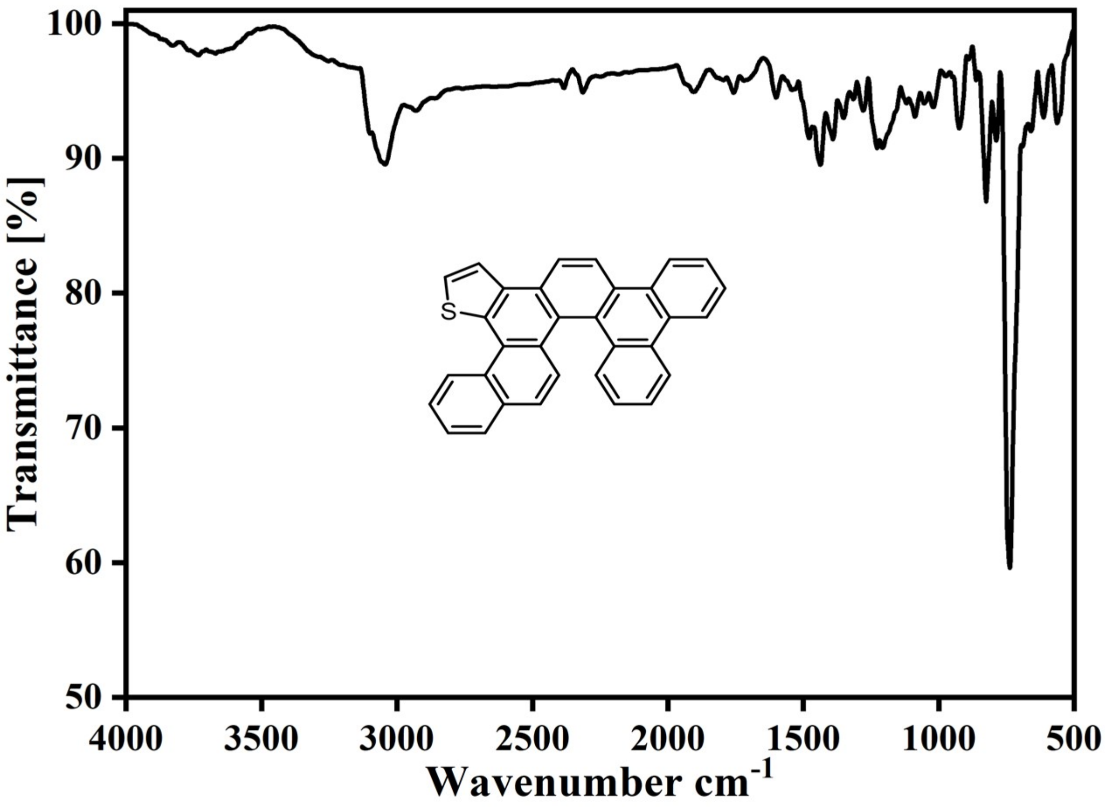

Figure S84. IR spectrum of **4b**.

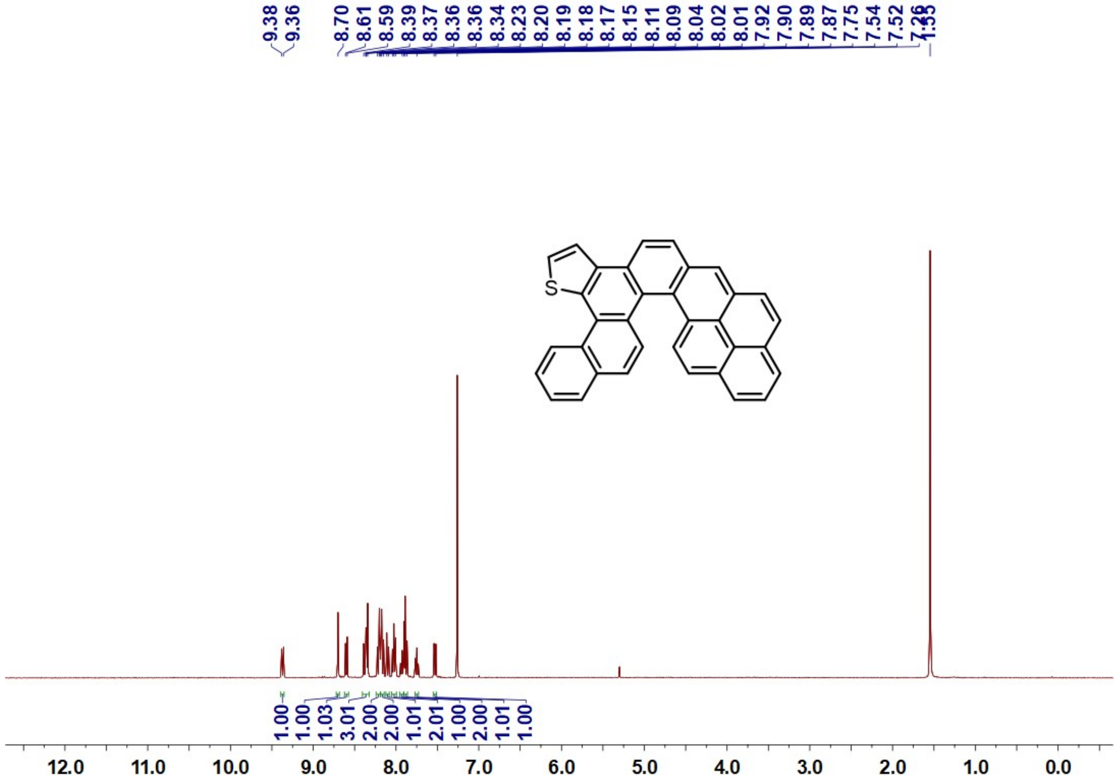

Figure S85. ^{1}H NMR (400 MHz, $CDCl_3$) spectrum of **4c**.

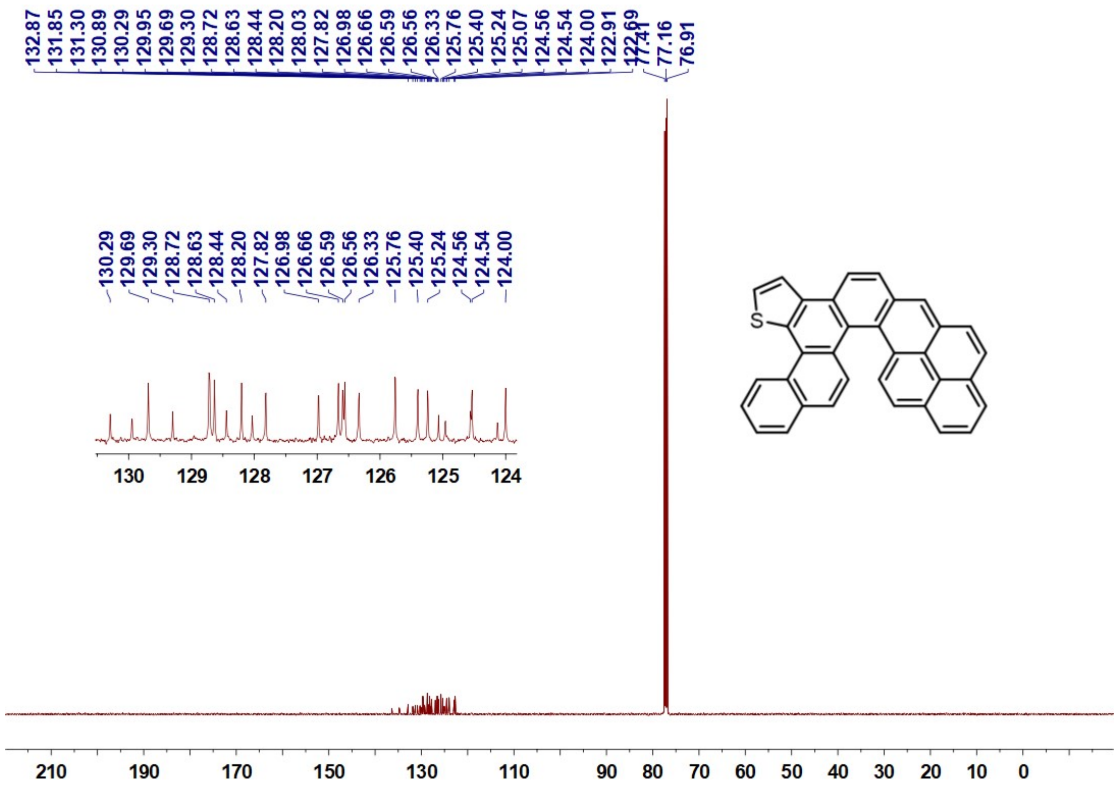

Figure S86. ^{13}C NMR (100 MHz, $CDCl_3$) spectrum of **4c**.

National Center for Organic Mass Spectrometry in Shanghai
Shanghai Institute of Organic Chemistry
Chinese Academic of Sciences
HIGH RESOLUTION MS REPORT

Instrument: JMS-S3000 MALDI-TOFMS

Sample Serial Number: SXL-2-38-COL-WS

Operator: Zhang, Li

Date: 2021/04/29

Operation Mode: MALDI-Positive

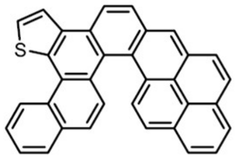

Elemental Composition Estimation

Parameters:

Mass	Tolerance	Electron Mode	Charge	DBE Range	Max Results
459.11987 ± 0.00230	5.0 ppm	Odd/Even	+1	-0.5 - 200.0	100

Elements

C	H	N	O	I	S	Si
0 - 34	0 - 200	0 - 0	0 - 0	0 - 0	0 - 1	0 - 3

Results:

#	Formula	Mass	DBE	Abs. Error (u)	Error (u)	Error (ppm)
1	C34 H19 S	459.12020	25.5	0.00032	-0.00032	-0.71

Figure S87. HRMS-MALDI spectrum of **4c**.

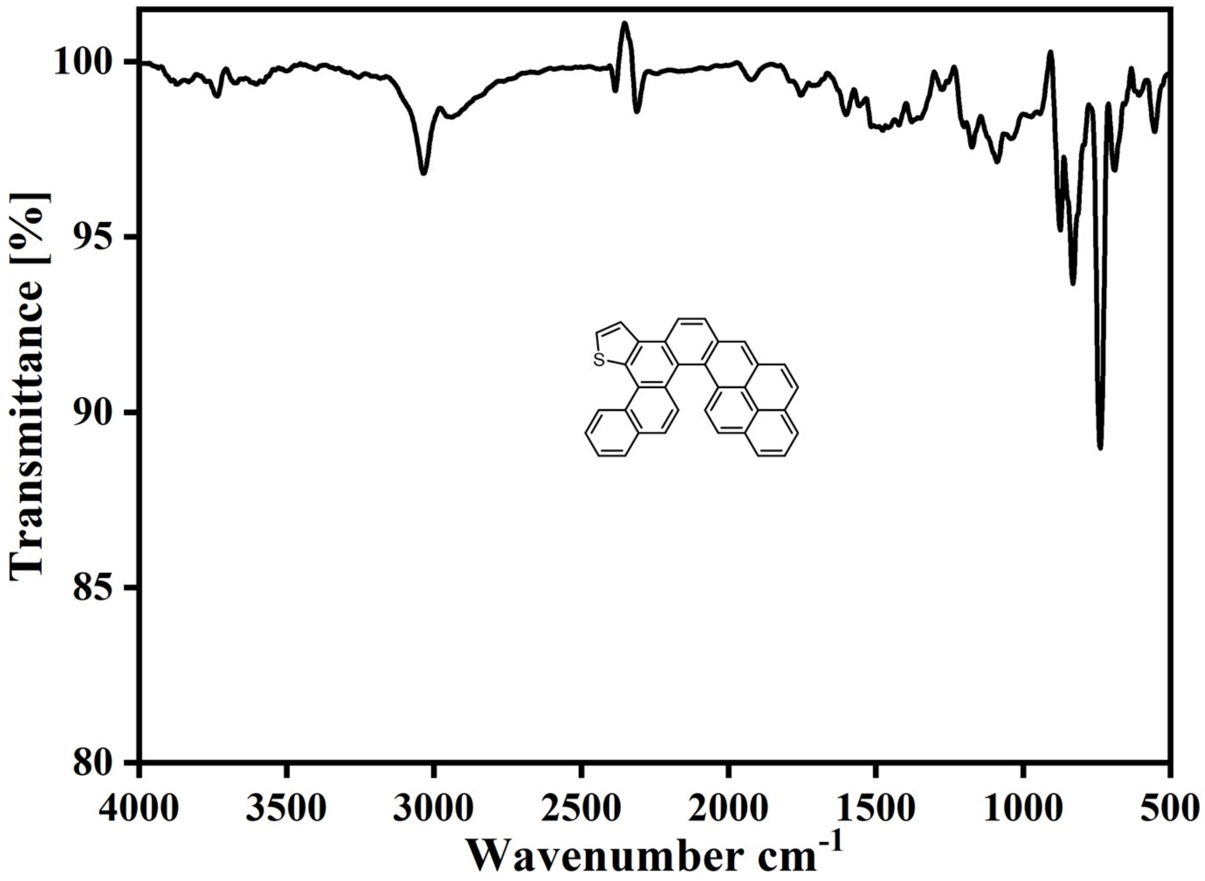

Figure S88. IR spectrum of **4c**.

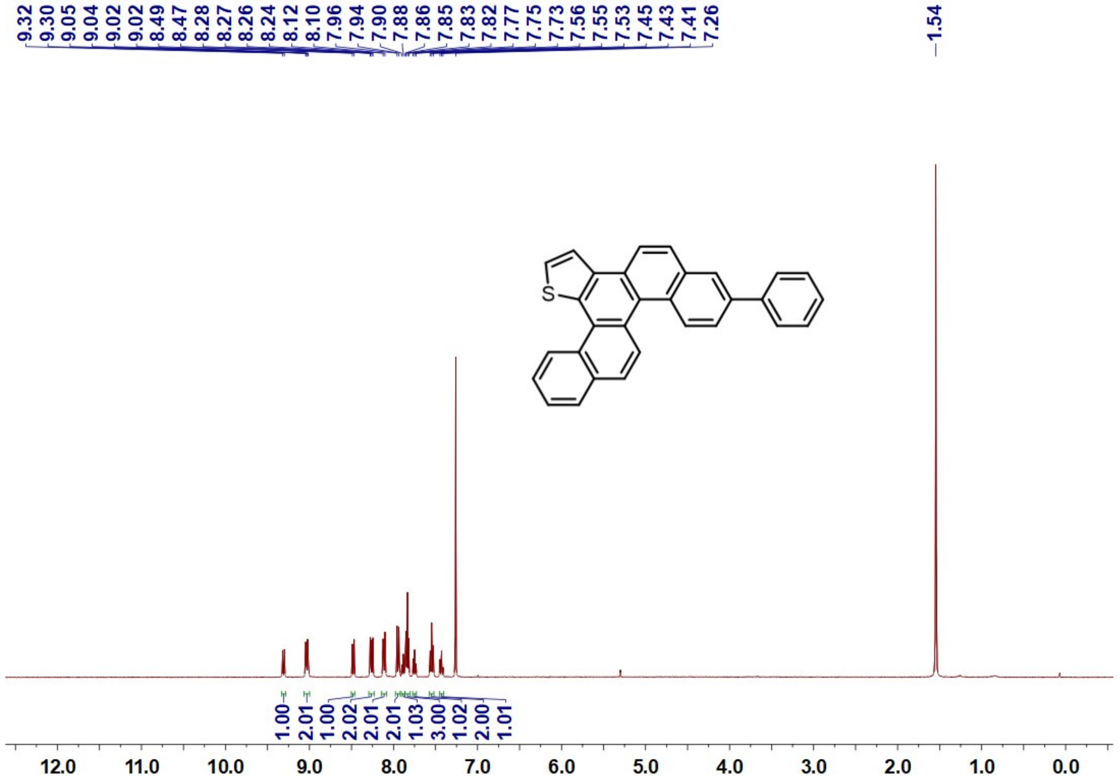

Figure S89. ^{1}H NMR (400 MHz, $CDCl_3$) spectrum of **4d**.

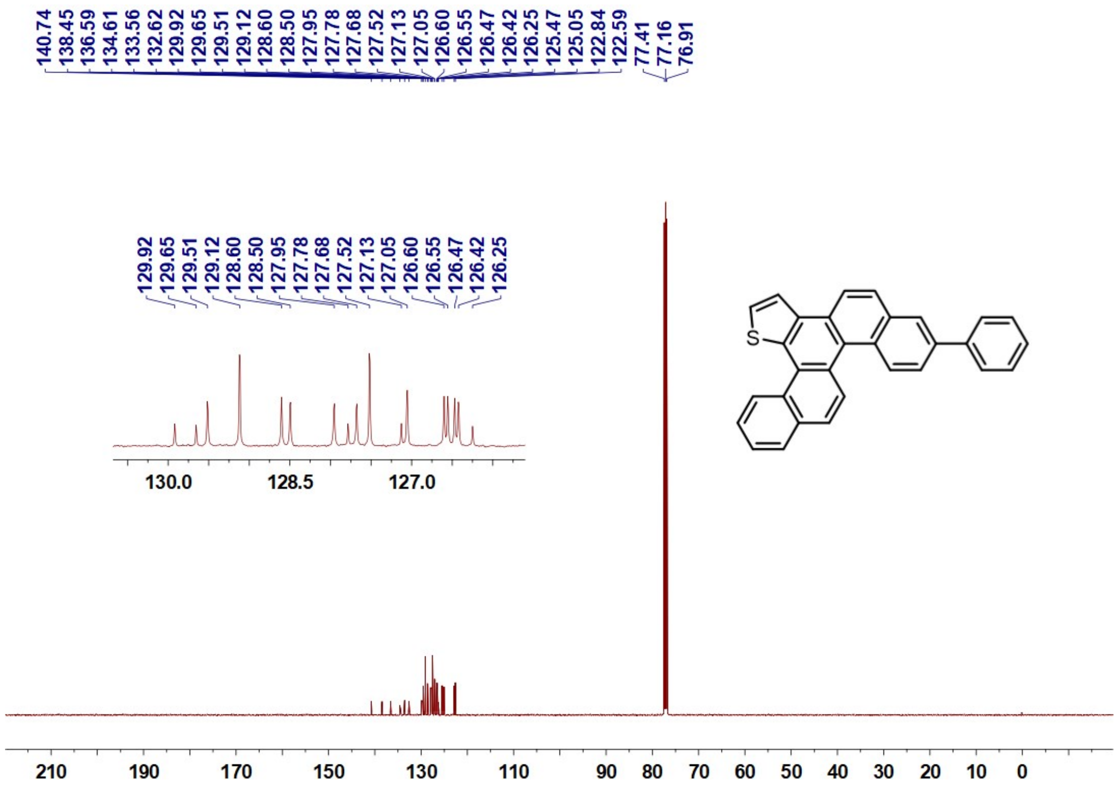

Figure S90. ^{13}C NMR (100 MHz, $CDCl_3$) spectrum of **4d**.

National Center for Organic Mass Spectrometry in Shanghai
Shanghai Institute of Organic Chemistry
Chinese Academic of Sciences
HIGH RESOLUTION MS REPORT

Instrument: JMS-S3000 MALDI-TOFMS

Sample Serial Number: SXL-2-57-COL-PP

Operator: Zhang, Li

Date: 2021/04/29

Operation Mode: MALDI-Positive

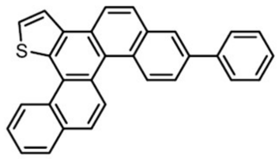

Elemental Composition Estimation

Parameters:

Mass	Tolerance	Electron Mode	Charge	DBE Range	Max Results
410.11080 ± 0.00205	5.0 ppm	Odd/Even	+1	-0.5 - 200.0	100

Elements

C 0 - 30 H 0 - 200 N 0 - 0 O 0 - 0 I 0 - 0 S 0 - 1 Si 0 - 3

Results:

#	Formula	Mass	DBE	Abs. Error (u)	Error (u)	Error (ppm)
1	C30 H18 S	410.11237	22.0	0.00157	-0.00157	-3.83

Figure S91. HRMS-MALDI spectrum of **4d**.

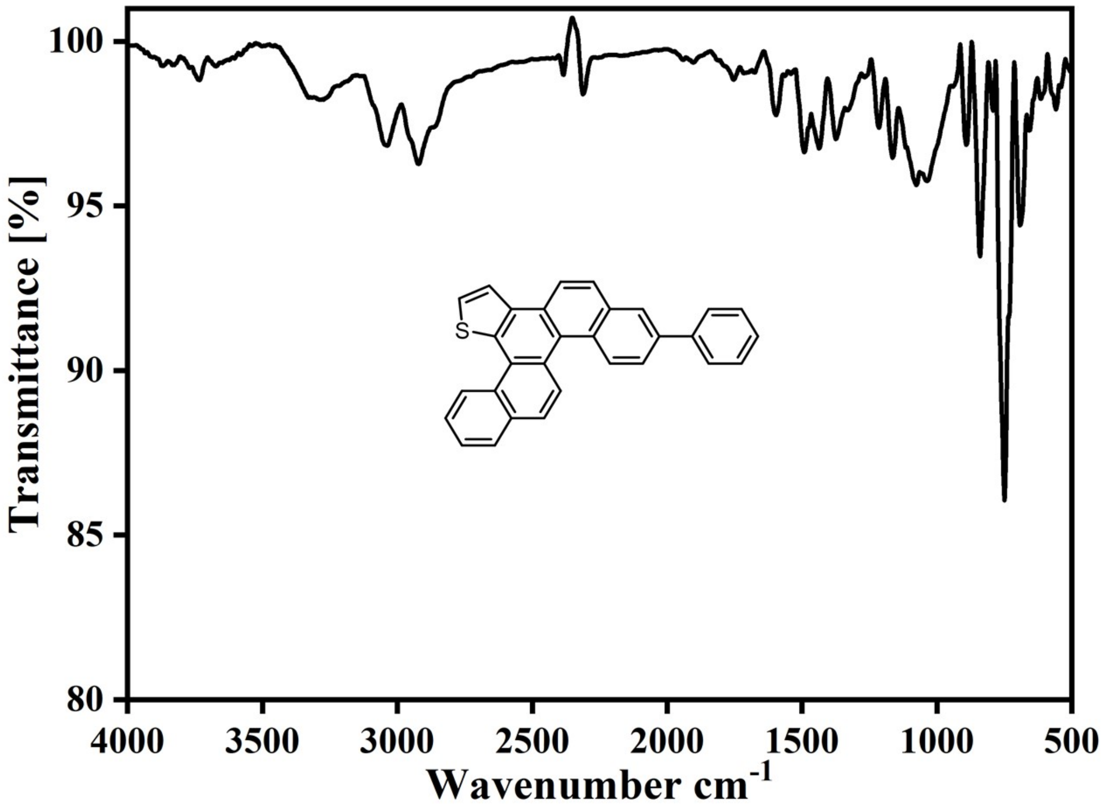

Figure S92. IR spectrum of **4d**.

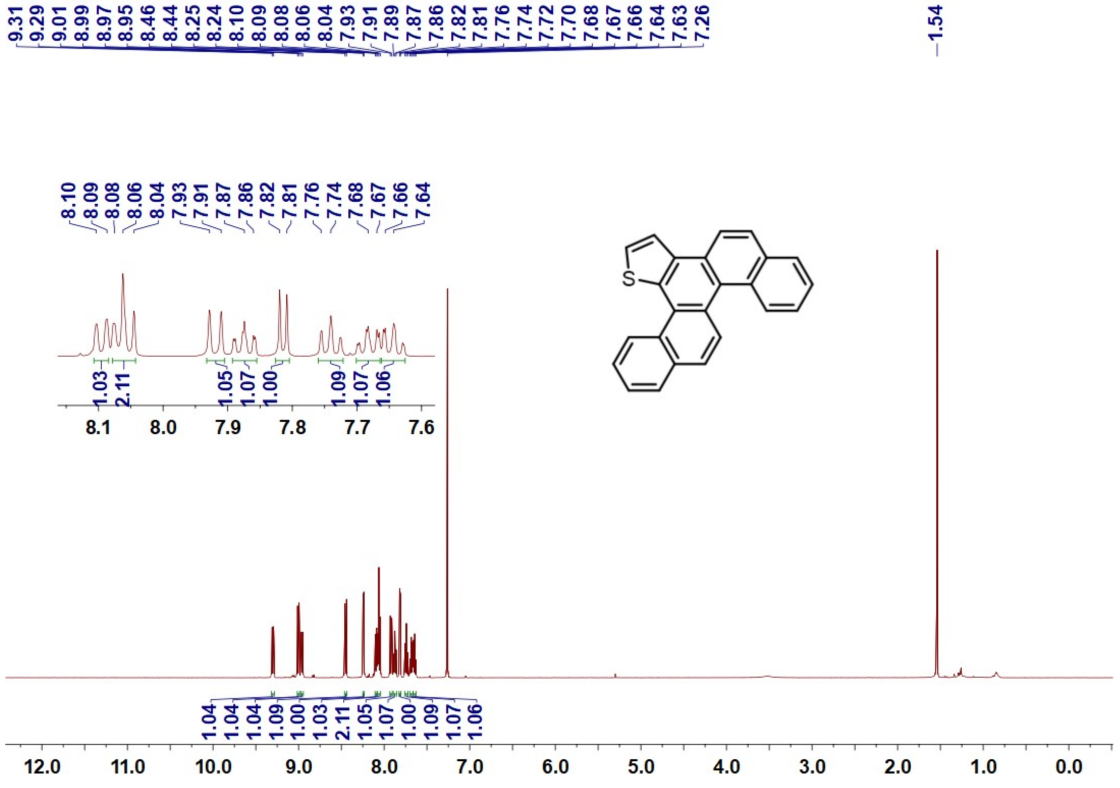

Figure S93. ^{1}H NMR (400 MHz, $CDCl_3$) spectrum of **4e**.

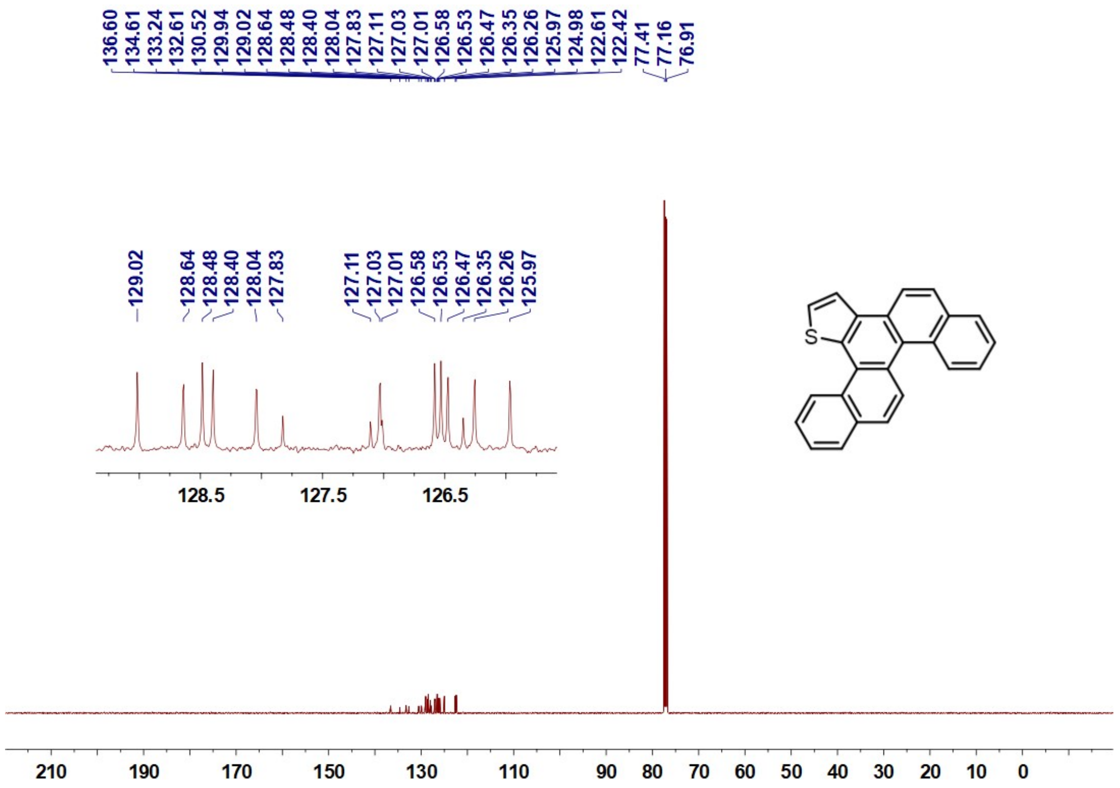

Figure S94. ^{13}C NMR (100 MHz, $CDCl_3$) spectrum of **4e**.

National Center for Organic Mass Spectrometry in Shanghai
Shanghai Institute of Organic Chemistry
Chinese Academic of Sciences
HIGH RESOLUTION MS REPORT

Instrument: JMS-S3000 MALDI-TOFMS

Sample Serial Number: SXL-3-67

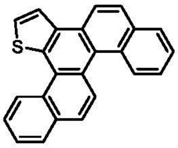

Operator: Zhang, Li

Date: 2021/12/02

Operation Mode: MALDI-Positive

Elemental Composition Estimation

Parameters:

Mass	Tolerance	Electron Mode	Charge	DBE Range	Max Results
334.07942 ± 0.00167	5.0 ppm	Odd/Even	+1	-0.5 - 200.0	100

Elements

C 0 - 25 H 0 - 20 S 0 - 2

Results:

#	Formula	Mass	DBE	Abs. Error (u)	Error (u)	Error (ppm)
1	C24 H14 S	334.08107	18.0	0.00165	-0.00165	-4.95

Figure S95. HRMS-MALDI spectrum of **4e**.

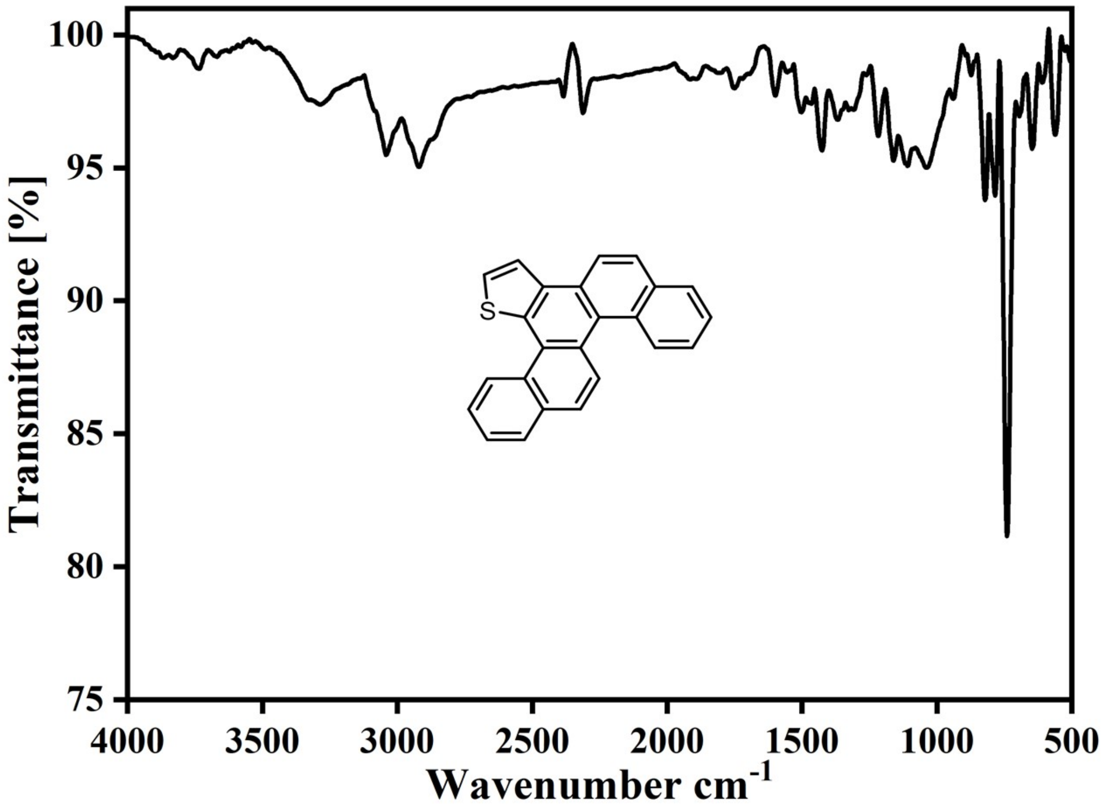

Figure S96. IR spectrum of **4e**.

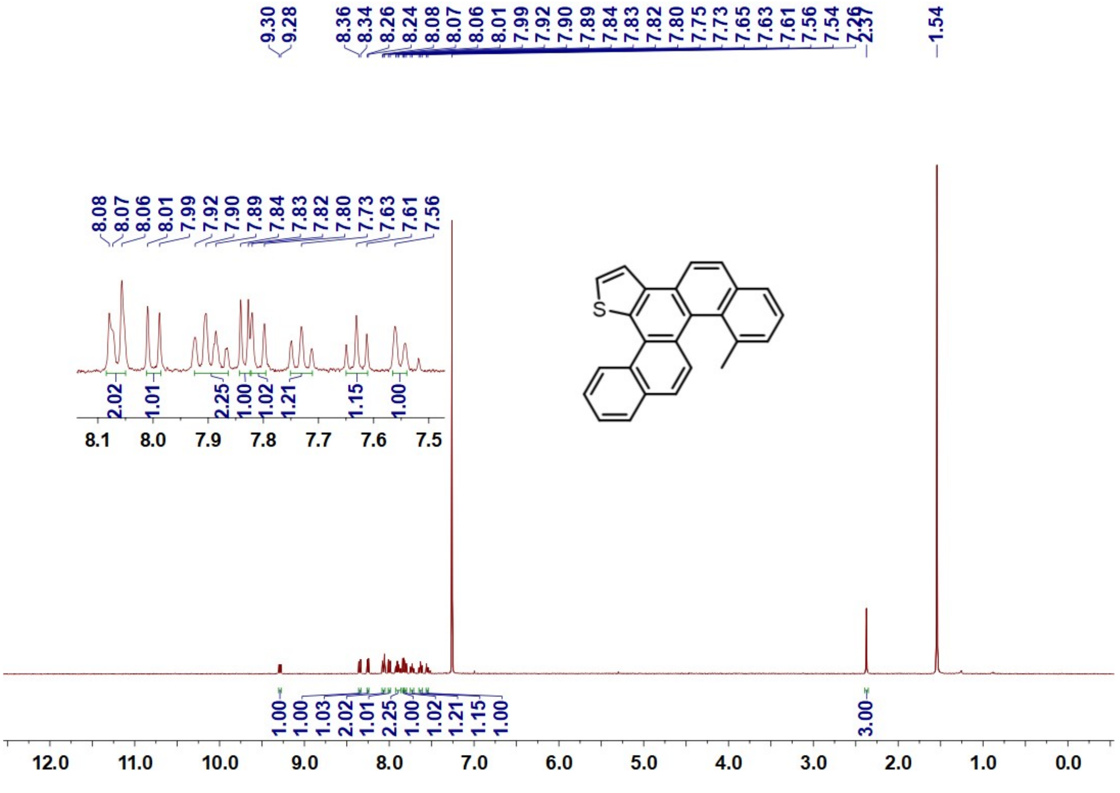

Figure S97. ^{1}H NMR (400 MHz, $CDCl_3$) spectrum of **4f**.

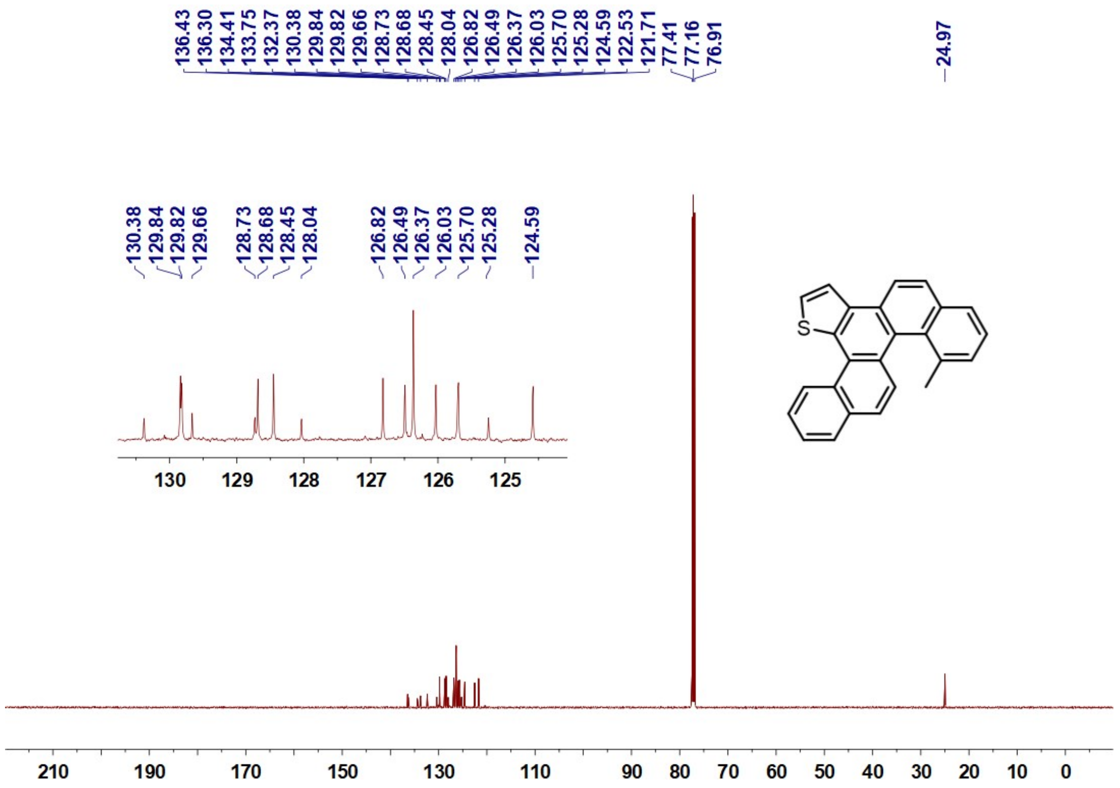

Figure S98. ^{13}C NMR (100 MHz, $CDCl_3$) spectrum of **4f**.

National Center for Organic Mass Spectrometry in Shanghai
Shanghai Institute of Organic Chemistry
Chinese Academic of Sciences
High Resolution MS DATA REPORT

Instrument: Thermo Fisher Scientific LTQ FTICR-MS

Card Serial Number : D20220786

Sample Serial Number: SXL-T2NB-3

Operator : DONG Date: 2022/01/13

Operation Mode: DART POSITIVE

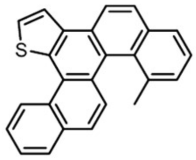

Elemental composition search on mass 349.1045

m/z= 344.1045-354.1045

m/z	Theo. Mass	Delta (ppm)	RDB equiv.	Composition
349.1045	349.1045	-0.17	17.5	$C_{25}H_{17}S$
	349.1044	0.29	6.5	$C_{17}H_{18}F_5S$
	349.1046	-0.33	10.5	$C_{19}H_{16}O_3F_3$
	349.1035	2.94	14.5	$C_{22}H_{15}O_2F_2$
	349.1057	-3.44	13.5	$C_{22}H_{18}OFS$
	349.1057	-3.60	6.5	$C_{16}H_{17}O_4F_4$

Figure S99. HRMS-DART spectrum of **4f**.

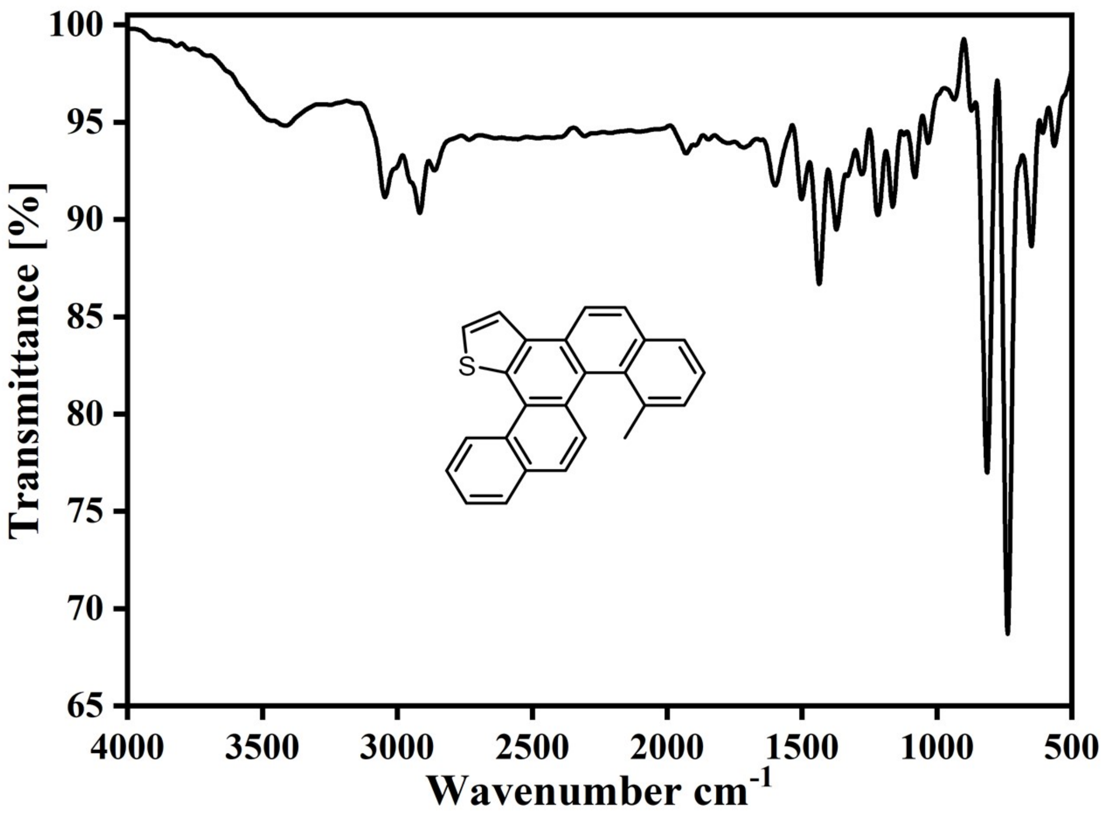

Figure S100. IR spectrum of **4f**.

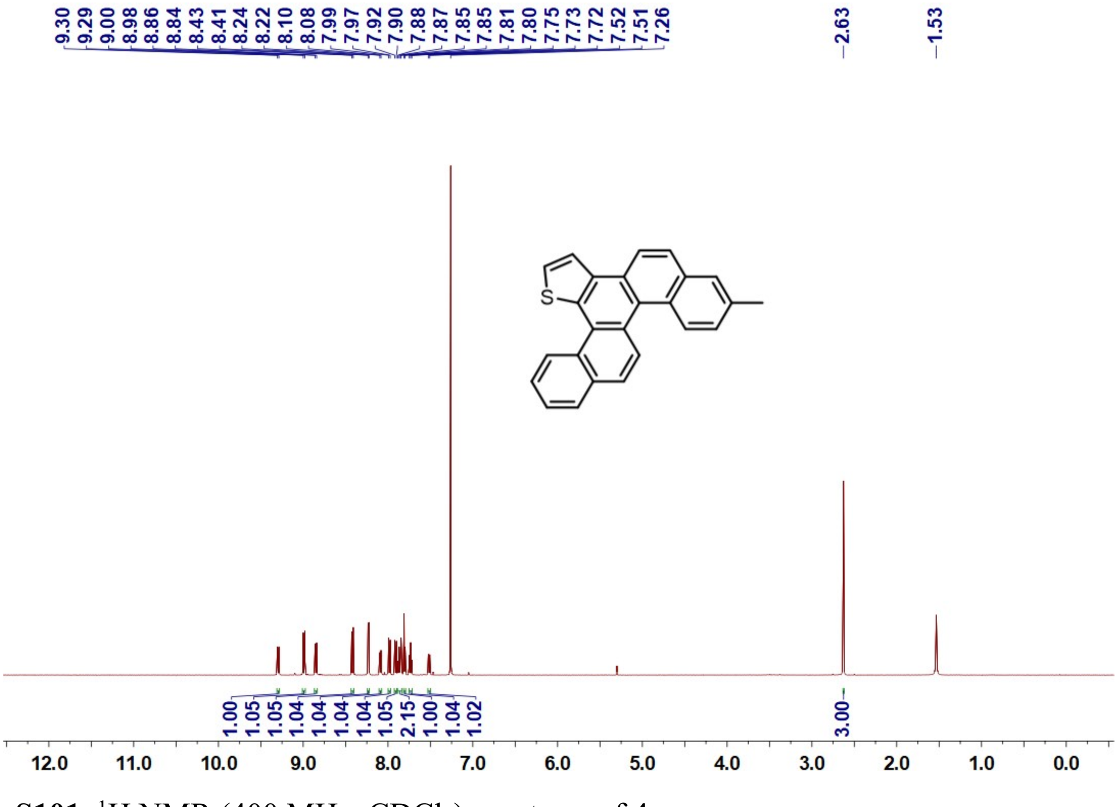

Figure S101. ^{1}H NMR (400 MHz, $CDCl_3$) spectrum of **4g**.

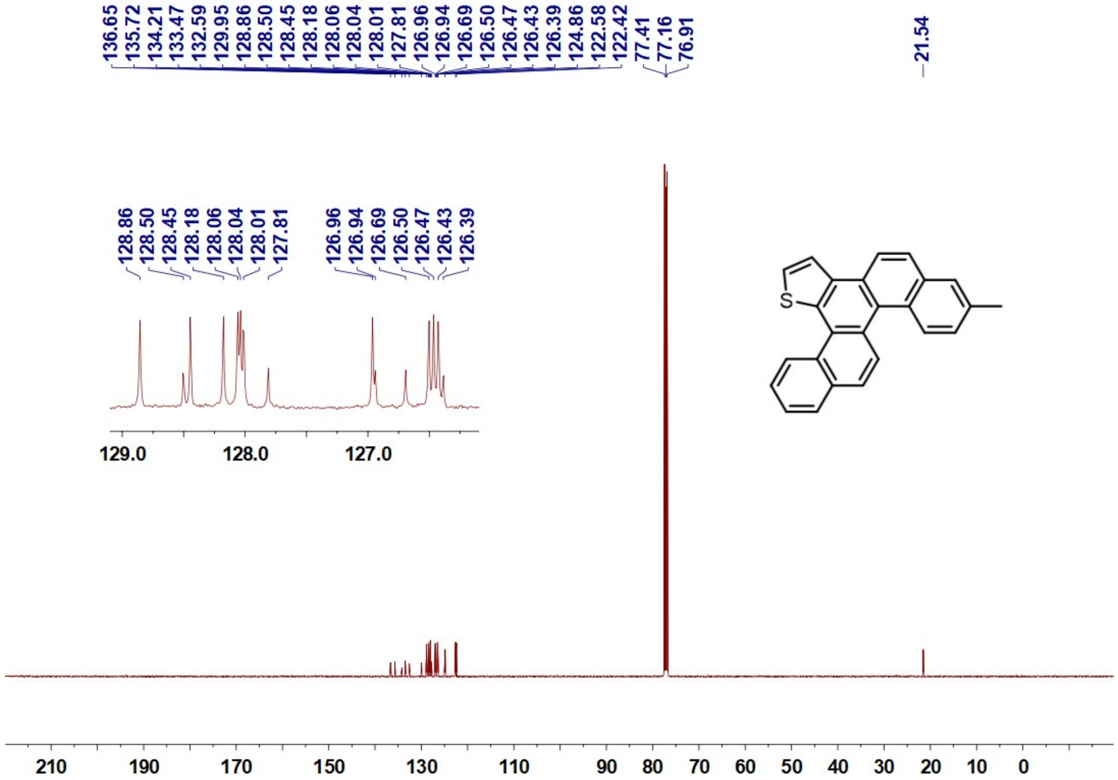

Figure S102. ^{13}C NMR (100 MHz, CDCl$_3$) spectrum of **4g**.

National Center for Organic Mass Spectrometry in Shanghai
Shanghai Institute of Organic Chemistry
Chinese Academic of Sciences
High Resolution MS DATA REPORT

Instrument: Thermo Fisher Scientific LTQ FTICR-MS

Card Serial Number : D20220784

Sample Serial Number: SXL-T4NB

Operator : DONG Date: 2022/01/13

Operation Mode: DART POSITIVE

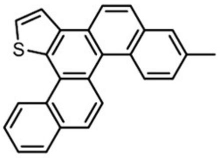

Elemental composition search on mass 349.1046

m/z= 344.1046-354.1046

m/z	Theo. Mass	Delta (ppm)	RDB equiv.	Composition
349.1046	349.1045	0.06	17.5	$C_{25}H_{17}S$
	349.1046	-0.10	10.5	$C_{19}H_{16}O_3F_3$
	349.1044	0.52	6.5	$C_{17}H_{18}F_5S$
	349.1035	3.17	14.5	$C_{22}H_{15}O_2F_2$
	349.1057	-3.21	13.5	$C_{22}H_{18}OFS$
	349.1057	-3.38	6.5	$C_{16}H_{17}O_4F_4$

Figure S103. HRMS-DART spectrum of **4g**.

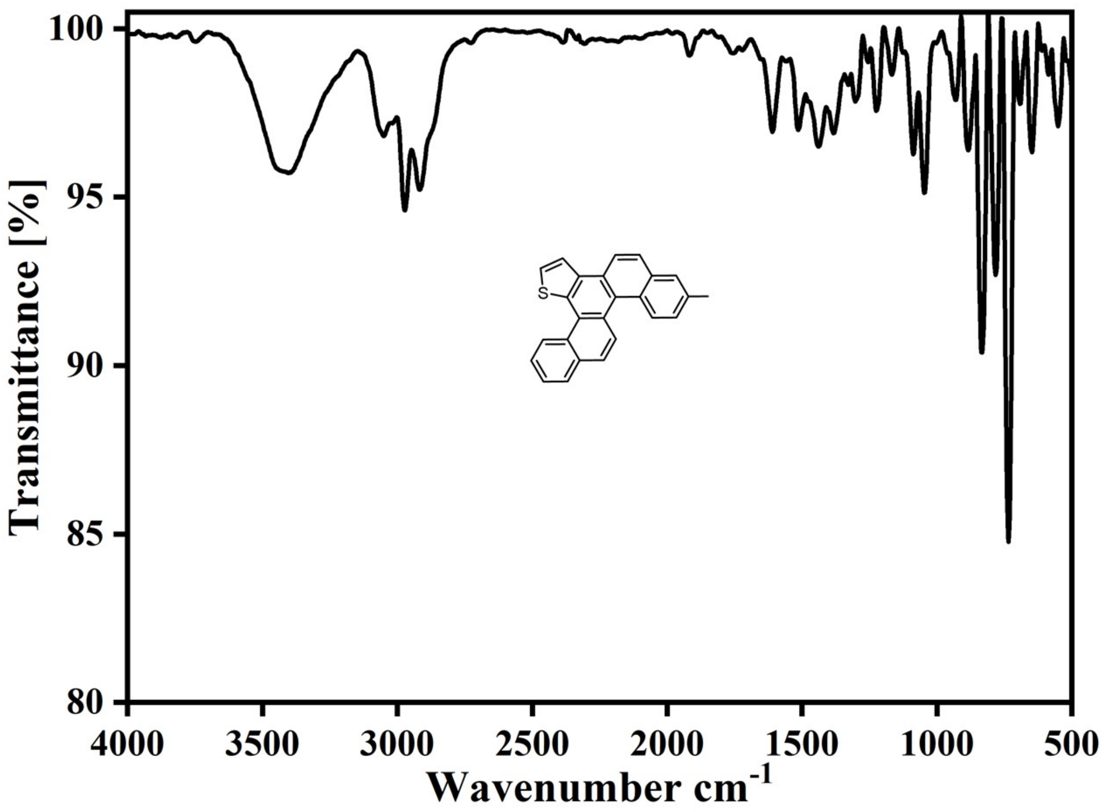

Figure S104. IR spectrum of **4g**.

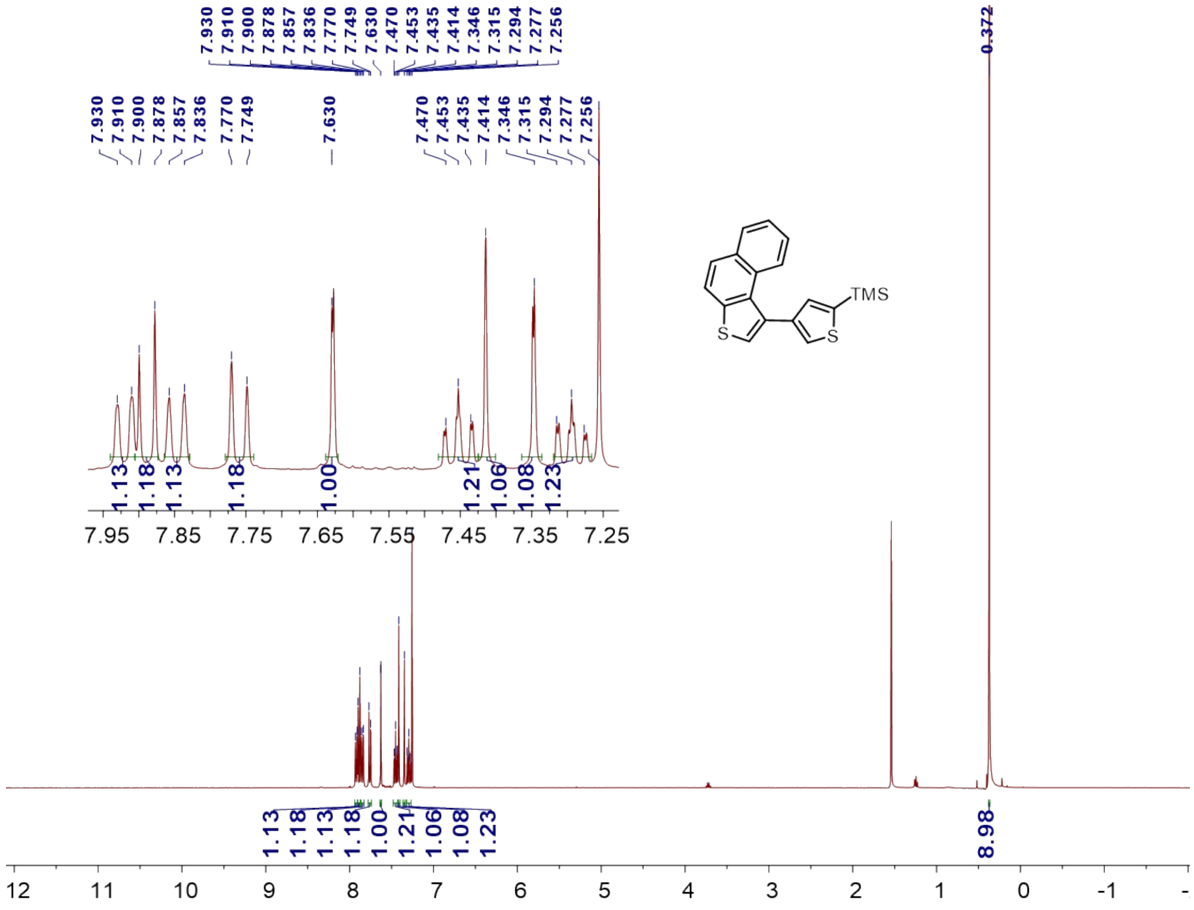

Figure S105. ^{1}H NMR (400 MHz, $CDCl_3$) spectrum of **12**.

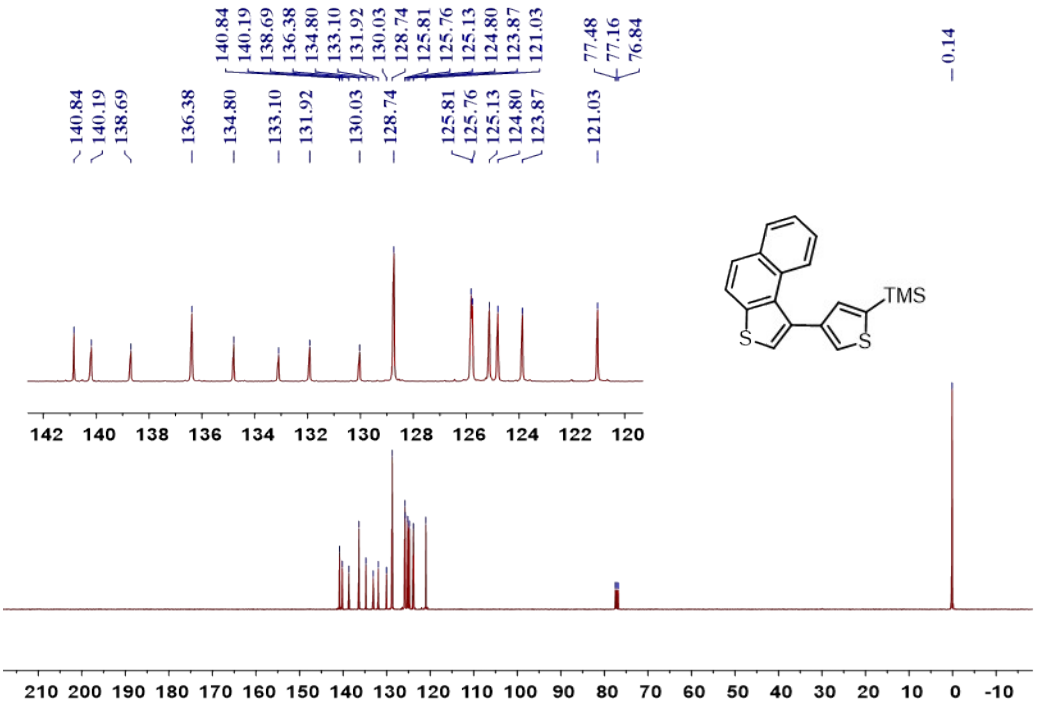

Figure S106. ^{13}C NMR (100 MHz, $CDCl_3$) spectrum of **12**.

National Center for Organic Mass Spectrometry in Shanghai
Shanghai Institute of Organic Chemistry
Chinese Academic of Sciences
High Resolution AP-MALDI- MS REPORT

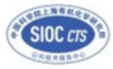

Instrument: Thermo Scientific Q Exactive HF Orbitrap-FTMS

Card Serial Number: E-W2023091232

Sample Serial Number: ML-3

Operator: Zhou BW Date: 2023/09/12

Operation Mode: AP-MALDI Positive Ion Mode

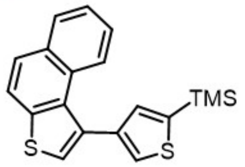

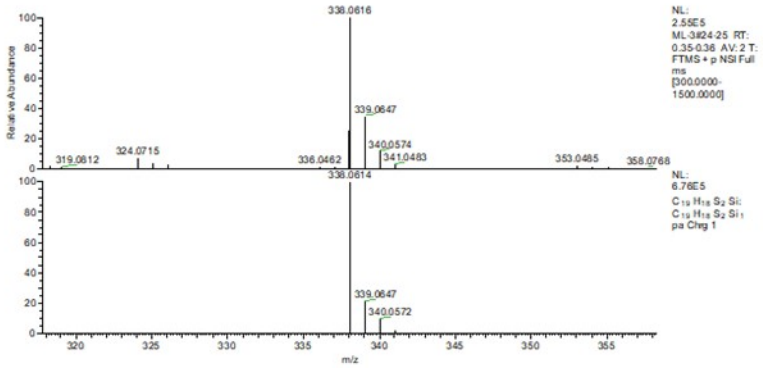

Elemental composition search on mass 338.0616

m/z= 333.0616-343.0616

m/z	Theo. Mass	Delta (ppm)	RDB equiv.	Composition
338.0616	338.0614	0.68	12.0	C 19 H 18 S 2 Si

Figure S107. HRMS-MALDI spectrum of **12**.

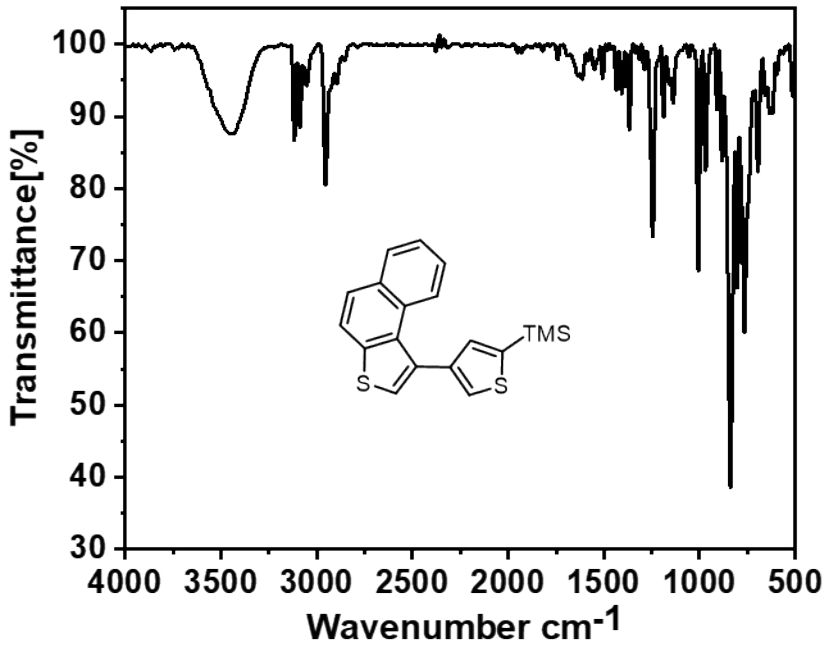

Figure S108. IR spectrum of **12**.

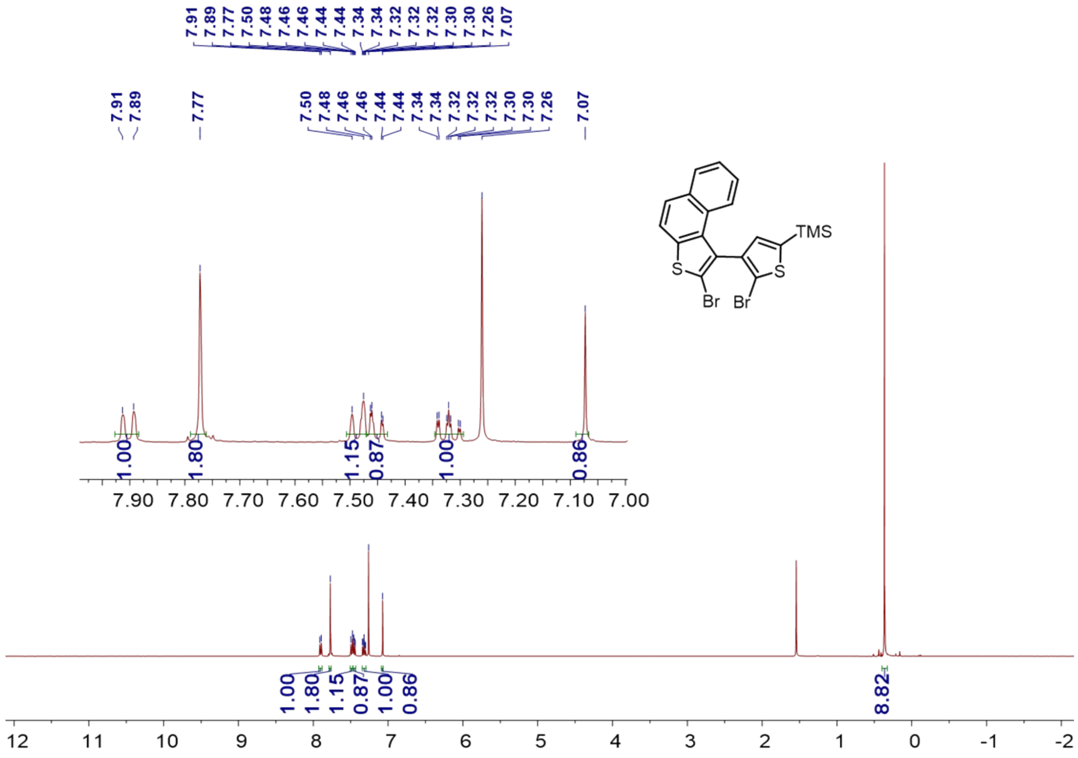

Figure S109. ^{1}H NMR (400 MHz, $CDCl_3$) spectrum of **13**.

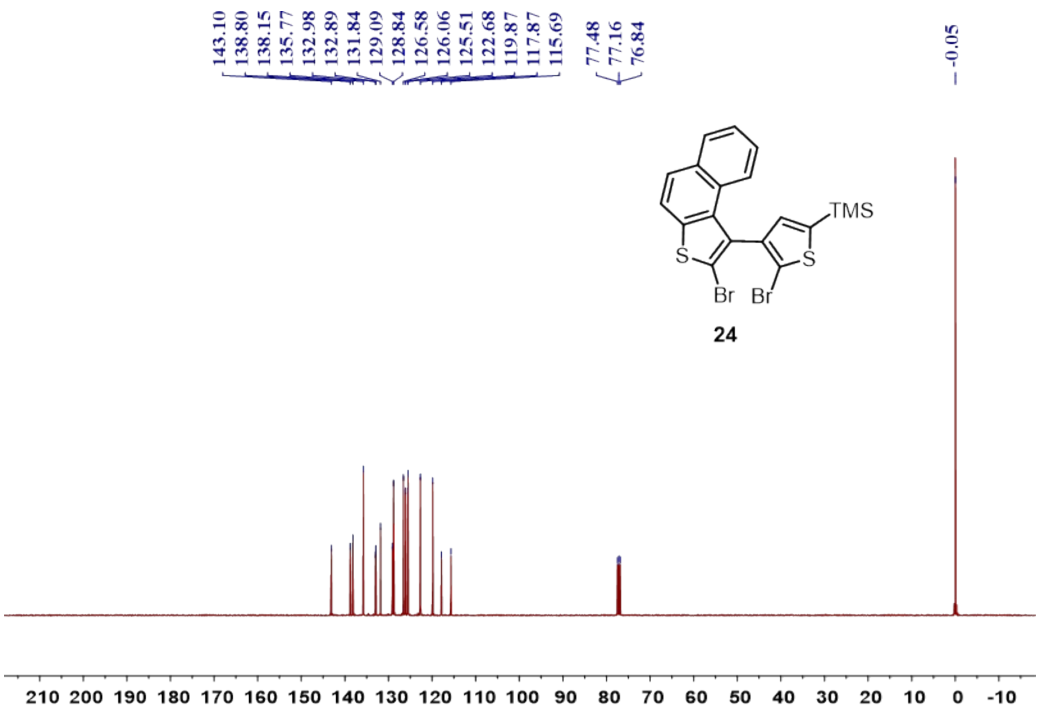

Figure S110. ^{13}C NMR (100 MHz, $CDCl_3$) spectrum of **13**.

National Center for Organic Mass Spectrometry in Shanghai
Shanghai Institute of Organic Chemistry
Chinese Academic of Sciences
High Resolution EI-MS Report
Low Resolution EI-MS Report

Instrument:	Waters Premier GC-TOF MS		
Operation Mode:	**EI** Positive Ion Mode		Electron Energy: 70eV
Card Serial Number:	GCT-P-EI-T24-01-OS-007		
Sample Serial Number:	HNU-ML-5		
Operator:	Liguangping	Date:	2023/12/21

m/z	Theo. Mass	Delta (ppm)	RDB equiv.	Composition
493.8823	493.8823	0.01	20.0	$C_{20}H_4O_4N_2BrFSSi$
	493.8822	0.19	18.5	$C_{22}H_7O_2NBr_2F$
	493.8824	-0.19	12.0	$C_{19}H_{16}Br_2S_2Si$
	493.8825	-0.31	5.0	$C_{13}H_{15}O_3Br_2F_3SSi$
	493.8821	0.45	16.0	$C_{18}H_6ON_2BrF_3S_2Si$

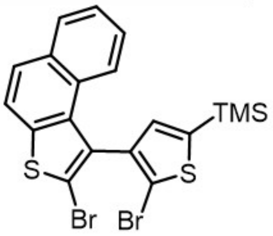

Figure S111. HRMS-EI spectrum of **13**.

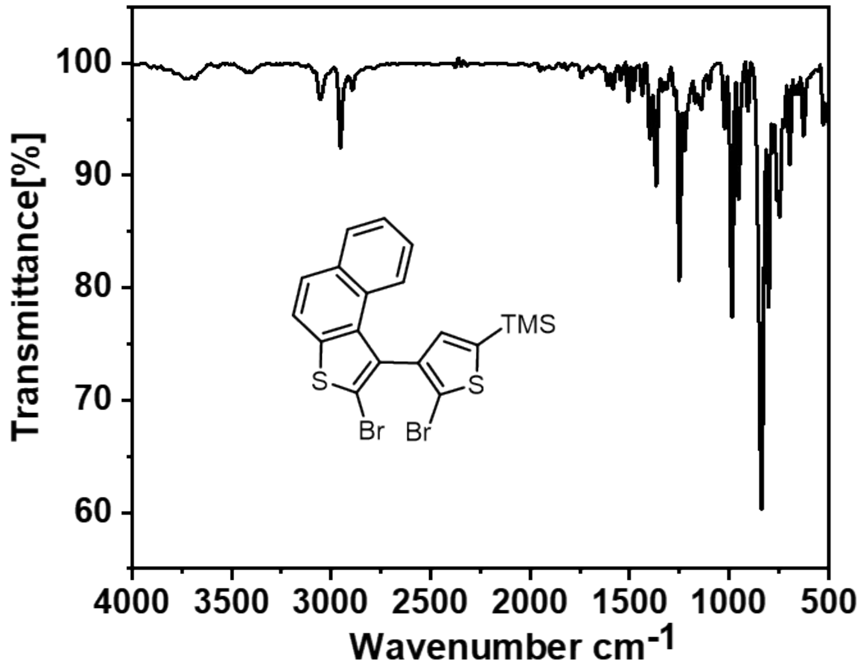

Figure S112. IR spectrum of **13**.

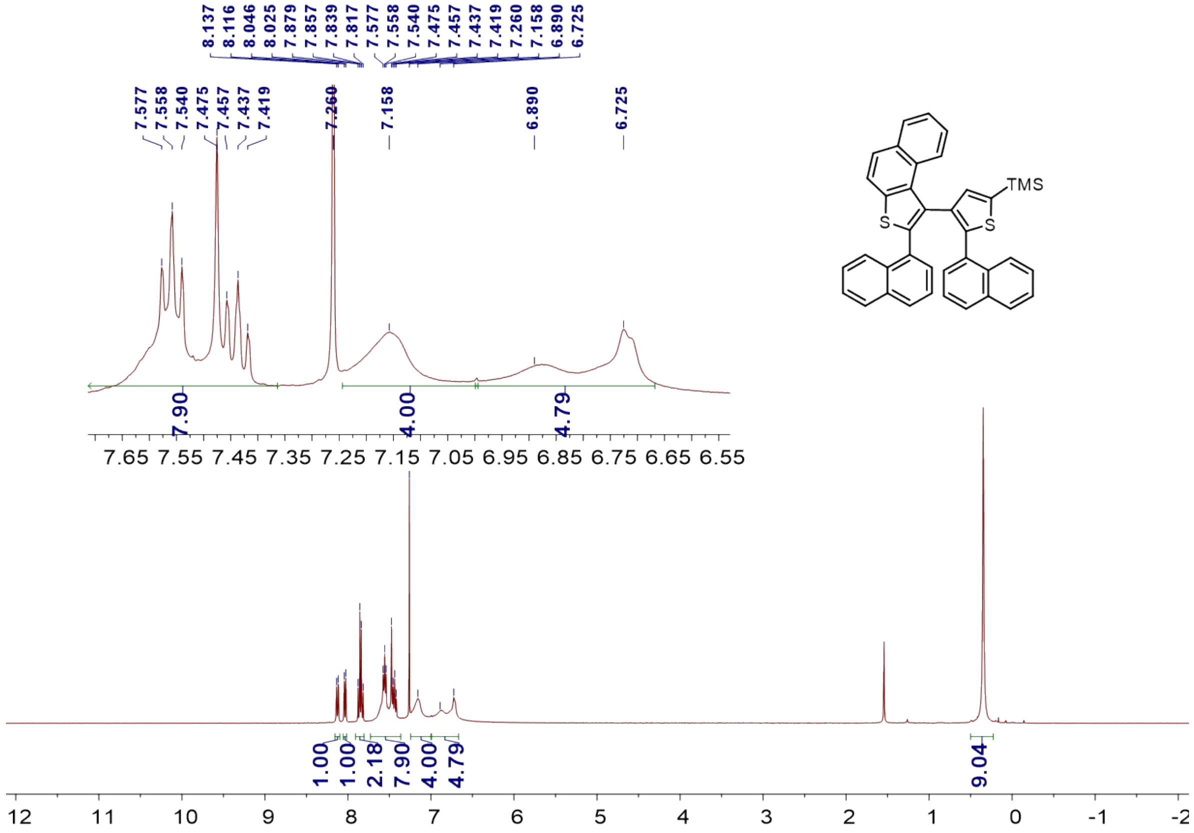

Figure S113. ^{1}H NMR (400 MHz, $CDCl_3$) spectrum of **5a**.

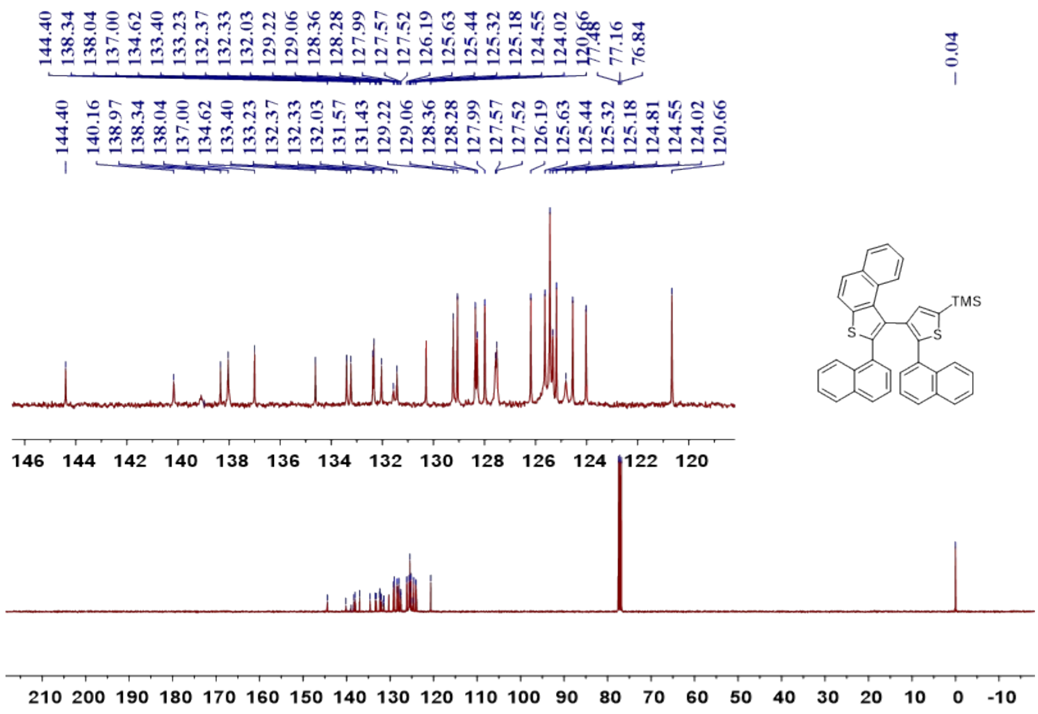

Figure S114. ^{13}C NMR (100 MHz, $CDCl_3$) spectrum of **5a**.

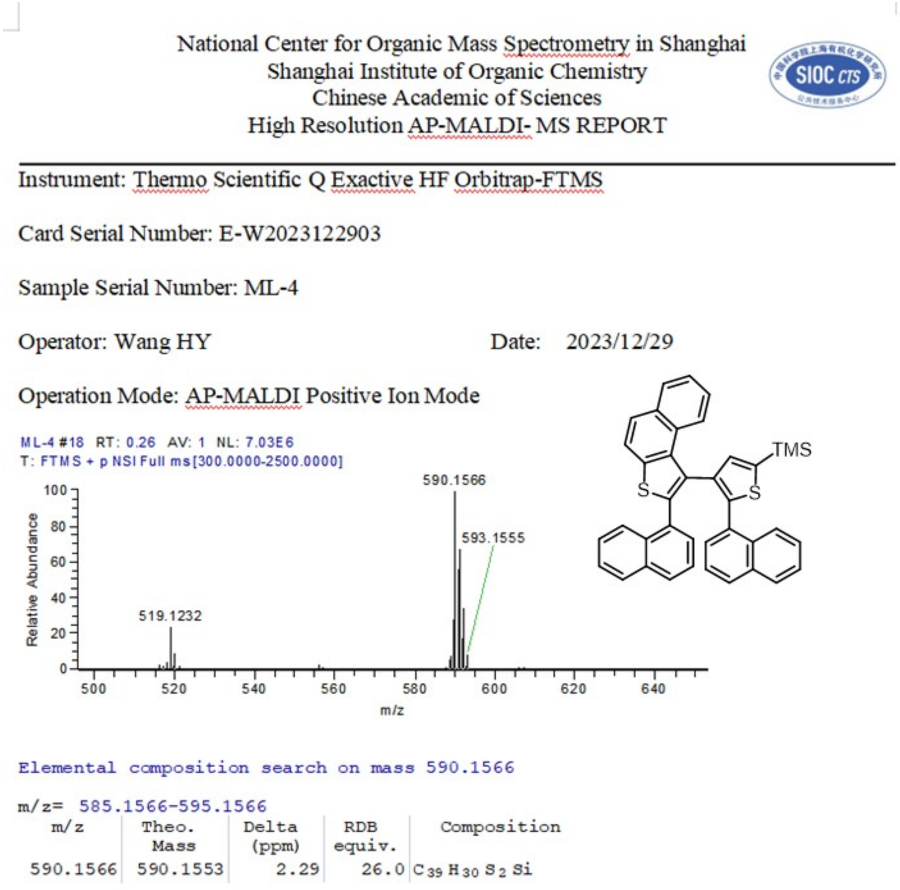

National Center for Organic Mass Spectrometry in Shanghai
Shanghai Institute of Organic Chemistry
Chinese Academic of Sciences
High Resolution AP-MALDI- MS REPORT

Instrument: Thermo Scientific Q Exactive HF Orbitrap-FTMS

Card Serial Number: E-W2023122903

Sample Serial Number: ML-4

Operator: Wang HY Date: 2023/12/29

Operation Mode: AP-MALDI Positive Ion Mode

Elemental composition search on mass 590.1566

m/z= 585.1566-595.1566

m/z	Theo. Mass	Delta (ppm)	RDB equiv.	Composition
590.1566	590.1553	2.29	26.0	$C_{39}H_{30}S_2Si$

Figure S115. HRMS-MALDI spectrum of **5a**.

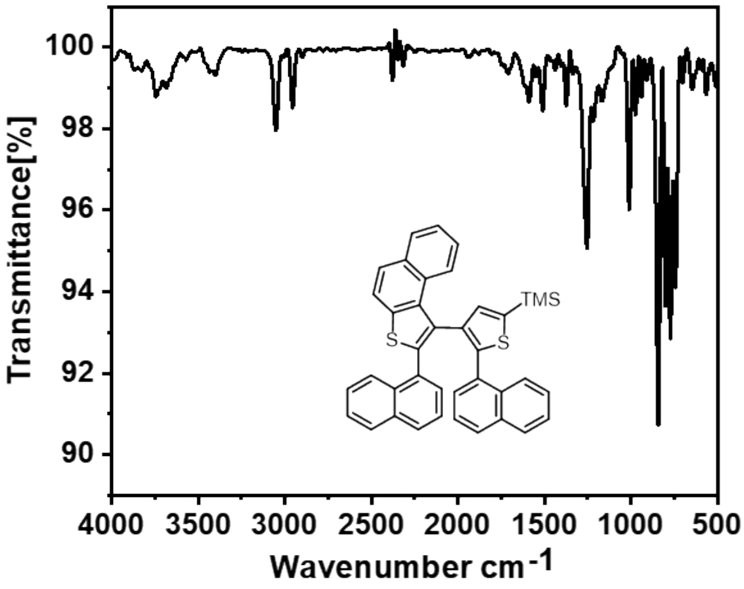

Figure S116. IR spectrum of **5a**.

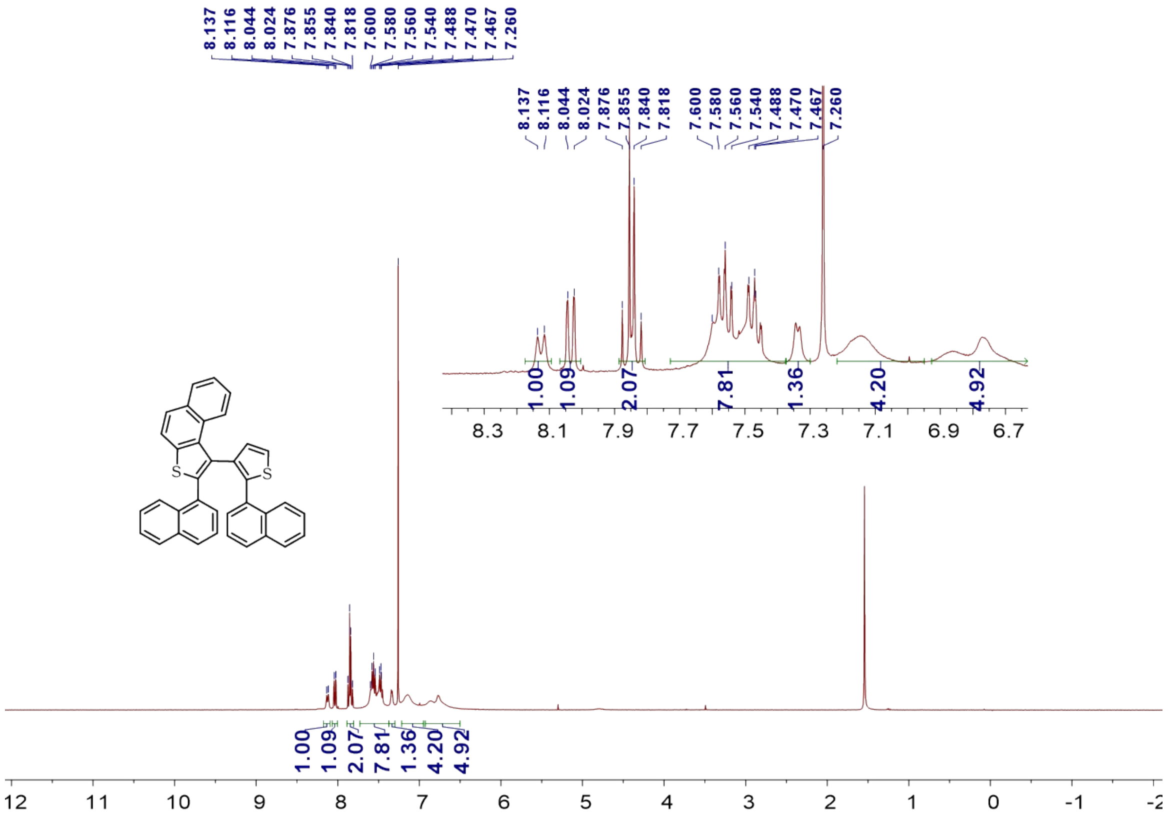

Figure S117. ^{1}H NMR (400 MHz, $CDCl_3$) spectrum of **5c**.

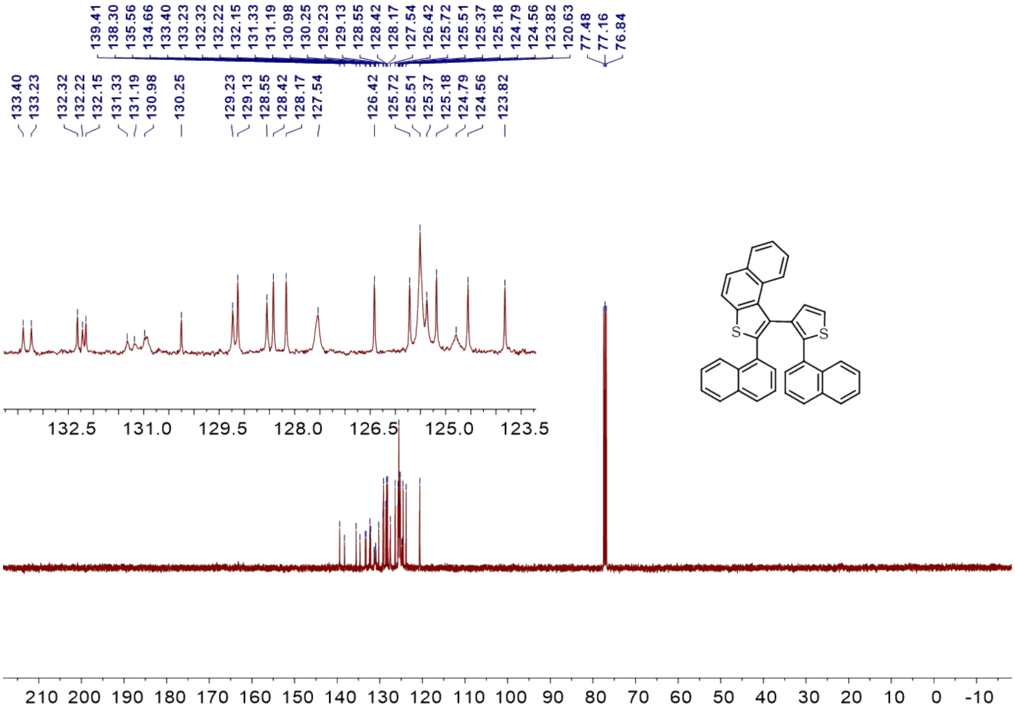

Figure S118. ^{13}C NMR (100 MHz, $CDCl_3$) spectrum of **5c**.

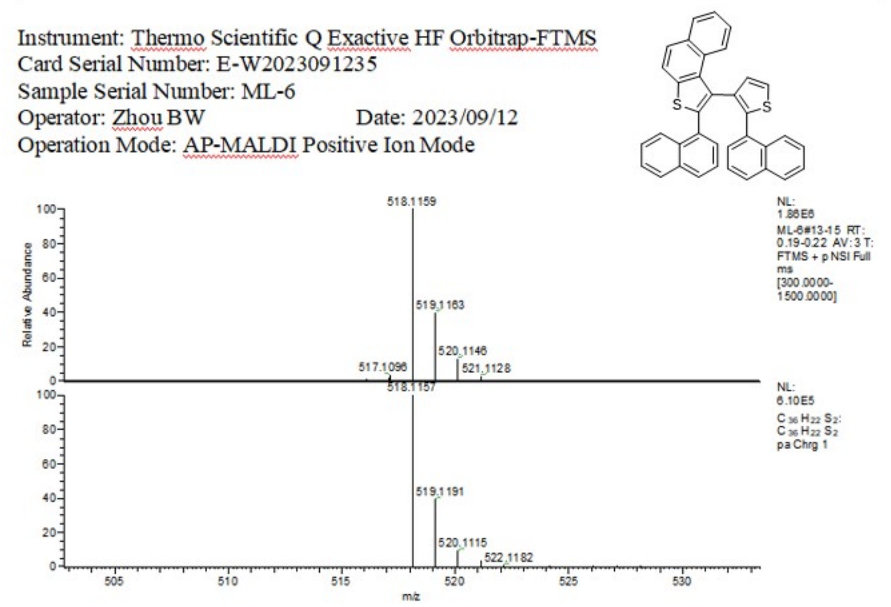

National Center for Organic Mass Spectrometry in Shanghai
Shanghai Institute of Organic Chemistry
Chinese Academic of Sciences
High Resolution AP-MALDI- MS REPORT

Instrument: Thermo Scientific Q Exactive HF Orbitrap-FTMS
Card Serial Number: E-W2023091235
Sample Serial Number: ML-6
Operator: Zhou BW Date: 2023/09/12
Operation Mode: AP-MALDI Positive Ion Mode

Elemental composition search on mass 518.1159

m/z= 513.1159-523.1159

m/z	Theo. Mass	Delta (ppm)	RDB equiv.	Composition
518.1159	518.1157	0.30	26.0	$C_{36}H_{22}S_2$

Figure S119. HRMS-MALDI spectrum of **5c**.

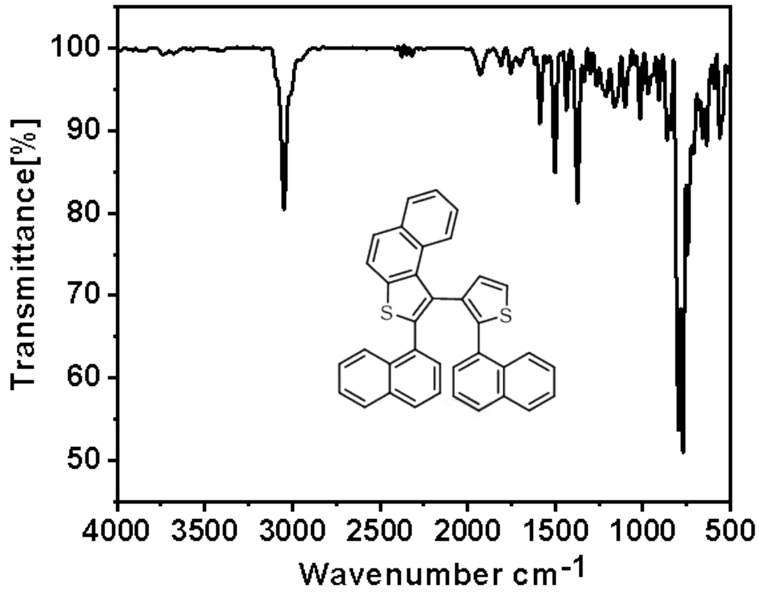

Figure S120. IR spectrum of **5c**.

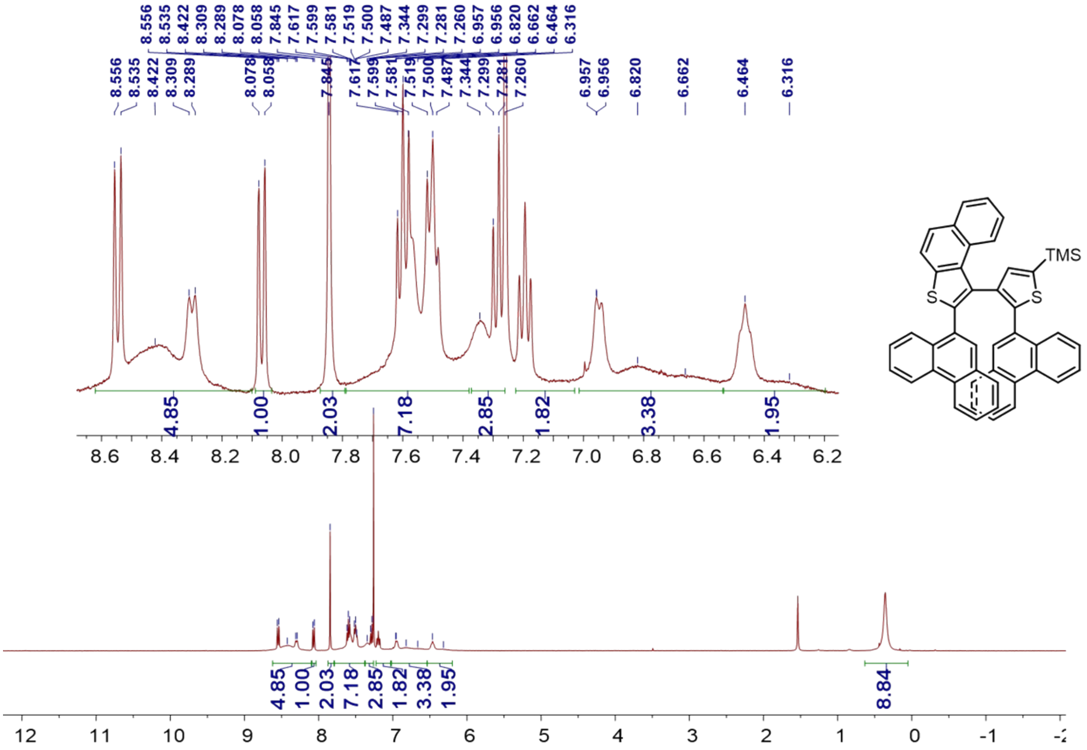

Figure S121. ^{1}H NMR (400 MHz, $CDCl_3$) spectrum of **5b**.

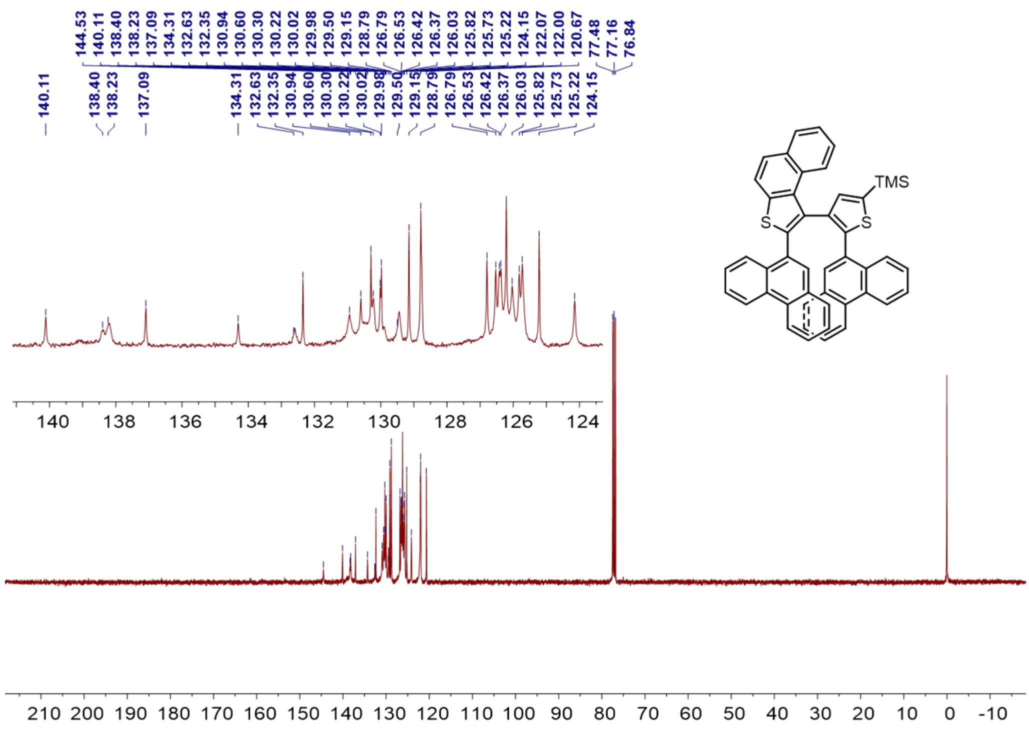

Figure S122. ^{13}C NMR (100 MHz, $CDCl_3$) spectrum of **5b**.

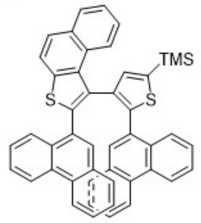

National Center for Organic Mass Spectrometry in Shanghai
Shanghai Institute of Organic Chemistry
Chinese Academic of Sciences
High Resolution AP-MALDI- MS REPORT

Instrument: Thermo Scientific Q Exactive HF Orbitrap-FTMS

Card Serial Number: E-W2023122905

Sample Serial Number: ML-7

Operator: Wang HY Date: 2023/12/29

Operation Mode: AP-MALDI Positive Ion Mode

Elemental composition search on mass 690.1870

m/z= 685.1870-695.1870

m/z	Theo. Mass	Delta (ppm)	RDB equiv.	Composition
690.1870	690.1866	0.64	32.0	$C_{47}H_{34}S_2Si$

Figure S123. HRMS-MALDI spectrum of **5b**.

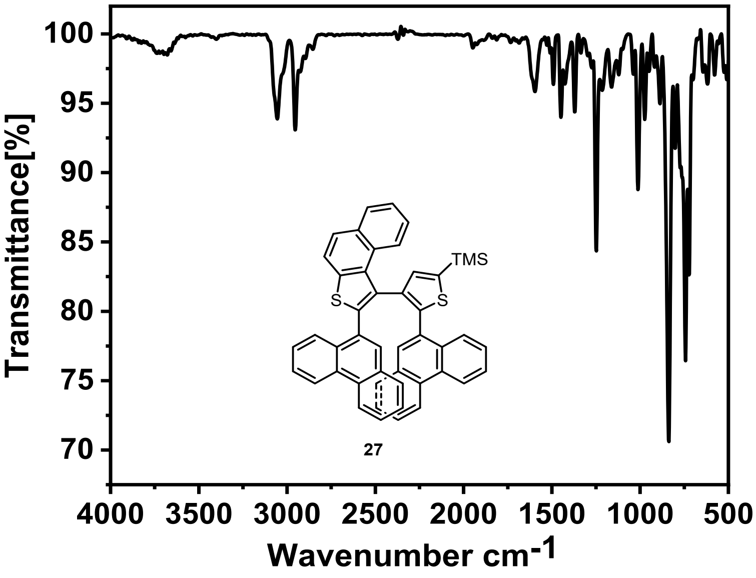

Figure S124. IR spectrum of **5b**.

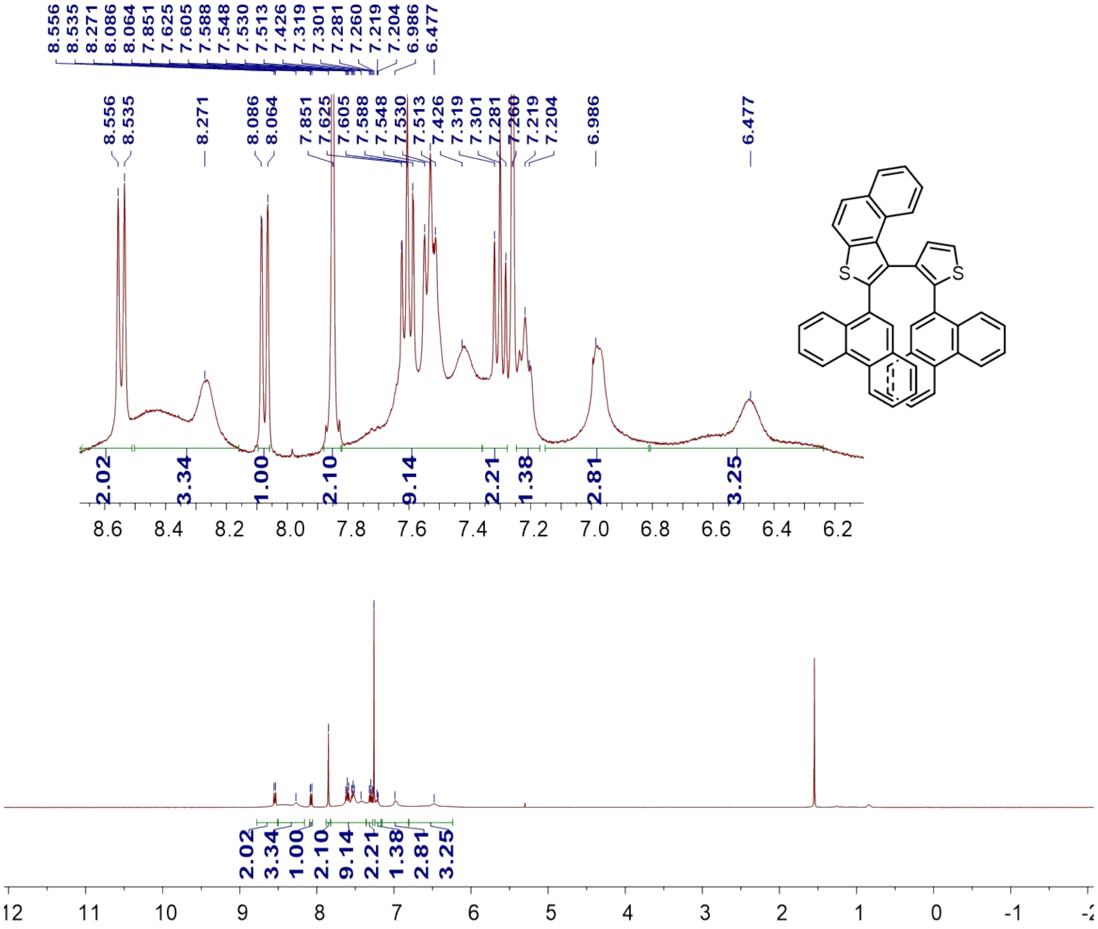

Figure S125. ^{1}H NMR (400 MHz, $CDCl_3$) spectrum of **5d**.

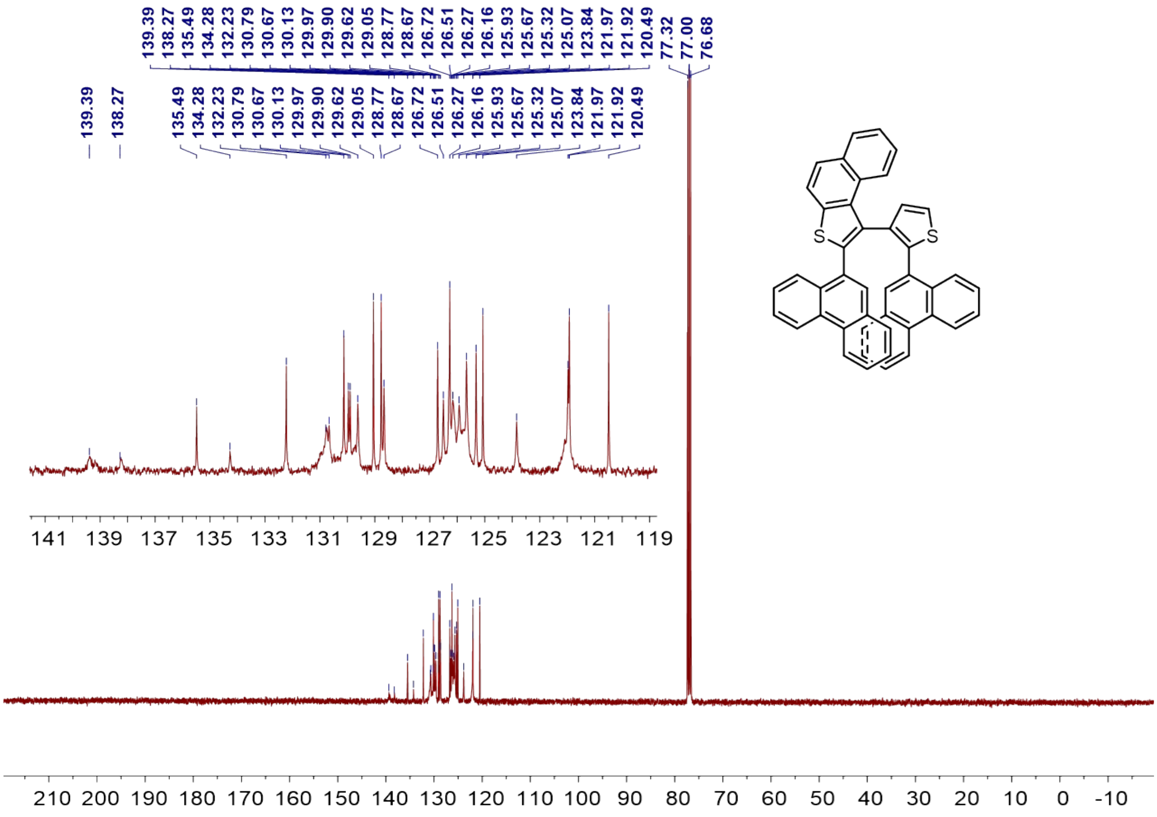

Figure S126. ^{13}C NMR (100 MHz, $CDCl_3$) spectrum of **5d**.

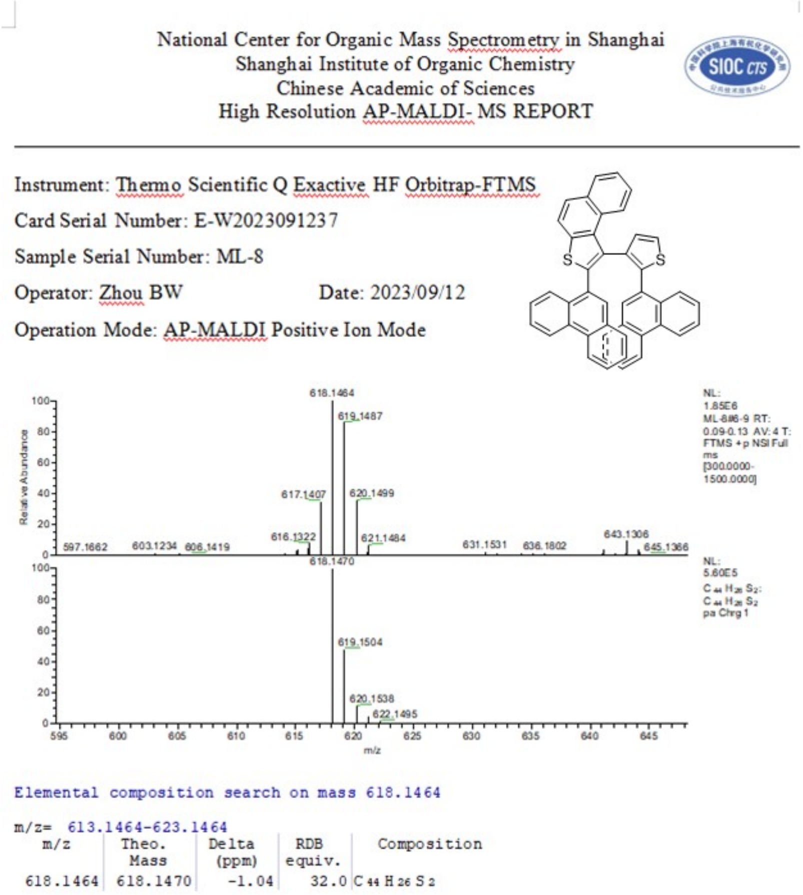
National Center for Organic Mass Spectrometry in Shanghai
Shanghai Institute of Organic Chemistry
Chinese Academy of Sciences
High Resolution AP-MALDI- MS REPORT

Instrument: Thermo Scientific Q Exactive HF Orbitrap-FTMS
Card Serial Number: E-W2023091237
Sample Serial Number: ML-8
Operator: Zhou BW Date: 2023/09/12
Operation Mode: AP-MALDI Positive Ion Mode

Elemental composition search on mass 618.1464

m/z= 613.1464-623.1464

m/z	Theo. Mass	Delta (ppm)	RDB equiv.	Composition
618.1464	618.1470	-1.04	32.0	$C_{44}H_{26}S_2$

Figure S127. HRMS-MALDI spectrum of **5d**.

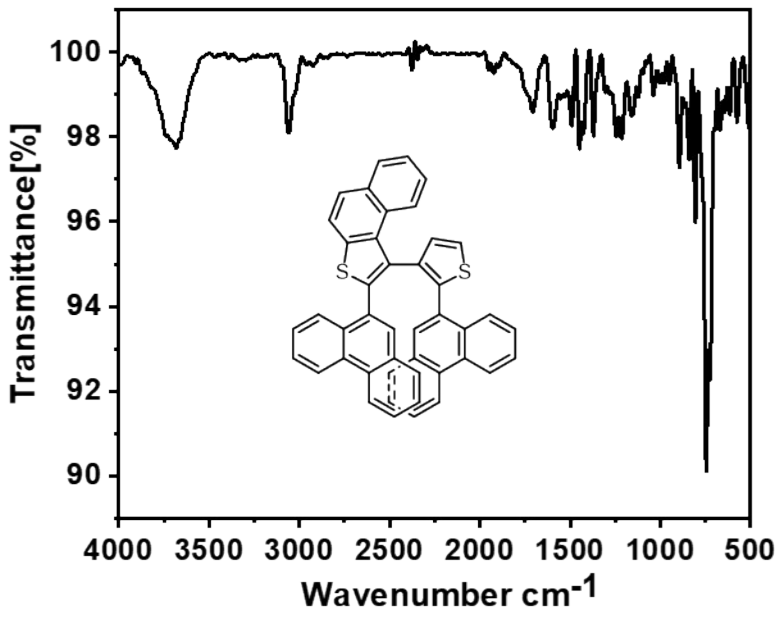

Figure S128. IR spectrum of **5d**.

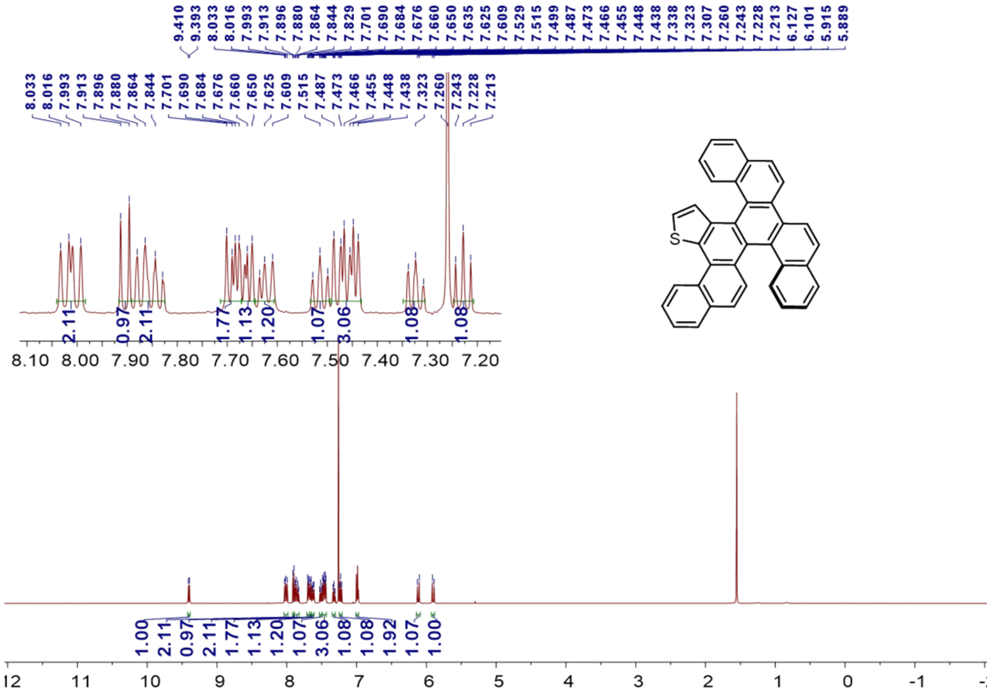

Figure S129. ^{1}H NMR (400 MHz, $CDCl_3$) spectrum of **6c**.

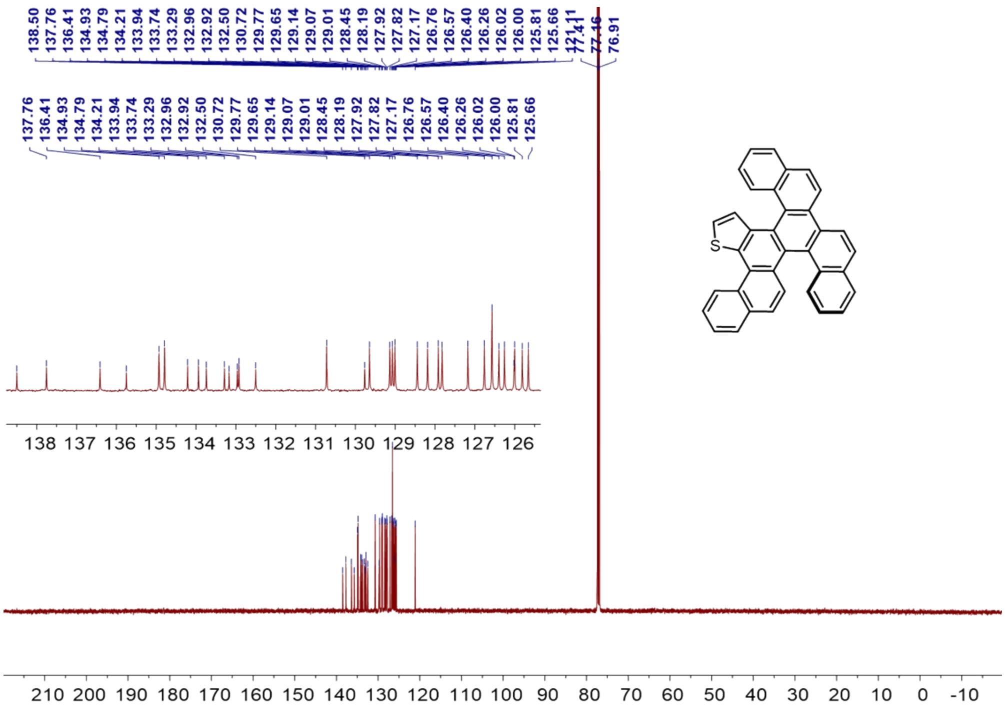

Figure S130. ^{13}C NMR (100 MHz, $CDCl_3$) spectrum of **6c**.

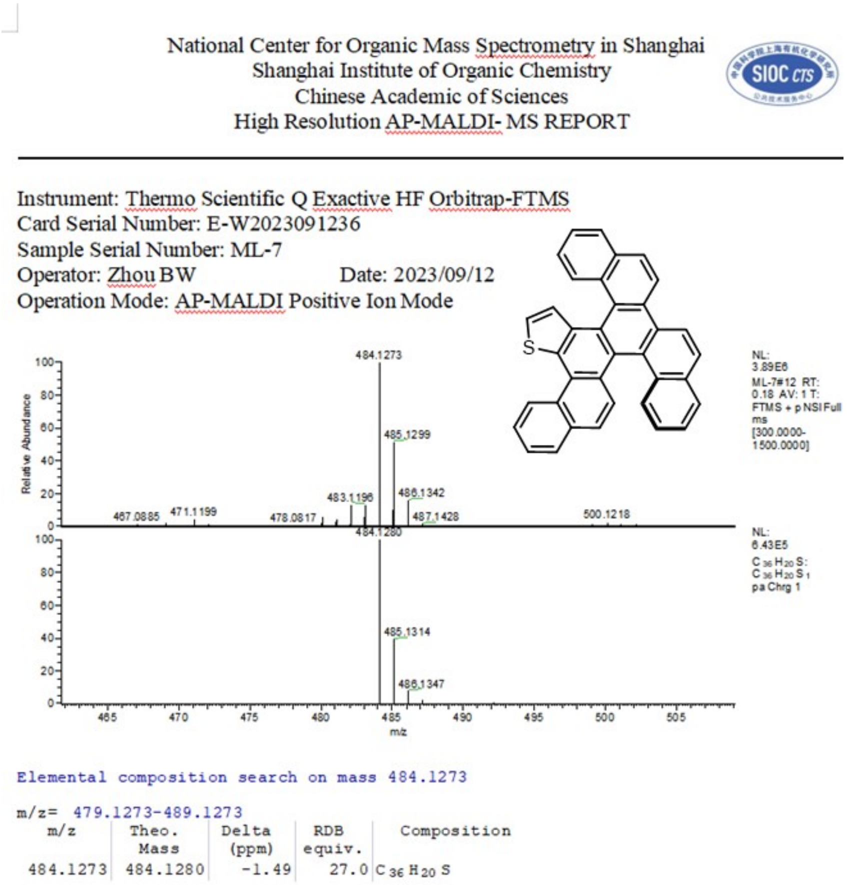

Figure S131. HRMS-MALDI spectrum of **6c**.

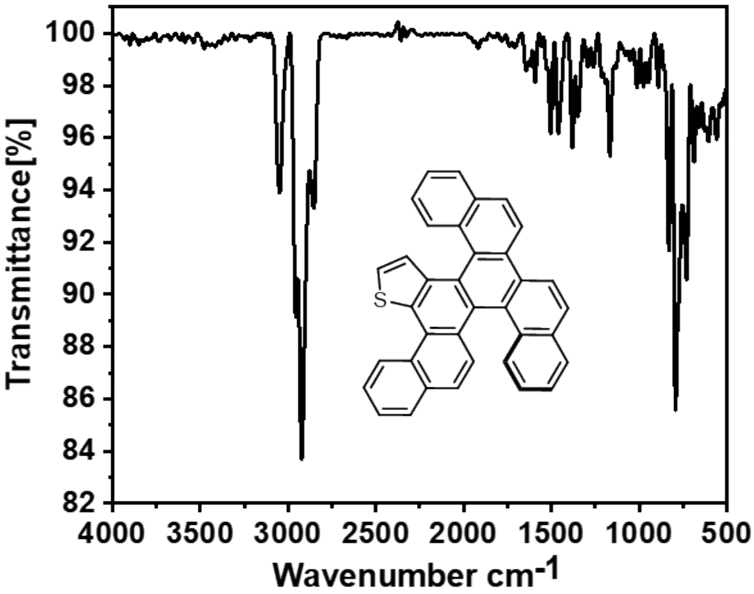

Figure S132. IR spectrum of **6c**.

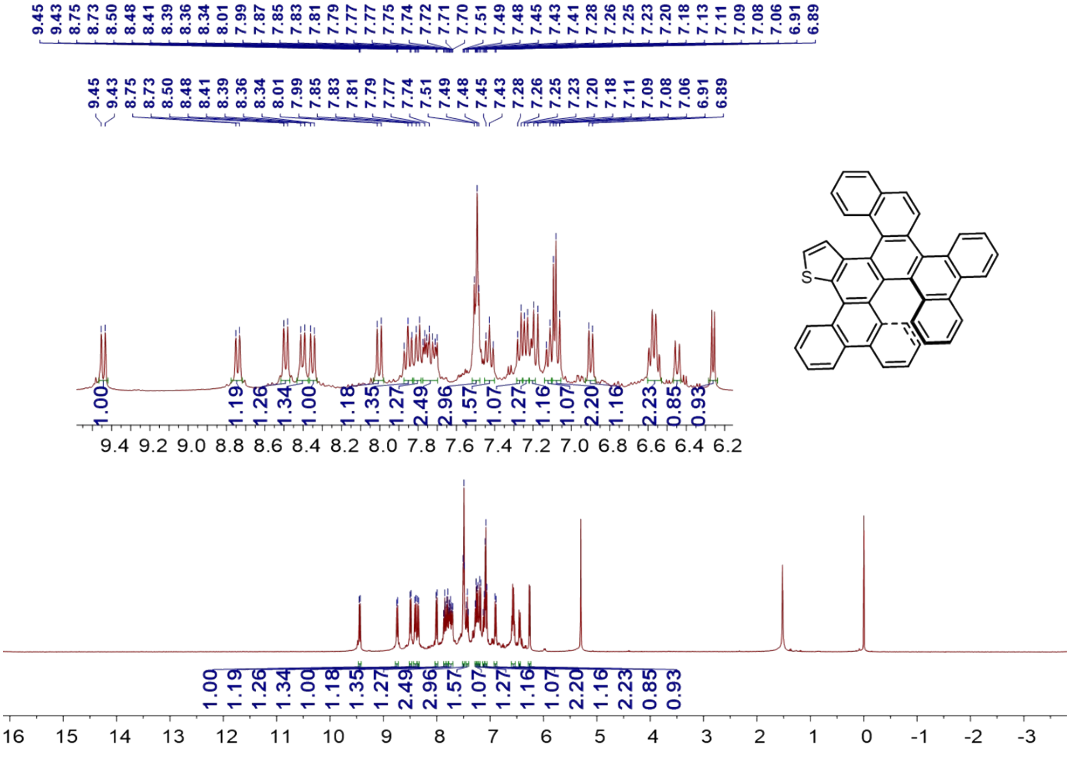

Figure S133. ^{1}H NMR (400 MHz, CD_2Cl_2) spectrum of **6d**.

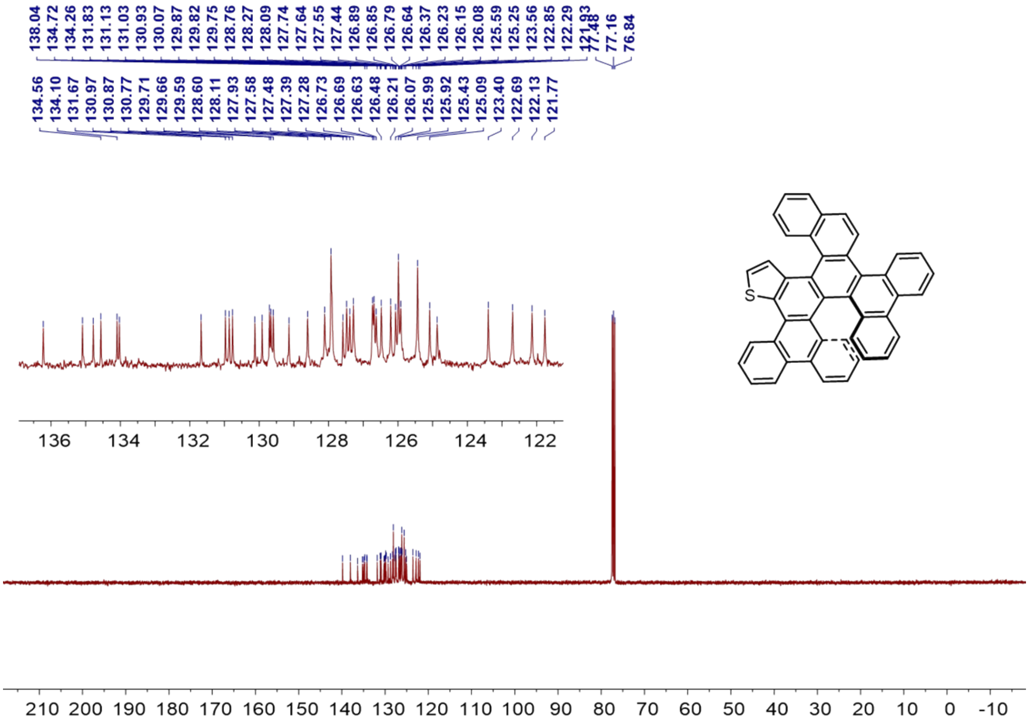

Figure S134. ^{13}C NMR (100 MHz, $CDCl_3$) spectrum of **6d**.

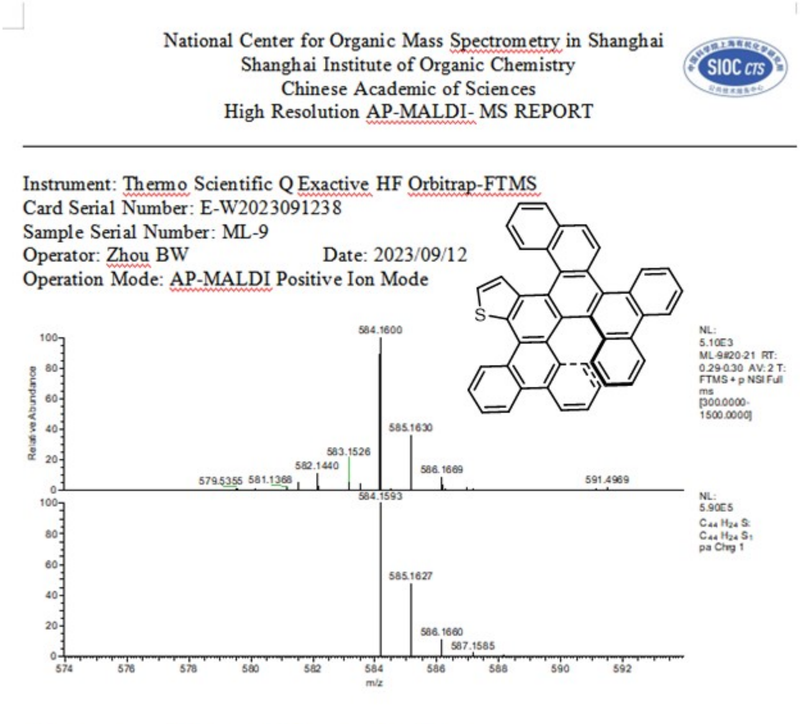

Elemental composition search on mass 584.1600

m/z= 579.1600-589.1600

m/z	Theo. Mass	Delta (ppm)	RDB equiv.	Composition
584.1600	584.1593	1.16	33.0	$C_{44}H_{24}S$

Figure S135. HRMS-MALDI spectrum of **6d**.

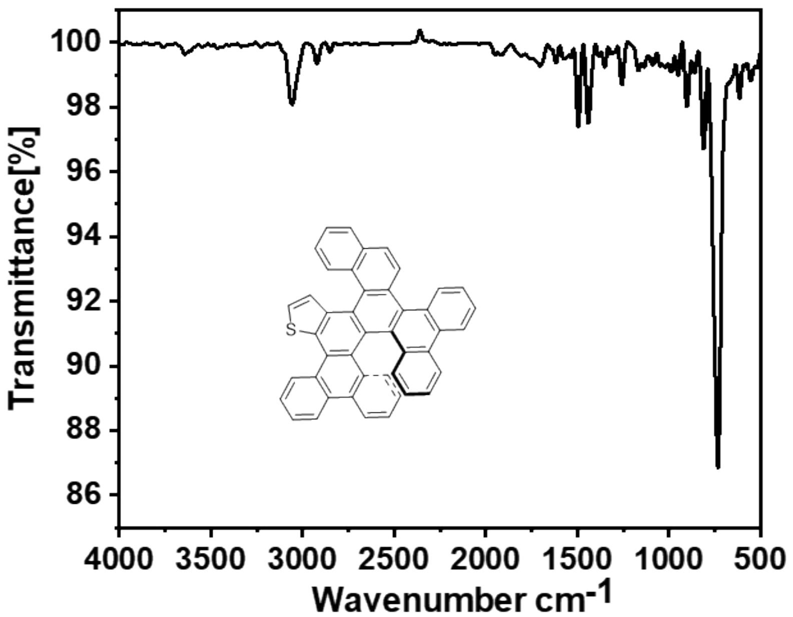

Figure S136. IR spectrum of **6d**.

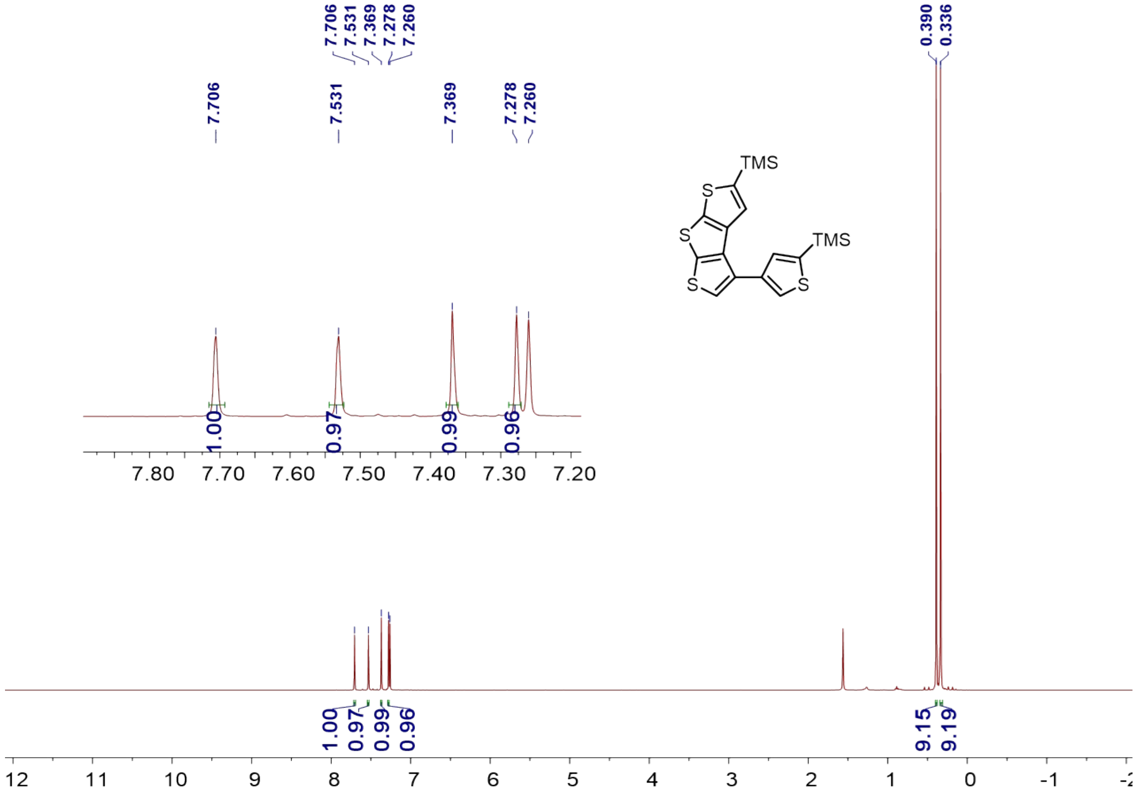

Figure S137. ^{1}H NMR (400 MHz, $CDCl_3$) spectrum of **15**.

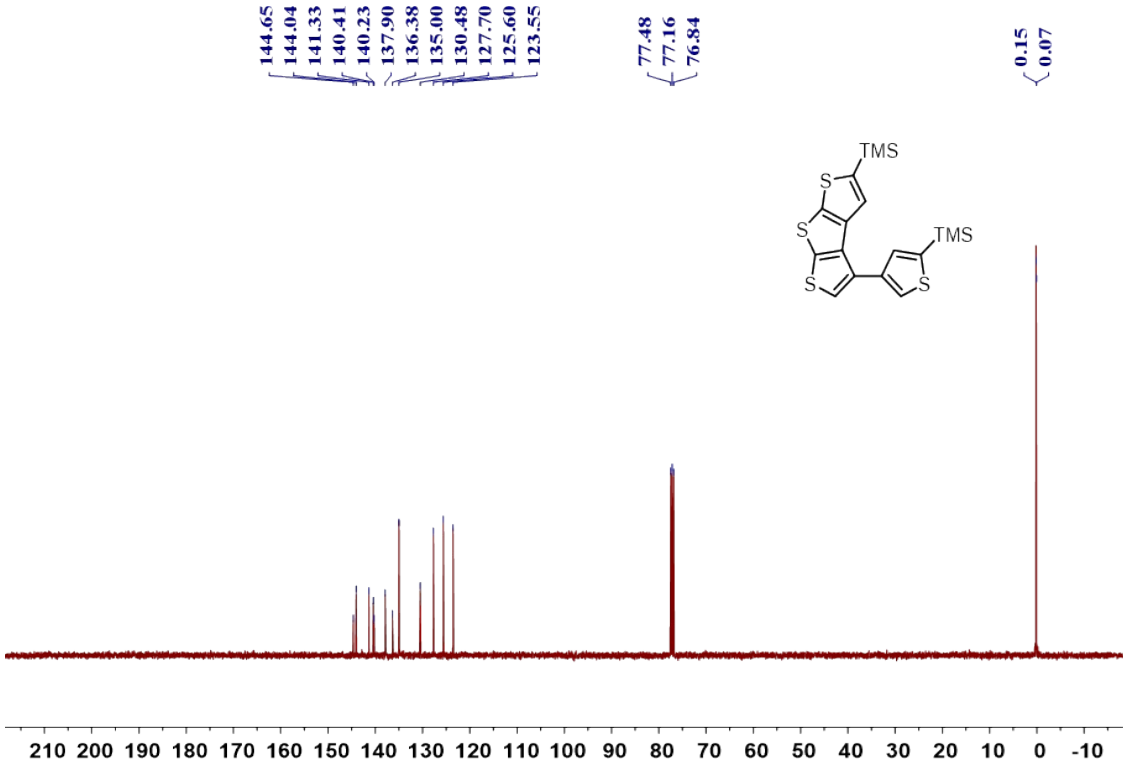

Figure S138. ^{13}C NMR (100 MHz, $CDCl_3$) spectrum of **15**.

National Center for Organic Mass Spectrometry in Shanghai
Shanghai Institute of Organic Chemistry
Chinese Academic of Sciences
High Resolution AP-MALDI- MS REPORT

Instrument: Thermo Scientific Q Exactive HF Orbitrap-FTMS

Card Serial Number: E-W2023051508

Sample Serial Number: ML-1

Operator: Wang HY   Date: 2023/05/15

Operation Mode: AP-MALDI Positive Ion Mode

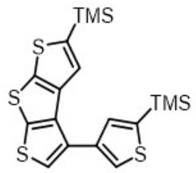

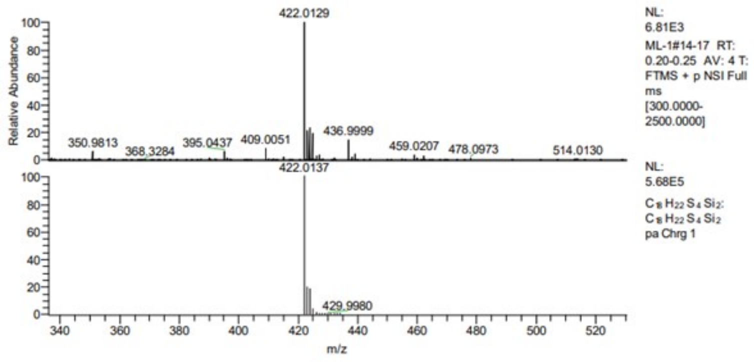

Elemental composition search on mass 422.01

m/z= 417.01-427.01

m/z	Theo. Mass	Delta (ppm)	RDB equiv.	Composition
422.0130	422.0137	-1.80	10.0	$C_{18}H_{22}S_4Si_2$

Figure S139. HRMS-MALDI spectrum of **15**.

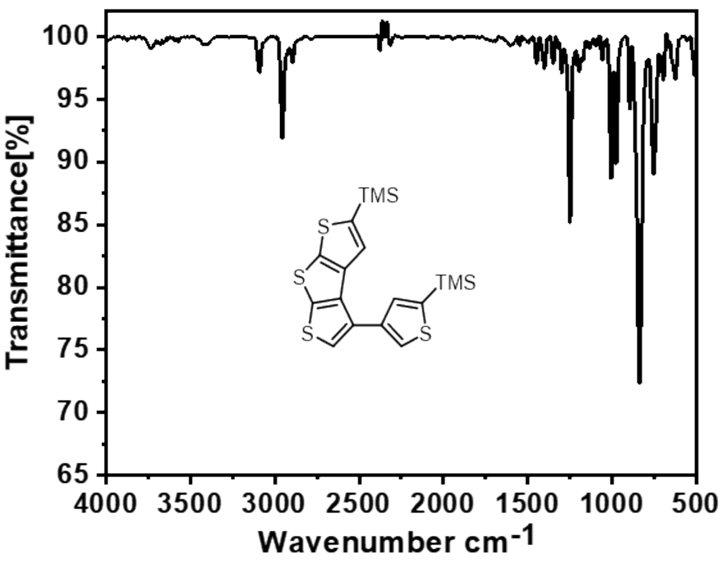

Figure S140. IR spectrum of **15**.

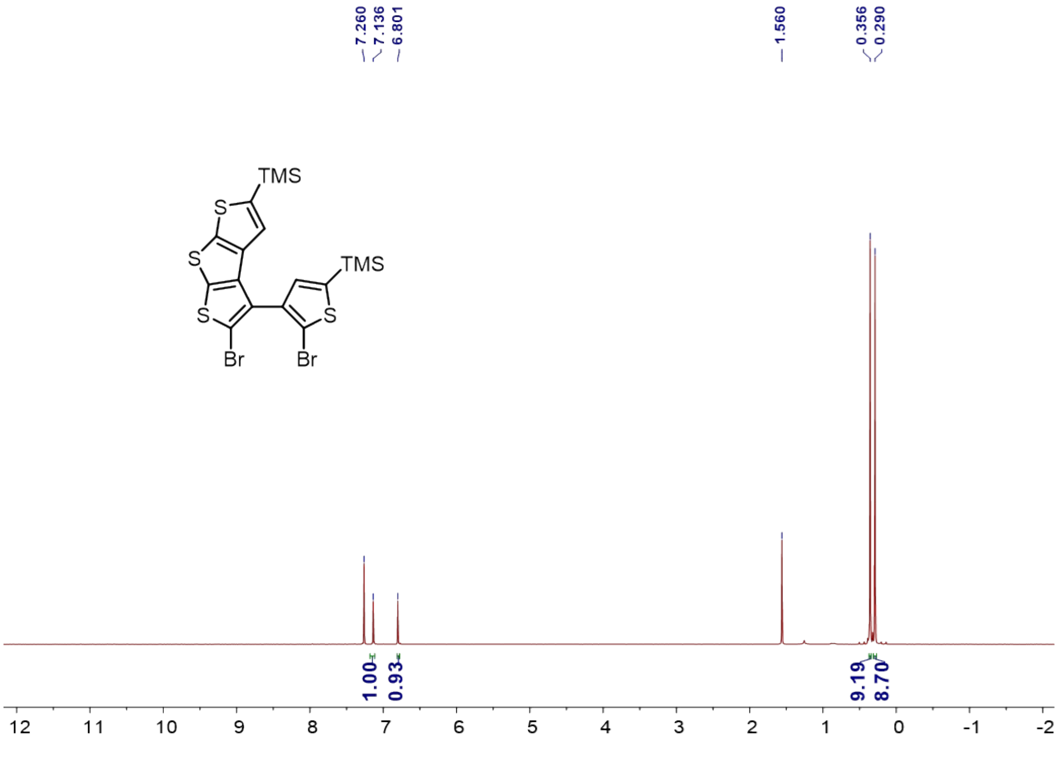

Figure S141. ^{1}H NMR (400 MHz, $CDCl_3$) spectrum of **16**.

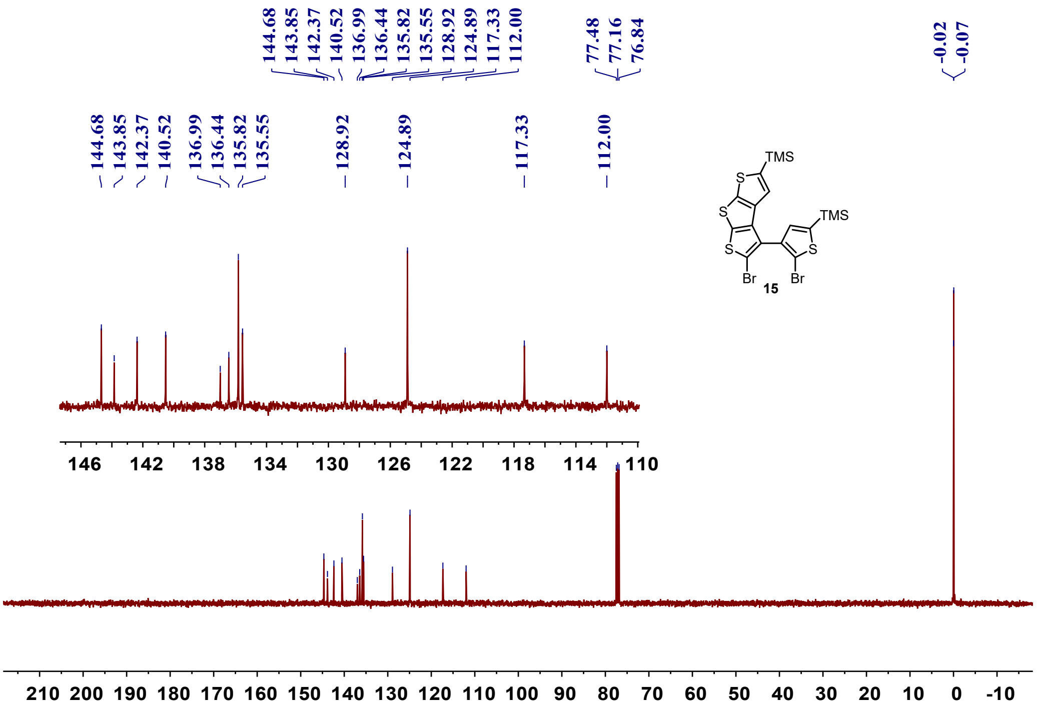

Figure S142. ^{13}C NMR (100 MHz, $CDCl_3$) spectrum of **16**.

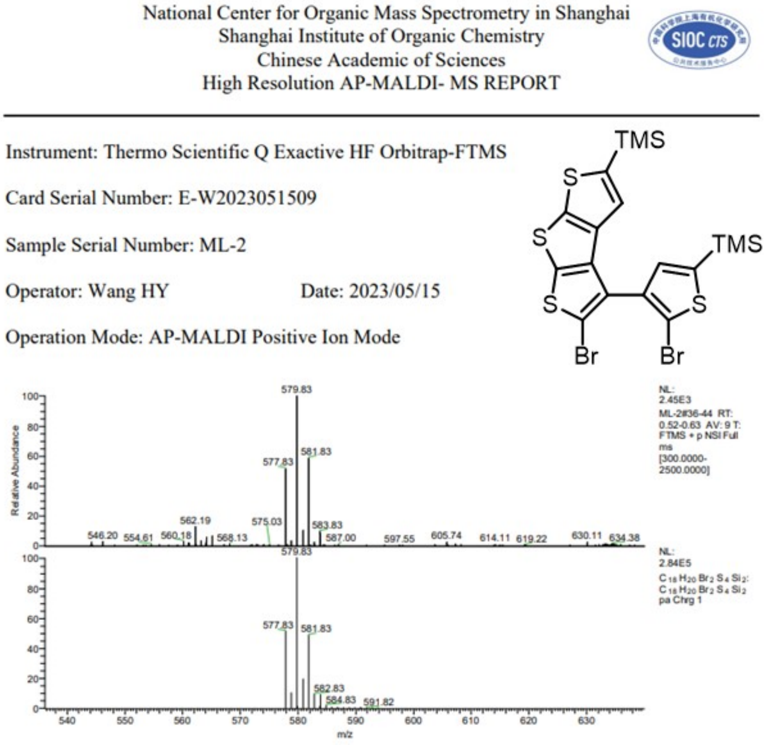

National Center for Organic Mass Spectrometry in Shanghai
Shanghai Institute of Organic Chemistry
Chinese Academic of Sciences
High Resolution AP-MALDI- MS REPORT

Instrument: Thermo Scientific Q Exactive HF Orbitrap-FTMS

Card Serial Number: E-W2023051509

Sample Serial Number: ML-2

Operator: Wang HY Date: 2023/05/15

Operation Mode: AP-MALDI Positive Ion Mode

Elemental composition search on mass 577.83

m/z= 572.83-582.83

m/z	Theo. Mass	Delta (ppm)	RDB equiv.	Composition
577.8335	577.8348	-2.22	10.0	$C_{18}H_{20}Br_2S_4Si_2$

Figure S143. HRMS-MALDI spectrum of **16**.

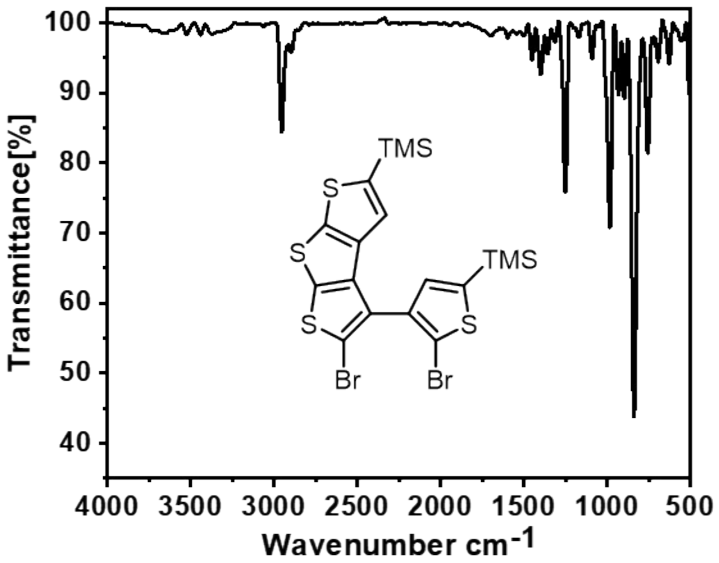

Figure S144. IR spectrum of **16**.

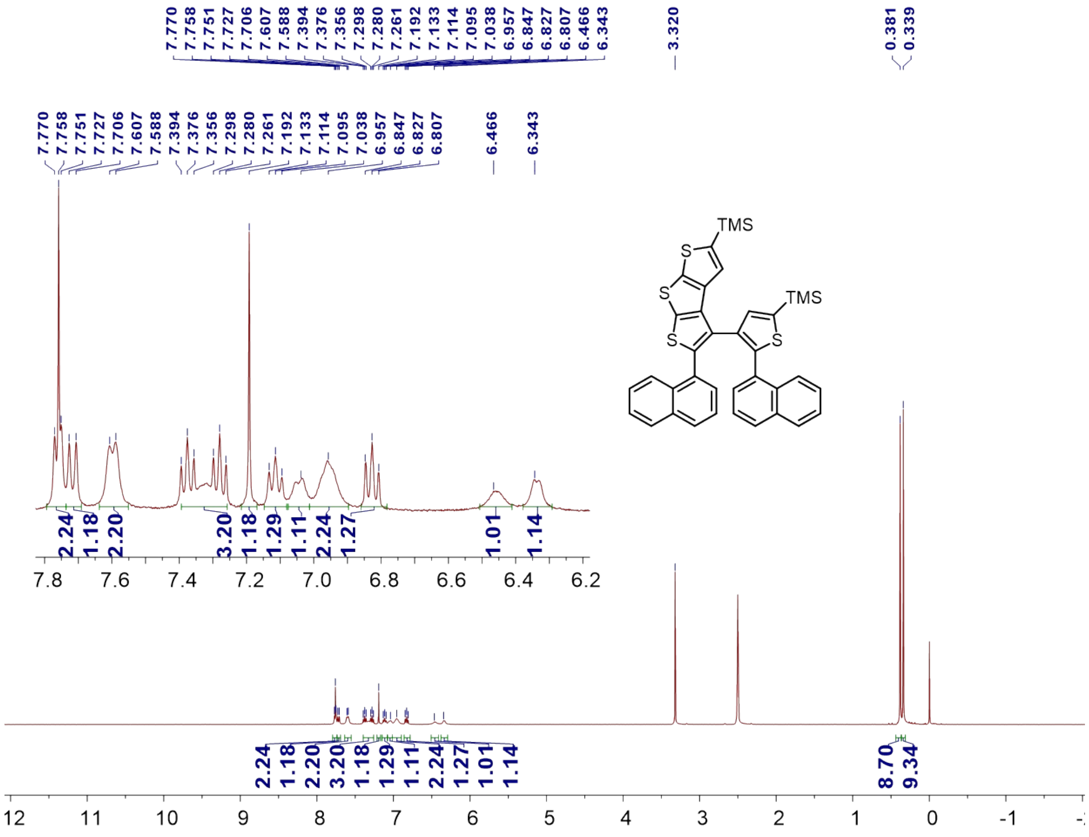

Figure S145. ^{1}H NMR (400 MHz, DMSO-d_6) spectrum of **5e**.

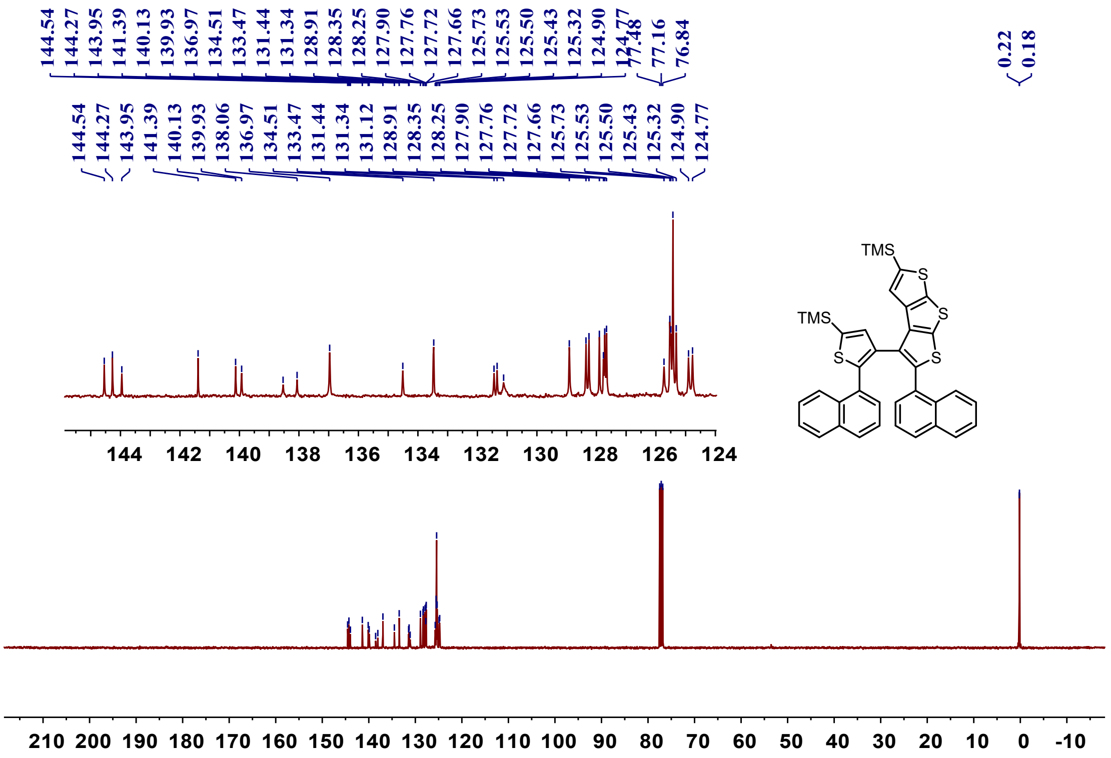

Figure S146. ^{13}C NMR (100 MHz, $CDCl_3$) spectrum of **5e**.

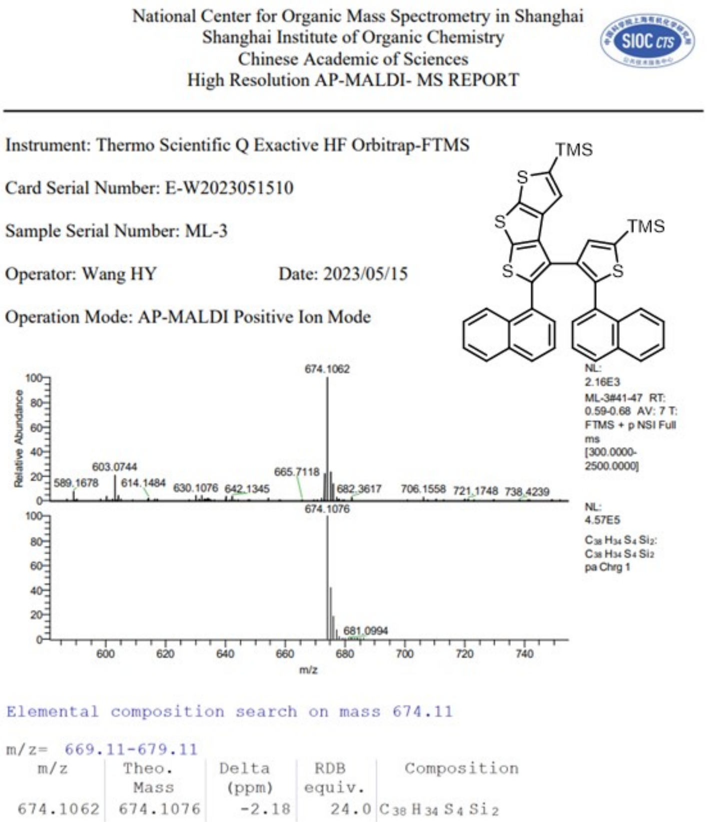
National Center for Organic Mass Spectrometry in Shanghai
Shanghai Institute of Organic Chemistry
Chinese Academic of Sciences
High Resolution AP-MALDI- MS REPORT

Instrument: Thermo Scientific Q Exactive HF Orbitrap-FTMS

Card Serial Number: E-W2023051510

Sample Serial Number: ML-3

Operator: Wang HY   Date: 2023/05/15

Operation Mode: AP-MALDI Positive Ion Mode

Elemental composition search on mass 674.11

m/z= 669.11-679.11

m/z	Theo. Mass	Delta (ppm)	RDB equiv.	Composition
674.1062	674.1076	-2.18	24.0	$C_{38}H_{34}S_4Si_2$

Figure S147. HRMS-MALDI spectrum of **5e**.

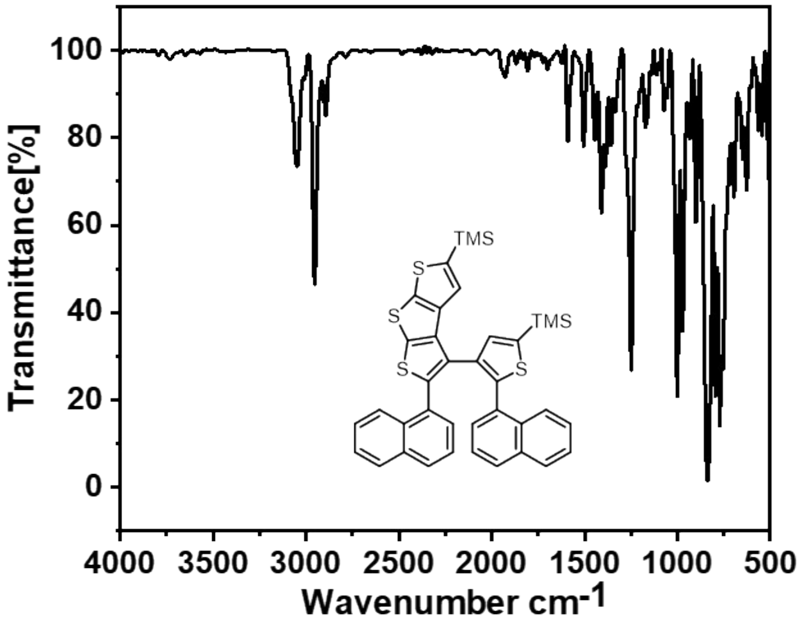

Figure S148. IR spectrum of **5e**.

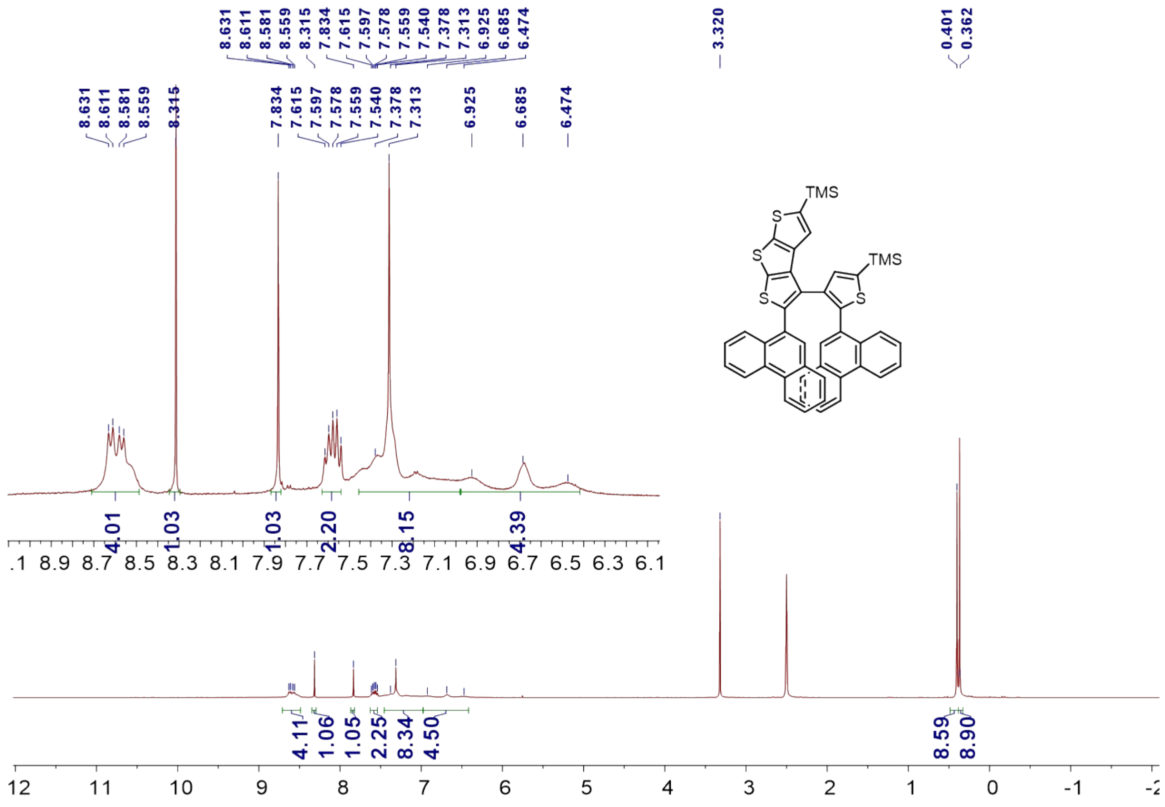

Figure S149. ^{1}H NMR (400 MHz, DMSO-d_6) spectrum of **5f**.

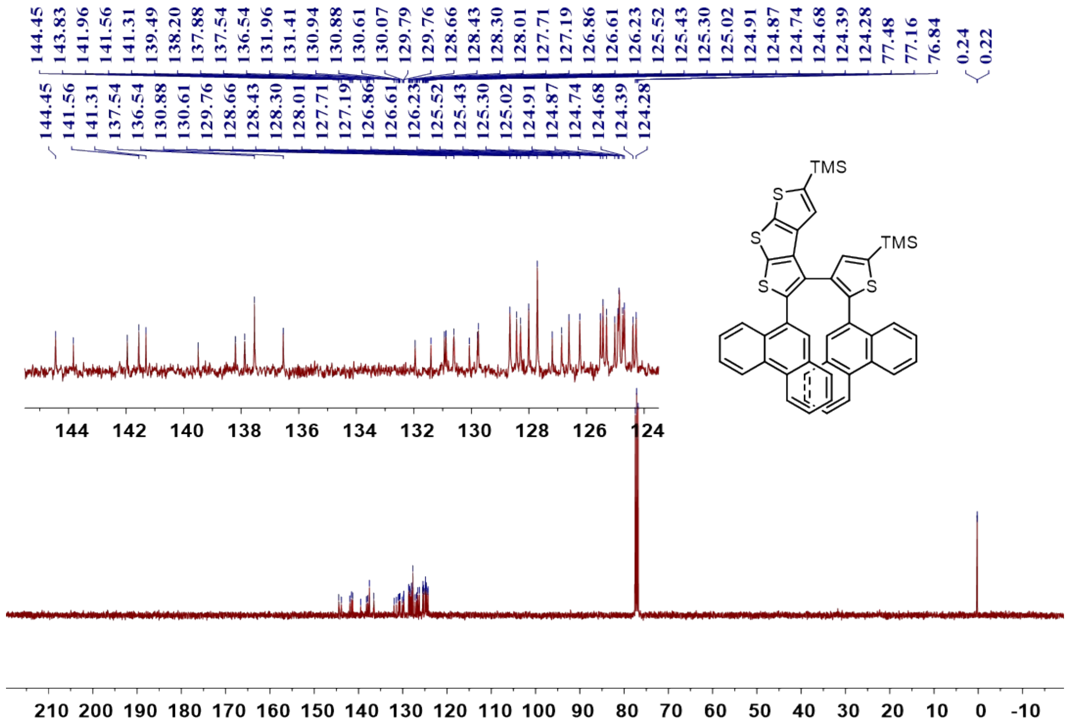

Figure S150. ^{13}C NMR (100 MHz, $CDCl_3$) spectrum of **5f**.

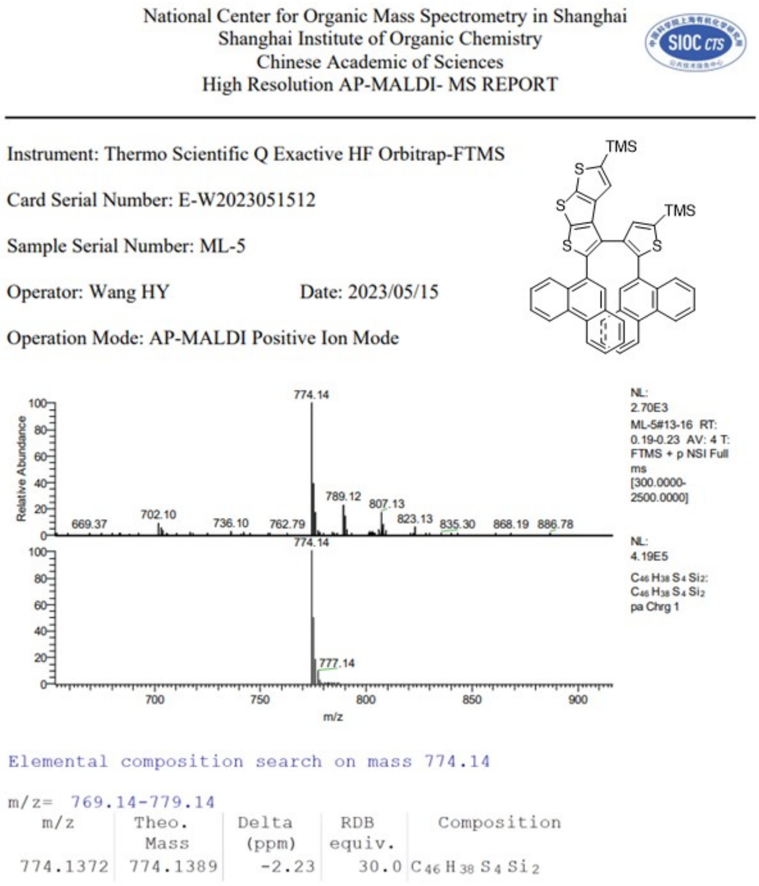

National Center for Organic Mass Spectrometry in Shanghai
Shanghai Institute of Organic Chemistry
Chinese Academic of Sciences
High Resolution AP-MALDI- MS REPORT

Instrument: Thermo Scientific Q Exactive HF Orbitrap-FTMS

Card Serial Number: E-W2023051512

Sample Serial Number: ML-5

Operator: Wang HY   Date: 2023/05/15

Operation Mode: AP-MALDI Positive Ion Mode

Elemental composition search on mass 774.14

m/z= 769.14-779.14

m/z	Theo. Mass	Delta (ppm)	RDB equiv.	Composition
774.1372	774.1389	-2.23	30.0	$C_{46}H_{38}S_4Si_2$

Figure S151. HRMS-MALDI spectrum of **5f**.

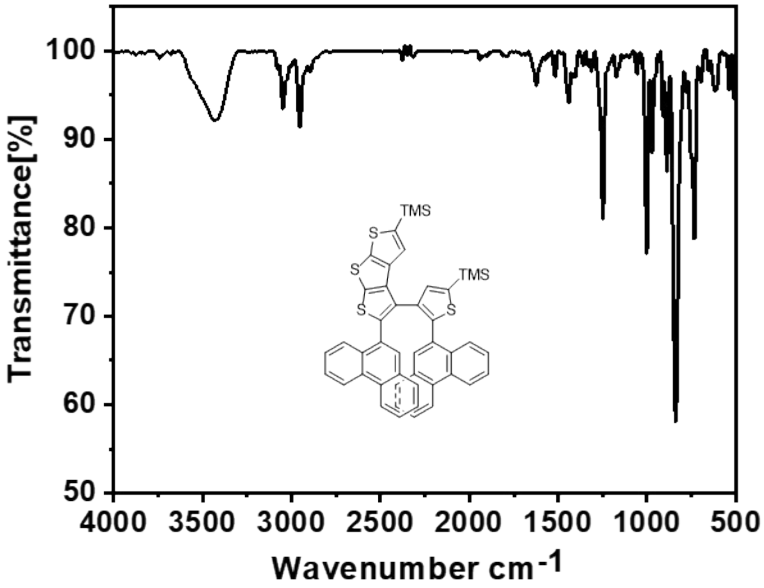

Figure S152. IR spectrum of **5f**.

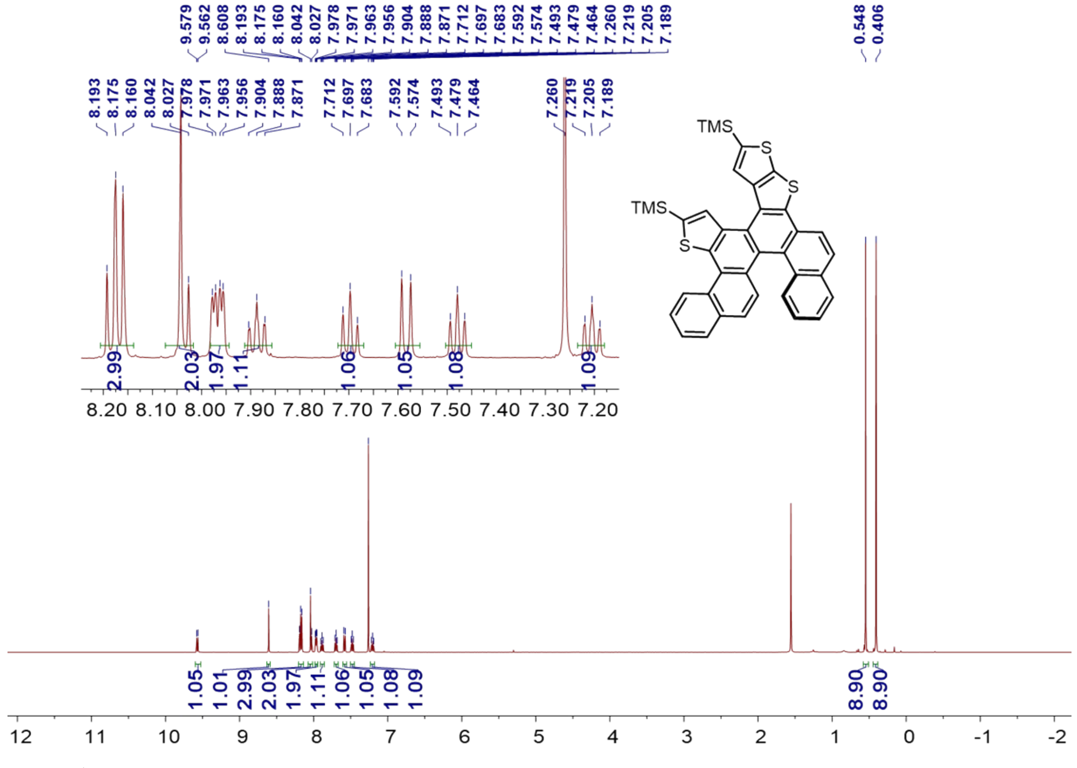

Figure S153. ^{1}H NMR (400 MHz, $CDCl_3$) spectrum of **6e**.

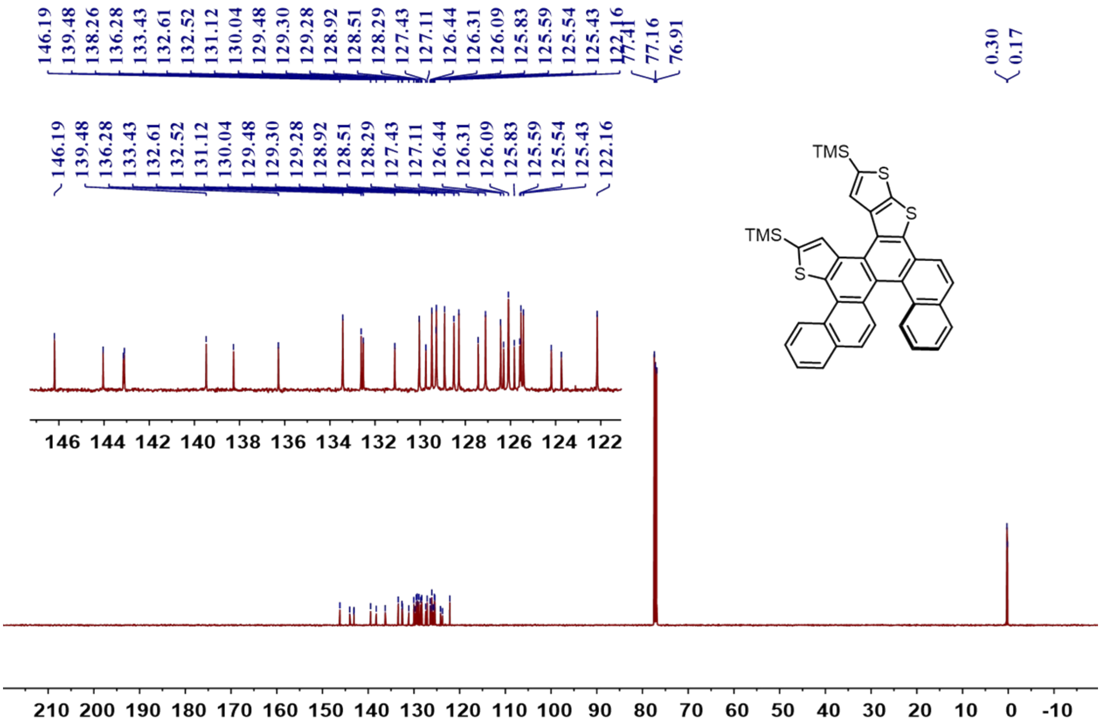

Figure S154. ^{13}C NMR (100 MHz, $CDCl_3$) spectrum of **6e**.

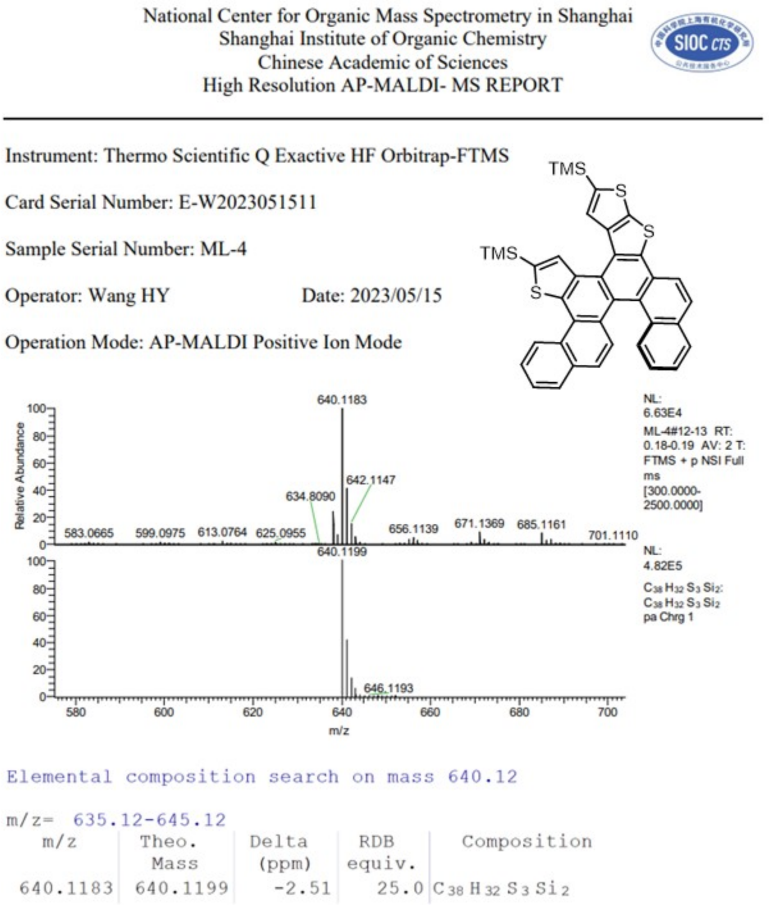

Figure S155. HRMS-MALDI spectrum of **6e**.

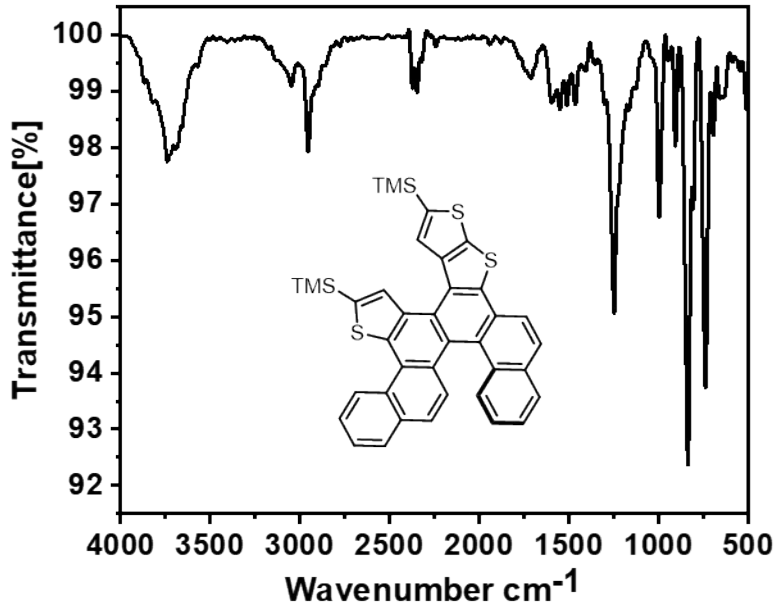

Figure S156. IR spectrum of **6e**.

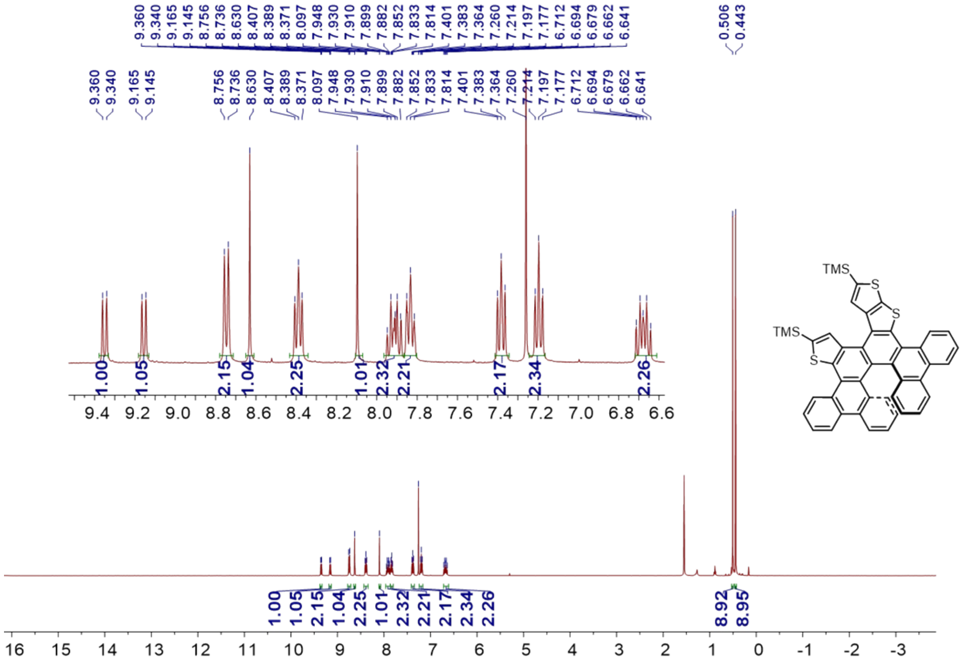

Figure S157. ^{1}H NMR (400 MHz, $CDCl_3$) spectrum of **6f**.

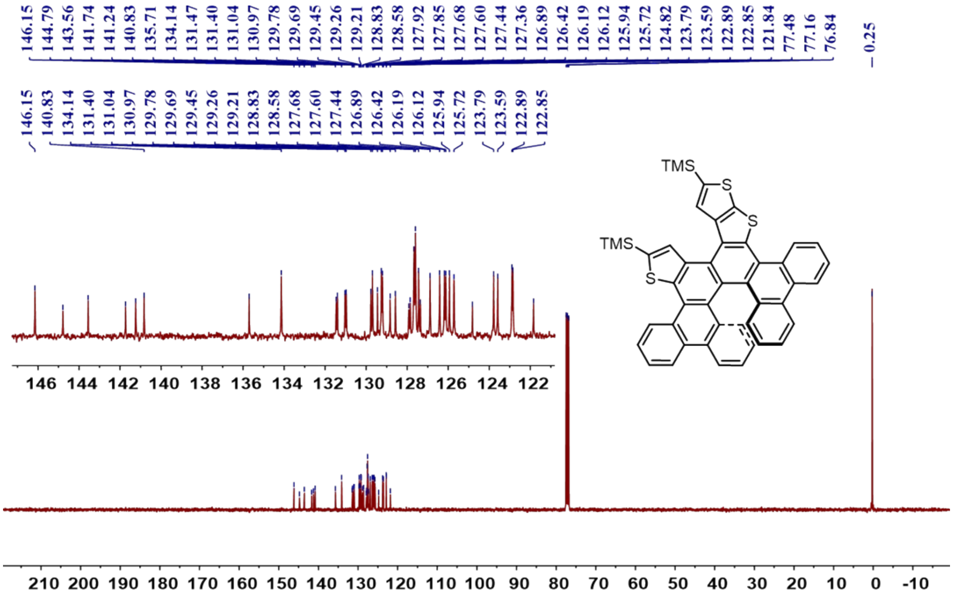

Figure S158. ^{13}C NMR (100 MHz, $CDCl_3$) spectrum of **6f**.

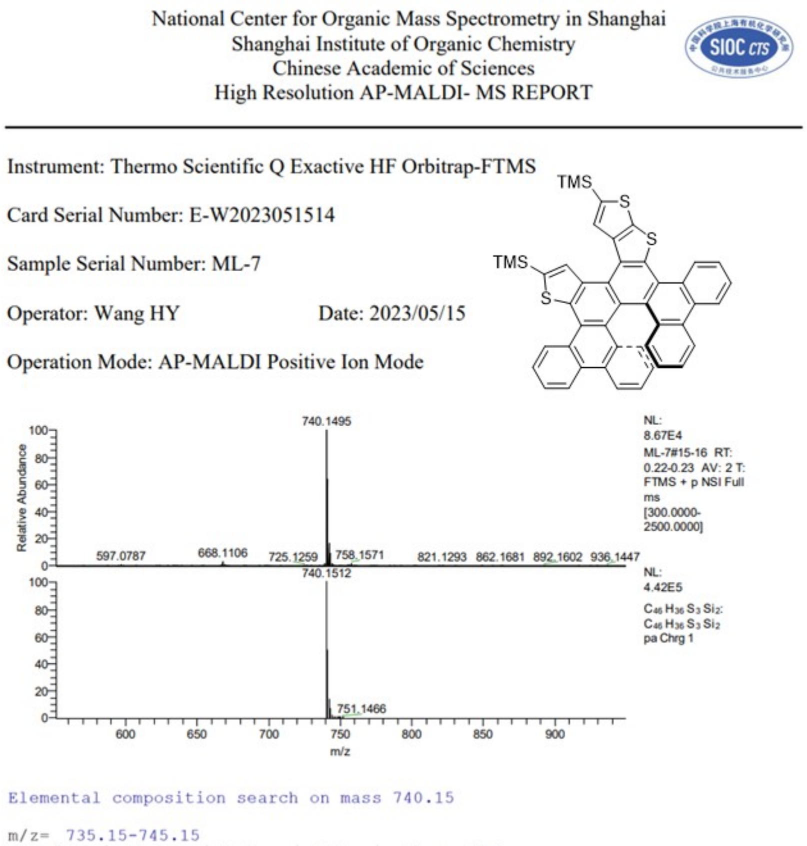

m/z	Theo. Mass	Delta (ppm)	RDB equiv.	Composition
740.1495	740.1512	-2.29	31.0	$C_{46}H_{36}S_3Si_2$

Figure S159. HRMS-MALDI spectrum of **6f**.

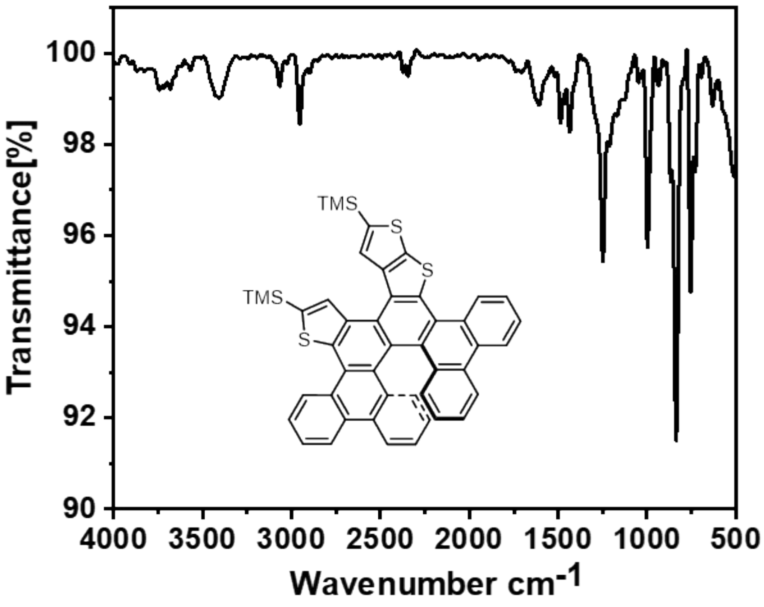

Figure S160. IR spectrum of **6f**.

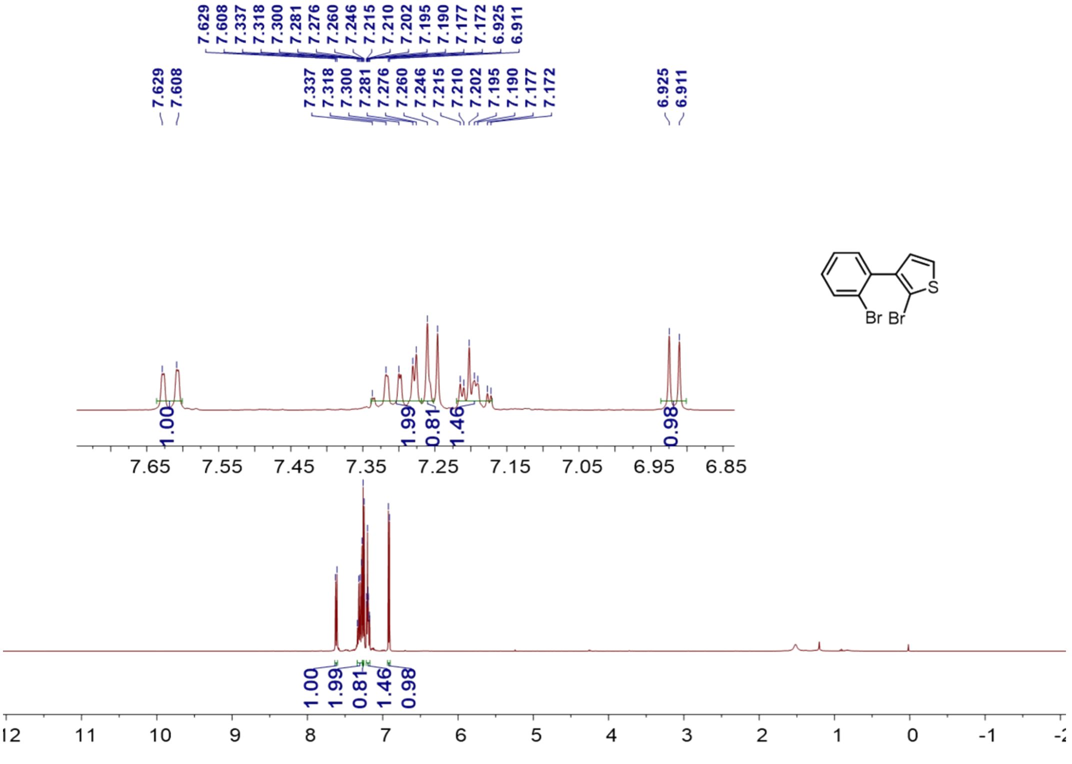

Figure S161. ^{1}H NMR (400 MHz, $CDCl_3$) spectrum of **18**.

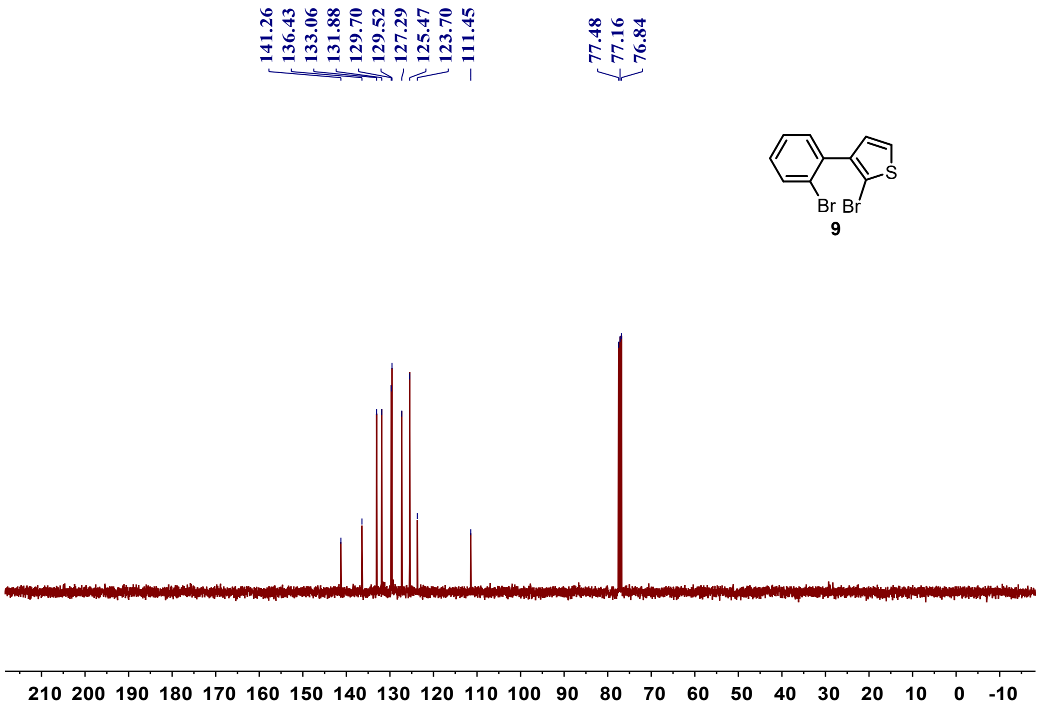

Figure S162. ^{13}C NMR (100 MHz, $CDCl_3$) spectrum of **18**.

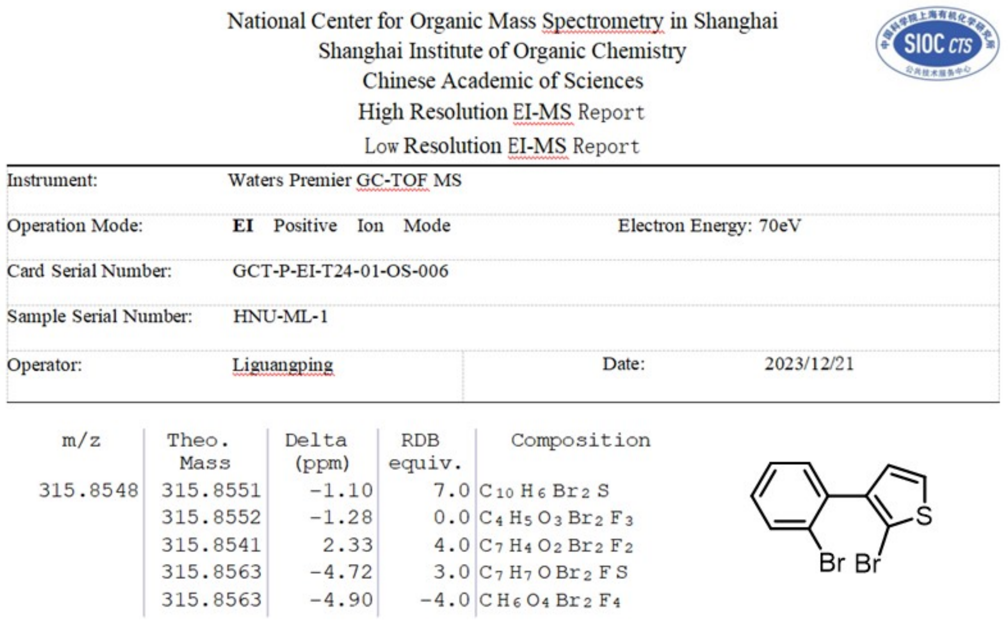

National Center for Organic Mass Spectrometry in Shanghai
Shanghai Institute of Organic Chemistry
Chinese Academic of Sciences
High Resolution EI-MS Report
Low Resolution EI-MS Report

Instrument:	Waters Premier GC-TOF MS		
Operation Mode:	EI Positive Ion Mode	Electron Energy: 70eV	
Card Serial Number:	GCT-P-EI-T24-01-OS-006		
Sample Serial Number:	HNU-ML-1		
Operator:	Liguangping	Date:	2023/12/21

m/z	Theo. Mass	Delta (ppm)	RDB equiv.	Composition
315.8548	315.8551	-1.10	7.0	$C_{10}H_6Br_2S$
	315.8552	-1.28	0.0	$C_4H_5O_3Br_2F_3$
	315.8541	2.33	4.0	$C_7H_4O_2Br_2F_2$
	315.8563	-4.72	3.0	$C_7H_7OBr_2FS$
	315.8563	-4.90	-4.0	$CH_6O_4Br_2F_4$

Figure S163. HRMS-EI spectrum of **18**.

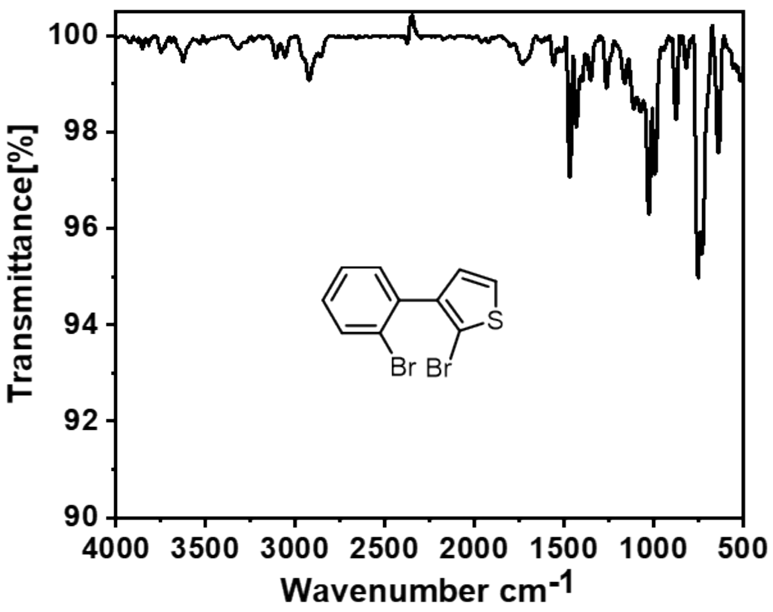

Figure S164. IR spectrum of **18**.

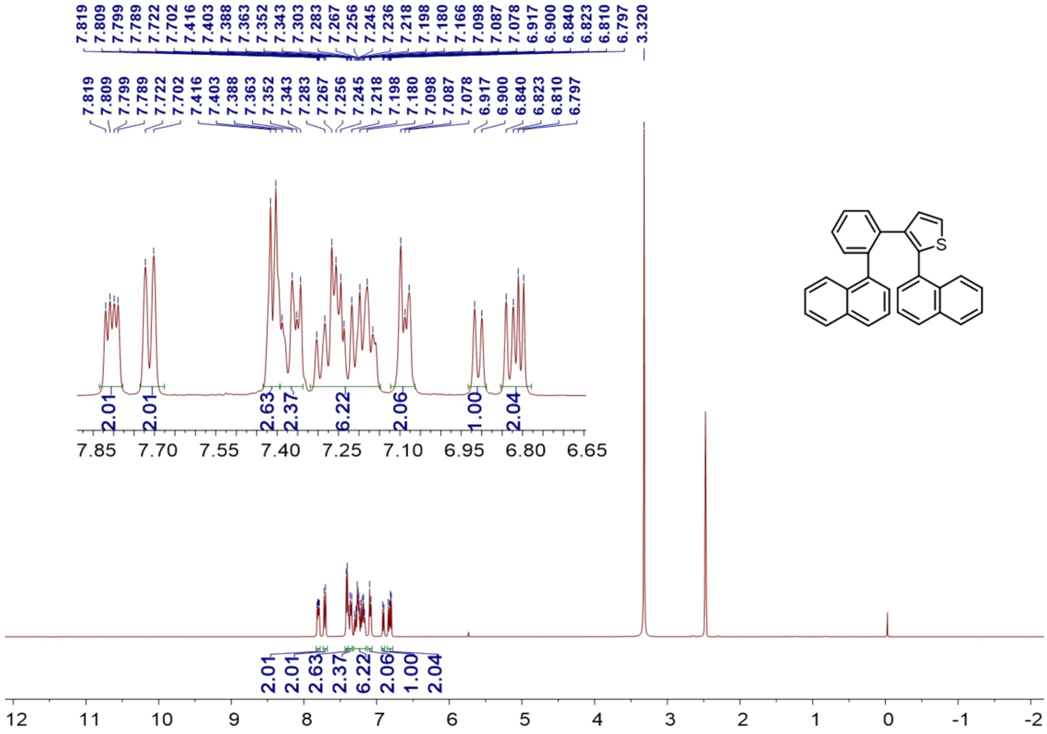

Figure S165. ^{1}H NMR (400 MHz, DMSO-d_6) spectrum of **7b**.

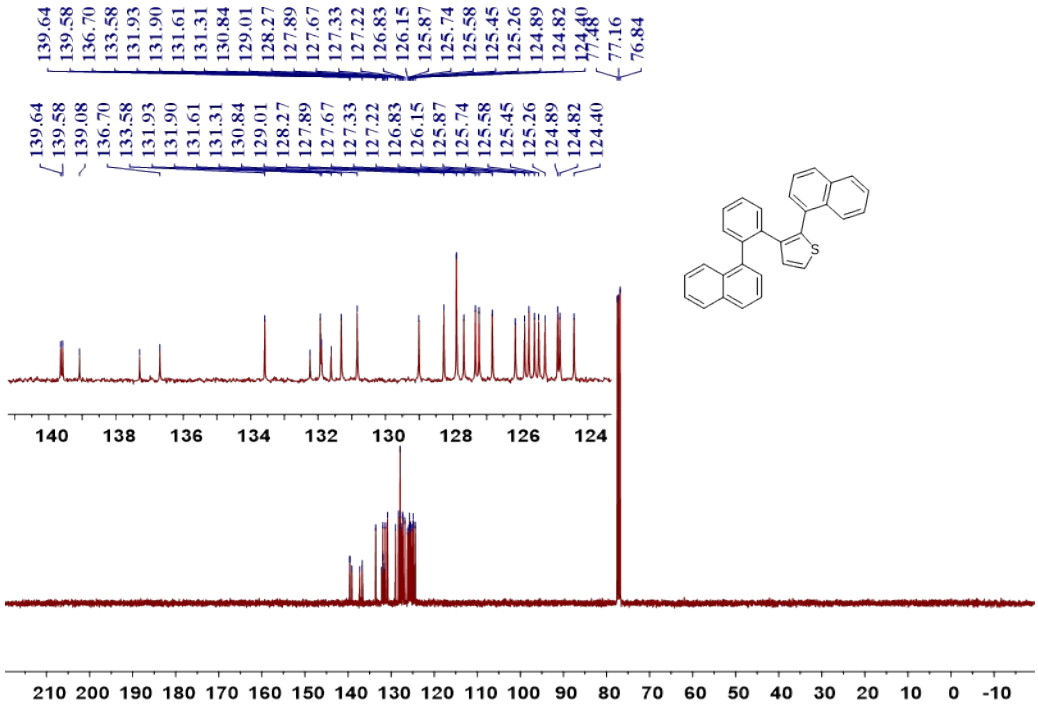

Figure S166. ^{13}C NMR (100 MHz, $CDCl_3$) spectrum of **7b**.

National Center for Organic Mass Spectrometry in Shanghai
Shanghai Institute of Organic Chemistry
Chinese Academic of Sciences
High Resolution AP-MALDI- MS REPORT

Instrument: Thermo Scientific Q Exactive HF Orbitrap-FTMS
Card Serial Number: E-W2023091239
Sample Serial Number: ML-10
Operator: Zhou BW Date: 2023/09/12
Operation Mode: AP-MALDI Positive Ion Mode

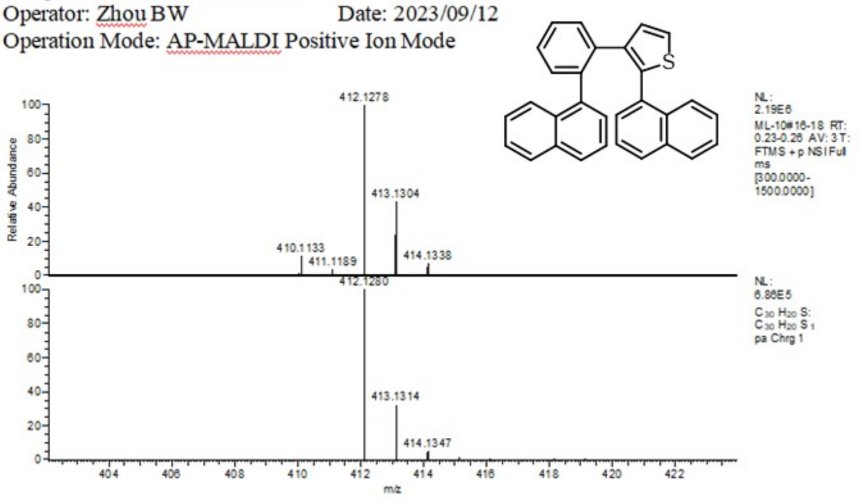

Elemental composition search on mass 412.1278

m/z= 407.1278-417.1278

m/z	Theo. Mass	Delta (ppm)	RDB equiv.	Composition
412.1278	412.1280	-0.54	21.0	$C_{30}H_{20}S$

Figure S167. HRMS-MALDI spectrum of **7b**.

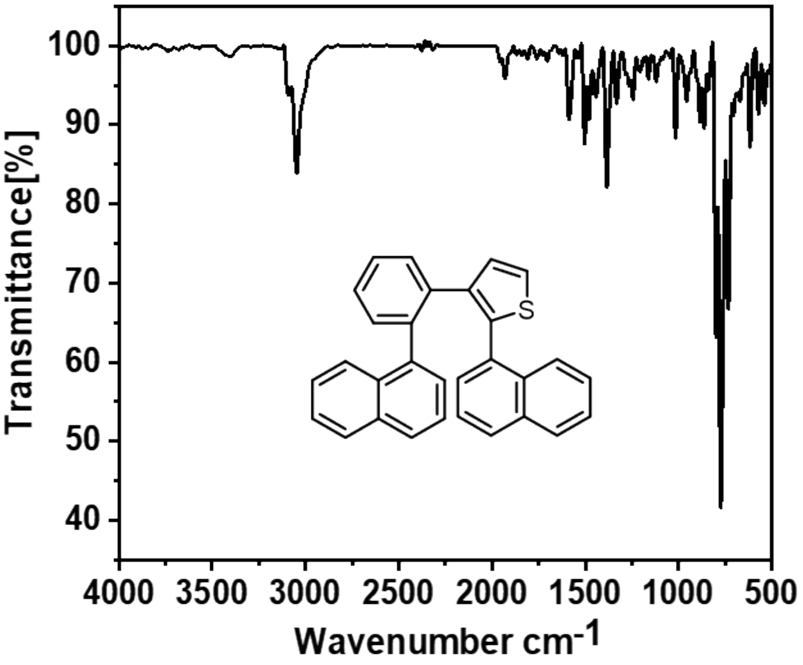

Figure S168. IR spectrum of **7b**.

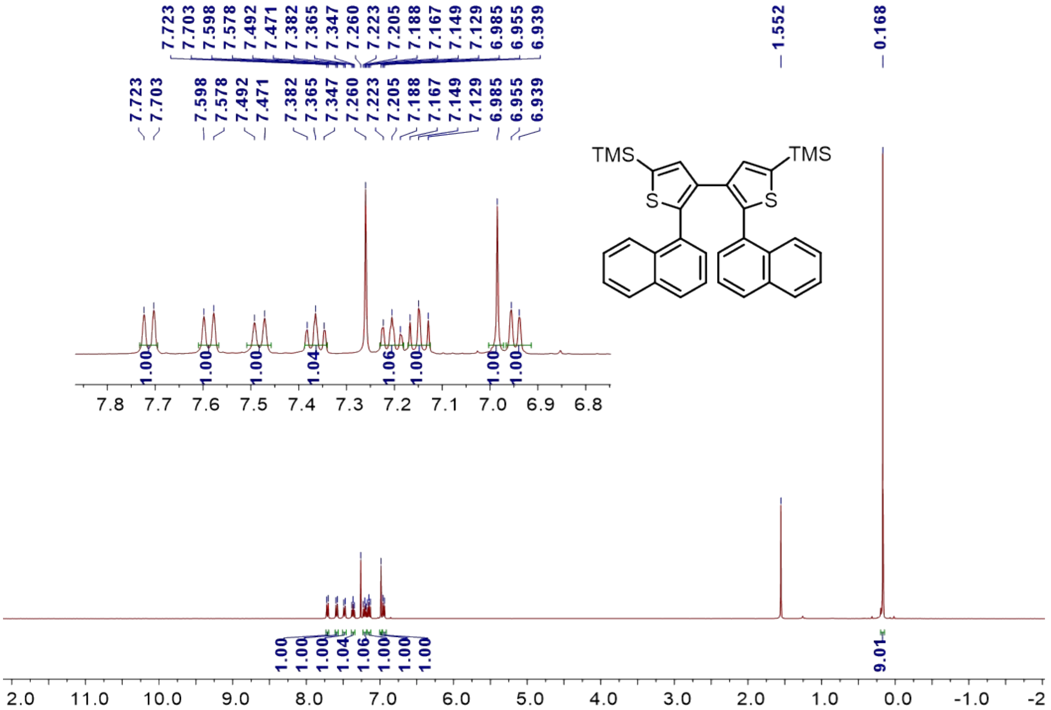

Figure S169. ^{1}H NMR (400 MHz, CDCl$_3$) spectrum of **7c**.

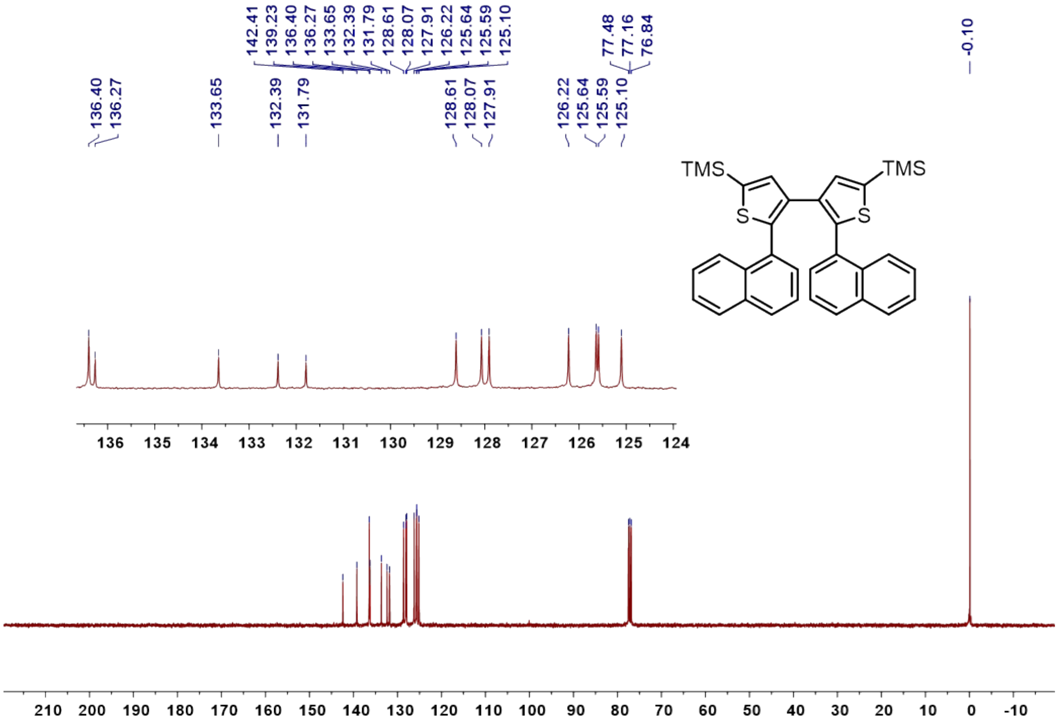

Figure S170. ^{13}C NMR (100 MHz, $CDCl_3$) spectrum of **7c**.

National Center for Organic Mass Spectrometry in Shanghai
Shanghai Institute of Organic Chemistry
Chinese Academic of Sciences
High Resolution AP-MALDI- MS REPORT

Instrument: Thermo Scientific Q Exactive HF Orbitrap-FTMS
Card Serial Number: E-W2023091231
Sample Serial Number: ML-1
Operator: Zhou BW   Date: 2023/09/12
Operation Mode: AP-MALDI Positive Ion Mode

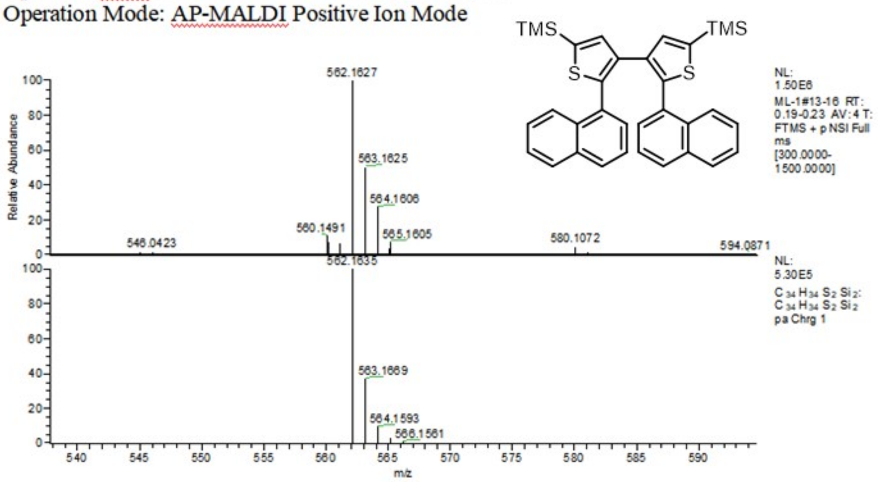

Elemental composition search on mass 562.1627

m/z= 557.1627-567.1627

m/z	Theo. Mass	Delta (ppm)	RDB equiv.	Composition
562.1627	562.1635	-1.42	20.0	$C_{34}H_{34}S_2Si_2$

Figure S171. HRMS-MALDI spectrum of **7c**.

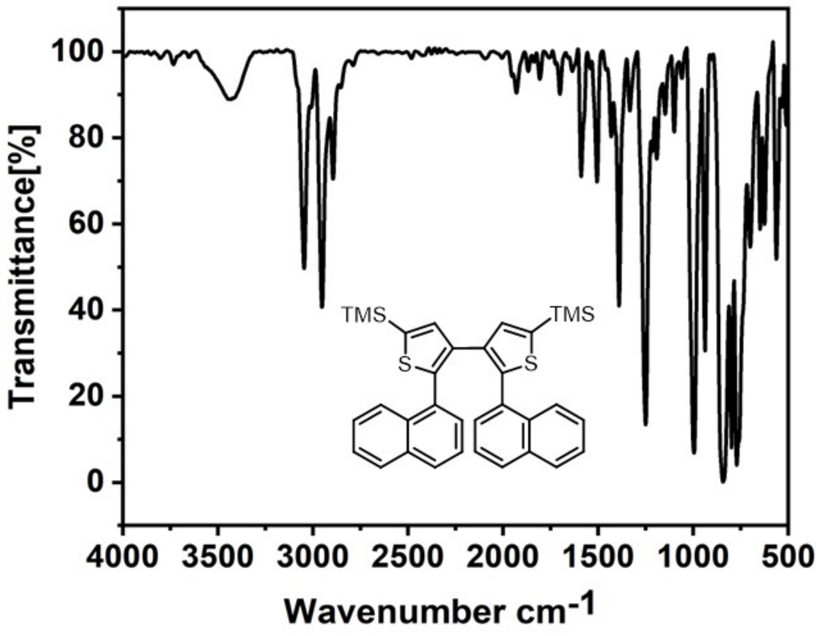

Figure S172. IR spectrum of **7c**.

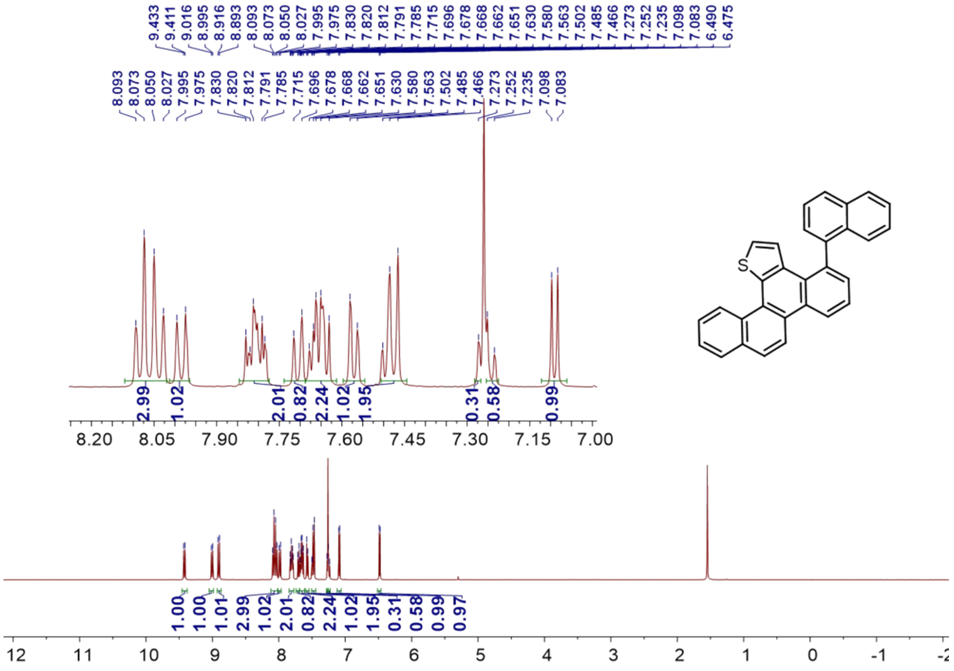

Figure S173. ^{1}H NMR (400 MHz, $CDCl_3$) spectrum of **8b**.

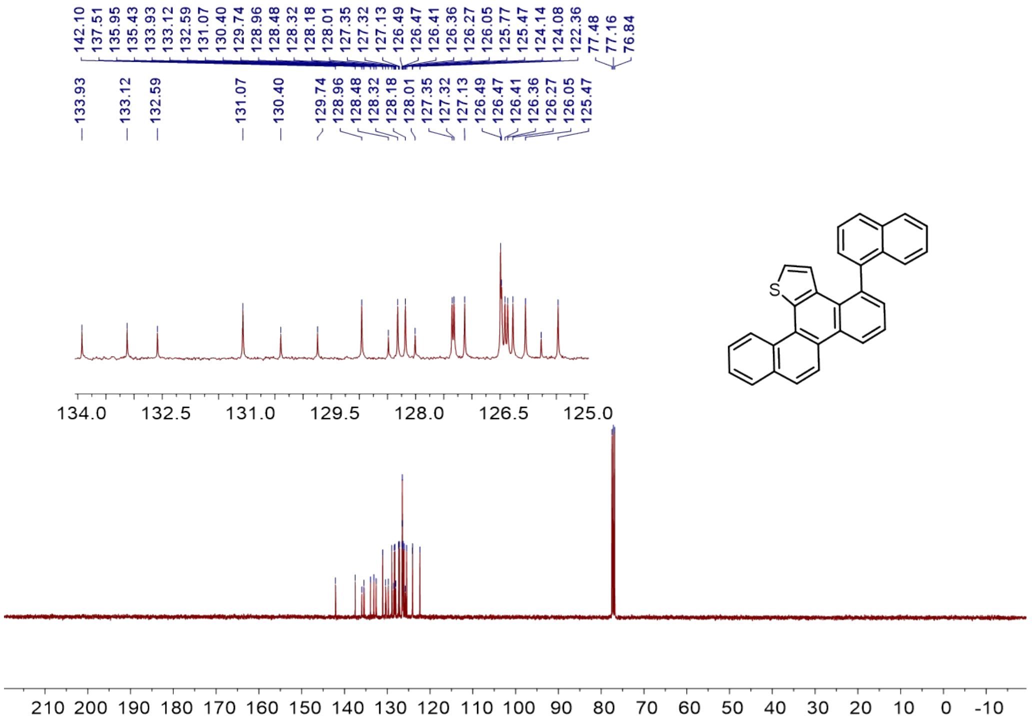

Figure S174. ^{13}C NMR (100 MHz, $CDCl_3$) spectrum of **8b**.

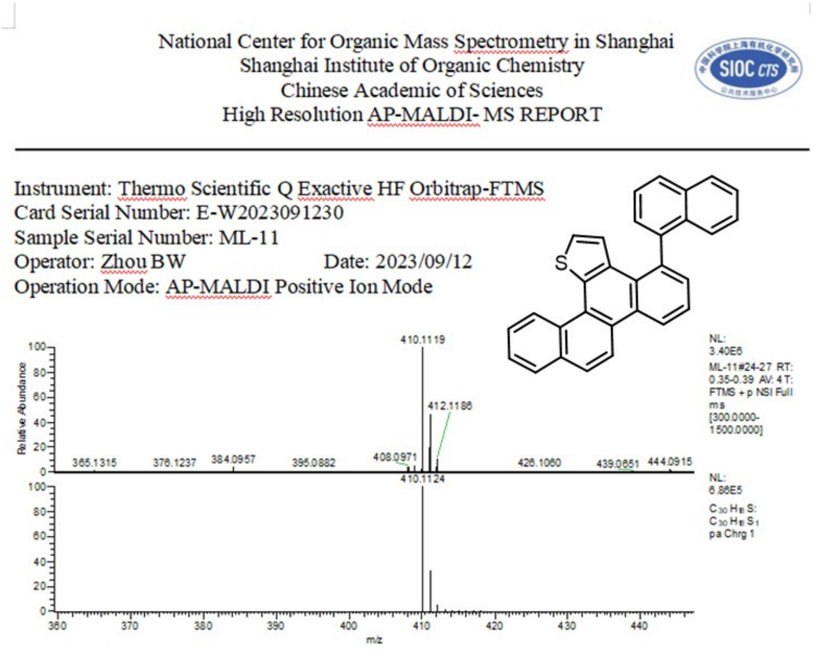

m/z	Theo. Mass	Delta (ppm)	RDB equiv.	Composition
410.1119	410.1124	-1.15	22.0	$C_{30}H_{18}S$

Figure S175. HRMS-MALDI spectrum of **8b**.

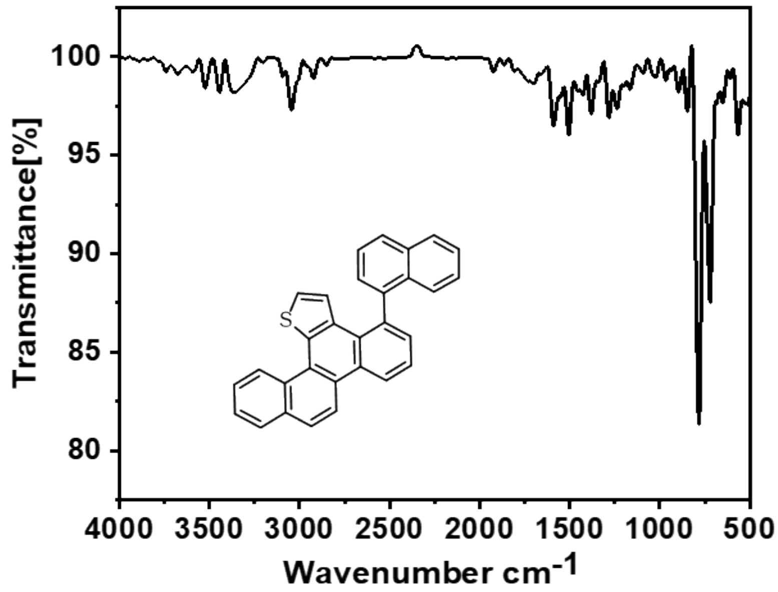

Figure S176. IR spectrum of **8b**.

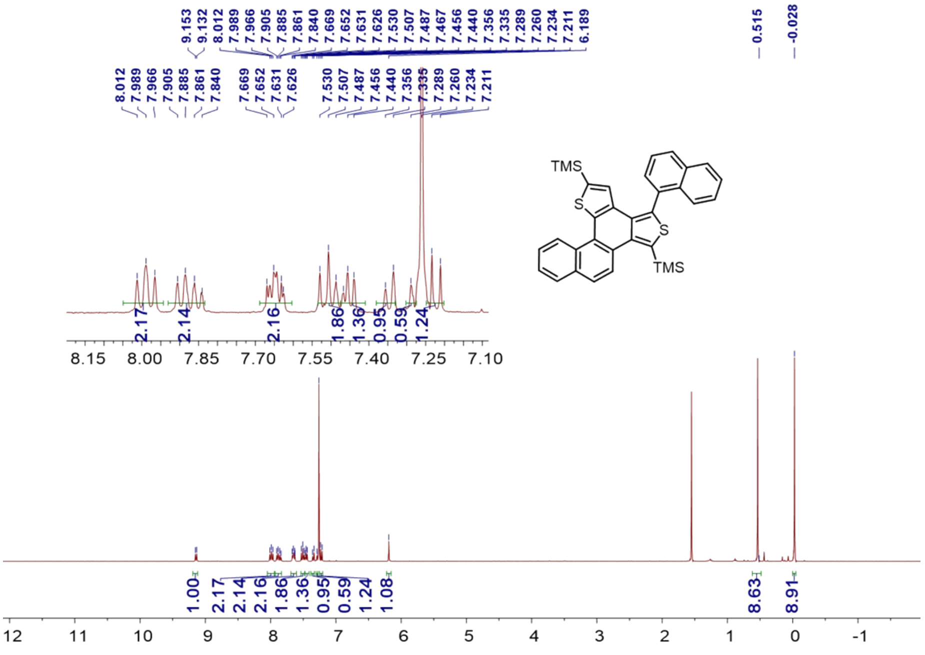

Figure S177. ^{1}H NMR (400 MHz, $CDCl_3$) spectrum of **8c**.

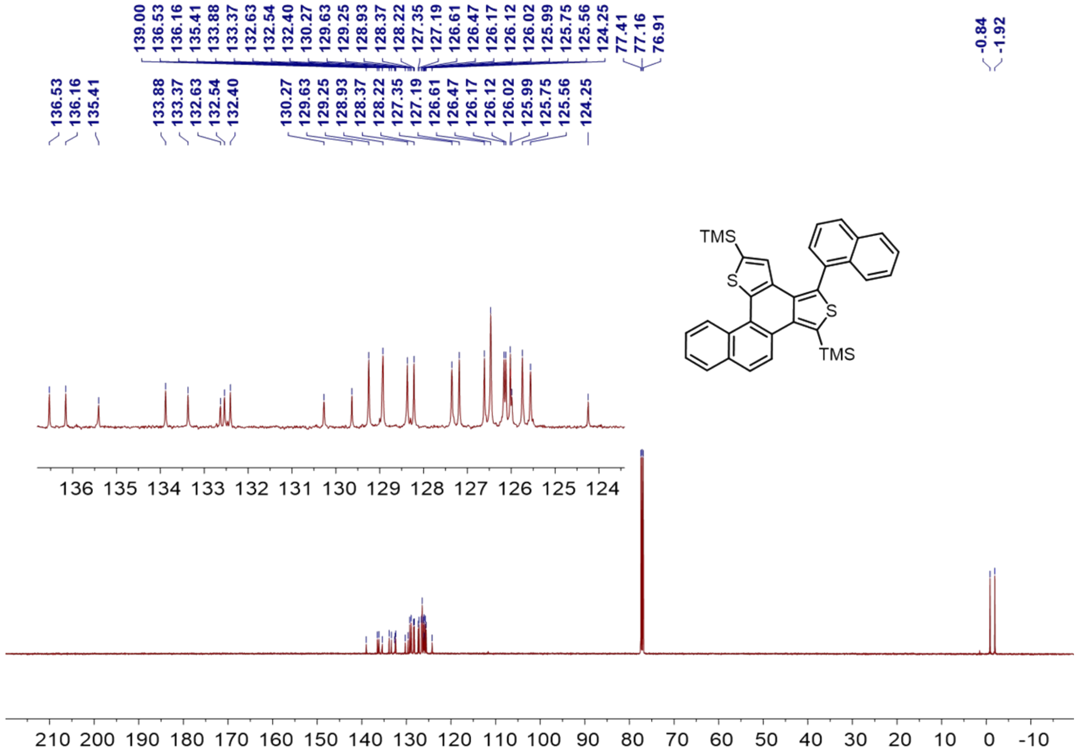

Figure S178. ^{13}C NMR (100 MHz, $CDCl_3$) spectrum of **8c**.

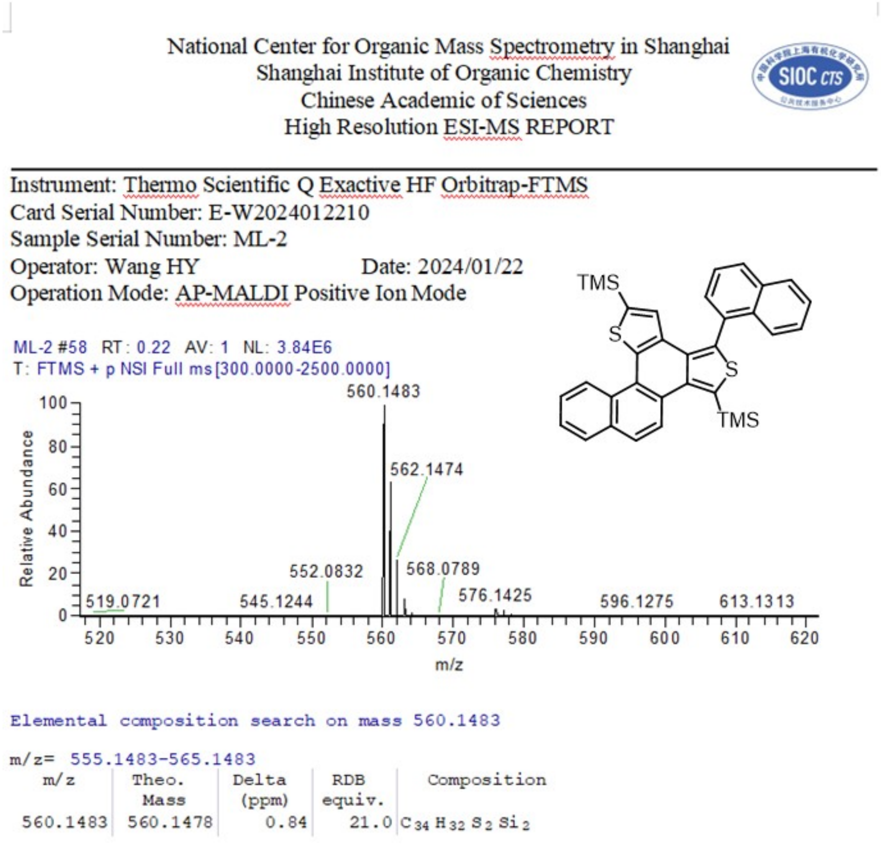
National Center for Organic Mass Spectrometry in Shanghai
Shanghai Institute of Organic Chemistry
Chinese Academic of Sciences
High Resolution ESI-MS REPORT

Instrument: Thermo Scientific Q Exactive HF Orbitrap-FTMS
Card Serial Number: E-W2024012210
Sample Serial Number: ML-2
Operator: Wang HY Date: 2024/01/22
Operation Mode: AP-MALDI Positive Ion Mode

Elemental composition search on mass 560.1483

m/z= 555.1483-565.1483

m/z	Theo. Mass	Delta (ppm)	RDB equiv.	Composition
560.1483	560.1478	0.84	21.0	$C_{34}H_{32}S_2Si_2$

Figure S179. HRMS-MALDI spectrum of **8c**.

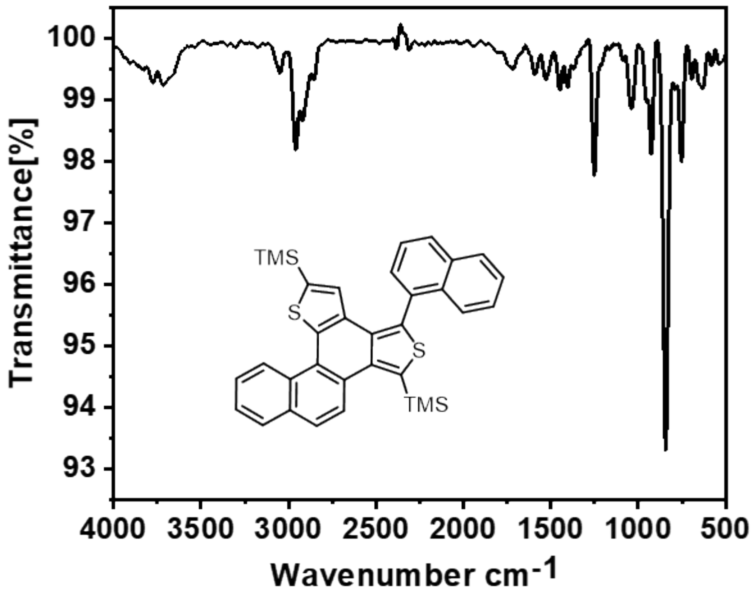

Figure S180. IR spectrum of **8c**.

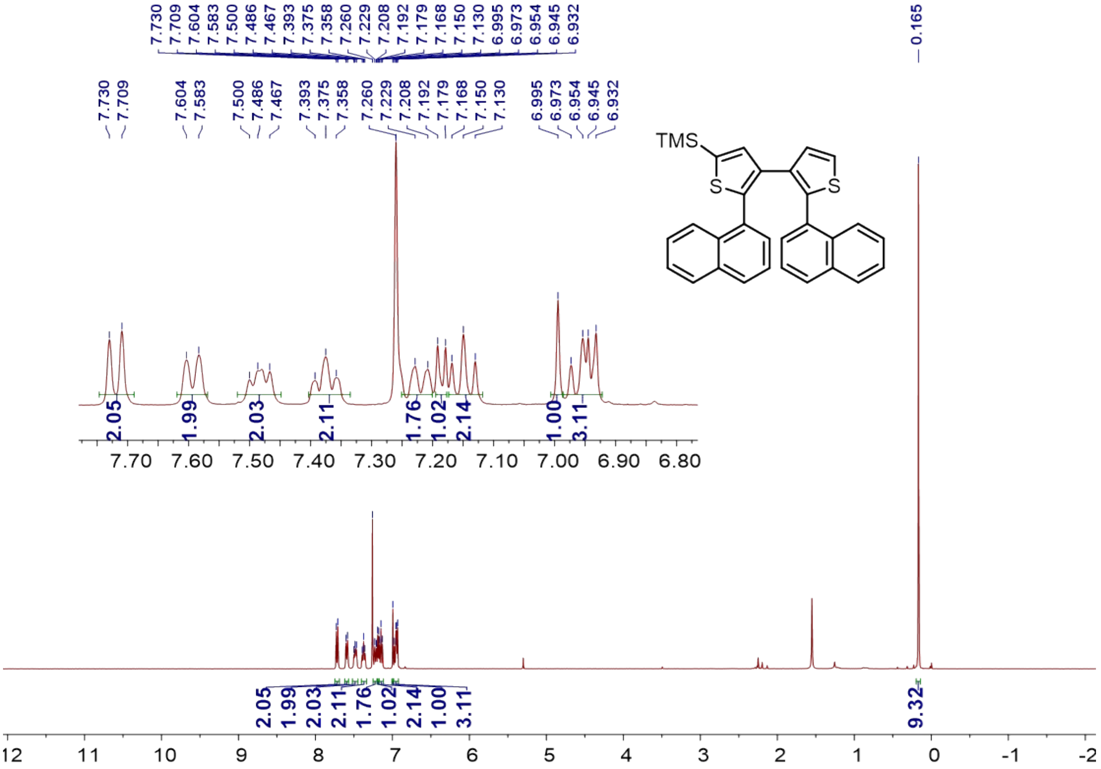

Figure S181. ^{1}H NMR (400 MHz, $CDCl_3$) spectrum of **7d**.

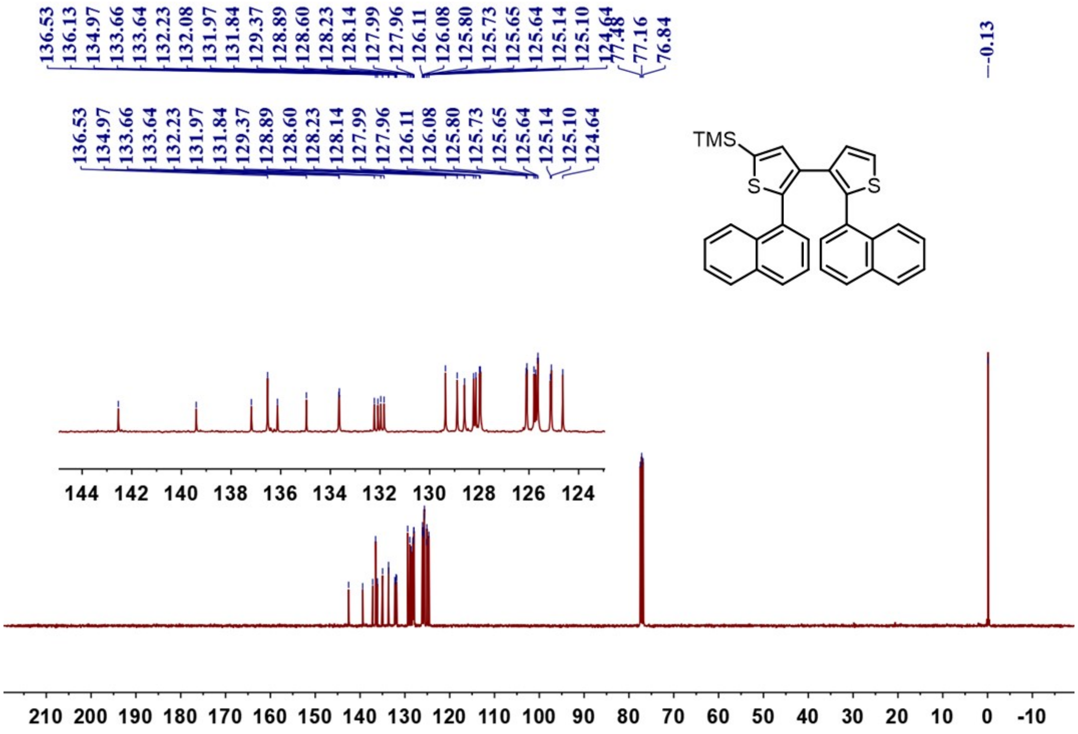

Figure S182. ^{13}C NMR (100 MHz, $CDCl_3$) spectrum of **7d**.

National Center for Organic Mass Spectrometry in Shanghai
Shanghai Institute of Organic Chemistry
Chinese Academic of Sciences
High Resolution MS DATA REPORT

Instrument: Thermo Fisher Scientific LTQ FTICR-MS
Card Serial Number : D20241660
Sample Serial Number: ML-1
Operator : DONG Date: 2024/03/13
Operation Mode: DART POSITIVE

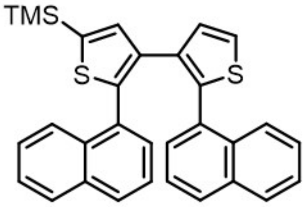

Elemental composition search on mass 491.1322

m/z= 486.1322-496.1322

m/z	Theo. Mass	Delta (ppm)	RDB equiv.	Composition
491.1322	491.1318	0.72	19.5	$C_{31}H_{27}S_2Si$
	491.1327	-1.17	16.0	$C_{24}H_{25}O_5N_3Si_2$
	491.1316	1.19	19.5	$C_{30}H_{27}OSSi_2$
	491.1330	-1.64	16.0	$C_{25}H_{25}O_4N_3SSi$
	491.1312	2.02	20.5	$C_{31}H_{23}O_4S$
	491.1332	-2.11	16.0	$C_{26}H_{25}O_3N_3S_2$
	491.1309	2.49	20.5	$C_{30}H_{23}O_5Si$
	491.1309	2.52	10.5	$C_{22}H_{31}O_3N_2S_2Si_2$
	491.1336	-2.94	15.0	$C_{25}H_{29}N_3S_2Si_2$
	491.1336	-2.97	25.0	$C_{33}H_{21}O_2NSi$

Figure S183. HRMS-DART spectrum of **7d**.

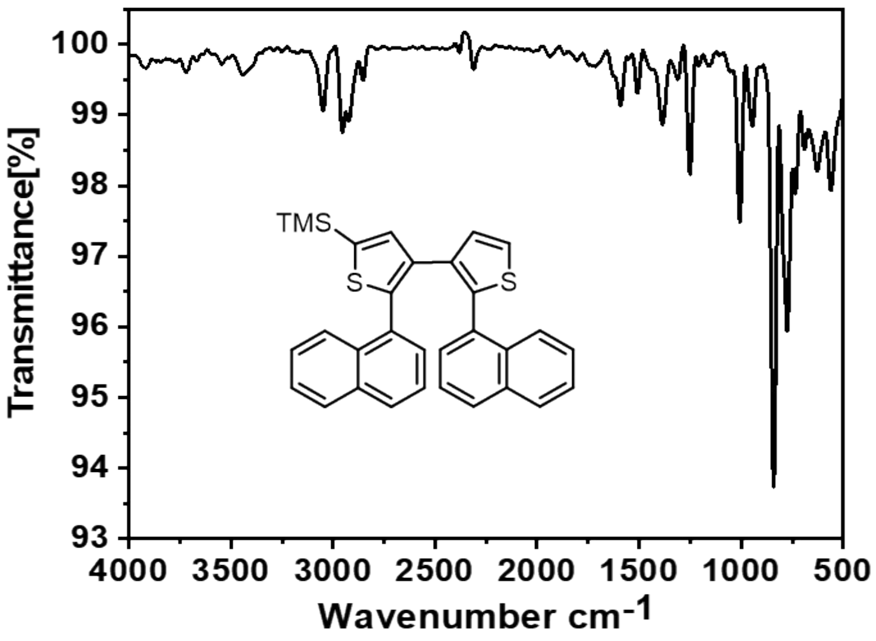

Figure S184. IR spectrum of **7d**.

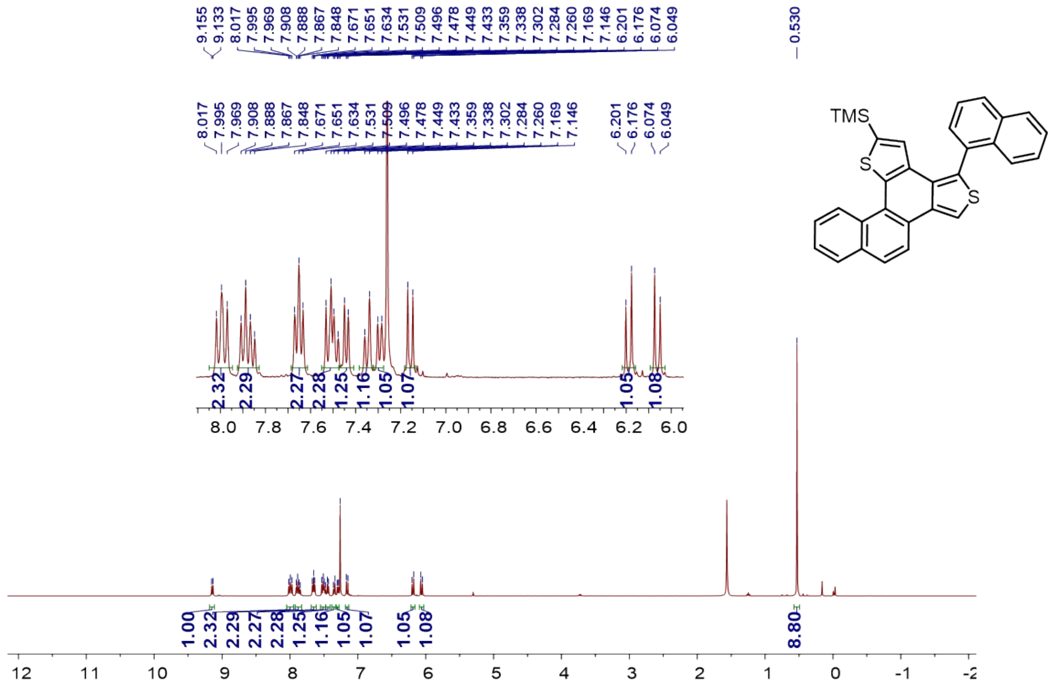

Figure S185. ^{1}H NMR (400 MHz, $CDCl_3$) spectrum of **8d**.

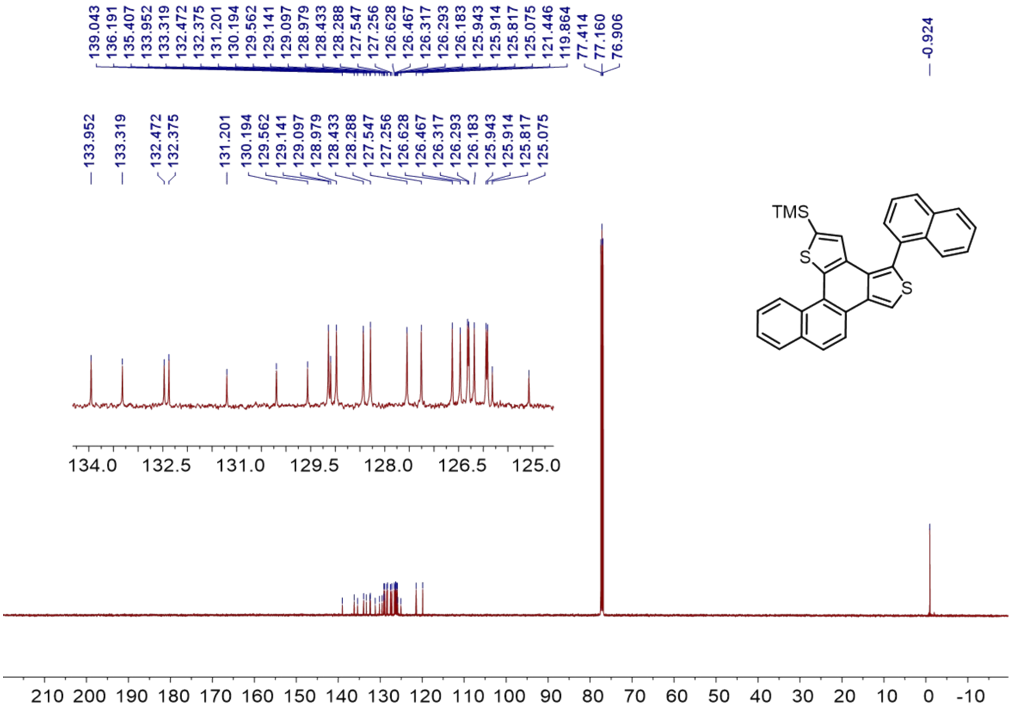

Figure S186. ^{13}C NMR (100 MHz, $CDCl_3$) spectrum of **8d**.

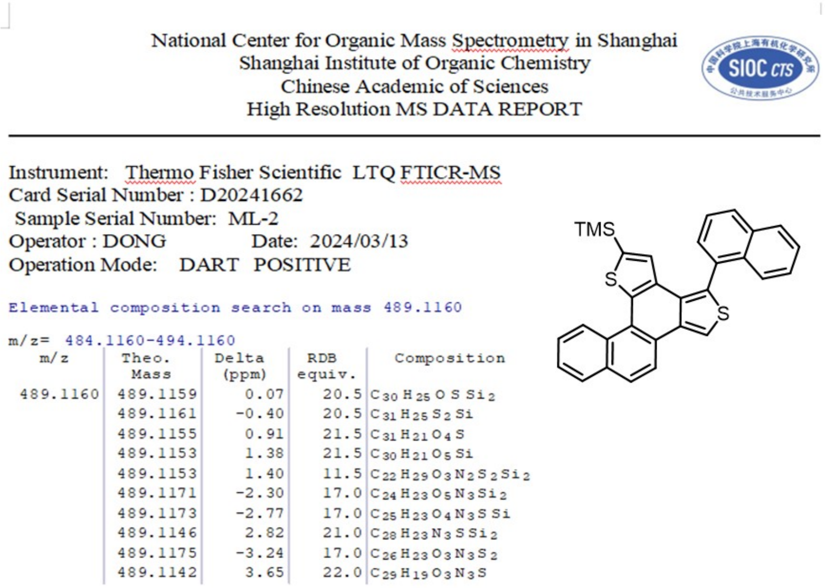

National Center for Organic Mass Spectrometry in Shanghai
Shanghai Institute of Organic Chemistry
Chinese Academic of Sciences
High Resolution MS DATA REPORT

Instrument: Thermo Fisher Scientific LTQ FTICR-MS
Card Serial Number : D20241662
Sample Serial Number: ML-2
Operator : DONG Date: 2024/03/13
Operation Mode: DART POSITIVE

Elemental composition search on mass 489.1160

m/z= 484.1160-494.1160

m/z	Theo. Mass	Delta (ppm)	RDB equiv.	Composition
489.1160	489.1159	0.07	20.5	$C_{30}H_{25}OSSi_2$
	489.1161	-0.40	20.5	$C_{31}H_{25}S_2Si$
	489.1155	0.91	21.5	$C_{31}H_{21}O_4S$
	489.1153	1.38	21.5	$C_{30}H_{21}O_5Si$
	489.1153	1.40	11.5	$C_{22}H_{29}O_3N_2S_2Si_2$
	489.1171	-2.30	17.0	$C_{24}H_{23}O_5N_3Si_2$
	489.1173	-2.77	17.0	$C_{25}H_{23}O_4N_3SSi$
	489.1146	2.82	21.0	$C_{28}H_{23}N_3SSi_2$
	489.1175	-3.24	17.0	$C_{26}H_{23}O_3N_3S_2$
	489.1142	3.65	22.0	$C_{29}H_{19}O_3N_3S$

Figure S187. HRMS-DART spectrum of **8d**.

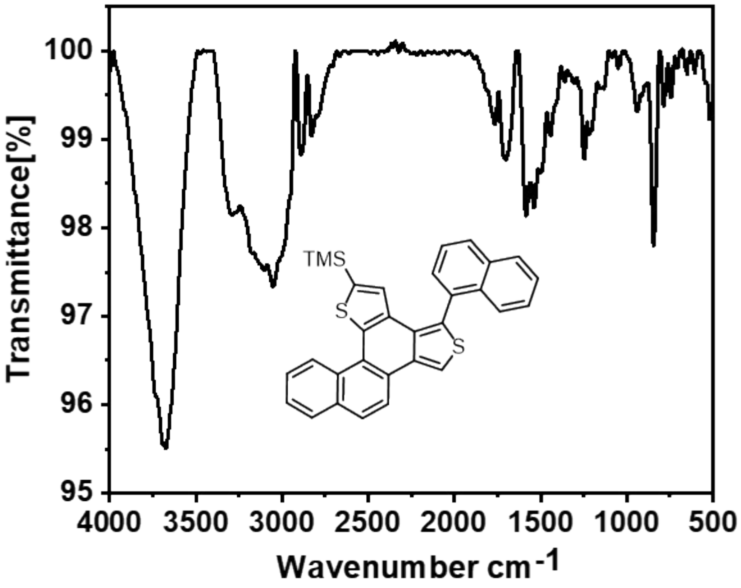

Figure S188. IR spectrum of **8d**.

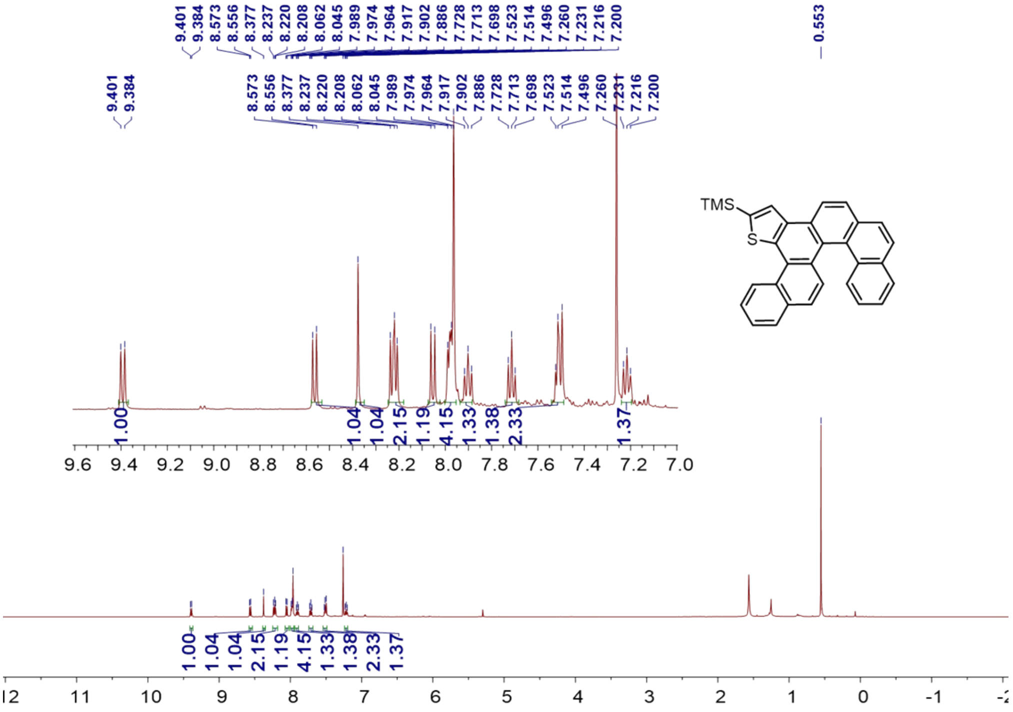

Figure S189. ^{1}H NMR (400 MHz, $CDCl_3$) spectrum of **8e**.

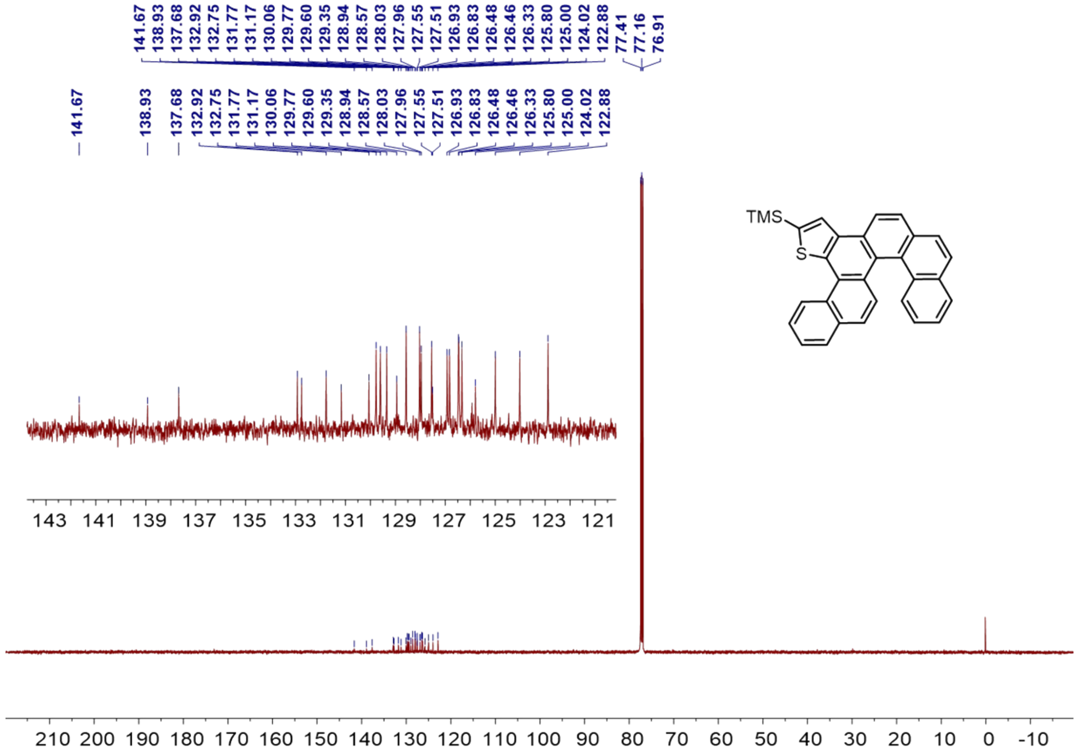

Figure S190. ^{13}C NMR (100 MHz, $CDCl_3$) spectrum of **8e**.

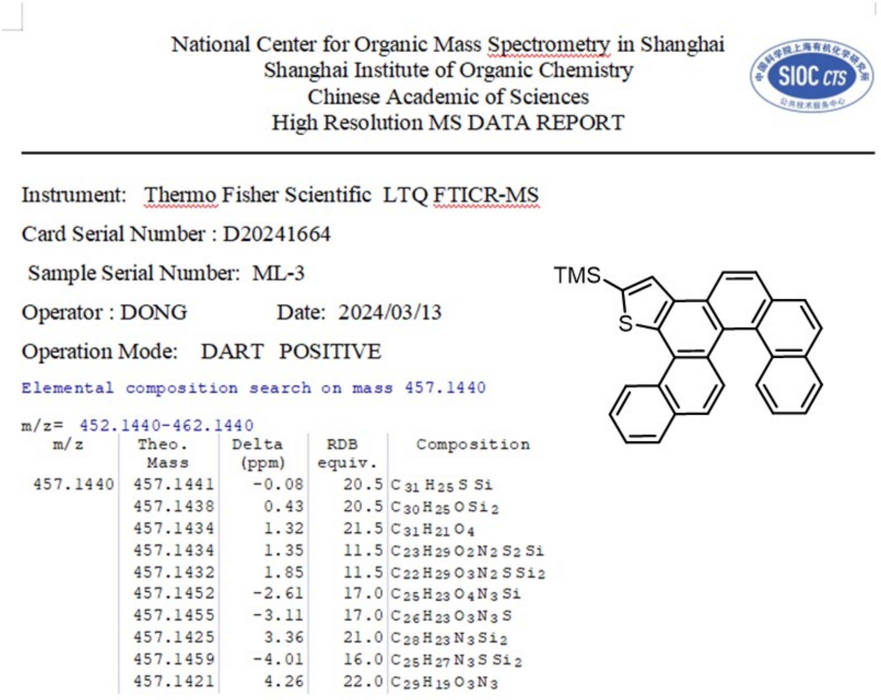
National Center for Organic Mass Spectrometry in Shanghai
Shanghai Institute of Organic Chemistry
Chinese Academic of Sciences
High Resolution MS DATA REPORT

Instrument: Thermo Fisher Scientific LTQ FTICR-MS

Card Serial Number : D20241664

Sample Serial Number: ML-3

Operator : DONG Date: 2024/03/13

Operation Mode: DART POSITIVE

Elemental composition search on mass 457.1440

m/z= 452.1440-462.1440

m/z	Theo. Mass	Delta (ppm)	RDB equiv.	Composition
457.1440	457.1441	-0.08	20.5	$C_{31}H_{25}S\,Si$
	457.1438	0.43	20.5	$C_{30}H_{25}O\,Si_2$
	457.1434	1.32	21.5	$C_{31}H_{21}O_4$
	457.1434	1.35	11.5	$C_{23}H_{29}O_2N_2S_2Si$
	457.1432	1.85	11.5	$C_{22}H_{29}O_3N_2S\,Si_2$
	457.1452	-2.61	17.0	$C_{25}H_{23}O_4N_3Si$
	457.1455	-3.11	17.0	$C_{26}H_{23}O_3N_3S$
	457.1425	3.36	21.0	$C_{28}H_{23}N_3Si_2$
	457.1459	-4.01	16.0	$C_{25}H_{27}N_3S\,Si_2$
	457.1421	4.26	22.0	$C_{29}H_{19}O_3N_3$

Figure S191. HRMS-DART spectrum of **8e**.

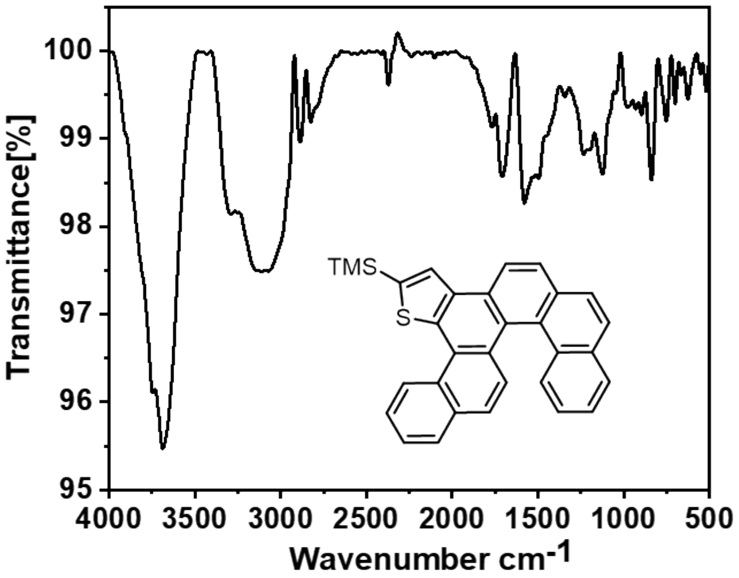

Figure S192. IR spectrum of **8e**.

10. Reference

S1. Suffert J. Simple direct titration of organolithium reagents using N-pivaloyl-o-toluidine and/or N-pivaloyl-o-benzylaniline. *J. Org. Chem.* **1989**, *54*, 509–510.

S2. Wang Y, Wang Z, Zhao D, Wang Z, Cheng Y, Wang H. Efficient Synthesis of Trimethylsilyl-Substituted Dithieno[2,3-b:3′,2′-d]thiophene, Tetra[2,3-thienylene] and Hexa[2,3-thienylene] from Substituted [3,3′]Bithiophenyl. *Synlett*, **2007**, *15*, 2390–2394.

S3. Uematsu K, Noguchi K, Nakano K. Synthesis and properties of [7]helicene and [7]helicene-like compounds with a cyclopenta-[1,2-b:4,3-b0]dithiophene or dithieno-[2,3-*b*:30,20-*d*]heterole skeleton. *Phys. Chem. Chem. Phys.*, **2018**, *20*, 3286–3295.

S4. Wang Y, Song J, Xu L, Kan Y, Shi J, Wang H. Synthesis and Characterization of Cyclooctatetrathiophenes with Different Connection Sequences. *J. Org. Chem.*, **2014**, *79*, 2255-2262.

S5. Li C, Shi J, Xu L, Wang Y, Cheng Y, Wang H. Syntheses and Crystal Structures of Fused Thiophenes: [7]Helicene and Double Helicene, a D2-Symmetric Dimer of 3,3′-Bis(dithieno[2,3-*b*:3′,2′-*d*]thiophene). *J. Org. Chem.*, **2009**, *74*, 408–411.

S6. Campo M. A, Larock R. C. Synthesis of Fluoren-9-ones by the Palladium-Catalyzed Cyclocarbonylation of o-Halobiaryls. *J. Org. Chem.,* **2002**, *67*, 5616–5620.
